# Influence of Polysorbate 80 on the Larvicidal and Ecotoxicological Profile of Essential Oil Nanoemulsion: Insights into Green Nanotechnology

**DOI:** 10.1021/acs.jafc.5c04690

**Published:** 2025-07-24

**Authors:** Clenilma M. Brandão, Djanira R. dos Santos, Lucas G. P. Silva, Mirla C. Ferreira, Joyce M. de F. Mesquita, Melissa P. Souza, Carlos A. Holanda, Renato S. Gonçalves, Emmanoel V. Costa, Georgiana E. de C. Marques, Rogério de M. Teles, Kiany S. B. Cavalcante

**Affiliations:** † Postgraduate Program in Chemistry, Associative Doctorate UFMA-IFMA, Federal Institute of Maranhão, São Luís-Monte Castelo Campus, São Luís, MA 65030-005, Brazil; ‡ Postgraduate Program in Chemistry, Federal Institute of Maranhão, São Luís-Monte Castelo Campus, São Luís, MA 65030-005, Brazil; § Laboratory of Microbiological Analysis, Department of Chemistry, Federal Institute of Maranhão, São Luís-Monte Castelo Campus, São Luís, MA 65030-005, Brazil; ∥ Postgraduate Program in Chemistry, Federal University of Amazonas, Manaus, AM 69080-900, Brazil; ⊥ Natural Sciences Degree Coordination, Federal University of Maranhão, Bom Jesus Campus, Imperatriz, MA 65915-060, Brazil; # Department of Chemistry, Postgraduate Program in Chemistry, Federal University of Maranhão, São Luís Campus, São Luís, MA 65080-805, Brazil; ∇ Department of Chemistry, Postgraduate Program in Chemistry, Federal University of Amazonas, Manaus, AM 69080-900, Brazil

**Keywords:** arbovirus vectors, biolarvicide, Aedes albopictus mosquito, simplex centroid design, chemical markers, Cerrado of Maranhão, A. salina microcrustaceans, nanobiotechnology

## Abstract

Essential oil (EO) nanoformulations are emerging as green nanotechnology strategies against and s mosquitoes, which are vectors of arboviruses such as dengue. For the first time, we investigated the larvicidal influence of polysorbate (PS_80_) against mosquito using simplex centroid design (SCD) in the formulation of EO*Dr* nanoemulsions derived from the species (Plantaginaceae), a plant native to the Cerrado region of Maranhão, Brazil. In addition, we present the ecotoxicological profile of the formulation against microcrustaceans. EO*Dr* was extracted by hydrodistillation, and its chemical profile was analyzed by GC-MS-FID and NMR (^1^H, ^13^C, DEPT-135, and 2D). The nanoformulations were characterized by DLS, PDI, ZP, and TEM. The chemical profile indicated the presence of compounds such as fenchol, fenchyl acetate, caryophyllene, and caryophyllene oxide. The SCD contributed to the development of nanostructured formulations with spheroidal morphology, demonstrating effective larvicidal action against mosquitoes with lower toxicity than commercial chemical larvicides.

## Graphical abstract

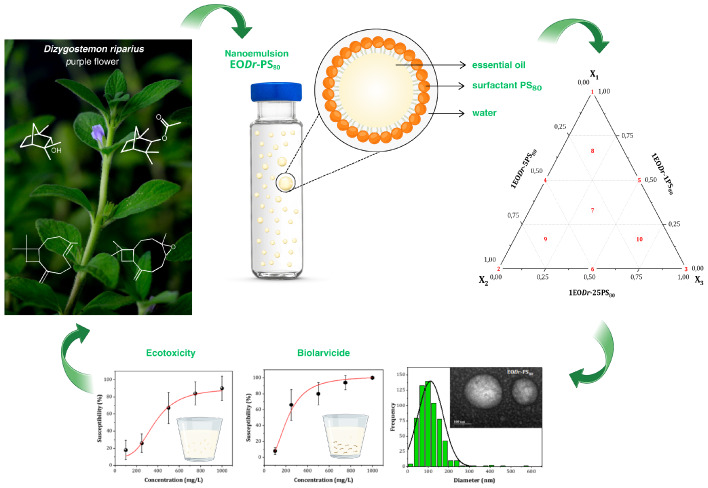

## Introduction

Emerging and reemerging arboviruses represent a global public health challenge. Dengue, chikungunya, and Zika viruses are primarily transmitted by and mosquitoes.^1,2^ This is a global concern, but especially in Brazil and Maranhão, where vaccination coverage is still expanding and the recurrent increase in epidemic cases raises concerns and requires urgent measures to prevent and combat the mosquito vectors.^3^ The main control strategies are the use of larvicides and synthetic insecticides.^4^ However, the repeated use of these synthetic compounds has contributed to the selection of resistant populations of *Aedes* mosquito species.^5^

In this way, the search for natural larvicides has proven to be a promising alternative strategy, with essential oils (EOs) standing out, since the complex mixture of bioactives in EOs includes classes of metabolites that give plants protection and reduce resistance against attacks by pathogens and insects.^6^

The bioactivity of EOs can be attributed to their complex mixture mainly composed of monoterpenes, sesquiterpenes, and their oxygenated derivatives.^7,8^ The biological activity of EOs against *Aedes* mosquitoes is associated with multiple mechanisms, depending on the target action, from digestive toxicity to enzyme inhibition and toxicity to the nervous system in the larval stage. They are therefore promising, effective, and potentially environmentally safe alternatives to current synthetic larvicides.^9–11^

However, the limited availability of raw materials, low yield, high volatility, and low miscibility of EOs in aqueous media make their application in natura unfeasible. In this context, the use of surfactants is necessary, with nonionic surfactants being the most viable for use with EOs, as they reduce changes in their properties and promote the formation of micellar and/or vesicular structures with greater efficiency on a micro and/or nanoemulsion scale.^12,13^

In this context, the study focuses on the chemical composition of the EO of the plant species identified in the Cerrado biome region in the state of Maranhão, with two floral morphotypes, lilac and white, registered as Scatigna and Colletta (Plantaginaceae),^14^ characterized the chemical profile of the EO of (EO*Dr*) as terpenic and evaluated the larvicidal action against larvae of the mosquito, with this action being attributed to the EO matrix consisting of the major chemotypes, fenchyl acetate and fenchol.^15^

In the above-mentioned study, polysorbate 80 (PS_80_), a nonionic surfactant, was used in the composition of the emulsified system. Although PS_80_ is widely used in the preparation of emulsions, microemulsions and nanoemulsions, optimal concentration for formulations of mixtures with EOs is not well-defined in the literature.^16–18^

In addition to the search for emulsified systems and promising strategies to enhance the action of the bioactives present in EO*Dr*, the use of design of experiments (DOE), such as simplex centroid design (SCD), are of paramount importance to understand the interaction between the constituents of emulsified systems, in addition to increasing target-biocidal action and decreasing biological resistance.^19^

The SCD is a mathematical-statistical approach to mixture design aimed at developing and optimizing analytical responses. The adoption of this approach has proven to be methodologically effective for modeling the combined biological activities of different secondary metabolites.^20,21^

Furthermore, this type of experimental design is of great practical interest, as it involves a minimum number of experiments, the development of improved and/or innovative formulations that provide targeted responses and highlight general aspects of the interactions between independent factors.^22–24^

The article presents the formulation and characterization of EO*Dr* nanoemulsion systems and the investigation of the influence of PS_80_ on the larvicidal and ecotoxicological activity of emulsified EO*Dr* mixtures, using the SCD methodology, as well as their EO*Dr*-PS_80_ interactions.

## Experimental Section

### Collection of Plant Material and Essential Oil Extraction

Plant material from the lilac morphotype of (Plantaginaceae) was collected in São Benedito do Rio Preto, Maranhão, Brazil (03°19′27.9’“S; 43°31′02.6”’W). A voucher specimen (SLUI 8656) was deposited in the Rosa Mochel Herbarium and registered with SISGEN (A5E5CD0).

The EO*Dr* was extracted by hydrodistillation (Ultrathermostatic bath; SSDu 10 L) using green solvent, water in the recycling system.^15,25,26^ Detailed methodology is provided in the Supporting Information.

### Chemical Composition: CG-FID and GC-MS

The GC-FID analysis was conducted using a Shimadzu GC-17A. The GC-MS analysis was performed using a Thermo Scientific Trace Ultra GC coupled to an ISQ MSQ.^27,28^ Detailed methodology is provided in the Supporting Information.

### Chemical Composition: NMR ^1^H and ^13^C

The NMR spectra of the EO*Dr* sample and the chemical standards of the main compounds, including 1D (^1^H, ^13^C, DEPT-135) and 2D (COSY, HSQC, HMBC), were recorded using a BRUKER AVANCE III HD spectrometer (Billerica, MA, USA), operating at 11.75 T (500.13 MHz for ^1^H NMR and 125.76 MHz for ^13^C NMR). Detailed methodology is provided in the Supporting Information.

### Nanoemulsions Formulation

The nanoemulsions were obtained by the low-energy oil–water emulsification method (Figure 1) without the use of specialized equipment.^29^ The steps of preparation of the mixtures involved some of the 12 principles of green chemistry.^30^

**Figure 1:**
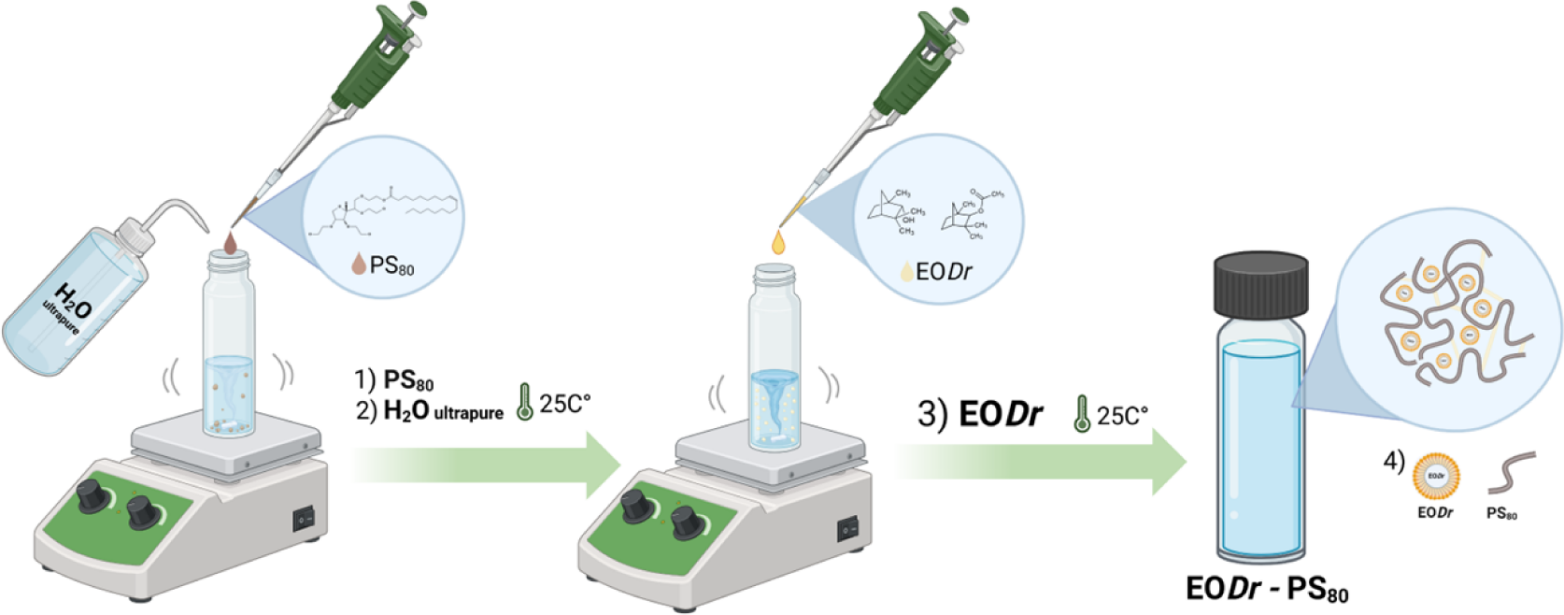
General scheme for the preparation of EO*Dr*-PS_80_ nanoformulations.

To determine the role of PS_80_ in the EO*Dr*-PS_80_ interaction, the concentration of EO*Dr* in the mixtures was kept constant (Figure 2).

**Figure 2:**
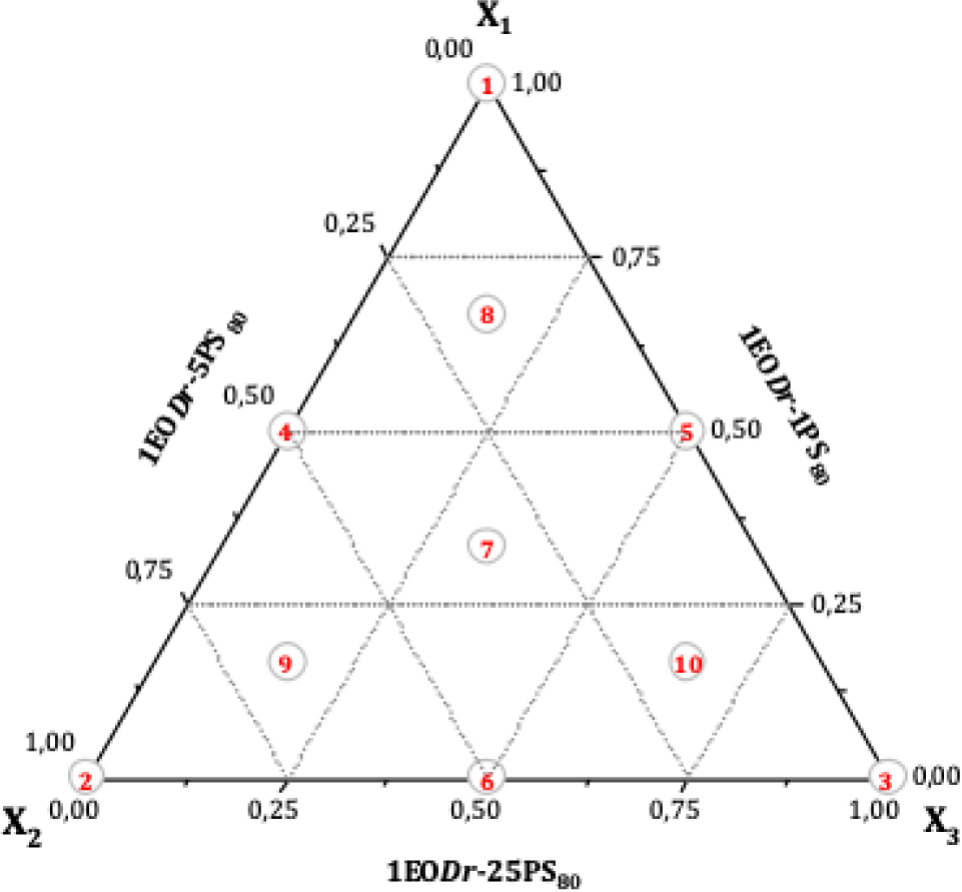
Distribution of EO*Dr*-PS_80_ interaction points in the SCD. Vertex points (1, 2, and 3); edge points (4, 5, and 6); center point (7); and face points (8, 9, and 10).

The proportions for emulsification^31^ were: 1EO*Dr*-1PS_80_ (X_1_); 1EO*Dr*-5PS_80_ (X_2_) and 1EO*Dr*-25PS_80_ (X_3_) g·L^–1^. Detailed methodology is provided in the Supporting Information.

### Hydrodynamic Diameter, PDI, and ZP

The hydrodynamic diameter, polydispersity index (PDI) were evaluated using the dynamic light scattering technique and zeta potential (ZP) was measured using the electrophoretic light scattering technique, both on a Zetasizer Nano ZS90 (Malvern). Detailed methodology is provided in the Supporting Information.

### Morphology and Size by TEM

The mean diameter and morphology were analyzed using a Transmission Electron Microscope (TEM), JEM-2100 (JEOL, Tokyo, Japan) equipped with EDS, Thermo Scientific; Lanthanum hexaboride filament electron beam (LaB_6_); Acceleration voltage of 200 kV, with a resolution of 2.5 Å resolution; ORIUS SC 1000 CCD camera, Gatan brand; Digital Micrograph software; Energy dispersive spectroscopy (EDS) with Thermo Scientific NSS Spectral Imaging detector for elemental identification. Detailed methodology is provided in the Supporting Information.

### Larvicidal Bioassays

The larvicidal bioassays of the EO*Dr*-PS_80_ emulsified systems against mosquito larvae followed the methodology recommended by the WHO with adaptations.^15,32–34^ Detailed methodology is provided in the Supporting Information.

### Ecotoxicity Tests

Aquatic ecotoxicology tests followed the protocols for toxicity tests standardized by the Brazilian Association of Technical Standards (ABNT), NBR 16530:2016 ^35^ against microcrustaceans, meeting the requirements for the competence of chemical testing laboratories, based on ABNT, NBR ISO IEC 17025:2017.^36^ Detailed methodology is provided in the Supporting Information.

### Statistical Analysis

The results were expressed as mean, standard deviation and mortality percentage values. Data analysis was performed using Origin software (version 8.5) and R software (version 4.2.3) to determine lethal concentrations (LC_50_ and LC_90_) and the adjusted coefficient of determination (R^2^_Adj_) with a 95% confidence interval (*p* < 0.05).

The larvicidal efficacy of EO*Dr*-PS_80_ against *s* larvae was determined by obtaining contour plots. The LC_50_ and LC_90_ values were obtained from the replicates in the SCD, the replicates being necessary for the analysis of variance (ANOVA).^37,38^ Detailed methodology is provided in the Supporting Information.

## Results and Discussion

### Chemical Profile: GC-MS and CG-FID

The chemical profiling of the EO*Dr* matrix confirmed the plant species’ chemical composition and allowed us to associate the interactions between its constituents with the observed biological responses.

Table 1 shows the chemical composition via GC-MS and CG-FID of the EO*Dr* matrix, lilac floral morphotype, as well as the analysis of the chemical patterns (Sigma-Aldrich) of the four chemotypes in relative percentage (Figures S1–S13).

**Table 1:** Chemical Composition of EO*Dr*, Lilac Morphotype, and Patterns of the Major Chemotypes^a^

peaks	EO*Dr* compounds	RT_min_	RI_Calc_	RI_Lit_	%	MF	TC
1	α-pinene	10.70	928	932	0.08	C_10_H_16_	M
2	α-fenchene	11.29	944	945	0.08	C_10_H_16_	M
3	sabinene	12.32	969	969	0.02	C_10_H_16_	M
4	α-terpinene	14.11	1014	1014	0.02	C_10_H_16_	M
5	p-cymene	14.41	1022	1020	0.13	C_10_H_14_	M
6	limonene	14.59	1026	1024	0.12	C_10_H_16_	M
7	1,8-cineole	14.70	1029	1026	0.11	C_10_H_18_O	OM
8	γ-terpinene	15.77	1056	1054	0.07	C_10_H_16_	M
9	fenchone	16.93	1085	1083	0.14	C_10_H_16_O	OM
10	fenchol <endo>	18.23	1119	1114	42.97	C_10_H_18_O	OM
11	α-terpineol	21.01	1194	1186	0.07	C_10_H_18_O	OM
12	fenchyl acetate <endo>	21.86	1217	1218	46.03	C_12_H_20_O_2_	OM
13	(E)-caryophyllene	28.61	1416	1417	4.81	C_15_H_24_	S
14	α-humulene	29.72	1452	1452	0.38	C_15_H_24_	S
15	caryophyllene oxide	33.56	1579	1582	1.94	C_15_H_24_O	OS
16	humulene epoxide II	34.36	1606	1608	0.27	C_15_H_24_O	OS
17	caryophylla-4(12),8(13)-dien-5α-ol	35.05	1631	1639	0.29	C_15_H_24_O	OS
18	caryophylla-4(12),8(13)-dien-5β-ol	35.14	1634	1639	1.00	C_15_H_24_O	OS
19	pogostol	35.71	1654	1639	0.24	C_15_H_26_O	OS
	Σ monoterpenes (M)				0.52		
	Σ oxygenated monoterpenes (OM)				89.32		
	Σ sesquiterpenes (S)				5.19		
	Σ oxygenated sesquiterpenes (OS)				3.74		
	Σ Total identified				98.77		
**Standard compounds**							
1	fenchol <endo>	18.42	1124	1114	94.82	C_10_H_18_O	OM
2	fenchyl acetate <endo>	21.97	1221	1218	98.87	C_12_H_20_O_2_	OM
3	(E)-caryophyllene	28.87	1425	1417	97.98	C_15_H_24_	S
4	caryophyllene oxide	33.77	1586	1582	98.26	C_15_H_24_O	OS

a P = chromatogram peaks (Figures S1, S6, S8, S10, and S12); RT_min_ = retention time; RI_Calc._ = calculated retention indices (on TR-5MS capillary column 30 m × 0.25 mm × 0.25 μm) according to van Den Dool and Kratz,^27^ based on a homologous series of normal alkanes; RI_Lit._ = literature retention indices, Adams;^28^ % = relative percentage; MF = molecular formula; TC = terpene classes.

The chemical matrix of EO*Dr* shows a terpene profile, displaying chemical compound classes of monoterpenes, oxygenated monoterpenes, sesquiterpenes and oxygenated sesquiterpenes, totaling 98.77% of the relative percentage of chemical constituents. The predominance of oxygenated monoterpenes fenchol and fenchyl acetate is significant, totaling 89.0% of the EO*Dr* matrix.

The chemical profile observed in the present study is in line with the results reported by Brandão and collaborators,^15^ as well as Galvão and associates^39^ who observed the terpene pattern of the EO of the same plant species, with emphasis on the chemotypes of the species, oxygenated monoterpenes, endo fenchol, 42.97%; endo fenchyl acetate, 46.03%; sesquiterpenes; (E)-caryophyllene, 4.81% and oxygenated sesquiterpenes, caryophyllene oxide, 1.94%.

The percentage variations observed in the chemical composition compared to previously published studies are due to circadian, seasonal and edaphoclimatic conditions, as the yield and chemical composition of EOs are associated with rainfall, relative humidity, solar radiation, atmospheric constitution and variations in temperature range, wind, soil and relief.^40–44^

Several studies suggest that the terpenic pattern of various chemical matrices of EOs extracted from different botanical families is correlated with high larvicidal activity against and larvae.^45,46^ In this respect, studies indicate that the bioactives in the essential oil and crude extracts of the lilac morphotype of the species from the Maranhão Cerrado, , have effective larvicidal activity, respectively, against the species and .^15,47^

### Chemical Profile: ^1^H NMR and ^13^C NMR

The ^1^H NMR data (Table 2) provide crucial insights into the differentiation of the four major compounds in the EO*Dr* sample: fenchol, fenchyl acetate, caryophyllene, and caryophyllene oxide (Figure 3).

**Figure 3:**
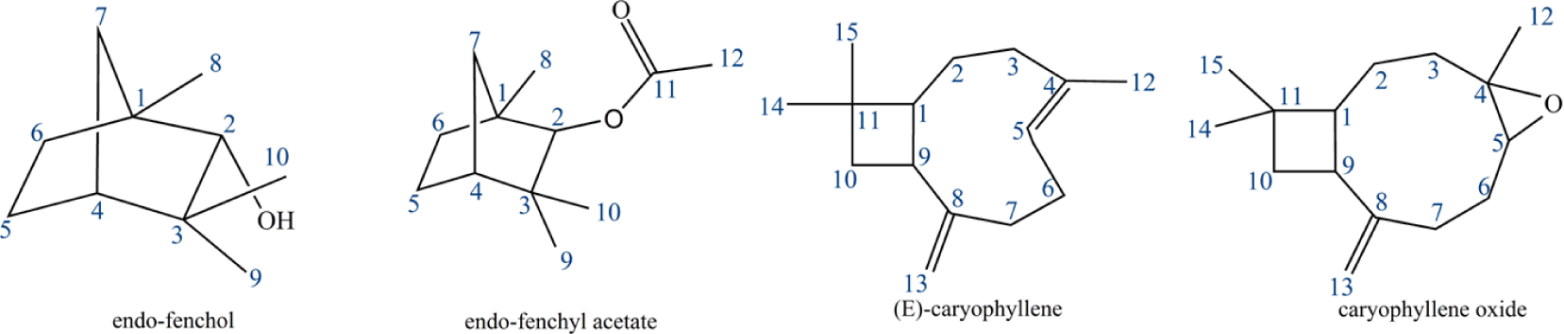
Structures of the main compounds, chemical markers of the species .

**Table 2:** NMR Data for ^1^H (125 MHz; CDCl_3_) of the Sample Presented as Chemical Shifts (δ)

Position	Fenchol	Fenchyl acetate	Caryophyllene	Caryophyllene oxide
1	-	-		
2	3.25 (d)	4.35 (d)		
3	-	-		
4	1.68–1.67 (m)	1.71–1.70 (m)		
5	1.59–1.56 (m); 1.05–1.00 (m)	1.47–1.41 (m); 1.69–1.66 (m)	5.43–529	
6	1.66–1.60 (m); 1.42–1.38 (m)	1.77–1.72 (m); 1.07–1.04 (m)		
7	1.47–1.43 (m); 1.19–1.69 (dd)	1.59–1.57 (q); 1.13–1.11 (dd)		
8	1.08 (s)	1.09 (s)		
9	0.98 (s)	1.03 (s)		
10	0.86 (s)	0.77 (s)		
11	-	-		
12	-	2.07 (s)		
13	-	-	4.94; 4.82	4.97; 4.86
14	-	-		
15	-	-		

Fenchol and fenchyl acetate exhibit characteristic singlets at δ 1.08 and 1.09 for C8, along with methyl signals at δ 0.98–1.03 (C9) and δ 0.86–0.77 (C10), which confirm their bicyclic structures. The presence of a doublet at δ 3.25 in fenchol and at δ 4.35 in fenchyl acetate further differentiates the hydroxyl and acetate functional groups at C2, respectively.

Caryophyllene is distinguished by its olefinic protons at δ 5.43–5.29 (H5), indicative of its bicyclo[3.3.0]­octane system, whereas caryophyllene oxide exhibits shifts at δ 4.97 and 4.86 (H13), confirming the presence of an epoxide moiety. Additionally, the overlapping multiplets in the aliphatic region (δ 1.05–1.77) for all four compounds complicate signal attribution, especially due to spectral congestion from fenchol and fenchyl acetate.

However, the combined use of ^13^C NMR, DEPT-135, and two-dimensional NMR techniques enabled the complete assignment of all proton resonances, ensuring accurate structural characterization (Figures S14–S19).

The NMR data (Table 3) clearly distinguish the four major compounds in the EO*Dr* sample. Fenchol and fenchyl acetate exhibit similar chemical shifts, particularly at δ 49.1 and δ 48.1 (C1) and in the C3–C10 region. The key differentiating feature is the ester carbonyl signal at δ 171.6 in fenchyl acetate, absent in fenchol. Both compounds also display an oxygenated carbon at δ 85.1 and δ 86.1 (C2), indicative of a tertiary alcohol or ester functionality.

**Table 3:** NMR Data for ^13^C (500 MHz; CDCl_3_) of the Sample Presented as Chemical Shifts (δ)

Position	Fenchol	Fenchyl acetate	Caryophyllene	Caryophyllene oxide
1	49.1	48.1	53.5	
2	85.1	86.1	29.3	27.2
3	39.0	39.4	28.3	39.1
4	47.9	48.3	135.5	
5	25.1	25.8	124.3	63.7
6	26.1	26.5	39.9	30.2
7	40.9	41.3	34.8	29.7
8	19.4	29.7	155.0	151.8
9	30.7	19.3	48.4	48.7
10	20.1	20.0	40.3	39.7
11	-	171.6	33.0	34.0
12	-	20.9	16.2	17.0
13	-	-	111.6	112.7
14	-	-	30.0	29.8
15	-	-	22.6	21.6

Caryophyllene is characterized by sp^2^ -hybridized carbons at δ 135.5 (C4) and δ 124.3 (C5), which are not observed in fenchol or fenchyl acetate. Upon oxidation to caryophyllene oxide, notable shifts occur at δ 63.7 (C5) and δ 151.8 (C8), confirming epoxide formation. Additionally, C13 signals at δ 111.6 and δ 112.7 further differentiate these sesquiterpenes.

The assignments of NMR signals were corroborated by comparison with reference spectra from standard samples of fenchol, fenchyl acetate, caryophyllene, and caryophyllene oxide, ensuring the accuracy of the structural characterization (Figures S20–S43). The corresponding chemical shifts are provided in Tables S1 and S2.

The larvicidal activity demonstrated by the essential oil of EO*Dr* can be attributed, in large part, to its major constituents, fenchol and fenchyl acetate, whose efficacy has been previously validated against . These findings corroborate the results of the present study, highlighting the relevance of these monoterpenes as principal bioactive agents.^15^ However, the presence of sesquiterpenes such as caryophyllene and caryophyllene oxide, even in lower concentrations, may contribute to the observed biological effect through synergistic interactions, as previously reported in studies involving essential oils of , where similar constituents exhibited larvicidal activity against both and .^48^ Such synergism between major and minor components is well-documented in essential oil research and underscores the importance of evaluating the complete phytochemical profile rather than isolated compounds alone. These results suggest that the larvicidal potential of EO*Dr* is a multifactorial phenomenon, in which both individual and combinatorial effects play critical roles.

### DLS, PDI, ZP, and TEM Droplet Sizes

The EO*Dr*-PS_80_ mixtures presented a homogeneous appearance with a clear and transparent visual appearance, pH (value ∼ 6), without visible precipitation or phase separation after 24 h and throughout the monitoring period of the experiments, over six months (Figure 4).

**Figure 4:**
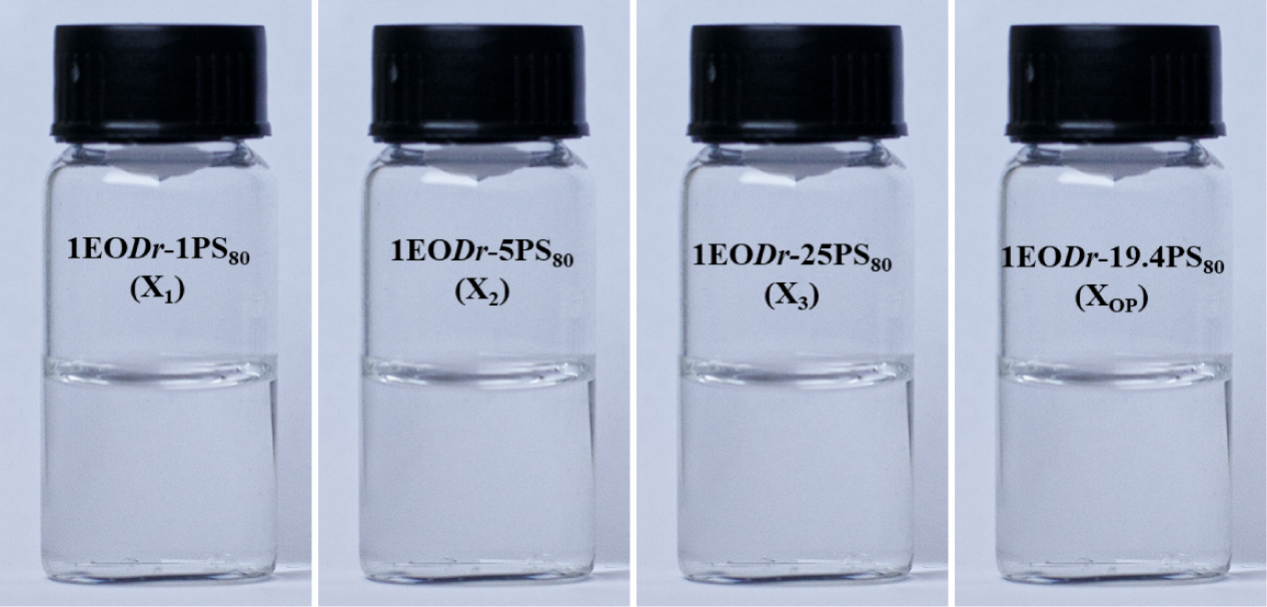
EO*Dr*-PS_80_ mixtures X_1_; X_2_; X_3_; and X_OP_.

The formulation of lipid nanosystems with safe, stable and efficient accumulation in target tissues for in vitro and/or in vivo applications depends on their physicochemical properties, such as size, size distribution and zeta potential.^49^

Table 4 shows the values of the mean droplet size diameter via DLS and TEM techniques, as well as the polydispersity index (PDI) values for the pure components (X_1_, X_2_, X_3_ and X_OP_) of the EO*Dr*-PS_80_ nanoemulsions.

**Table 4:** Droplet Size, PDI, ZP, and TEM of EO*Dr*-PS_80_ Interactions^a^

Components	Diameter (nm)	PDI	Zeta Potential (mV)	TEM (nm)
	MV ± SD	MV ± SD	MV ± SD	MV ± SD
X_1_	933.8 ± 43.1	0.691 ± 0.045	–18.1 ± 0.9	132.0 ± 103.0
X_2_	615.6 ± 39.7	0.660 ± 0.050	–17.9 ± 2.8	86.0 ± 100.0
X_3_	239.8 ± 3.9	0.122 ± 0.025	–36.2 ± 2.0	100.0 ± 72.0
X_OP_	253.4 ± 50.0	0.524 ± 0.031	–24.1 ± 1.1	110.7 ± 60.9

a X_1_: 1EO*Dr*-1PS_80_; X_2_: 1EO*Dr*-5PS_80_; X_3_: 1EO*D*-25PS_80_; X_OP_: 1EO*Dr*-19.4PS_80_; MV ± SD: mean value and standard deviation; replicates: 3.

### Droplet Size, PDI, and ZP

The mean hydrodynamic diameter (Table 4) showed a decreasing trend with an increase in the proportion of nonionic surfactant (PS_80_) in the EO*Dr*-PS_80_ mixtures (X_3_ < X_2_ < X_1_). Although increasing surfactant concentration helps reduce droplet size, excessive amounts may affect biological systems. Being observed for the mixture under optimality conditions (1EO*Dr*-19.4PS_80_) average size (253.4 ± 50.0 nm) with nanoformulated system profile.

In line with the study described in this paper, research by Pascual-Mathey and collaborators^20^ indicated that the optimum conditions for producing rosemary essential oil nanoemulsions with nonionic surfactant were found to be a higher proportion of surfactant than EO in the formulation matrix. The average particle sizes shown in the study were in the 50 nm range, so the authors suggest that the droplets are completely covered by the surfactant and the excess surfactant in the continuous phase.

Regarding the use of the dynamic light scattering (DLS) technique, the heterogeneity or polydispersity index (PDI) is a dimensionless parameter that indicates that indicates the width of the size distribution of molecules, particles, droplets or nanovesicles in the population within a sample.^50^ It provides insights into system homogeneity, in which nanosystems are classified as highly monodisperse (0.0 < PDI ≤ 0.1), moderately polydisperse (0.1 < PDI ≤ 0.4) and highly polydisperse (PDI > 0.4).^51,52^

The PDI values (Table 4) in the EO*Dr*-PS_80_ mixtures follow the same trend as the particle size of the mixtures, in which the pure components with a higher amount of PS_80_ present a lower polydispersity index (X_3_ < X_2_ < X_1_).

The mixtures X_1_ (0.691 ± 0.045), X_2_ (0.660 ± 0.050) and X_OP_ (0.524 ± 0.031) presented a highly polydisperse profile, while X_3_ (0.122 ± 0.025) exhibited a moderately polydisperse profile.

In general, the aggregation or decantation phenomena in emulsified systems tend to be less evident when they exhibit PDI values (0.0 < PDI ≤ 0.1) presenting profiles classified as monodisperse systems.^53,54^ In contrast, the EO*Dr*-PS_80_ mixtures (X_1_, X_2_, X_3_ and X_OP_) did not exhibit visible phase separation or precipitation (Figure 5).

**Figure 5:**
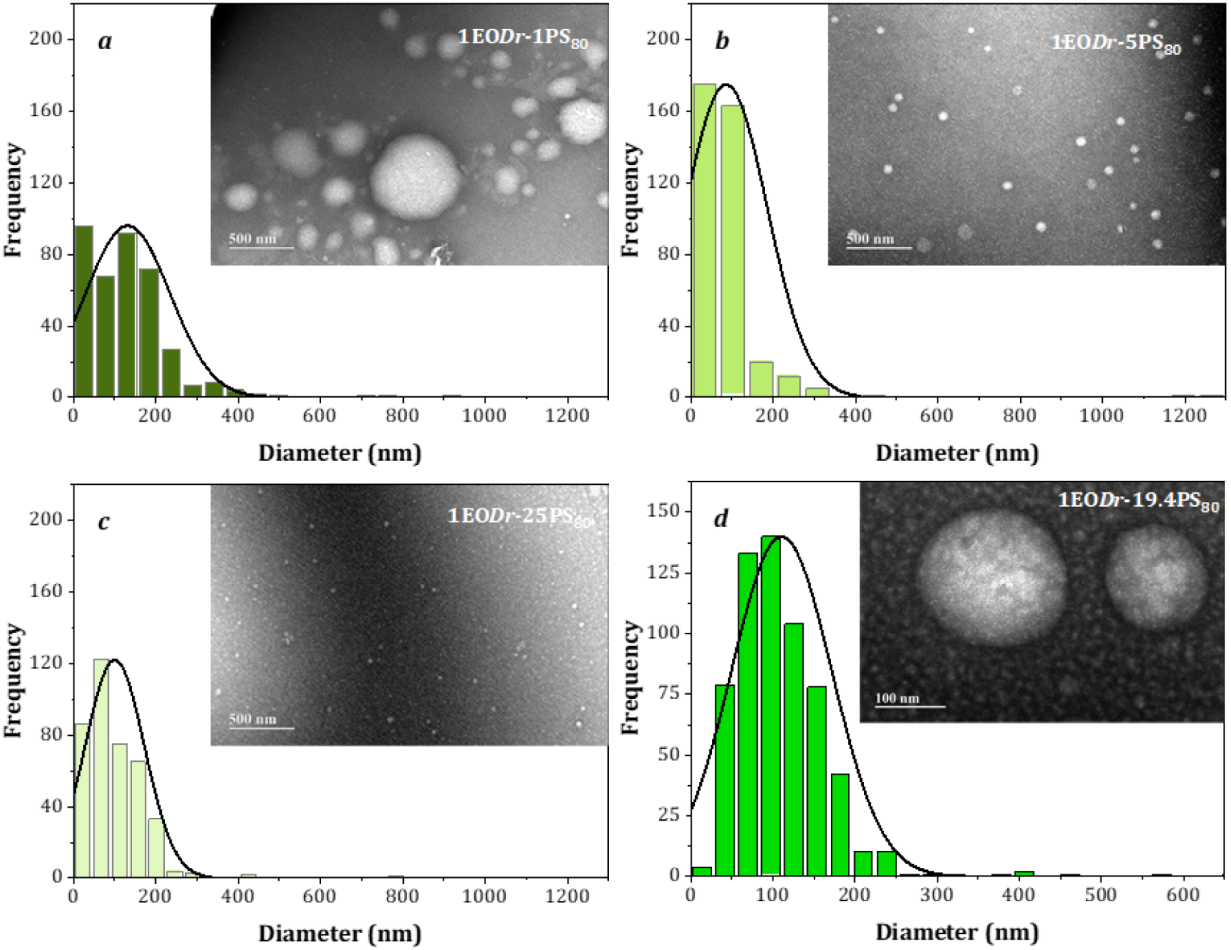
MET micrographs and histograms of the emulsified EO*Dr*-PS_80_ nanoemulsions.

In general, ZP values between ± 0–10 mV indicate high instability due to weak electrostatic repulsion, while values in the range ± 10–20 mV suggest limited stability. Systems with ZP ± 20–30 mV are considered moderately stable and ZP ≥± 30 mV tend to be more electrostatically stable.^52^

For the EO*Dr*-PS_80_ mixtures, the negatively charged ZP values (Table 4) can be attributed to the presence of functional groups of the nonionic surfactant (PS_80_) that are associated with the differences in the degree of dissociation and dipole moments presented by the monoterpene compounds in the matrices of EOs, water and the ethylene oxide groups of the polysorbate.^53–55^

In general, values observed for the pure components, X_1_ and X_2_, are indicative of relative electrical stability (−18.1 and −17.9 mV). For the mixture under optimal conditions X_OP_, moderate stability was observed (−24.1 ± 1.1 mV) and X_3_, high electrical stability (−36.2 mV).

The evaluated EO*Dr*-PS_80_ mixtures show a tendency to increase stability through repulsive electrostatic interactions and steric effect promoted by the long chain of polysorbate 80 responsible for the reduction of the droplet aggregation phenomenon with the increase of PS_80_ in the EO*Dr*-PS_80_ mixtures.^56^

### Morphology and Size by TEM

The size and shape of droplets in emulsified essential oil-surfactant–water systems depend on the molecular geometry and dimensions of the surfactant head and tail groups, because droplets tend to adopt a spherical shape as it minimizes the interfacial free energy.^57^ The TEM electron micrographs of EO*Dr*-PS_80_ (X_1_; X_2_; X_3_ and X_OP_) exhibit the formation of nanodroplets with a spheroidal morphology (Figure 5).

The electron micrographs and the distribution of size values in the histograms (Table 4) indicate nanodroplets with average sizes of 132.0 ± 103.0 nm (Figure 5a), 86.0 ± 100.0 nm (Figure 5b) and 100.0 ± 72.0 nm (Figure 5c) with marked standard deviation values indicated by the polydisperse profile of the mixtures EO*Dr*-PS_80_. Figure 5d shows the profile of EO*Dr*-PS_80_ interactions under optimality conditions (X_OP_) with value (110.7 ± 60.9 nm) showing characteristics of a nanostructured system.

In general, polydispersity phenomena were observed in the TEM micrographs of the mixtures (X_1_, X_2_, X_3_ and X_OP_), displayed by the asymmetric frequency of the normal distribution in the histograms and corroborated by the results of the polydispersity indices (Table 4).

The relative trend of decreasing nanodroplet size increasing was observed with increasing PS_80_ concentration in the pure components, EO*Dr*-PS_80_ (X_3_ < X_2_ < X_1_), up to a critical surfactant concentration. A similar trend was observed in the dynamic light scattering technique of the analyzed samples.

From the analysis of the micrographs and the evaluation of the average size distribution observed in the histograms, there are indications of the action of PS_80_ in the formation, reduction and stabilization of the EO nanodroplets observed in the EO*Dr*-PS_80_ mixtures, since PS_80_ contributes to the stabilization of the EO nanodroplets by reducing the interfacial tension and steric and electrostatic stabilization.^58^

### Larvicidal Evaluation of PS_80_

The use of surfactants is necessary to form a water-miscible system and, consequently, to reduce the volatility of EO emulsified systems. The interaction of EO-H_2_O via a nonionic surfactant depends on its size, micro and/or nanoemulsified to the detriment of macro-emulsified systems. Size affects characteristics such as physical stability, water solubility, protection against degradation agents, controlled release, attenuation of loss of control due to evaporation and increased bioactivity, such as larvicidal activity.^48^

The percentage of larval mortality against the mosquito was analyzed at different concentrations of PS_80_, as shown in Table 5. Tests performed at the listed concentrations of PS_80_ indicate a limitation to the use of PS_80_ in the preparation of emulsified systems in EO*Dr*-PS_80_ mixtures.

**Table 5:** Bioactivity of PS_80_ against Larvae in 24 h^a^

Concentrations (%)	**n,** *Aa*	Dead MV ± SD	%Mortality MV ± SD
		MV ± SD	MV ± SD
0.10	50	0.0 ± 0.0	0.0 ± 0.0
0.50	50	0.0 ± 0.0	0.0 ± 0.0
1.94	50	0.0 ± 0.0	0.0 ± 0.0
2.50	50	0.8± 0.4	8.0 ± 4.5
5.00	50	2.4± 1.5	24.0 ± 15.2
10.00	50	4.4 ± 1.7	44.0 ± 16.7

a MV ± SE: mean value and standard deviation; replicates: 5; n: number of larvae used.

The concentrations equivalent to 0.10, 0.50 and 2.50% PS_80_ showed mortality percentages ranging from 0 to 8%, thus indicating that PS_80_ has no direct influence on the larvicidal action of the EO*Dr*-PS_80_ mixture. Meanwhile, concentrations equivalent to 5 and 10% of PS_80_ showed mortality in the larval populations tested of between 22 and 44%, respectively. In this case, the preparation of EO*Dr*-PS_80_ mixtures with these levels of PS_80_ has a direct influence on the larvicidal activity analyzed.

Concentrations of less than 2.50% PS_80_ are necessary for the formulation of emulsified systems. Highlighting the optimal conditions (1.94% PS_80_) that did not influence larval mortality, being an action inherent to the bioactivity of the EO*Dr* matrix. In general, it is suggested that the concentration of nonionic surfactants and their proportions used in emulsified EOs formulations be better described in the negative control tests of biolarvicidal formulations.

### Larvicidal Evaluation via SCD

The use of SCD instead of conventional pseudoternary phase diagrams provided savings in time and financial resources, reducing the number of experiments and optimizing the development of the formulations.^21–24^ The larvicidal evaluation against field larvae of the mosquito for the lethal concentrations (LC_50_ and LC_90_) of the EO*Dr*-PS_80_ interactions.

These were analyzed at the primary mixture corresponding to the vertex points (1, 2 and 3), in the binary mixtures corresponding to the edge points (4, 5 and 6), the ternary mixture points (7, 8, 9 and 10) corresponding to the central and face points. The mortality percentages and larvicidal activity profile of EO*Dr*-PS_80_ for the determination of LC_50_ and LC_90_ against in SCD are shown in Table S3; Figure S44 and Table 6.

**Table 6:** LC_50_ and LC_90_ Values Obtained by Sigmoidal Adjustment for the Two Replicates and Their Mean and Standard Deviation^a^ ^b^

SCD	LC_50_			LC_90_			Sigmoidal Fit		
Points	R_1_ (mg·L^–1^)	R_2_ (mg·L^–1^)	MV ± SD (mg·L^–1^)	R_1_ (mg·L^–1^)	R_2_ (mg·L^–1^)	MV ± SD (mg·L^–1^)	R^2^_Adj,1_	R^2^_Adj,2_	R^2^_Adj,M_
1	437.9	471.9	482.3 ± 56.4	640.4	548.5	529.5 ± 98.0	0.993	1.000	1.000
2	467.4	453.3	586.6 ± 34.5	715.2	587.5	535.7 ± 96.1	0.997	1.000	1.000
3	361.1	319.8	340.8 ± 44.8	589.8	428.3	528.1 ± 133.7	0.946	0.991	0.980
4	410.0	321.3	365.3 ± 26.9	603.1	596.1	615.5 ± 41.5	0.951	0.994	1.000
5	402.8	276.9	353.6 ± 6.9	772.1	297.7	509.5 ± 18.6	0.991	0.995	1.000
6	356.4	295.4	337.8 ± 18.8	514.4	349.6	456.4 ± 51.0	0.988	1.000	1.000
7	392.6	336.2	353.1 ± 28.8	562.0	532.6	501.1 ± 1.4	0.853	0.999	1.000
8	413.7	353.6	390.2 ± 12.9	694.8	499.8	609.4 ± 40.3	0.998	1.000	1.000
9	350.4	253.4	289.1 ± 36.0	670.7	463.5	507.8 ± 5.2	0.873	0.996	1.000
10	296.9	313.1	311.4 ± 61.3	643.7	392.0	520.4 ± 196.6	0.976	1.000	1.000

a The adjusted coefficient of determination (R^2^_Adj_) was used to assess the adjustment.

b R: replicate; MV: mean value and SD: standard deviation.

Table 6 shows the LC_50_ and LC_90_ values obtained from the EO*Dr*-PS_80_ interaction.

The data obtained indicate that the higher the concentration of PS_80_, the greater the lethality of the emulsified system. We can visualize this logic when we compare the vertex points (1, 2 and 3) in the replicates and the average LC_50_ values, where we see that increasing the concentration of PS_80_ in relation to EO*Dr* reduces the size of the dispersed droplets and consequently increases the larvicidal potential.

It should also be noted that the lowest average LC_50_ observed in the ternary mixtures corresponds to the face point 9 (289.1 mg·L^–1^), while the lowest average LC_90_ value in the binary mixtures refers to the edge point 6 (456.4 mg·L^–1^).

With regard to values CL_50_ and CL_90_, the WHO does not establish concentration parameters to indicate larvicidal efficiency for the mosquito.^32^

On the other hand, they have indicated that compounds and/or bioproducts with values CL_50_ < 50 mg·L^–1^ are highly active; 50 < CL 50 < 100 mg·L^–1^ active; 100 < CL_50_ < 750 mg·L^–1^ effective and CL_50_ > 750 mg·L^–1^ inactive,^5,11,59,60^ the classification being adopted in general for mosquitoes, and . It should also be noted that some of these studies do not present data on the characterization of the formulations used in the selected bioassays, nor the LC_90_ values.

It should be noted that the LC_50_ values must be associated with the LC_90_ values, since the lower and closer the LC_50_ and LC_90_ values, the more information is provided about the maximum larvicidal action analyzed, since low LC_50_ values may not mean concentrations with maximum efficiency (LC_90_) on the larval populations evaluated.^5^ In this respect, this logic can be observed in the ternary mixtures corresponding to edge point 6, which shows average values LC_50_ (337.8 ± 18.8 mg·L^–1^) and LC_90_ (456.4 ± 51 mg·L^–1^).

Therefore, the interaction between EO*Dr* and PS_80_ was determined by analysis of variance (ANOVA) of the replicates (Table S4).

For LC_50_, the coefficients of the components X_1_, X_2_ and X_3_, of very high probabilistic significance (99.9%), X_1_X_2_, significance of 95%, and X_2_X_3_, statistical significance (90%), were defined to represent the EO*Dr*-PS_80_ interaction via LC_50_. Equation 1 describes this interaction:

1 $${\mathrm{LC}}_{50}=461.3\mathrm{EO}\mathrm{Dr}‐{\mathrm{PS}}_{80}+445.3\mathrm{EO}\mathrm{Dr}‐5{\mathrm{PS}}_{80}+339.5\mathrm{EO}\mathrm{Dr}‐25{\mathrm{PS}}_{80}-385.2\mathrm{EO}\mathrm{Dr}‐{\mathrm{PS}}_{80}:\mathrm{EO}\mathrm{Dr}‐5{\mathrm{PS}}_{80}-330.1\mathrm{EO}\mathrm{Dr}‐5{\mathrm{PS}}_{80}:\mathrm{EO}\mathrm{Dr}‐25{\mathrm{PS}}_{80}$$

The contour plot (Figure 6) presents the EO*Dr*-PS_80_ interactions and the larvicidal profile against larvae, based on the LC_50_ and LC_90_.

**Figure 6:**
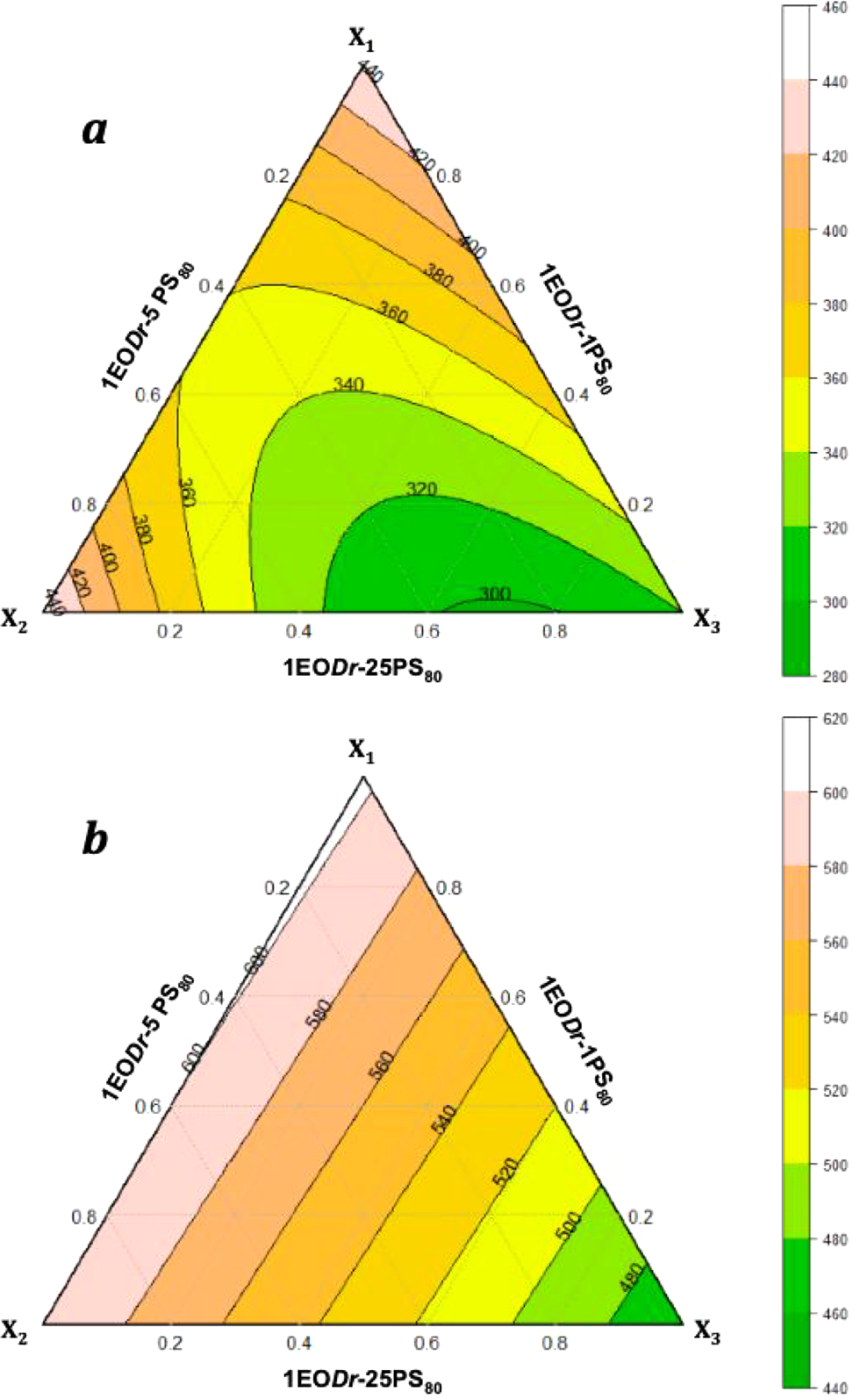
Contour plot of the EO*Dr*-PS_80_ interaction in relation to LC_50_ (a) and LC_90_ (b).

The EO*Dr*-PS_80_ interaction via LC_50_ follows a reduced quadratic polynomial model (R^2^_Adj_ 0.981), illustrates according to the contour surface for this model (Figure 6a), indicating a region of optimality for the EO*Dr*-PS_80_ interaction, around point 6. The optimal value found with the Nlcoptim package for the pure components was 28% of EO*Dr*-5PS_80_ (X_2_) and 72% of EO*Dr*-25PS_80_ (X_3_) was observed with no influence of PS_80_ on larval mortality.

For LC_90_ only the pure components (X_1_, X_2_ and X_3_) were significant, in which case the model followed is linear (R^2^_Adj_ 0.946), shows according to the contour surface for the EO*Dr*-PS_80_ interaction, indicating the vertex points as the best condition (Figure 6b).

This indicates that the higher the concentration of PS_80_ the lower the LC_90_ and, consequently, the greater the larvicidal action. Although the LC_90_ (Figure 6b) signaled the vertex point (X_3_ mixtures) as the best condition, the increase of PS_80_ in the EO*Dr*-PS_80_ interactions presents a limitation of the use of PS_80_ (Table 5) in the mixtures due to its influence on the larvicidal activity. Equation 2 describes this interaction:

2 $${\mathrm{LC}}_{90}=596.7\mathrm{EO}\mathrm{Dr}‐{\mathrm{PS}}_{80}+649.6\mathrm{EO}\mathrm{Dr}‐5{\mathrm{PS}}_{80}+512.7\mathrm{EO}\mathrm{Dr}‐25{\mathrm{PS}}_{80}$$

The data obtained correlates with the EO*Dr* matrix, which has a majority profile of monoterpene and oxygenated monoterpene chemotypes.^15,37^ The interaction of PS_80_ should present variations with the types of chemical constituents present in the different EO matrices.

### Larvicidal Evaluation under Optimal Conditions

The larvicidal profile against mosquito larvae was evaluated based on the LC_50_ under experimentally determined optimal conditions, as observed in the contour diagram (Figure 6a).

Table 7 shows the percentage of mortality observed in interactions under optimal conditions (1EO*Dr*-19.4PS_80_).

**Table 7:** Mortality Profile against under Optimal Conditions in the SCD over a 24 h Period^a^ ^b^ ^c^

EO*Dr*PS_80_ (mg·L^–1^)	n, *Aa*	Dead MV ± SD	%Mortality MV ± SD	LC_50_ MV ± SD	LC_90_ MV ± SD	R^2^_Aj_
		MV ± SD	MV ± SD	MV ± SD	MV ± SD	
100	50	0.8 ± 0.4	8.0 ± 4.5	214.5 ± 11.6	503.8 ± 12.1	0.997
250	50	6.6 ± 1.9	66.0 ± 19.5			
500	50	8.0 ± 1.4	80.0 ± 14.1			
750	50	9.4 ± 0.9	94.0 ± 8.9			
1000	50	10.0± 0.0	100.0 ± 0.0			
BC	50	0.0 ± 0.0	0.0 ± 0.0	-	-	-
NC	50	00.0 ± 0.0	0.0 ± 0.0			
NP	50	10.0 ± 0.0	100.0 ± 0.0			

a MV: mean value; SD: standard deviation; BC: blank control (hatching system mineral water).

b NC: negative control (19.4 × 10^3^ mg·L^–1^); NP: positive control (fersol 1 G/temephos 100 mg·L^–1^).

c Replicates: 5; n: number of larvae used.

The bioassays conducted following the optimality parameters at concentrations of 100 to 1000 mg·L^–1^ showed mortality rates ranging from 8% (100 mg·L^–1^) to 100% (1000 mg·L^–1^), with a percentage above 50% (at 250 mg·L^–1^), exhibiting a sigmoidal profile with increasing mortality following a dose–response relationship (Figure S45).

This suggests a larvicidal effect of the EO*Dr*-PS_80_ formulation attributed to the bioactive compounds in EO*Dr*. It is noteworthy that the concentration of PS_80_ (NC) under optimality conditions did not influence the larvicidal activity of the nanoformulation (0.0 ± 0.0).

The bioassays larvicidal (Figure S45) conducted according to these parameters showed a high correlation coefficient (R^2^_Adj_ 0.997), indicating a strong sigmoidal fit and reliable biological response. The values LC_50_ (214.5 ± 11.6 mg·L^–1^) and LC_90_ (503.8 ± 12.1 mg·L^–1^) indicate effective bioactivity (100 < CL_50_ < 750 mg·L^–1^) against the field larvae of .^11,59,60^

In general, when comparing the values LC_50_ (296.5 mg·L^–1^) and LC_90_ (895.4 mg·L^–1^) from the study carried out by Brandão and collaborators,^15^ of the emulsified system of EO*Dr*-PS_80_ against larvae, the LC_50_ and LC_90_ values obtained under the optimal conditions of the SCD applied in this study show effective target-action activity against larval populations of this species, originating from eggs collected in a real field environment that are susceptible to synthetic chemical larvicides.

In contrast, the use of chemical larvicides such as temephos (an organophosphate larvicide) has been associated with increased resistance in *Aedes* mosquitoes, including and and, consequently, to the increase in arboviruses.^61^ Furthermore, even low-dose use causes toxic effects in aquatic and terrestrial nontarget organisms (insects, plants, animals and humans) due to inhibition of the enzyme acetylcholinesterase with adverse effects on the respiratory, reproductive, nervous, hepatic and renal systems of nontarget organisms.^62,63^

In general, the statistical parameters of SCD presented reliability above 90% for the biological assays, contributing with new insights in nanobiotechnology. The results support the use of SCD as an effective approach to optimize nanoemulsions, indicating the appropriate proportion of PS_80_ in the formulation with effective bioactivity of EO*Dr* against arbovirus-transmitting agents, such as the mosquito.

### Ecotoxicological Evaluation under Optimal Conditions

Ecotoxicological bioassays were performed on microcrustaceans in aquatic environments at EO*Dr*-PS_80_. It should be noted that lethality tests against are rapid, low-cost, and reproducible for the preliminary evaluation of natural and/or synthetic products against a variety of substances. Furthermore, microcrustaceans are considered halophilic organisms that thrive in environments with high salt concentrations, thus playing an important role in aquatic and marine ecosystems as ecological and ecotoxicological bioindicators.^64^

Table 8 presents the results of the bioassays with microcrustaceans of from EO*Dr*-PS_80_, based on the optimality parameters in the SCD.

**Table 8:** Percentage of Mortality against under Optimal Conditions in the SCD in the 48 h Period^a^ ^b^ ^c^

EO*Dr* **-**PS_80_, mg L^–1^	n, *As*	Dead	%Mortality	LC_50_	**LC_50_**	R^2^_Aj_
		MV ± SD	MV ± SD	MV ± SD	MV ± SD	
100	100	1.8 ± 1.2	18.0 ± 11.4	378.8 ± 27.2	716.2 ± 102.7	0.979
250	100	2.6 ± 1.1	26.0 ± 10.8			
500	100	6.7 ± 1.8	67.0 ± 18.3			
750	100	8.4 ± 1.3	84.0 ± 13.5			
1000	100	9.0 ± 1.4	90.0 ± 14.1			
BC	100	0.4 ± 0.9	4.0 ± 9.7	-	-	-
NC	100	0.1 ± 0.3	1.0 ± 3.2			
NP	100	10.0 ± 0.0	100.0 ± 0.0			

a MV: mean value; SD: standard deviation; BC: blank control (hatching system saline solution).

b NC: negative control (PS_80_ 19.4 × 10^3^ mg·L^–1^); NP: positive control (K_2_Cr_2_O_7_ 0.1%); replicates: 10.

c n: Number of used.

The EO*Dr*-PS_80_ nanoformulation under optimal conditions showed mortality rates below 50% at 100 mg·L^–1^ (18.0 ± 11.4) and 250 mg·L^–1^ (26.0 ± 10.8) against microcrustaceans. For concentrations from 500 to 1000 mg·L^–1^, percentages above 50% were observed, displaying an acute toxicity profile (Table 8, Figure S46) of the nanoformulation under optimal SCD conditions against nontarget organisms ( microcrustaceans) with increasing dose tested.

The influence of PS_80_ (NC) on the toxicity of the nanoformulation was negligible, as the mortality rate (1.0 ± 3.2) was below the limit percentage (10%) established as ecotoxicological against microcrustaceans .^35^

The ecotoxicological profile (Figure S46) against of the EO*Dr*-PS_80_ under optimal conditions in the SCD, indicates LC_50_ (378.8 ± 27.2 mg·L^–1^) and LC_90_ (716.2 ± 102.7 mg·L^–1^) values, with a correlation coefficient (R^2^_Aj_ 0.979), showing reliability of the tests carried out, above 90%. The LC_50_ suggests moderate toxicity (100 ≤ LC_50_ ≤ 500 mg·L^–1^) against microcrustaceans.^64–68^

Considering that the microcrustacean plays a significant role in the dynamics of aquatic ecosystems, the ecotoxicological profile of EO*Dr*-PS_80_ nanoformulations indicates that the terpenoid constituents have potential for use in habitats where arbovirus vector larvae reproduce.^68,69^

The results obtained offer promising perspectives for the application of EO*Dr*-PS_80_ nanoformulations, and further tests with nontarget organisms are needed to more fully evaluate the toxicity profile of the developed formulations.^70^ The EO*Dr*-PS_80_ presented a toxicity profile against nontarget organisms with lower toxicity than the chemical larvicides currently used.

The formulation strategy adopted for the EO*Dr*-PS_80_ nanoemulsion was deliberately guided by the principles of green chemistry and environmental sustainability. The system was developed using a low-energy oil-in-water emulsification method, relying exclusively on water and polysorbate 80-both nontoxic, biodegradable, and widely accepted as environmentally benign solvents. No hazardous organic solvents or energy-intensive procedures were employed at any stage. Moreover, the implementation of a Simplex Centroid Design (SCD), in lieu of conventional pseudoternary phase diagrams, enabled a substantial reduction in experimental workload, raw material use, and waste generation. Collectively, these features underscore the eco-friendly and resource-efficient character of the formulation, highlighting its potential as a sustainable alternative for larvicidal applications.

## Supplementary Material



## Notes

Additional experimental details, including figures and tables (PDF)
