# Supplementary material for: Influence of Polysorbate 80 on the Larvicidal and Ecotoxicological Profile of Essential Oil Nanoemulsion: Insights into Green Nanotechnology
Source: J Agric Food Chem. 2025 Jul 24;73(31):19327–39. doi: 10.1021/acs.jafc.5c04690 (PMC12333368; doi:10.1021/acs.jafc.5c04690)
Supplement: Supplementary file 1 [file jf5c04690_si_001.pdf]

**SUPPORTING INFORMATION**

# Influence of Polysorbate 80 on the Larvicidal and Ecotoxicological Profile of Dizygostemon riparius Essential Oil Nanoemulsion: Insights into Green Nanotechnology

Clenilma M. Brandão[1,3]; Djanira R. dos Santos[1,3]; Lucas G. P. Silva[2,3]; Mirla C. Ferreira[2,3]; Joyce M. de F. Mesquita[3]; Melissa P. Souza[4]; Carlos A. Holanda[5]; Renato S. Gonçalves[6]; Emmanoel V. Costa[7]; Georgiana E. de C. Marques[3]; Rogério de M. Teles[3]; Kiany S. B. Cavalcante[1,2,3*]

[1] Postgraduate Program in Chemistry, Associative Doctorate UFMA-IFMA, Federal Institute of Maranhão, São Luís-Monte Castelo campus, São Luís, MA, 65030-005, Brazil;
[2] Postgraduate Program in Chemistry, Federal Institute of Maranhão, São Luís-Monte Castelo campus, São Luís, MA, 65030-005, Brazil;
[3] Laboratory of Microbiological Analysis. Department of Chemistry, Federal Institute of Maranhão, São Luís-Monte Castelo campus, São Luís, MA, 65030-005, Brazil;
[4] Postgraduate Program in Chemistry, Federal University of Amazonas, Manaus, AM, 69080-900, Brazil;
[5] Natural Sciences Degree Coordination, Federal University of Maranhão, Bom Jesus campus, Imperatriz, MA, 65915-060, Brazil;
[6] Department of Chemistry, Federal University of Maranhão. Postgraduate Program in Chemistry, São Luis campus, São Luís, MA, 65080-805, Brazil;
[7] Department of Chemistry, Federal University of Amazonas. Postgraduate Program in Chemistry, Federal University of Amazonas, Manaus, AM, 69080-900, Brazil.

[*]Email: kiany@ifma.edu.br. Phone: (+55) (98) 98116 6809.

## List of Supplementary Figures


**Figure S 1**.Chromatogram of the total ions of EODr, lilac floral morphotype........... 11
**Figure S 2.**Mass spectrum the fragmentation pattern of the major compound, fenchol <endo> (RT 18.23 min). .......................................................................... 12
**Figure S 3.**Mass spectrum the fragmentation pattern of the major compound, fenchyl acetate <endo> (RT 21.86 min)........................................................................ 13
**Figure S 4.**Mass spectrum the fragmentation pattern of the major compound, E-caryophyllene (RT 28.61 min). ........................................................................ 14
**Figure S 5.**Mass spectrum the fragmentation pattern of the major compound, caryophyllene oxide (RT 33.56 min)................................................................ 15
**Figure S 6.**Chromatogram of fenchol <endo> of the pure compound, chemical standard (Sigma-Aldrich).......................................................................................... 16
**Figure S 7.**Mass spectrum the fragmentation pattern of the fenchol <endo> from Sigma-Aldrich (TR 18.42 min). ......................................................................... 17
**Figure S 8.**Chromatogram of total ions of fenchyl acetate <endo> of the pure compound, chemical standard (Sigma-Aldrich).................................................... 18
**Figure S 9**.Mass spectrum the fragmentation pattern of the fenchyl acetate <endo> from Sigma-Aldrich (TR 21.97 min). ......................................................................... 19
**Figure S 10**.Chromatogram of the total ions of E-caryophyllene of the pure compound, chemical standard (Sigma-Aldrich). ................................................................... 20
**Figure S 11**.Mass spectrum the fragmentation pattern of the E-caryophyllene from Sigma-Aldrich (TR 28.87 min). ......................................................................... 21
**Figure S 12.**Chromatogram of the total ions of caryophyllene oxide of the pure compound, chemical standard (Sigma-Aldrich).................................................... 22
**Figure S 13**.Mass spectrum the fragmentation pattern of the caryophyllene oxide from Sigma-Aldrich (TR 33.77 min). ......................................................................... 23
**Figure S 14**.$^{1}$H NMR (500 MHz) spectrum of EODr in $CDCl_3$ .............................. 24
**Figure S 15.**$^{13}$C NMR (125 MHz) spectrum of EODr in $CDCl_3$ ............................ 28
**Figure S 16.**$^{13}$C NMR and DEPT 135 (125 MHz) spectrum of EODr in $CDCl_3$ ........ 30
**Figure S 17.**$^{1}$H-$^{1}$H-COSY (500 MHz) spectrum of EODr in $CDCl_3$........................ 32
**Figure S 18.**-$^{13}$C$^{1}$H -HSQC ($^{1}$H 500 MHz; $^{13}$C 125 MHz) spectrum of EODr in $CDCl_3$ ...................................................................................................... 33
**Figure S 19**.$^{1}$H-$^{13}$C-HMBC ($^{1}$H 500 MHz; $^{13}$C 125 MHz) spectrum of EODr in $CDCl_3$ ...................................................................................................... 37
**Figure S 20.**1H NMR (500 MHz) spectrum of fenchol in $CDCl_3$ ........................... 44
**Figure S 21**.$^{13}$C NMR (125 MHz) spectrum of fenchol in $CDCl_3$........................... 47
**Figure S 22**.$^{13}$C NMR and DEPT 135 (125 MHz) spectrum of fenchol in $CDCl_3$ ...... 48
**Figure S 23**.$^{1}$H-$^{1}$H-COSY (500 MHz) spectrum of fenchol in $CDCl_3$ ...................... 49
**Figure S 24**.$^{1}$H-$^{13}$C-HSQC ($^{1}$H 500 MHz; $^{13}$C 125 MHz) spectrum of fenchol in $CDCl_3$ ...................................................................................................... 50
**Figure S 25**.$^{1}$H-$^{13}$C-HMBC ($^{1}$H 500 MHz; $^{13}$C 125 MHz) spectrum of fenchol in $CDCl_3$ ............................................................................................ 52
**Figure S 26**. $^{1}$H NMR (500 MHz) spectrum of fenchyl acetate in $CDCl_3$................. 55
**Figure S 27**. $^{13}$C NMR (125 MHz) spectrum of fenchyl acetate in $CDCl_3$................ 59
**Figure S 28.** $^{13}$C NMR and DEPT 135 (125 MHz) spectrum of fenchyl acetate in $CDCl_3$ ...................................................................................................... 60
**Figure S 29**. $^{1}$H-$^{1}$H-COSY (500 MHz) spectrum of fenchyl acetate in $CDCl_3$ .......... 61

**Figure S 30**. $^1H$-$^{13}C$-HSQC ($^1H$ 500 MHz; $^{13}C$ 125 MHz) spectrum of fenchyl acetate in $CDCl_3$........ 62
**Figure S 31**. $^1H$-$^{13}C$-HMBC ($^1H$ 500 MHz; $^{13}C$ 125 MHz) spectrum of fenchyl acetate in $CDCl_3$........ 64
**Figure S 32.** $^1H$ NMR (500 MHz) spectrum of E-caryophyllene in $CDCl_3$........ 68
**Figure S 33**. $^{13}C$ NMR (125 MHz) spectrum of E-caryophyllene in $CDCl_3$......... 71
**Figure S 34.** $^{13}C$ NMR and DEPT 135 (125 MHz) spectrum of E-caryophyllene in $CDCl_3$........ 73
**Figure S 35**. $^1H$-$^1H$-COSY (500 MHz) spectrum of E-caryophyllene in $CDCl_3$........ 75
**Figure S 36**. $^1H$-$^{13}C$-HSQC ($^1H$ 500 MHz; $^{13}C$ 125 MHz) spectrum of E-caryophyllene in $CDCl_3$........ 76
**Figure S 37**. $^1H$-$^{13}C$-HMBC ($^1H$ 500 MHz; $^{13}C$ 125 MHz) spectrum of E-caryophyllene in $CDCl_3$........ 79
**Figure S 38.** $^1H$ NMR (500 MHz) spectrum of caryophyllene oxide in $CDCl_3$......... 84
**Figure S 39**. $^{13}C$ NMR (125 MHz) spectrum of caryophyllene oxide in $CDCl_3$......... 87
**Figure S 40.** $^{13}C$ NMR and DEPT 135 (125 MHz) spectrum of caryophyllene oxide in $CDCl_3$......... 88
**Figure S 41.** $^1H$-$^1H$-COSY (500 MHz) spectrum of caryophyllene oxide in $CDCl_3$.... 89
**Figure S 42.** $^1H$-$^{13}C$-HSQC ($^1H$ 500 MHz; $^{13}C$ 125 MHz) spectrum of caryophyllene oxide in $CDCl_3$......... 90
**Figure S 43.** $^1H$-$^{13}C$-HMBC ($^1H$ 500 MHz; $^{13}C$ 125 MHz) spectrum of caryophyllene oxide in $CDCl_3$........ 93
**Figure S 44.** Profile of the larvicidal activity of EO*Dr*-$PS_{80}$ interactions against *A. albopictus* in SCD........ 98
**Figure S 45.** Larvicidal profile against *A. albopictus* of EO*Dr*-$PS_{80}$ under optimal conditions ($LC_{50}$ 214.5±11.6 mg.$L^{-1}$; $LC_{90}$ 503.8±12.1 mg.$L^{-1}$; $R^2_{Aj}$ 0.997)......... 99
**Figure S 46.** Ecotoxicological profile against *A. salina* under optimal SCD conditions. $LC_{50}$ 372.8±27.2 mg.$L^{-1}$; $LC_{90}$ 716.2±102.7 mg.$L^{-1}$; $R^2_{Aj}$ 0.979......... 100

## List of Supplementary Tables

**Table S 1**. NMR data for $^1H$ (125 MHz; $CDCl_3$) of the standard samples fenchol, fenchyl acetate, caryophyllene, and caryophyllene oxide presented as chemical shifts (δ)........ 101
**Table S 2.** NMR data for $^{13}C$ (500 MHz; $CDCl_3$) of the standard samples fenchol, fenchyl acetate, caryophyllene, and caryophyllene oxide presented as chemical shifts (δ)........ 102
**Table S 3**. The mortality percentages of EO*Dr*-$PS_{80}$ for the determination of the larvicidal profiles and $LC_{50}$ and $LC_{90}$ against *A. albopictus* in SCD......... 103
**Table S 4.** Parameters for determining the predictive equation for the EO*Dr*-$PS_{80}$ interaction. The special cubic model is used as a reference for the SCD......... 104

# EXPERIMENTAL SECTION

## Materials

### Materials Chemical Tests

Milli-Q® ultrapure water; commercially purchased mineral water; anhydrous sodium sulfate ($Na_2SO_4$ P.A. purity 99% Synth); polysorbate 80 ($C_{64}H_{124}O_{26}$ P.A. Neon); potassium dichromate ($K_2Cr_2O_7$ P.A. Impex); fenchol ($C_{10}H_{18}O$ purity 96% Sigma-Aldrich); fenchyl acetate ($C_{12}H_{20}O_2$ purity 96% Sigma-Aldrich); caryophyllene ($C_{15}H_{24}$ purity 98% Sigma-Aldrich); caryophyllene oxide ($C_{15}H_{24}O$ purity 95% Sigma-Aldrich) and alkanes calibration standard ($C_8$-$C_{40}$) for performance tests of GC-systems supelco commercially purchased from Sigma-Aldrich Brasil Ltda.

### Materials Biological Tests

*Aedes albopictus* eggs were collected in ovitraps in a field environment; the commercial larvicides Fersol 1G and VectoBac were provided by health surveillance agents (São Luís-MA, Brazil); *Artemia salina* cysts were commercially purchased (Maramar brand, 5g package).

### Collection of Plant Material and Extraction

The plant material of the species *D. riparius*, lilac morphotype, was collected in the municipality of São Benedito do Rio Preto, Maranhão (geographic coordinates 03°19'27.9"S; 43°31'02.6"W) and deposited as a voucher specimen in the Rosa Mochel Herbarium (SLUI) with registration nº 8656 and SISGEN record nº A5E5CD0.

The leaf biomass (100g) was dried at room temperature in the shade for 5 days, after which it was ground in a stainless-steel cyclone rotor mill (model TE-651/2; TECNAL; 60 mesh). The EO*Dr* was extracted by hydrodistillation using a glass Clevenger extractor with a water recycling system (Ultrathermostatic bath; SSDu - 10 L). The residual moisture in EO*Dr* was removed by adding anhydrous sodium sulphate ($Na_2SO_4$ P.A., purity 99%, Synth) and then the oil was subjected to a centrifugation process and finally packed in a glass vial and stored under refrigeration (2-7 ºC) in a refrigerator[1-3].

**Chemical composition: GC-FID-MS**

GC-FID analysis was conducted using a Shimadzu GC-17A gas chromatograph equipped with a DB-5MS capillary column (30 m × 0.25 mm × 0.25 µm). Helium was used as the carrier gas, with a flow rate of 1 mL.min$^{-1}$. The injection solution was prepared by dissolving 10 mg of oil in 1 mL of dichloromethane ($CH_2Cl_2$), a solvent selected for its high volatility and efficiency in dissolving essential oils, and 1 µL of this solution was injected with a split ratio of 1:50. The column temperature program was as follows: an initial temperature of 40 °C maintained for 4 minutes, followed by a temperature ramp of 4 °C.min$^{-1}$ to 240 °C, then 10 °C.min$^{-1}$ to 280 °C, which was held for 2 minutes. The injector and detector temperatures were set to 250 °C and 220 °C, respectively.

GC-MS analysis was performed using a Trace Ultra gas chromatograph coupled with an ISQ single quadrupole mass spectrometer (Thermo Scientific). The system was equipped with a Tri Plus autosampler and a DB-5MS capillary column (30 m × 0.25 mm × 0.25 µm). The injector, interface, and ion source temperatures were maintained at 250 °C, 250 °C, and 220 °C, respectively. Mass spectra were acquired over a range of *m/z* 40–440. All other conditions were identical to those used for the GC-FID analysis. The RI values were determined using a homologous ($C_8$–$C_{40}$), which provided sufficient reference points for most identified compounds. Calculations were performed according to the Van den Dool and Kratz equation[4]. The identification of oil chemical constituents was carried out by comparing the obtained mass spectra with those available in the NIST library and by comparing retention indices (RI) with published data[5].

**Chemical composition: NMR $^{1}$H and $^{13}$C**

The nuclear magnetic resonance (NMR) spectra of the EO*Dr* sample and chemical patterns of major compounds, including one-dimensional ($^{1}$H, $^{13}$C and DEPT-135) and two-dimensional (COSY, HSQC, and HMBC), were recorded using a BRUKER AVANCE III HD spectrometer (Billerica, MA, USA), operating at 11.75 Tesla (500.13 MHz for $^{1}$H NMR and 125.76 MHz for $^{13}$C NMR). The sample was dissolved in deuterated chloroform ($CDCl_3$), and chemical shifts were reported in parts per million (ppm), referenced to tetramethylsilane (TMS, δ 0.00 ppm) as the internal standard.

**Nanoemulsions Formulation**

The nanoemulsions were obtained by the low-energy oil-water emulsification method without the use of specialized equipment[6].

The steps of preparation of the mixtures involved some of the 12 principles of green chemistry[7], of which we highlight: use and generation of less toxic products (principles 3 and 4); use of eco-efficient solvents (principle 5); the steps of preparation and application of the emulsified mixtures were conducted with energy efficiency (principle 6).

The use of SCD instead of conventional pseudoternary phase diagrams provided savings in time and financial resources, reducing the number of experiments and optimizing the development of the formulations[8-11].

To determine the role of $PS_{80}$ in the EO*Dr*-$PS_{80}$ interaction, the concentration of EO*Dr* in the mixtures was kept constant. The proportions for emulsification were: 1EO*Dr*-1$PS_{80}$ ($X_1$); 1EO*Dr*-5$PS_{80}$ ($X_2$) and 1EO*Dr*-25$PS_{80}$ ($X_3$) g.$L^{-1}$.

The distribution of the proportions and their mixtures in the SCD, the proportions $X_1$ , $X_2$ and $X_3$ are equivalent to the vertex points (1, 2 and 3), the binary mixtures $1/2X_1$-$1/2X_2$, $1/2X_1$-$1/2X_3$ and $1/2X_2$-$1/2X_3$ refer to the edge points (4, 5 and 6), the ternary mixtures $2/3X_1$-$1/6X_2$-$1/6X_3$,$1/6X_1$-$2/3X_2$-$1/6X_3$ and $1/6X_1$-$1/6X_2$-$2/3X_3$ correspond to the face points (8, 9 and 10) and the ternary mixture $1/3X_1$-$1/3X_2$-$1/3X_3$ corresponds to the central point (7).

However, the direct use of mortality percentage values in the SCD was unable to determine the influence of $PS_{80}$ via the larvicidal activity of the emulsified system[12]. In this case, five concentrations (100 to 1000 mg.$L^{-1}$) were prepared for the proportions and their mixtures.

This procedure made it possible to calculate the lethal concentrations with fifty ($LC_{50}$) and ninety ($LC_{90}$) percent larvicidal activity, and consequently determined the best amount of $PS_{80}$ for the EO*Dr*-$PS_{80}$ interaction.

Linear, quadratic, and cubic models were used to explain the interactions in the mixture designs, however, the ternary interaction in the SCD was explained by the special cubic model, which is represented by equation S1. The first term indicates the non-interaction between the components $X_1$, $X_2$ and $X_3$ (linear), the second term represents the binary interactions (quadratic) and the third shows the ternary interactions (cubic).

$$Y = \sum_{i=1}^{q} \beta_i \mathcal{X}_i + \sum \sum_{i<j}^{q} \beta_{ij} \mathcal{X}_i \mathcal{X}_j + \sum \sum_{i<j<\kappa} \sum^{q} \beta_{ij\kappa} \mathcal{X}_i \mathcal{X}_j \mathcal{X}_\kappa$$

**Eq S1**

## Hydrodynamic Diameter, PDI and ZP

The EO*Dr*-$PS_{80}$ interaction was evaluated by size distribution, polydispersity index (PDI) and zeta potential (ZP).

The average diameter and PDI were evaluated using the dynamic light scattering technique. The ZP was measured using the electrophoretic light scattering technique, both in a Zetasizer Nano ZS90 (Malvern), with a laser beam at 633 nm; 25°C and 1.467 refractive index. An aliquot of 1 mL of the EO*Dr*-$PS_{80}$ mixtures was used, and the readings were taken in triplicate.

The analyses were carried out in polystyrene disposable sizing cuvette and polystyrene disposable sizing cuvette-zeta dip cell, respectively, for the size distribution and zeta potential analyses.

## Morphology and Size by TEM

To determine the average diameter and study the morphology of the EO*Dr*-$PS_{80}$ emulsified systems we used a Transmission Electron Microscope (TEM), JEM-2100 (JEOL, Tokyo, Japan) equipped with EDS, Thermo scientific; Lanthanum hexaboride filament electron beam ($LaB_6$); Acceleration voltage of 200kV, with a resolution of 2.5 angstrom resolution; ORIUS™ SC 1000 CCD camera, Gatan brand; Digital Micrograph software; Energy dispersive spectroscopy (EDS) with Thermo Scientific NSS Spectral Imaging detector for elemental identification.

The EO*Dr*-$PS_{80}$ emulsified systems were diluted 50% (v/v) in ultrapure water (purified in a Milli-Q system®). The negative stain technique was used for the samples of the EO*Dr*-$PS_{80}$ emulsified systems; 430 mesh carbon-coated copper grid. For EO*Dr*-$PS_{80}$, a contrast agent (2% uranyl acetate, $UO_2(CH_3COO)_2$).

Nanodroplet counting was performed on original images using the open access software ImageJ (https://imagej.net/ij/). Nanodroplet size distribution histograms were constructed from counting 380 for the pure EO*Dr*-$PS_{80}$ nanoemulsions ($X_1$; $X_2$; $X_3$) and 610 nanodroplets for the EO*Dr*-$PS_{80}$ nanoemulsions under optimality conditions ($X_{OP}$) and the results were evaluated by calculating the mean and standard deviation values using the Origin Pro software (version 8.5) with a 95% confidence interval ($p < 0.05$).

**Larvicidal bioassays**

The larvicidal bioassays of the EO*Dr*-$PS_{80}$ emulsified systems against *A. albopictus* mosquito larvae followed the methodology recommended by the WHO with adaptations[1, 13].

The eggs were collected in the field (F0 generation) with oviposition traps (ovitraps) distributed in the region of Estrada da Maioba, a rural area in the municipality of Paço do Lumiar, Maranhão, Brazil.

Larvae in the third larval stage were immersed in a volume of 20 mL of the emulsified EO*Dr*-$PS_{80}$ formulations packaged in polystyrene cups with a final volumetric capacity of 50 mL. The 10 SCD experiments were performed in duplicate for concentrations of 100; 250; 500; 750 and 1000 $mg.L^{-1}$, with each experiment using 10 *A. albopictus* larvae from a rural environment. Totaling a sample universe of 1.000 larvae.

For the white control, mineral water used in the hatching system of the *A. albopictus* mosquito eggs present in the eucatex of the ovitraps was used. Five experiments were carried out in quintuplicate, totaling a sample universe of 250 *A. albopictus* larvae.

For the negative control, five concentrations of aqueous solution of $PS_{80}$ ($C_{64}H_{124}O_{26}$ P.A., Neon) were prepared, 0.10; 0.50; 1.94; 2.50; 5.00 and 10.00% mass/volume carried out in quintuplicate with 10 larvae in each replicate, totaling a sample universe of 300 larvae.

For the positive control, the commercial larvicide Fersol 1G, provided by health surveillance agents (São Luís, Maranhão, Brazil), was evaluated. The concentration (100 $mg.L^{-1}$) indicated in the commercial application technical sheet (nº29/2024-CGARB/DEDT/SVSA/MS) was adopted, in accordance with WHO guidelines[14]. The test was performed in quintuplicate with 10 larvae in each repetition, totaling a sample of 50 larvae.

For optimal conditions, the same concentrations of SCD (100; 250; 500; 750 and 1000 $mg.L^{-1}$) were applied, in addition to the blank (mineral water), negative ($PS_{80}$ 1.94%) and positive (FERSOL 1G 100 $mg.L^{-1}$) controls, being carried out in quintuplicate with 10 *A. albopictus* larvae in each replicate. Totaling a sample universe of 400 larvae.

The bioassays were monitored over a 24-hour period and the percentage of dead and live larvae was subsequently counted. Larval identification was carried out based on the larval identification keys[15], after applying the test to all the larvae (dead and alive), using a trinocular optical microscope (Alltion) with 4X and 10X magnification objectives.

**Ecotoxicity Tests**

Aquatic ecotoxicology tests followed the protocols for toxicity tests standardized by the Brazilian Association of Technical Standards (ABNT), NBR 16530:2016 [16] against *A. salina* microcrustaceans, meeting the requirements for the competence of chemical testing laboratories, based on ABNT, NBR ISO IEC 17025:2017[17]. High-hatching cysts purchased commercially (Maramar brand, 5g package) were used.

To perform the bioassays, five dilutions were prepared, starting from the optimal point, and 10 mL aliquots of the formulation were transferred in five concentrations (100; 250; 500; 750 and 1000 $mg.L^{-1}$), containing 10 microcrustaceans in the metanauplius phase.

For the blank control, saline solution from the hatching system was used in simulation of the natural environment of *A. salina* microcrustaceans, according to the parameters of NBR 16530:2016 [35]. For the negative control, a 1.94% $PS_{80}$ solution ($C_{64}H_{124}O_{26}$ P.A., Neon) was used. For the positive control, a 0.1% potassium dichromate solution ($K_2Cr_2O_7$ P.A., Impex) was used.

Ten replicates were performed for each concentration tested, as well as for blank, positive and negative controls. In total, 80 experiments and a sample universe of 800 *A. salina* microcrustaceans in the metanauplius phase were evaluated.

The tests were monitored for 24 and 48 hours, and the results were expressed as a percentage of mortality of *A. salina* microcrustaceans.

**Statistical analysis**

The larvicidal efficacy of EO*Dr*-$PS_{80}$ against *A. albopictus* larvae was determined by obtaining contour plots. The larvicidal data and statistics were analyzed using Origin software (version 8.5) and R software (version 4.2.3). The $LC_{50}$ and $LC_{90}$ were determined using the Origin pro 8.5 computer program and the adjusted coefficient of determination ($R^2_{Adj}$) was used to determine the adjustment of the larvicide tests.

The $LC_{50}$ and $LC_{90}$ values were obtained from the replicates in the SCD, the replicates being necessary for the analysis of variance (ANOVA). The mixexp package was used to generate the SCD, analyze the ANOVA, obtain the equations representing the EO*Dr*-$PS_{80}$ interaction (linear, quadratic or special cubic) and construct the contour surface plots while the Nlcoptim package was used to optimize the data. The fit of the model was determined by the coefficients of determination ($R^2$ and $R^2_{Adj}$) and by the

probabilistic significance at 0.001 (***), 0.01 (**), 0.05 (*) and 0.1 (#) of the statistical parameters[18, 19].

The results of the ecotoxicological tests against *A. salina* microcrustaceans were evaluated by calculating the mean and standard deviation values. The lethal concentrations 50 and 90 ($LC_{50}$ and $LC_{90}$) in the toxicity tests, as well as the sigmoidal profile and the adjusted coefficient of determination ($R^2_{Adj}$) were determined using the Origin Pro software (version 8.5) with a 95% confidence interval ($p < 0.05$).

## RESULTS AND DISCUSSION

### Chemical profile: GC-MS and CG-FID

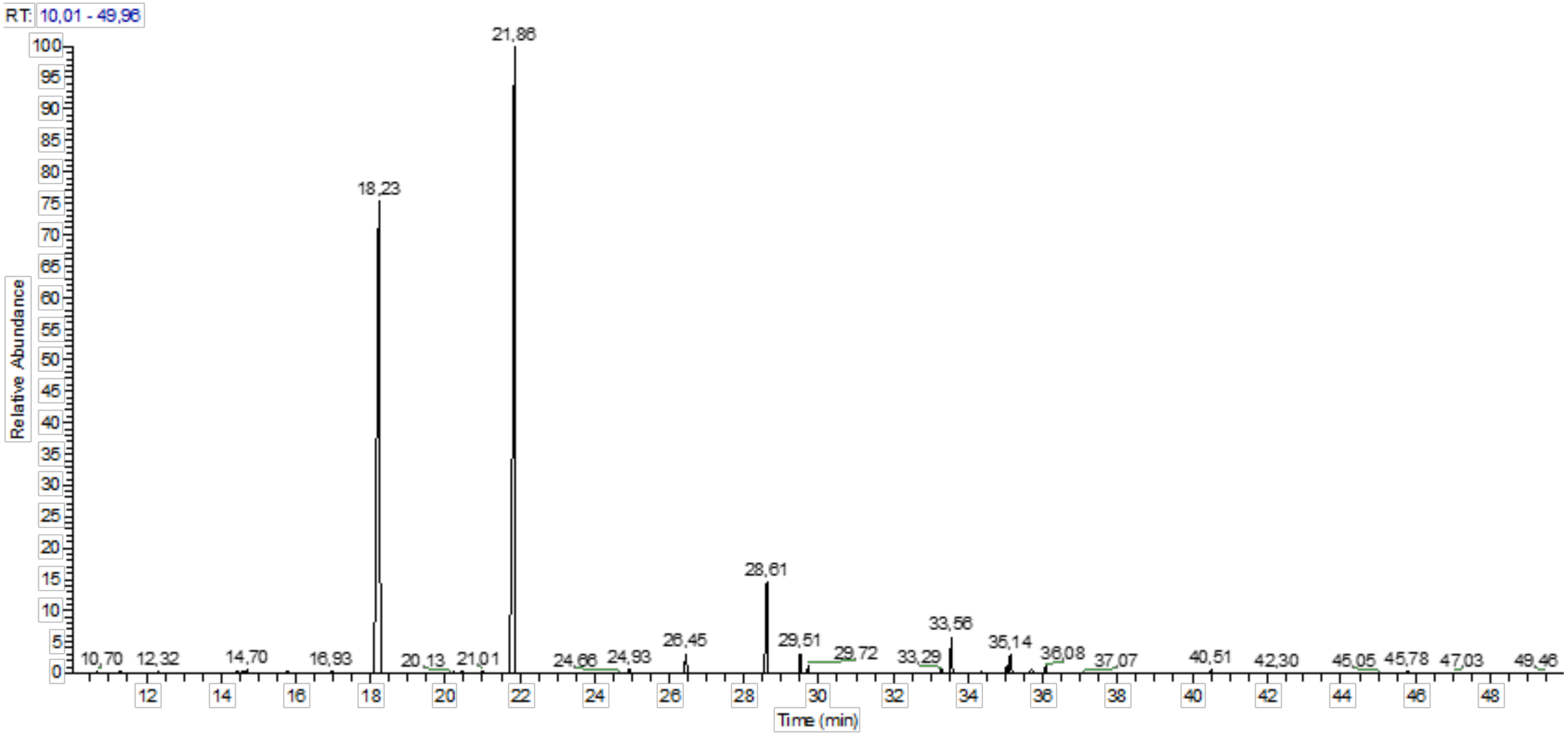


**Figure S 1**.Chromatogram of the total ions of EODr, lilac floral morphotype.

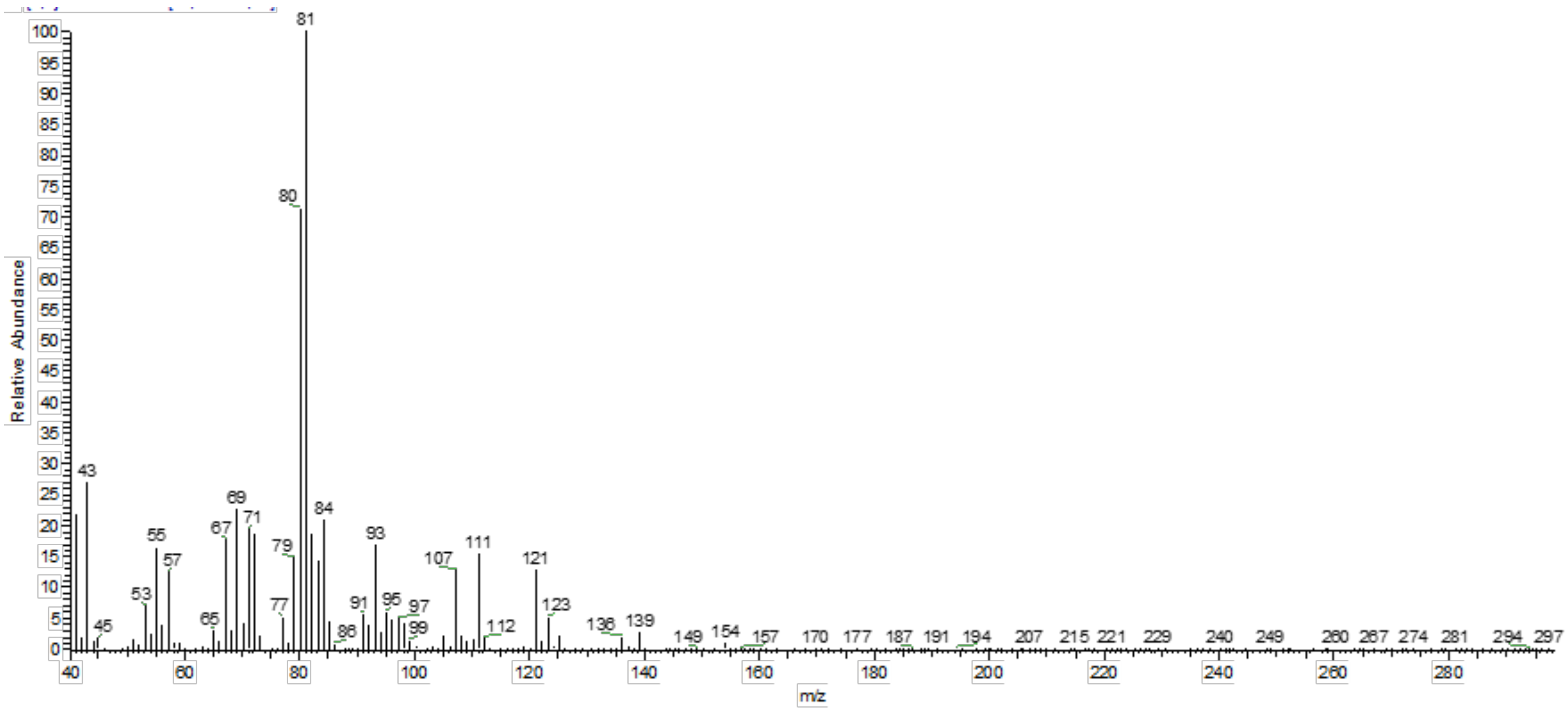


**Figure S 2.**Mass spectrum the fragmentation pattern of the major compound, fenchol <endo> (RT 18.23 min).

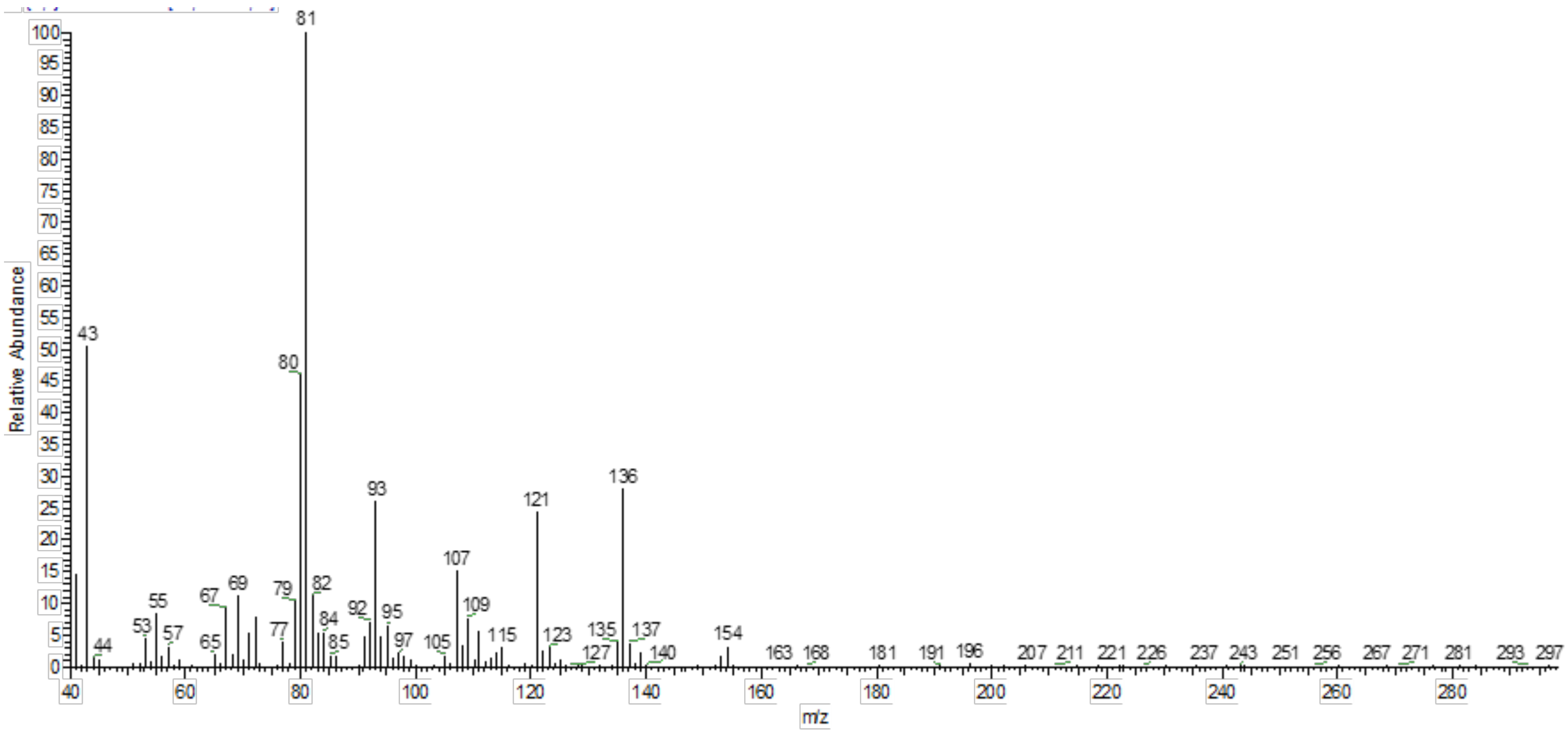


**Figure S 3.**Mass spectrum the fragmentation pattern of the major compound, fenchyl acetate <endo> (RT 21.86 min).

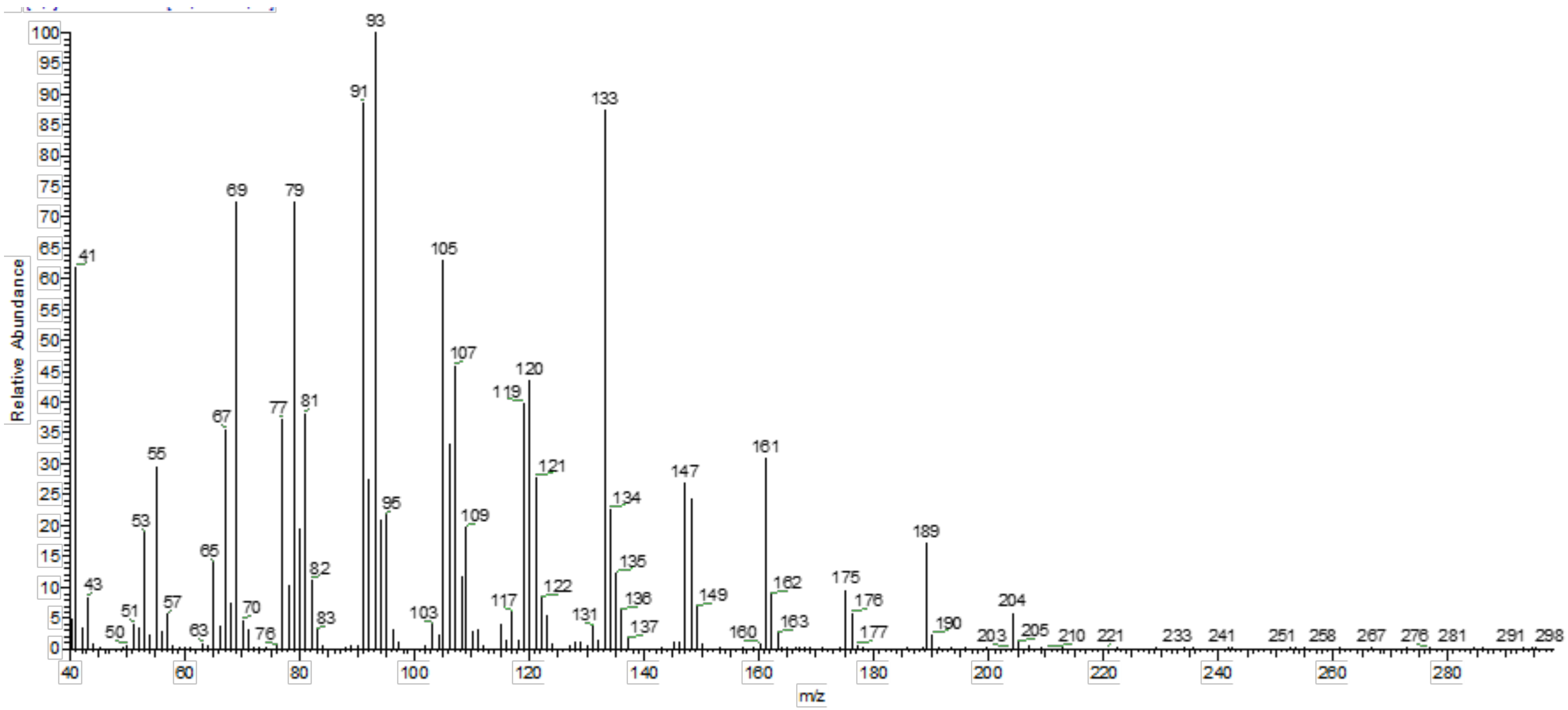


**Figure S 4.**Mass spectrum the fragmentation pattern of the major compound, E-caryophyllene (RT 28.61 min).

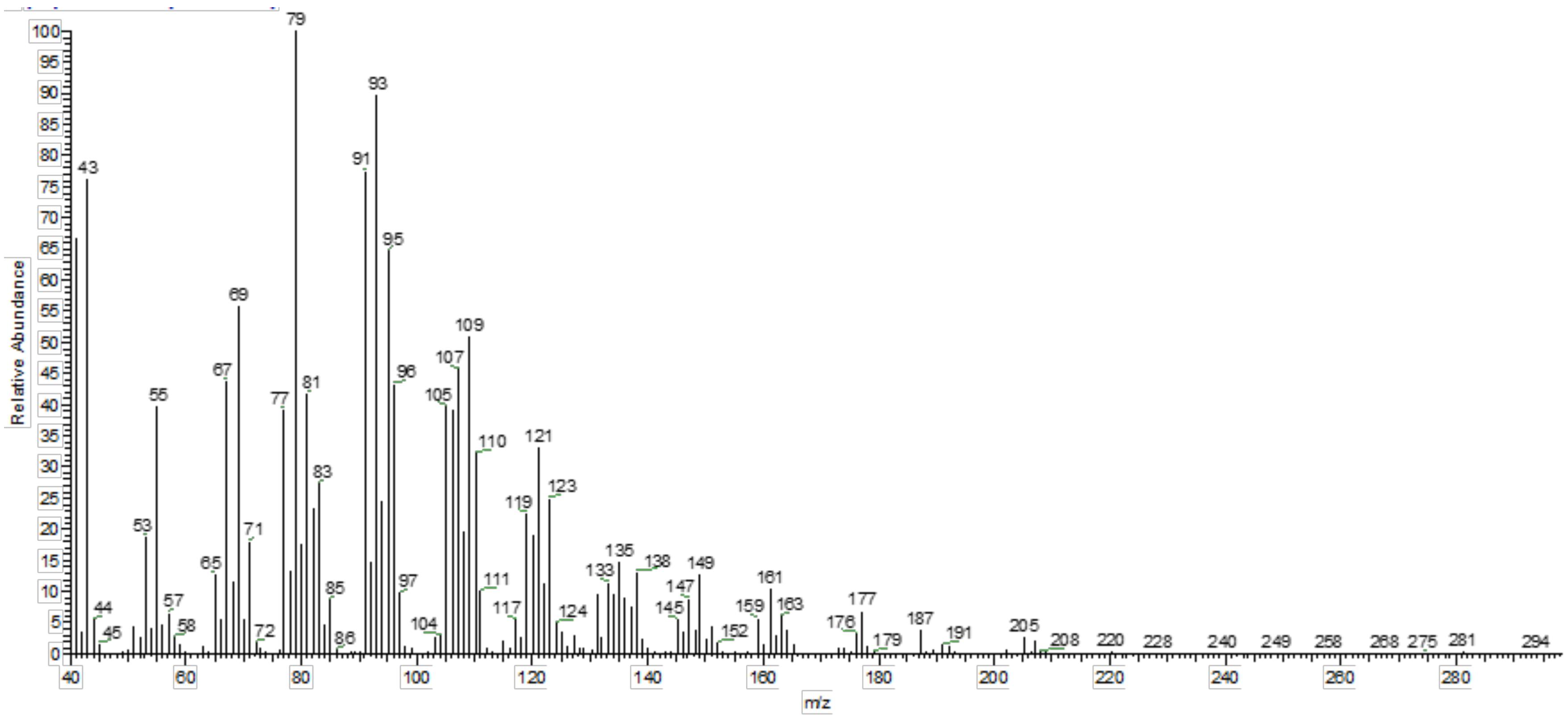


**Figure S 5.**Mass spectrum the fragmentation pattern of the major compound, caryophyllene oxide (RT 33.56 min).

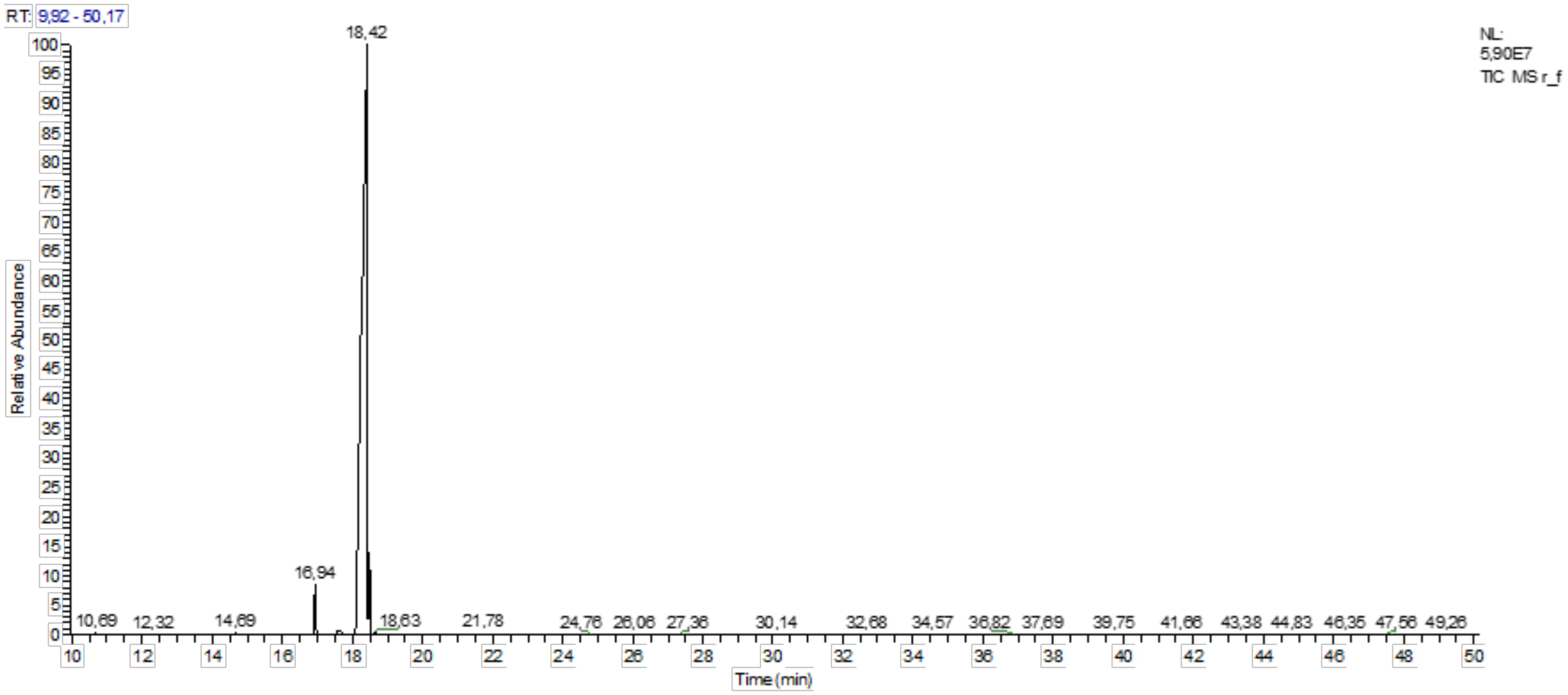


**Figure S 6.**Chromatogram of fenchol <endo> of the pure compound, chemical standard (Sigma-Aldrich).

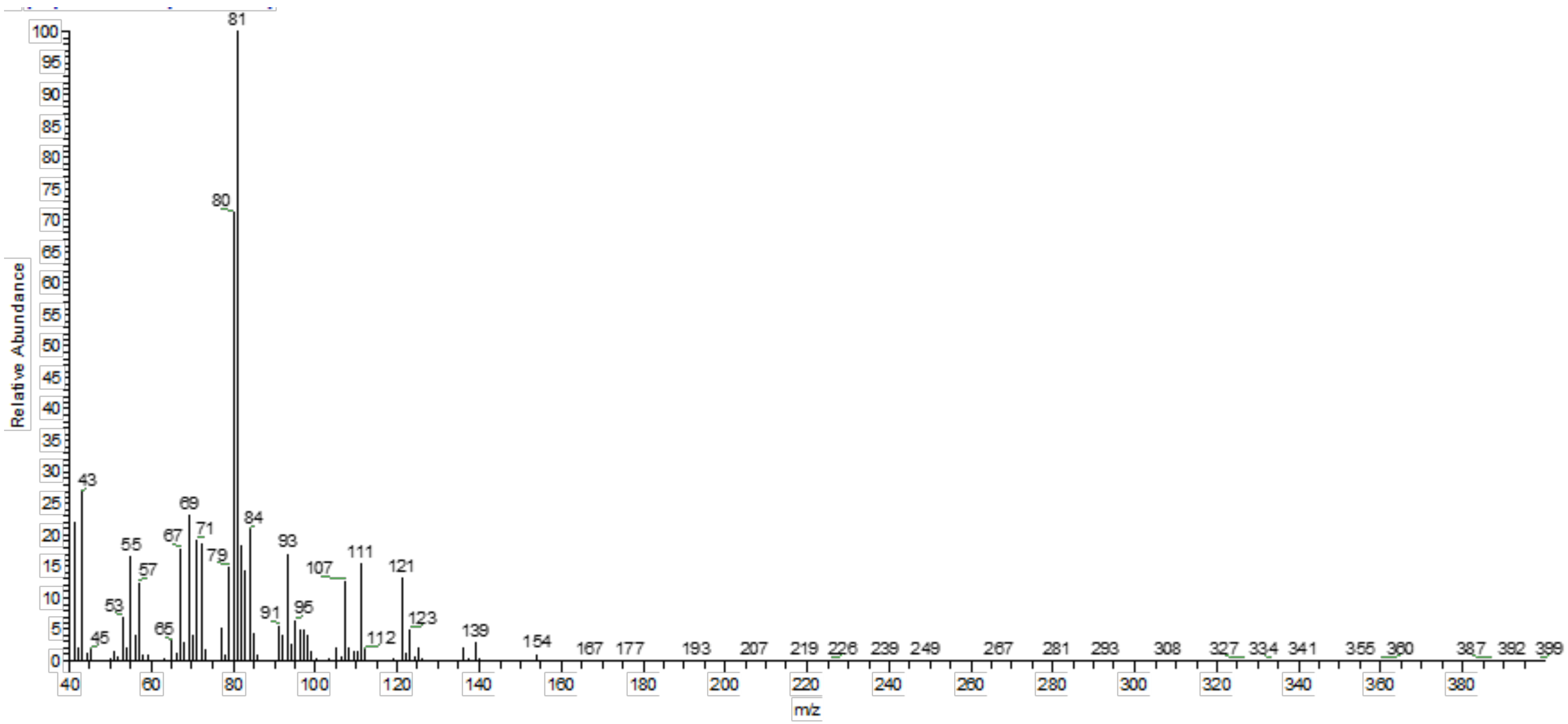


**Figure S 7.**Mass spectrum the fragmentation pattern of the fenchol <endo> from Sigma-Aldrich (TR 18.42 min).

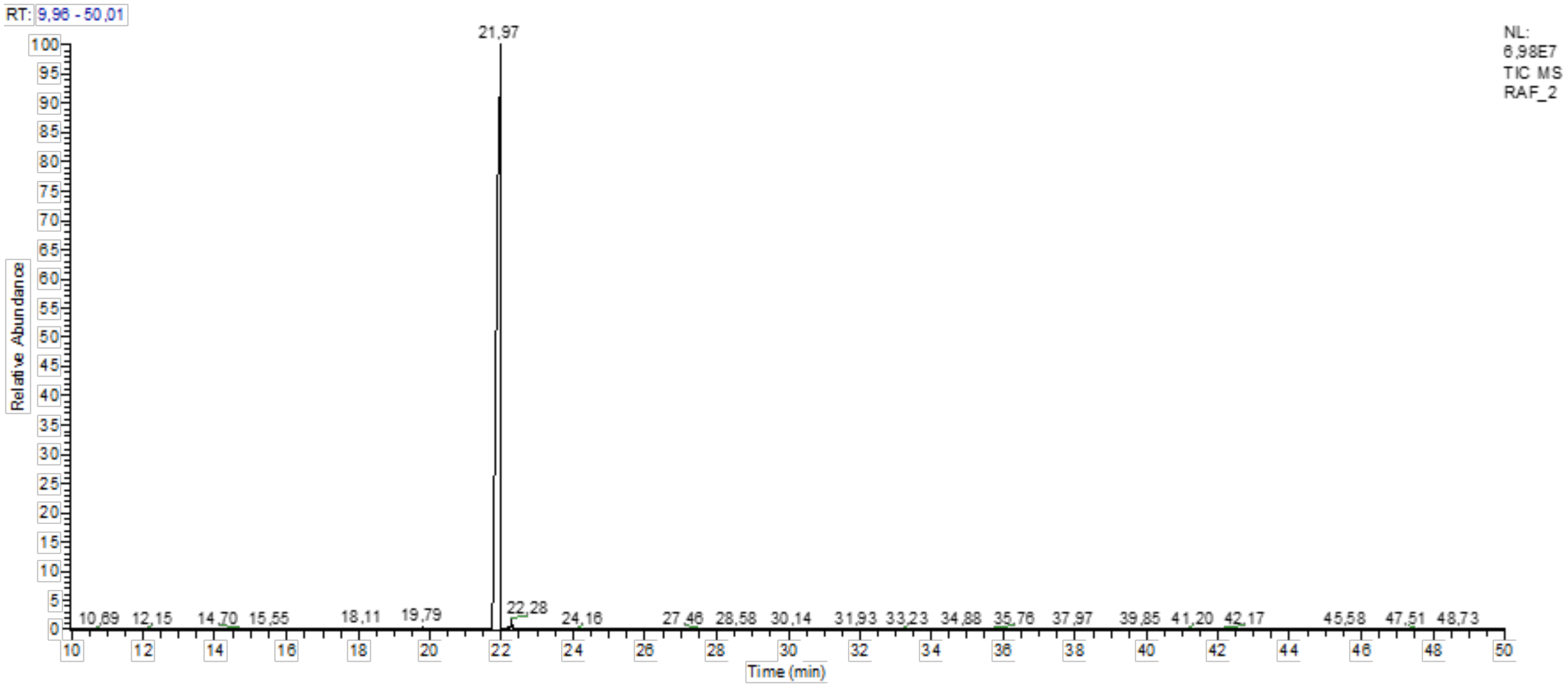


**Figure S 8.**Chromatogram of total ions of fenchyl acetate <endo> of the pure compound, chemical standard (Sigma-Aldrich).

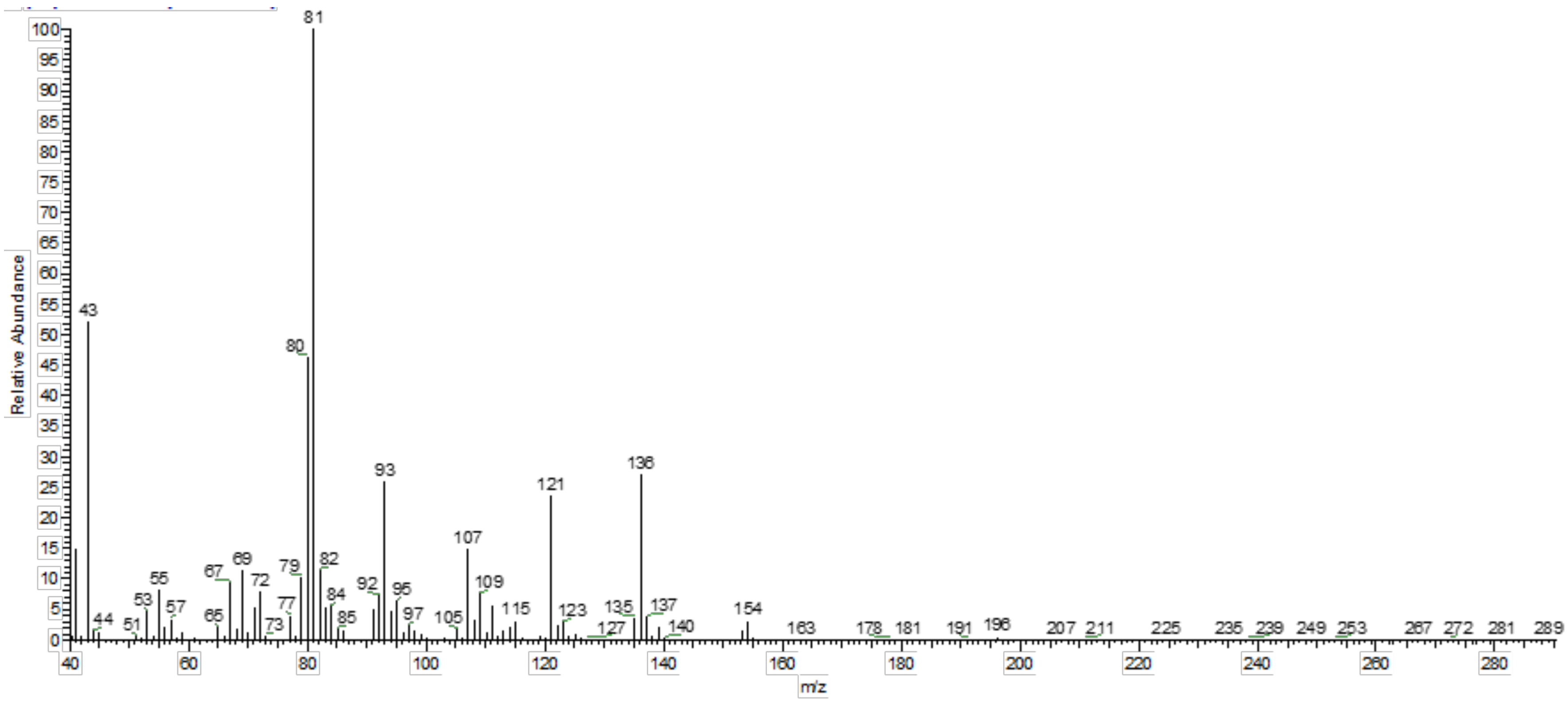


**Figure S 9**.Mass spectrum the fragmentation pattern of the fenchyl acetate <endo> from Sigma-Aldrich (TR 21.97 min).

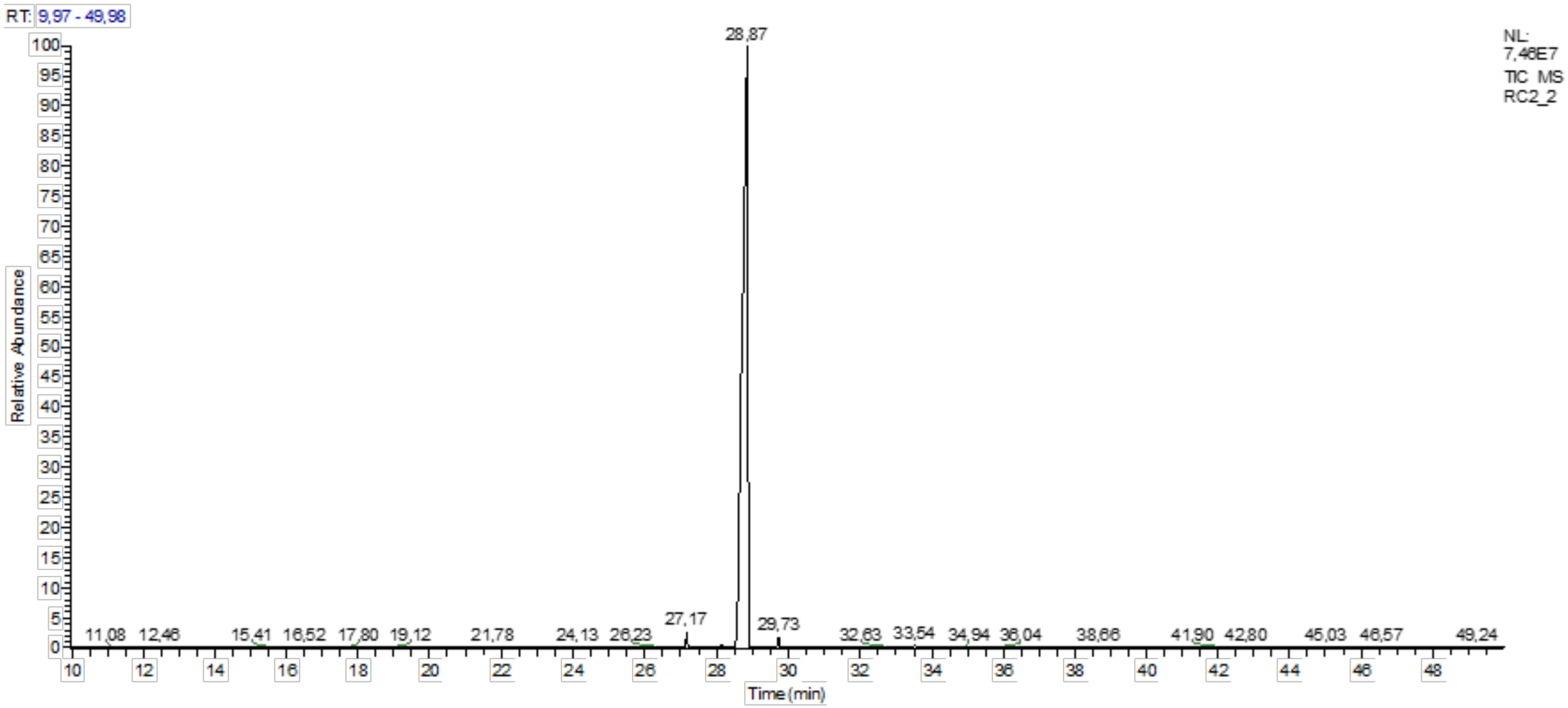


**Figure S 10**.Chromatogram of the total ions of E-caryophyllene of the pure compound, chemical standard (Sigma-Aldrich).

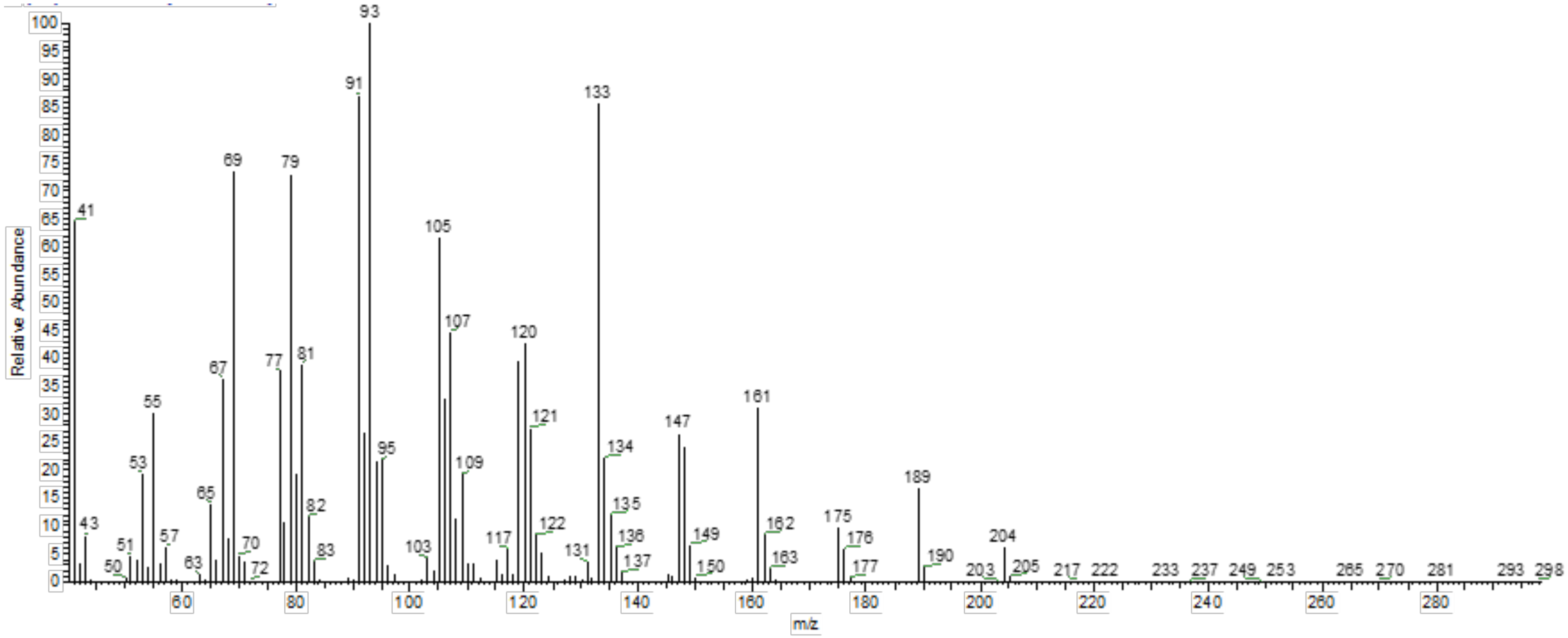


**Figure S 11**.Mass spectrum the fragmentation pattern of the E-caryophyllene from Sigma-Aldrich (TR 28.87 min).

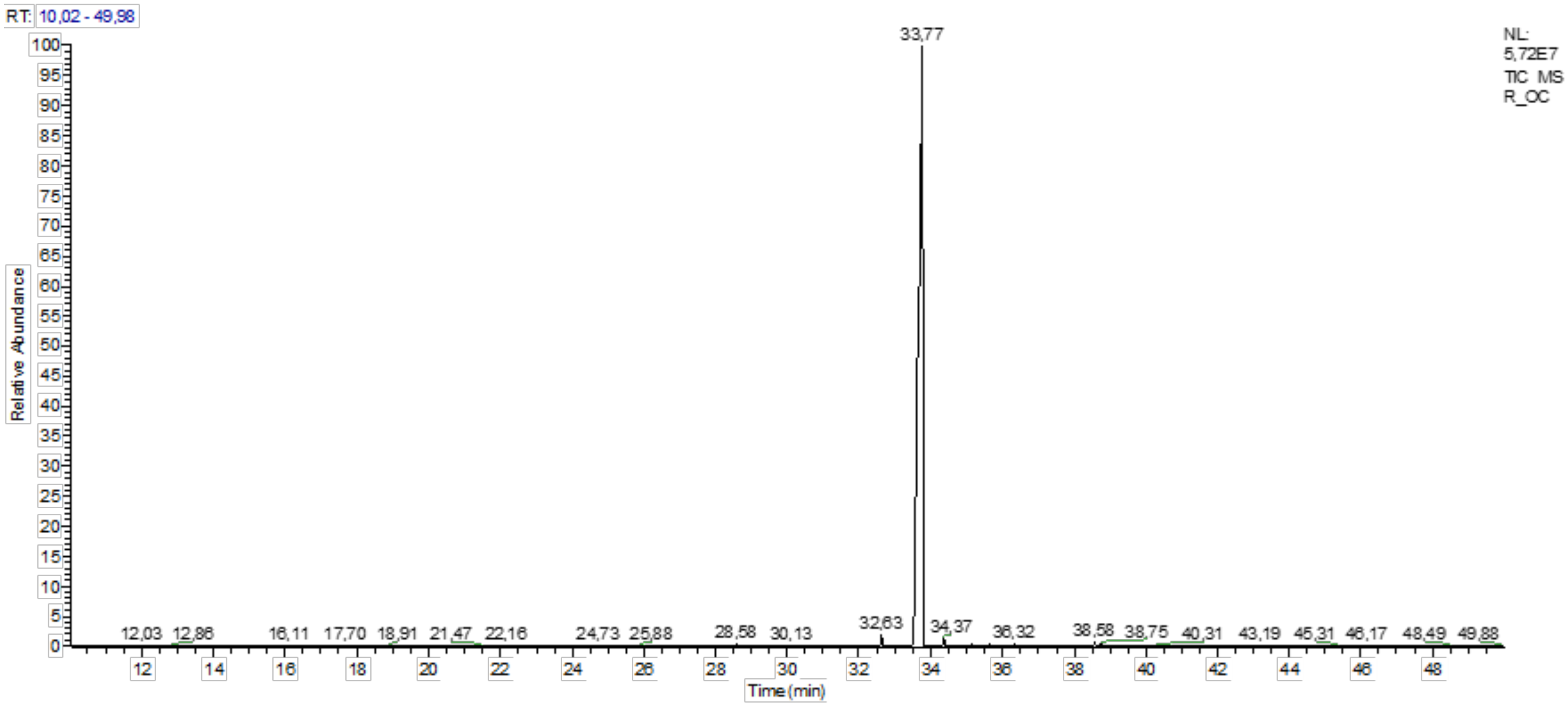


**Figure S 12.**Chromatogram of the total ions of caryophyllene oxide of the pure compound, chemical standard (Sigma-Aldrich).

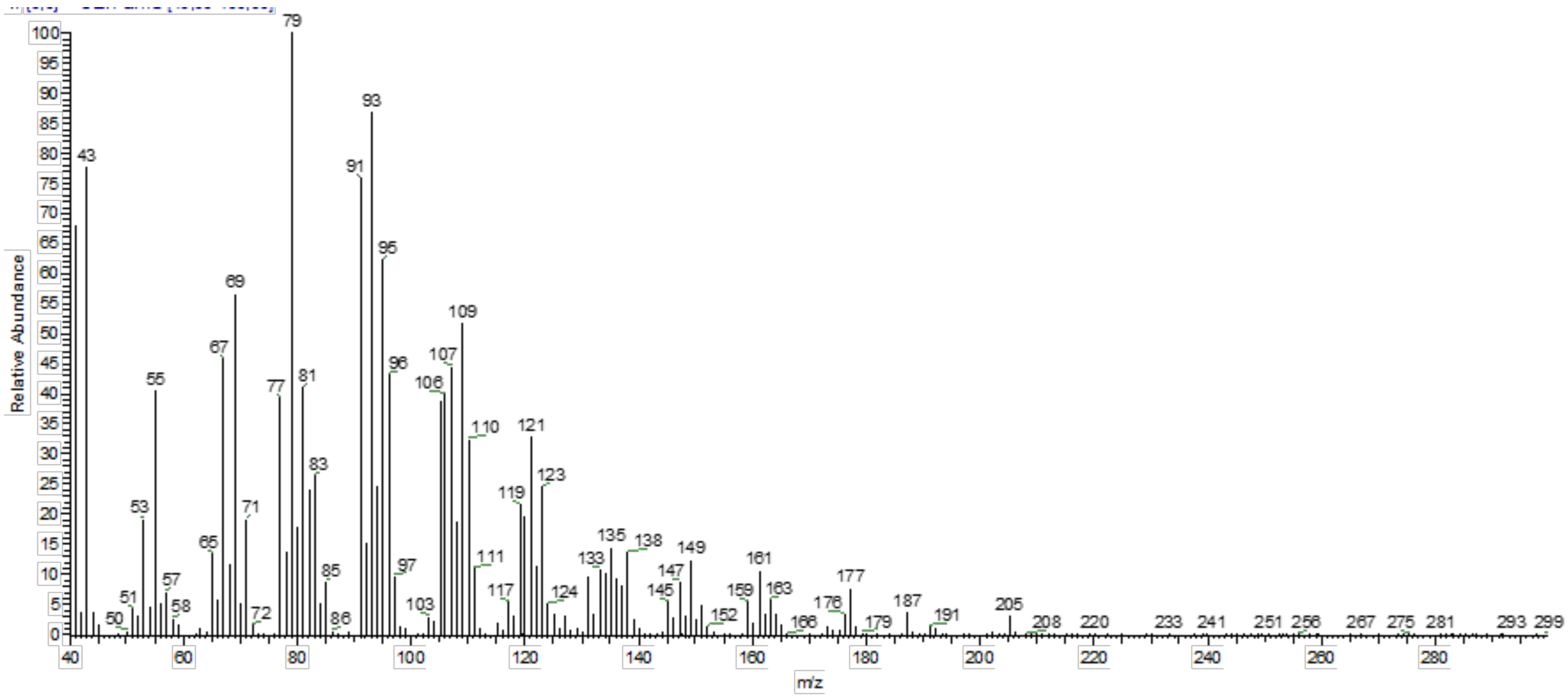


**Figure S 13**.Mass spectrum the fragmentation pattern of the caryophyllene oxide from Sigma-Aldrich (TR  33.77 min).

## Chemical profile: NMR 1D ($^1H$, $^{13}C$, DEPT-135) and 2D (COSY, HSQC, HMBC)

**Figure S 14.** $^1H$ NMR (500 MHz) spectrum of EODr in $CDCl_3$

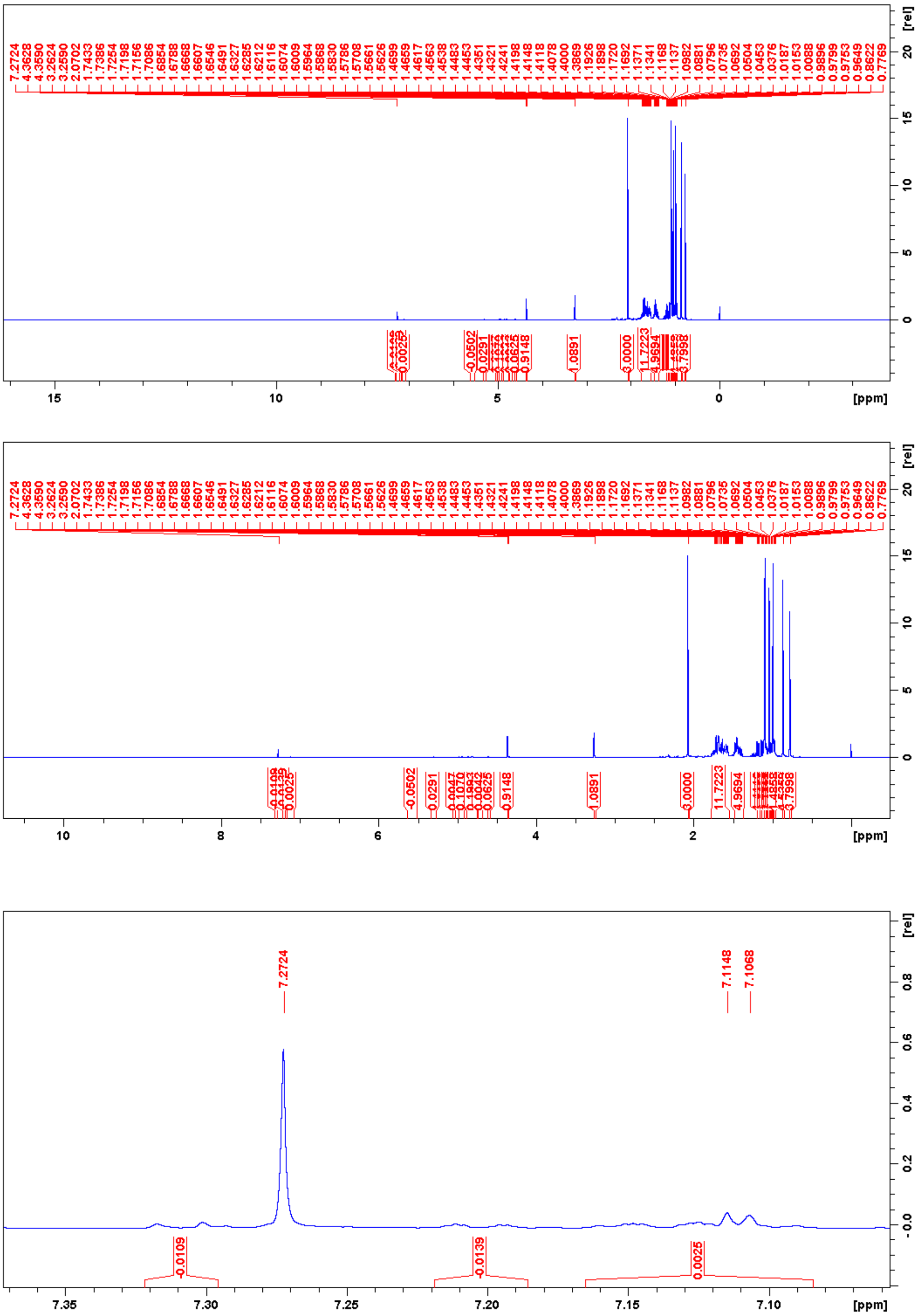

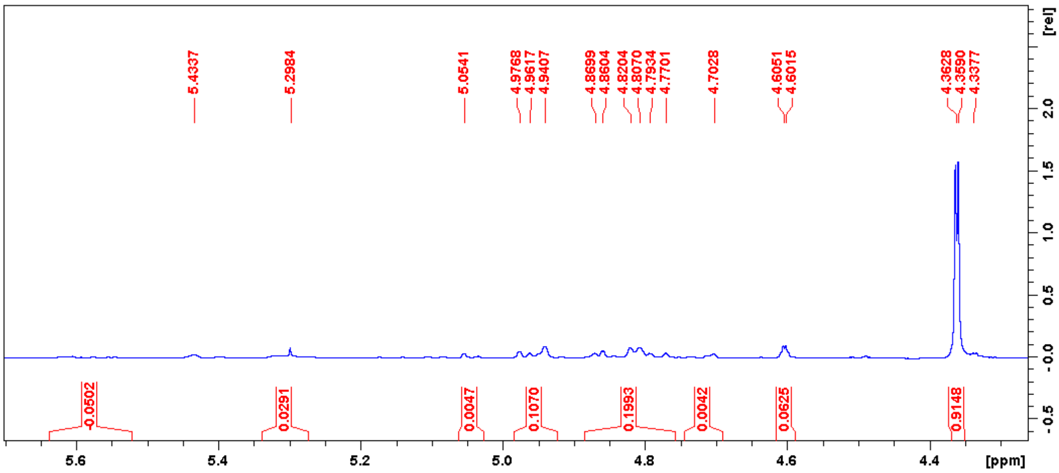
5.4337
5.2984
5.0541
4.9768
4.9617
4.9407
4.8699
4.8604
4.8204
4.8070
4.7934
4.7701
4.7028
4.6051
4.6015
4.3628
4.3590
4.3377
0.0502
0.0291
0.0047
0.1070
0.1993
0.0042
0.0625
0.9148
[rel]
[ppm]

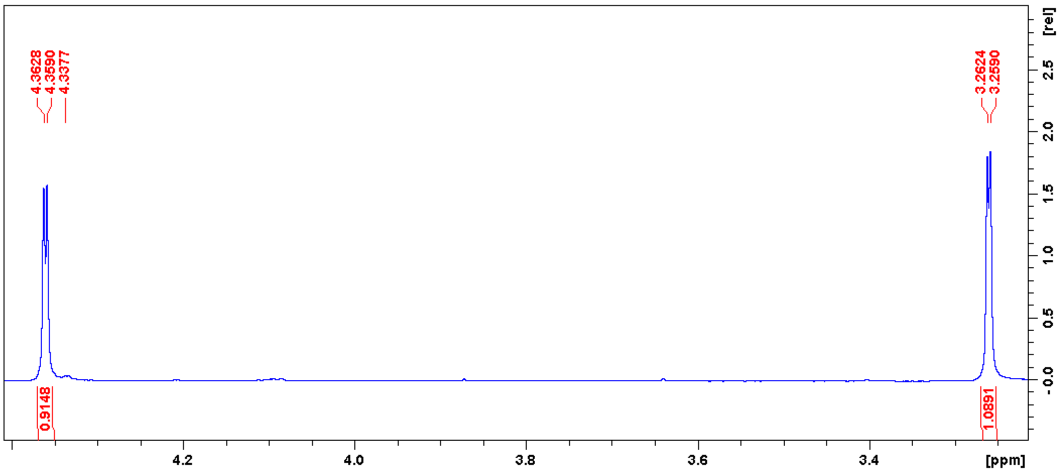
4.3628
4.3590
4.3377
3.2624
3.2590
0.9148
1.0891
[rel]
[ppm]

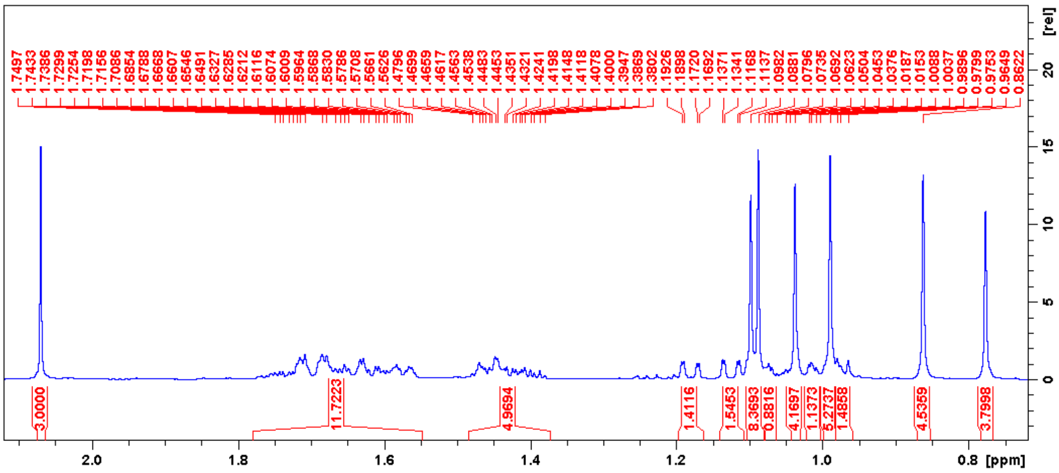
1.7497
1.7433
1.7386
1.7299
1.7254
1.7198
1.7156
1.7086
1.6854
1.6788
1.6668
1.6607
1.6546
1.6491
1.6327
1.6285
1.6212
1.6116
1.6074
1.6009
1.5964
1.5863
1.5830
1.5786
1.5708
1.5661
1.5626
1.4796
1.4699
1.4659
1.4617
1.4563
1.4538
1.4483
1.4453
1.4351
1.4321
1.4241
1.4198
1.4148
1.4118
1.4078
1.4000
1.3947
1.3869
1.3802
1.1926
1.1898
1.1720
1.1692
1.1371
1.1341
1.1168
1.1137
1.0982
1.0881
1.0796
1.0735
1.0692
1.0623
1.0504
1.0453
1.0376
1.0187
1.0153
1.0088
1.0037
0.9896
0.9799
0.9753
0.9649
0.8622
3.0000
11.7223
4.9694
1.4116
1.5453
8.3693
0.8816
4.1697
1.1373
5.2737
1.4858
4.5359
3.7998
[rel]
[ppm]

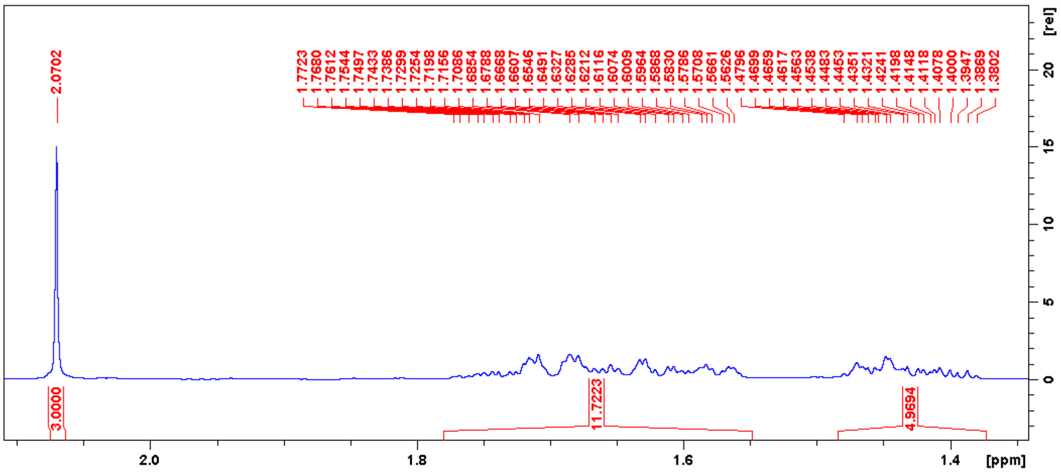
2.0702
1.7723 1.7680 1.7612 1.7544 1.7497 1.7433 1.7386 1.7299 1.7254 1.7198 1.7156 1.7086 1.6854 1.6788 1.6668 1.6607 1.6546 1.6491 1.6327 1.6285 1.6212 1.6116 1.6074 1.6009 1.5964 1.5868 1.5830 1.5786 1.5708 1.5661 1.5626 1.4796 1.4699 1.4659 1.4617 1.4563 1.4538 1.4483 1.4453 1.4351 1.4321 1.4241 1.4198 1.4148 1.4118 1.4078 1.4000 1.3947 1.3869 1.3802
3.0000
11.7223
4.9694
[rel]
[ppm]

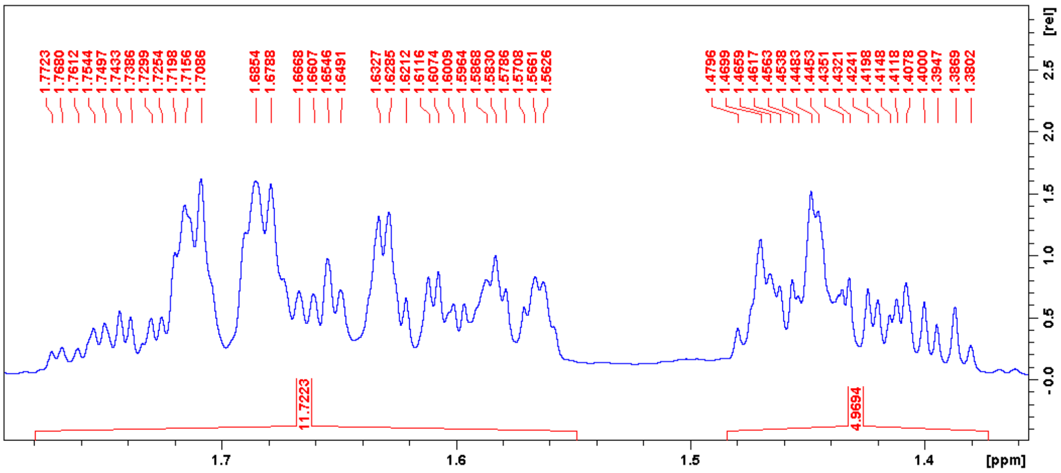
1.7723 1.7680 1.7612 1.7544 1.7497 1.7433 1.7386 1.7299 1.7254 1.7198 1.7156 1.7086 1.6854 1.6788 1.6668 1.6607 1.6546 1.6491 1.6327 1.6285 1.6212 1.6116 1.6074 1.6009 1.5964 1.5868 1.5830 1.5786 1.5708 1.5661 1.5626
1.4796 1.4699 1.4659 1.4617 1.4563 1.4538 1.4483 1.4453 1.4351 1.4321 1.4241 1.4198 1.4148 1.4118 1.4078 1.4000 1.3947 1.3869 1.3802
11.7223
4.9694
[rel]
[ppm]

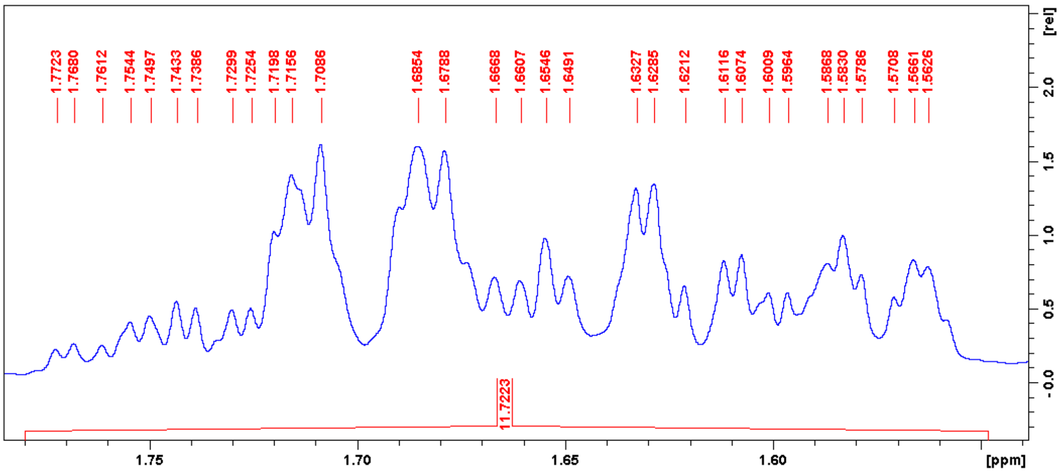
1.7723 1.7680 1.7612 1.7544 1.7497 1.7433 1.7386 1.7299 1.7254 1.7198 1.7156 1.7086 1.6854 1.6788 1.6668 1.6607 1.6546 1.6491 1.6327 1.6285 1.6212 1.6116 1.6074 1.6009 1.5964 1.5868 1.5830 1.5786 1.5708 1.5661 1.5626
11.7223
[rel]
[ppm]

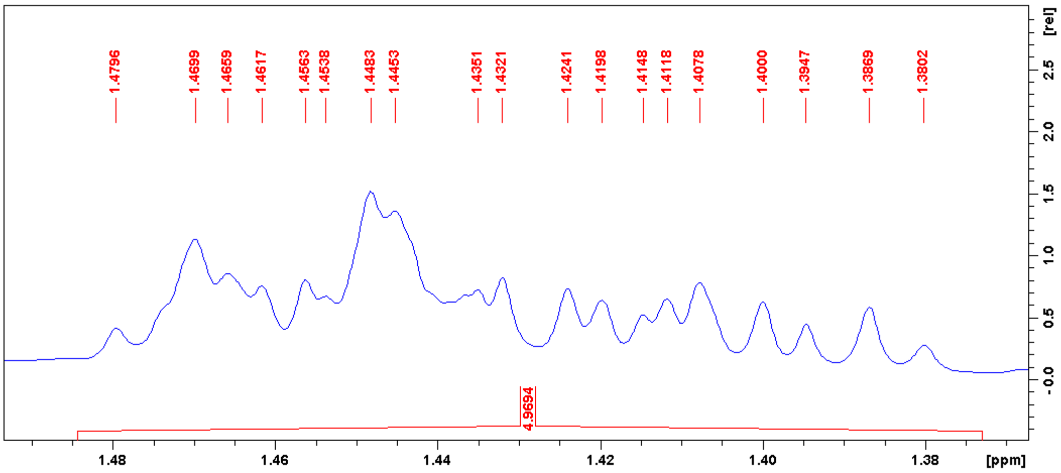

1.4796 1.4699 1.4659 1.4617 1.4563 1.4538 1.4483 1.4453 1.4351 1.4321 1.4241 1.4198 1.4148 1.4118 1.4078 1.4000 1.3947 1.3869 1.3802
4.9694
1.48 1.46 1.44 1.42 1.40 1.38 [ppm]
[rel]


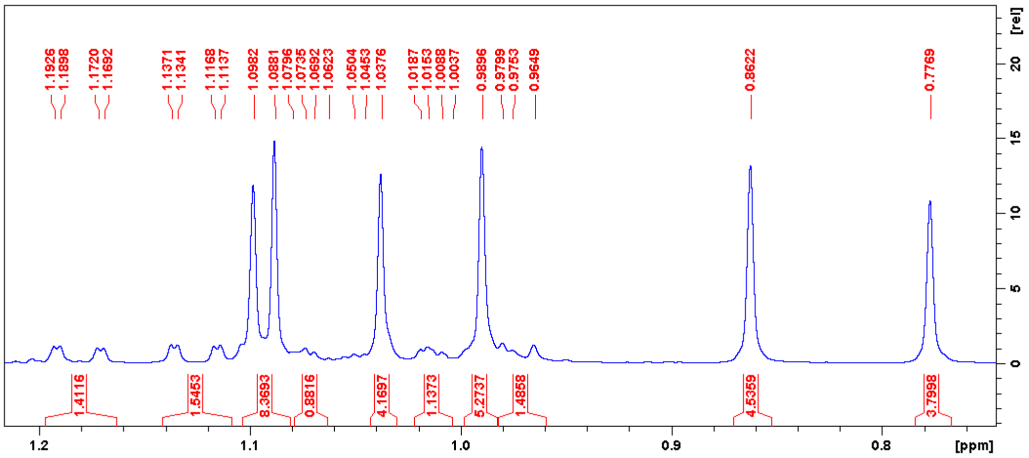

1.1926 1.1898 1.1720 1.1692 1.1371 1.1341 1.1168 1.1137 1.0982 1.0881 1.0796 1.0735 1.0692 1.0623 1.0504 1.0453 1.0376 1.0187 1.0153 1.0088 1.0037 0.9896 0.9799 0.9753 0.9649 0.8622 0.7769
1.4116 1.5453 8.3693 0.8816 4.1697 1.1373 5.2737 1.4858 4.5359 3.7998
1.2 1.1 1.0 0.9 0.8 [ppm]
[rel]

**Figure S 15.**$^{13}$C NMR (125 MHz) spectrum of EODr in $CDCl_3$

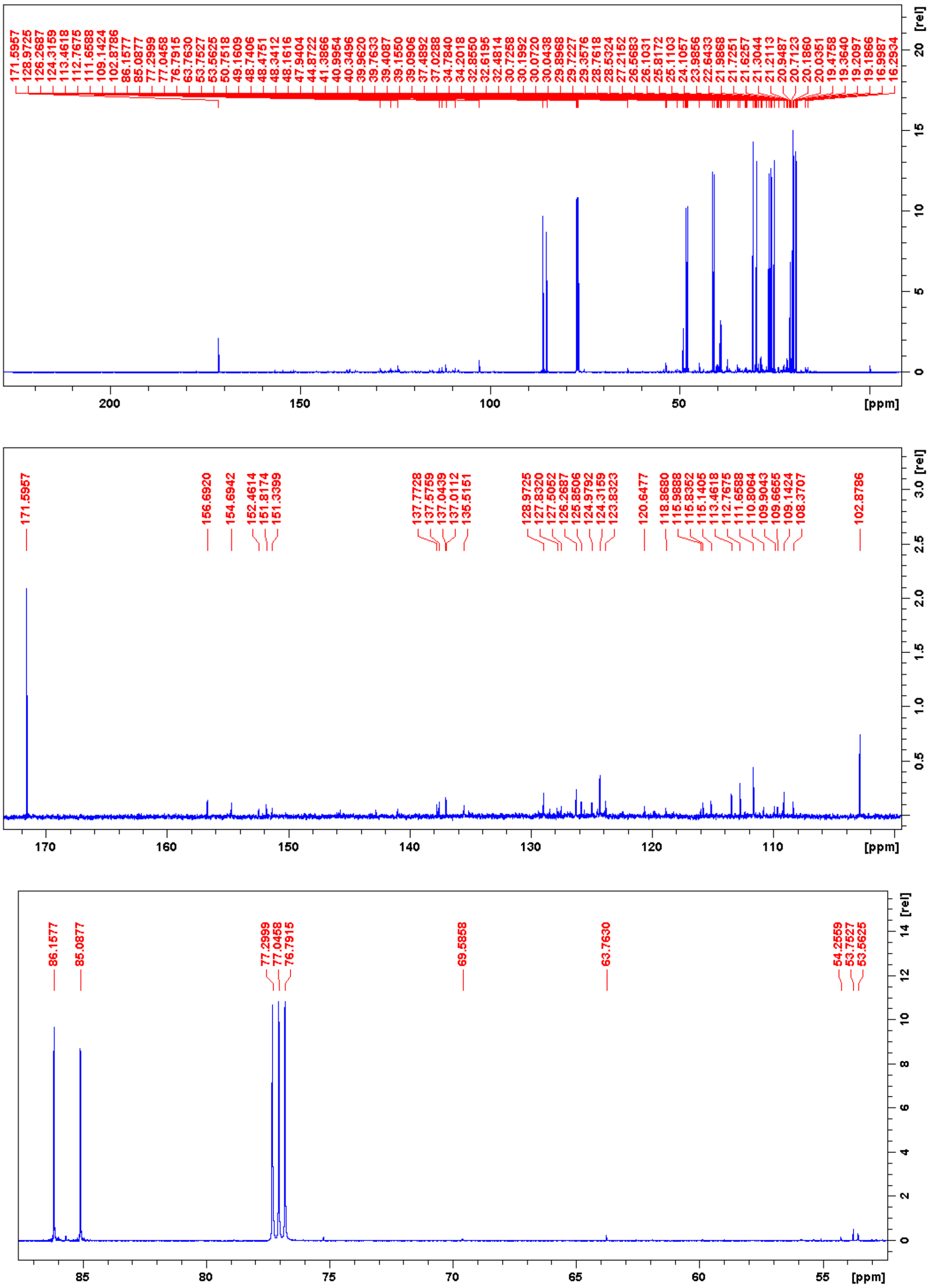

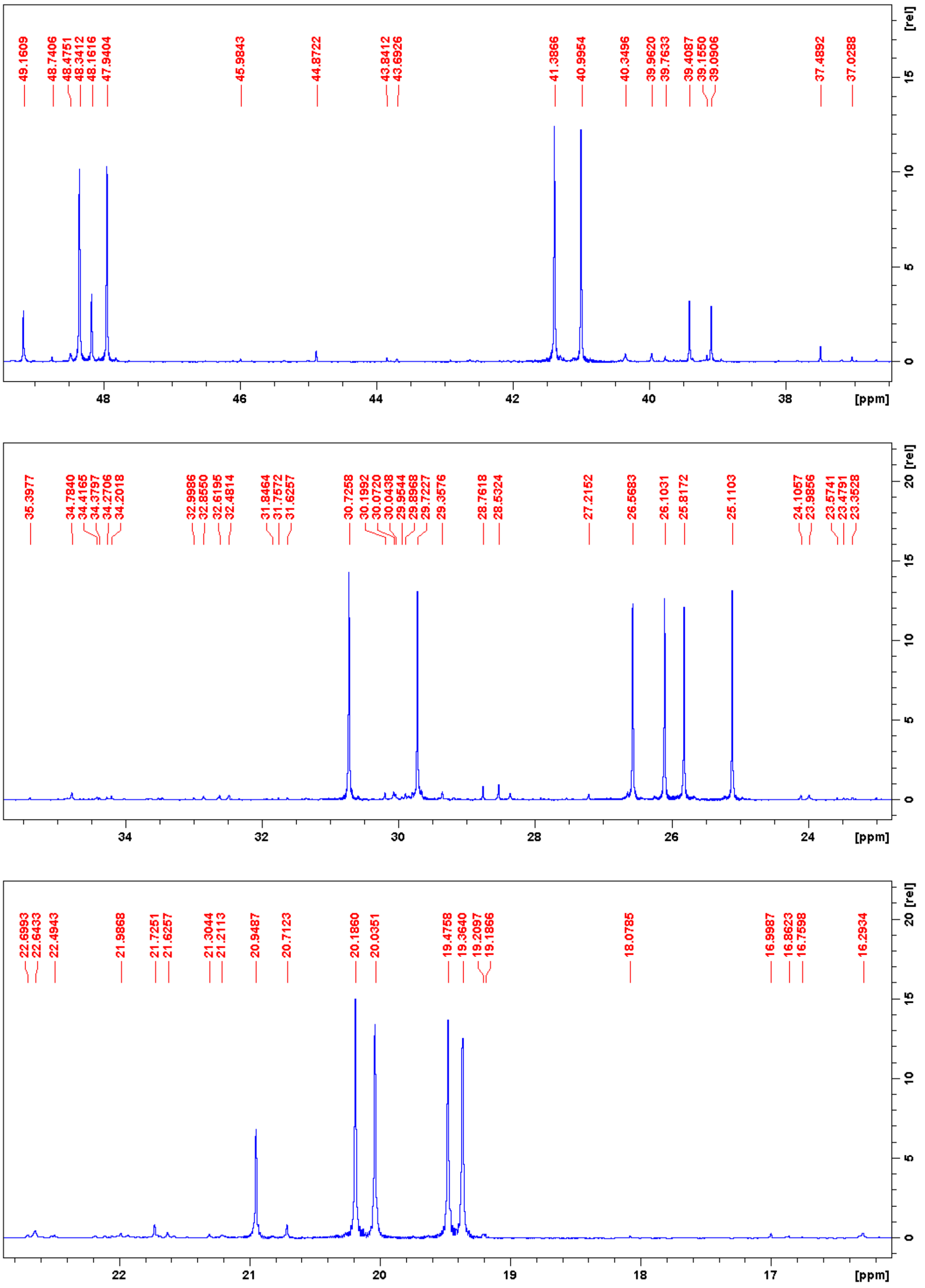
49.1609
48.7406
48.4751
48.3412
48.1616
47.9404
45.9843
44.8722
43.8412
43.6926
41.3866
40.9954
40.3496
39.9620
39.7633
39.4087
39.1550
39.0906
37.4892
37.0288
[rel]
[ppm]
35.3977
34.7840
34.4165
34.3797
34.2706
34.2018
32.9986
32.8550
32.6195
32.4814
31.8464
31.7572
31.6257
30.7258
30.1992
30.0720
30.0438
29.9544
29.8968
29.7227
29.3576
28.7618
28.5324
27.2152
26.5683
26.1031
25.8172
25.1103
24.1057
23.9856
23.5741
23.4791
23.3528
22.6993
22.6433
22.4943
21.9868
21.7251
21.6257
21.3044
21.2113
20.9487
20.7123
20.1860
20.0351
19.4758
19.3640
19.2097
19.1866
18.0785
16.9987
16.8623
16.7598
16.2934

**Figure S 16.**$^{13}$C NMR and DEPT 135 (125 MHz) spectrum of EODr in $CDCl_3$

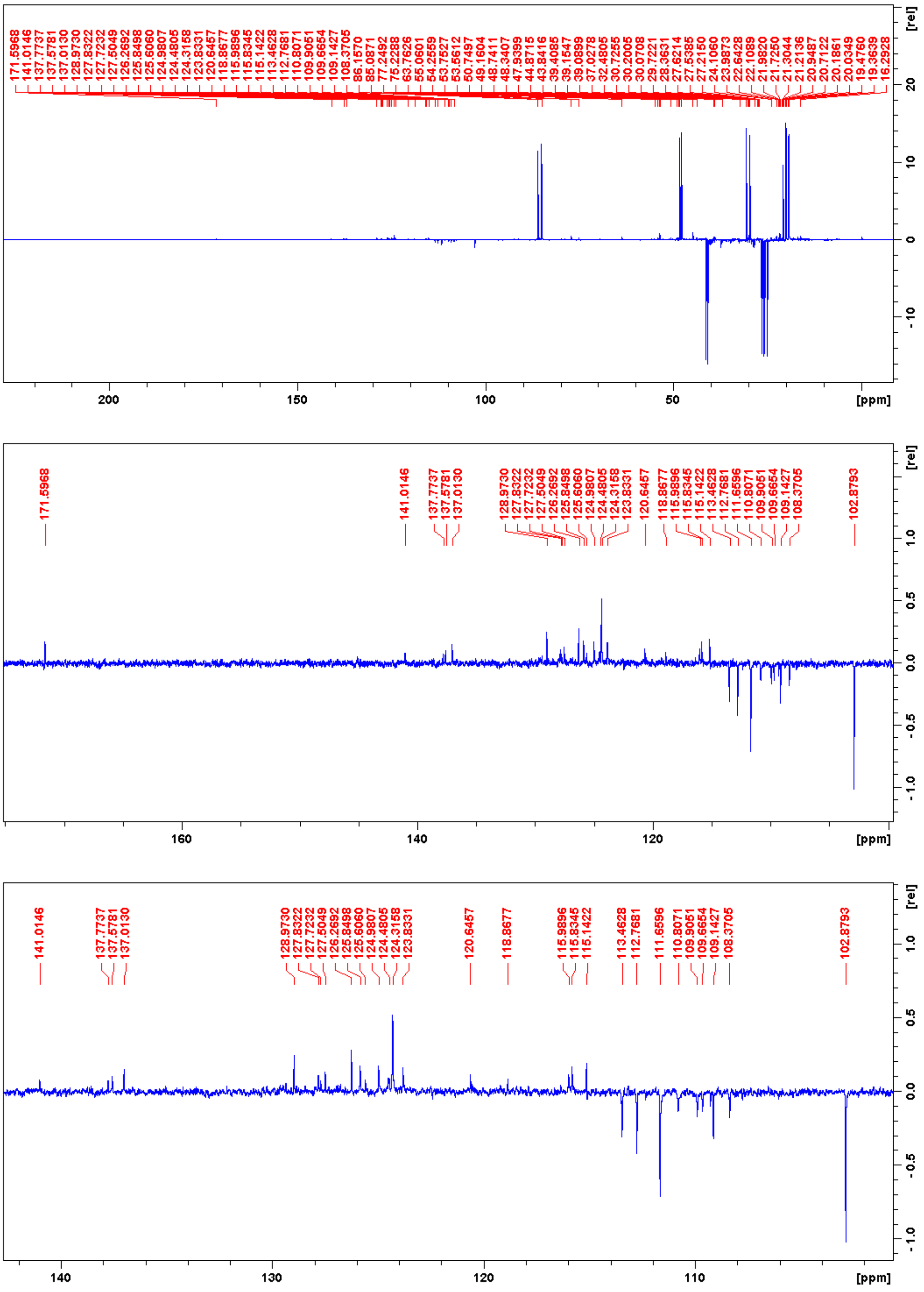

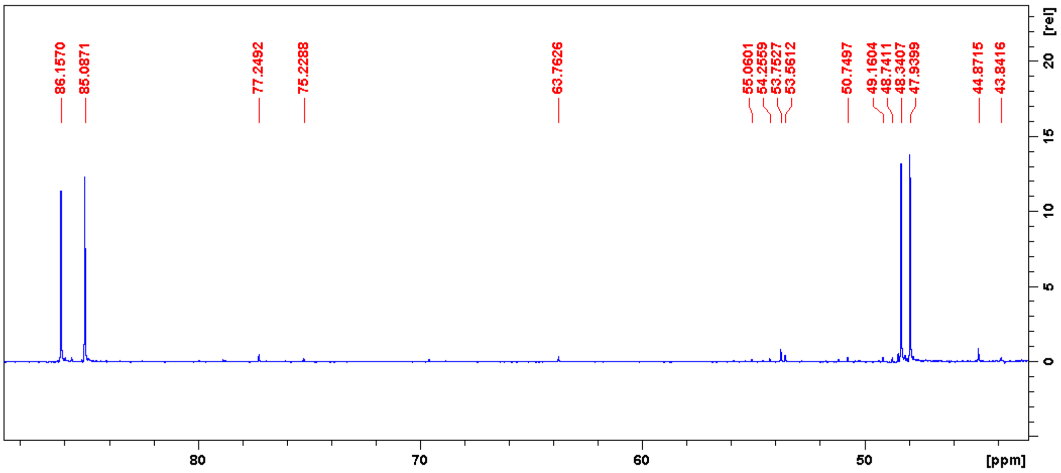
86.1570
85.0871
77.2492
75.2288
63.7626
55.0601
54.2559
53.7527
53.5612
50.7497
49.1604
48.7411
48.3407
47.9399
44.8715
43.8416
80
70
60
50
[ppm]
[rel]

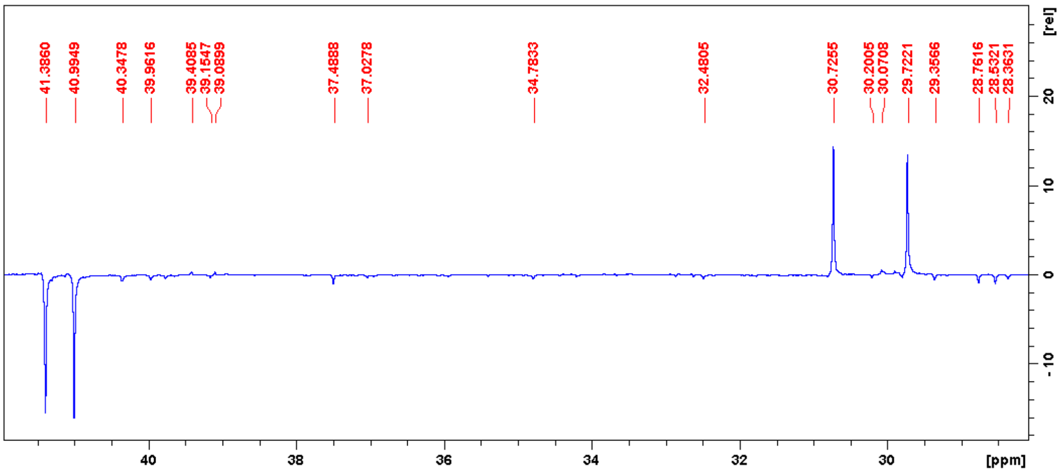
41.3860
40.9949
40.3478
39.9616
39.4085
39.1547
39.0899
37.4888
37.0278
34.7833
32.4805
30.7255
30.2005
30.0708
29.7221
29.3566
28.7616
28.5321
28.3631
40
38
36
34
32
30
[ppm]
[rel]

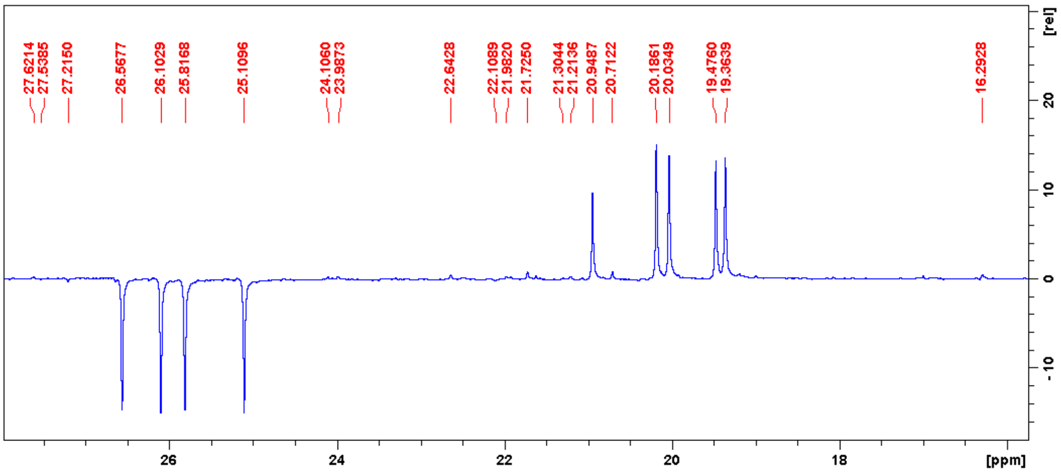
27.6214
27.5385
27.2150
26.5677
26.1029
25.8168
25.1096
24.1060
23.9873
22.6428
22.1089
21.9820
21.7250
21.3044
21.2136
20.9487
20.7122
20.1861
20.0349
19.4760
19.3639
16.2928
26
24
22
20
18
[ppm]
[rel]

**Figure S 17.**$^{1}$H-$^{1}$H-COSY (500 MHz) spectrum of EODr in $CDCl_3$

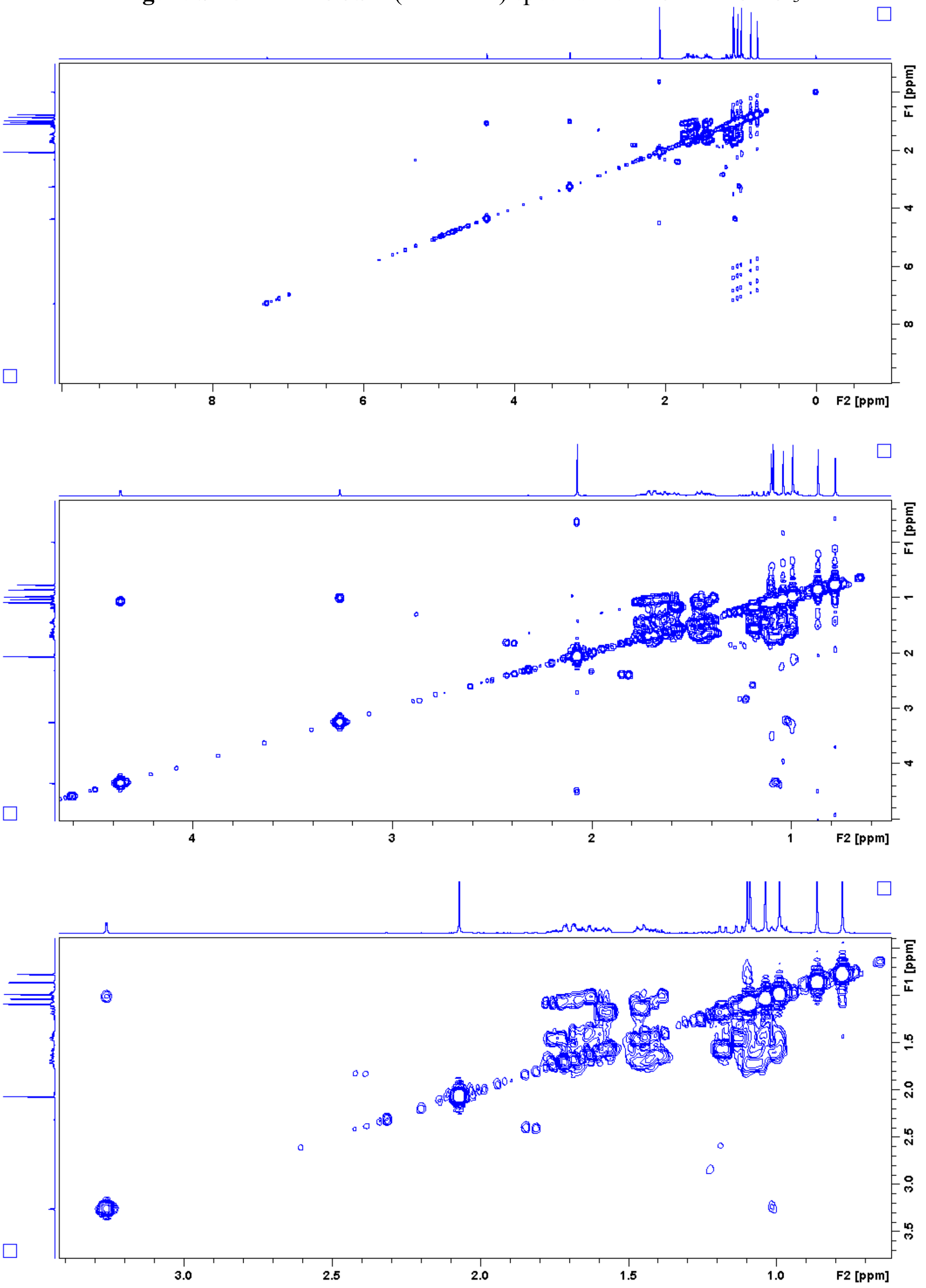

**Figure S 18**.-$^{13}$C$^{1}$H -HSQC ($^{1}$H 500 MHz; $^{13}$C 125 MHz) spectrum of EODr in $CDCl_3$

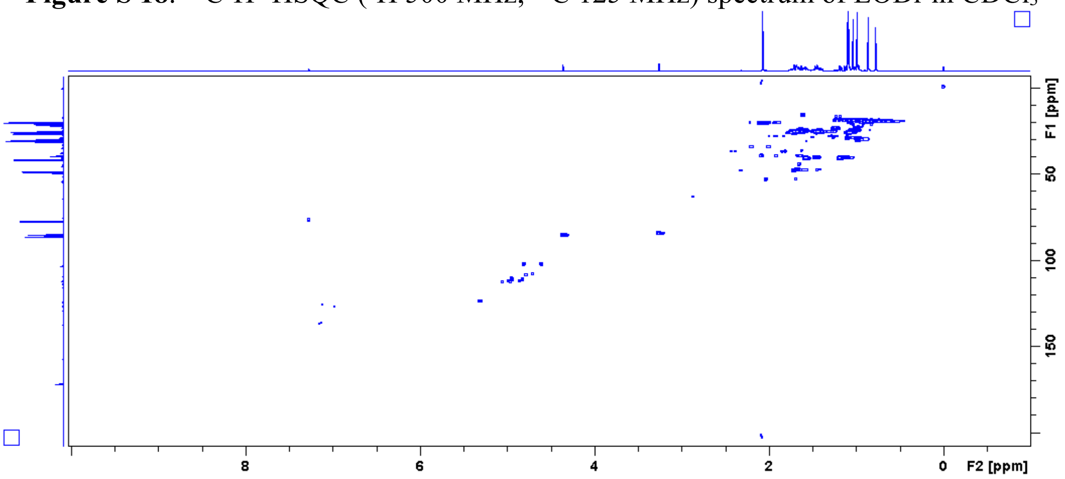


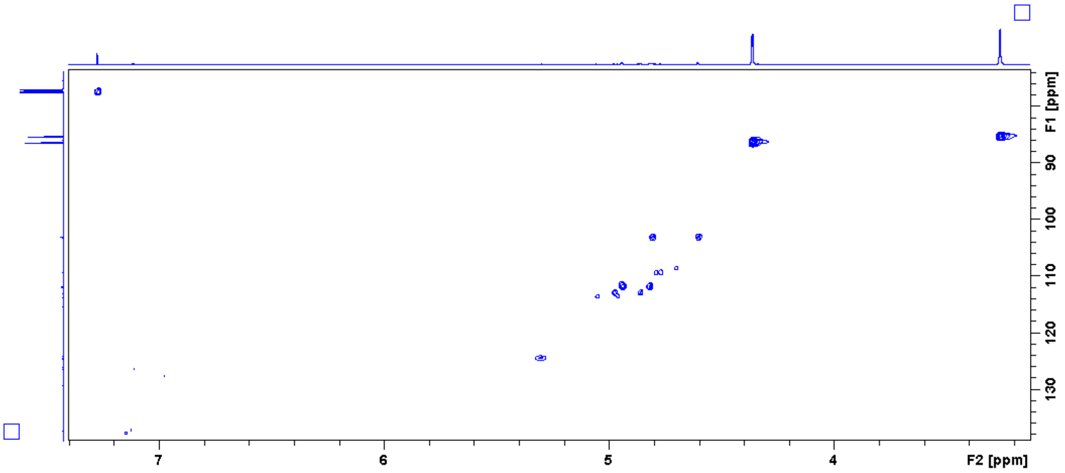


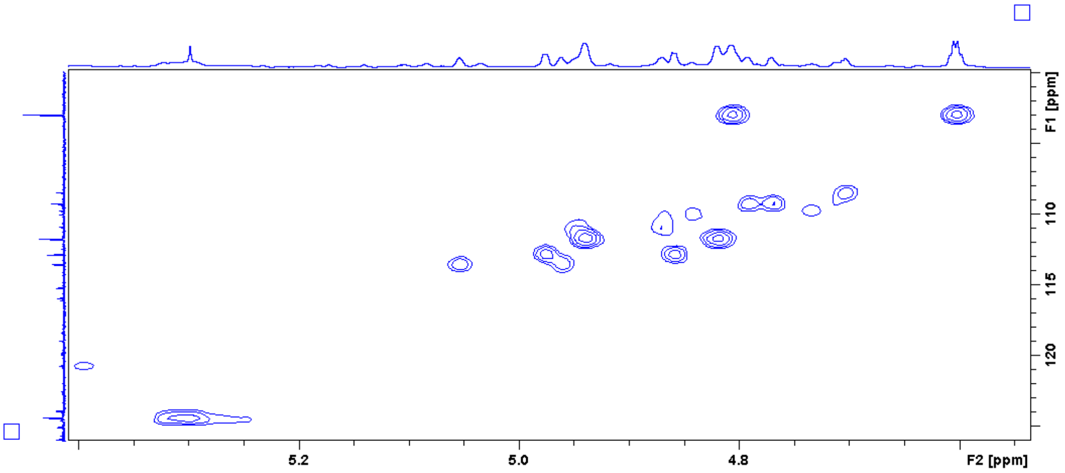

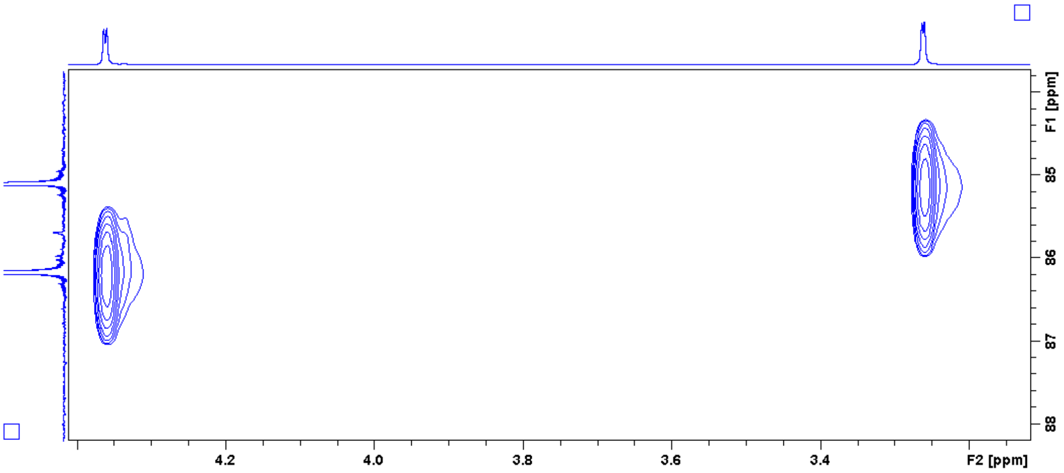

F1 [ppm]
85
86
87
88
4.2
4.0
3.8
3.6
3.4
F2 [ppm]


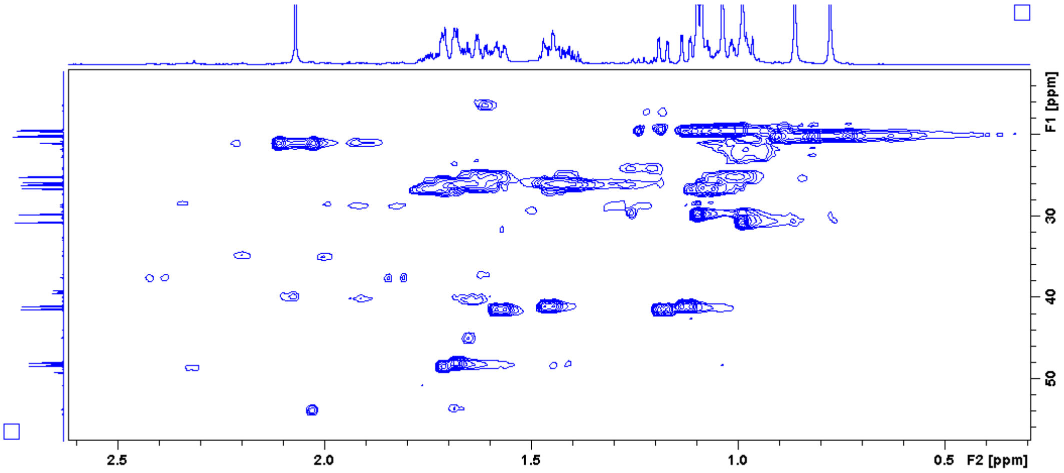

F1 [ppm]
30
40
50
2.5
2.0
1.5
1.0
0.5
F2 [ppm]


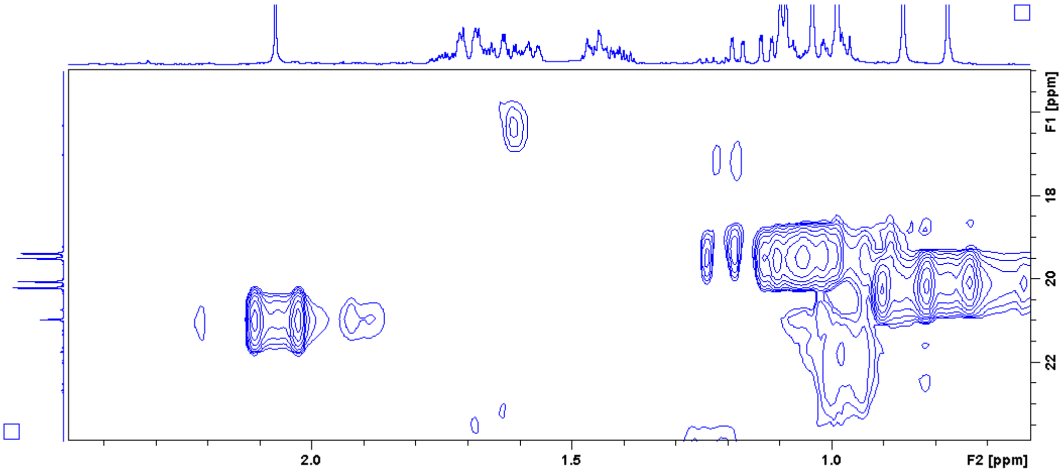

F1 [ppm]
18
20
22
2.0
1.5
1.0
F2 [ppm]

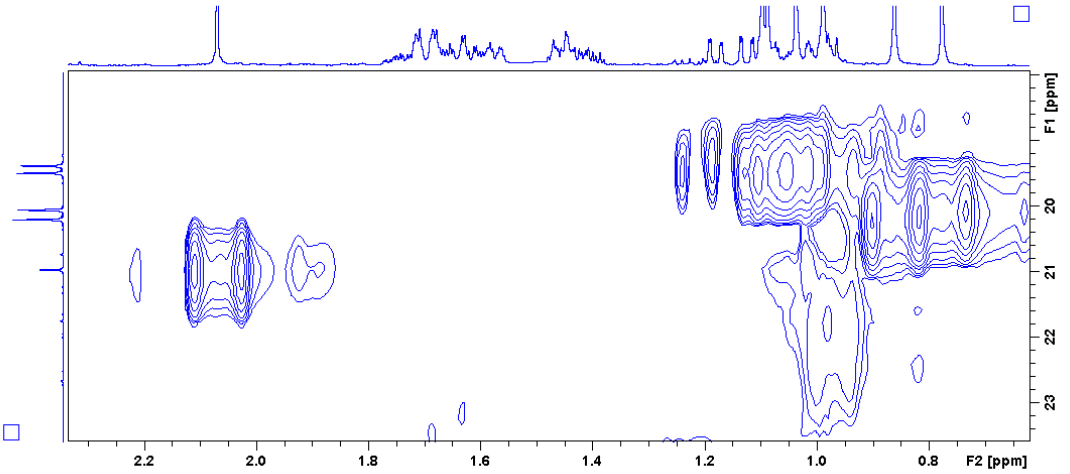
F1 [ppm]
20
21
22
23
2.2
2.0
1.8
1.6
1.4
1.2
1.0
0.8
F2 [ppm]

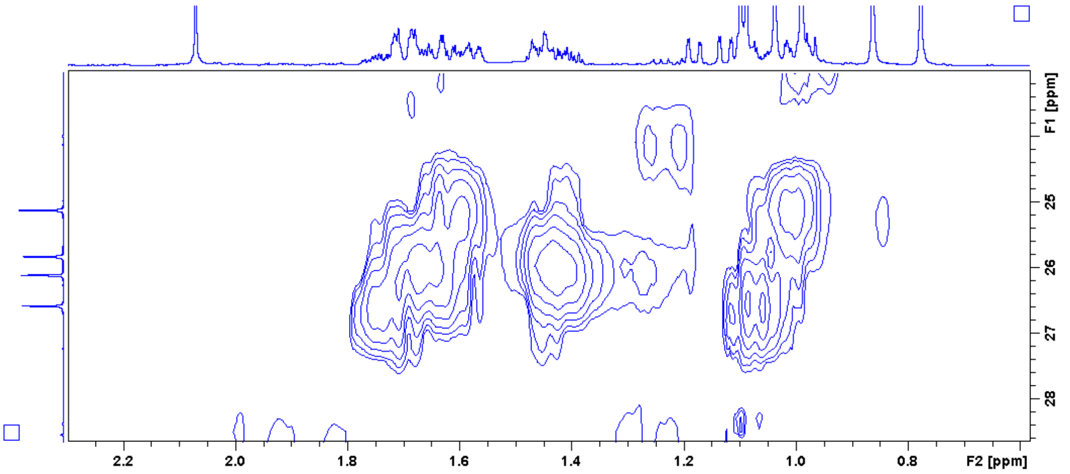
F1 [ppm]
25
26
27
28
2.2
2.0
1.8
1.6
1.4
1.2
1.0
0.8
F2 [ppm]

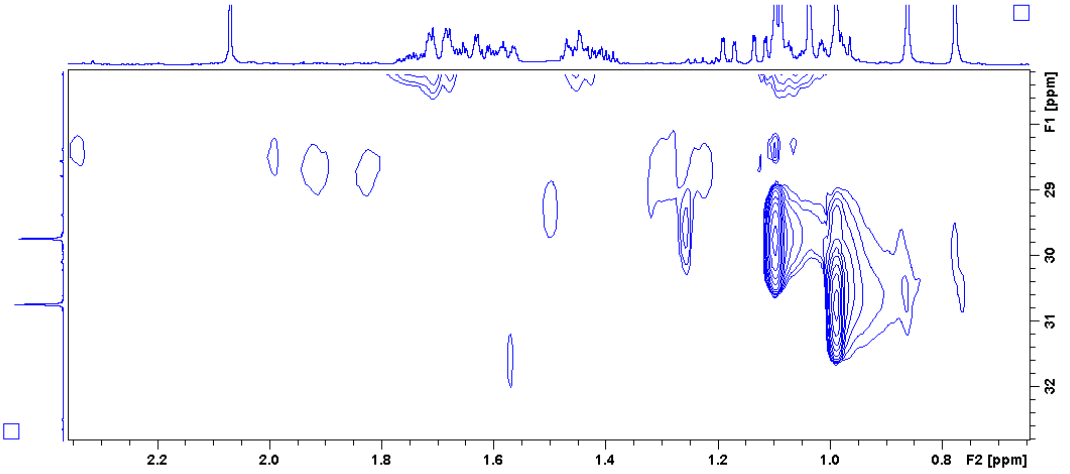
F1 [ppm]
29
30
31
32
2.2
2.0
1.8
1.6
1.4
1.2
1.0
0.8
F2 [ppm]

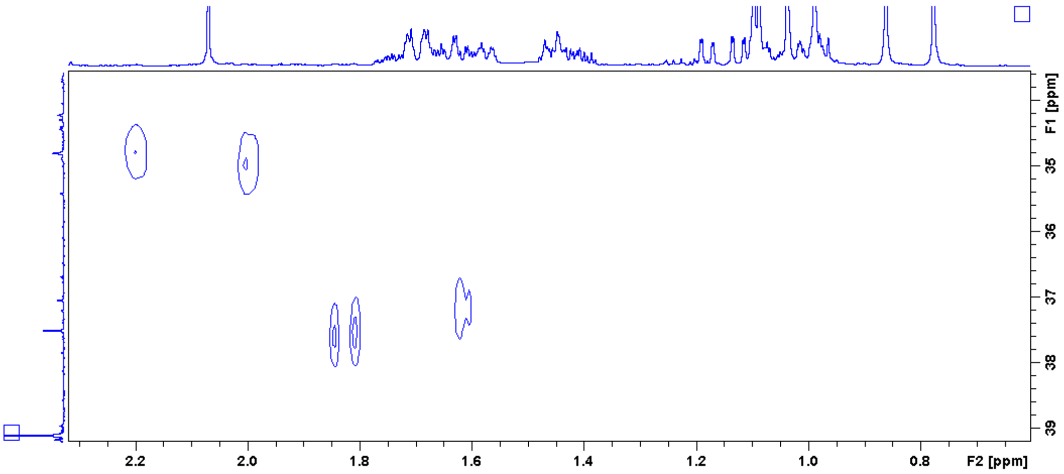
F1 [ppm]
35
36
37
38
39
2.2
2.0
1.8
1.6
1.4
1.2
1.0
0.8
F2 [ppm]

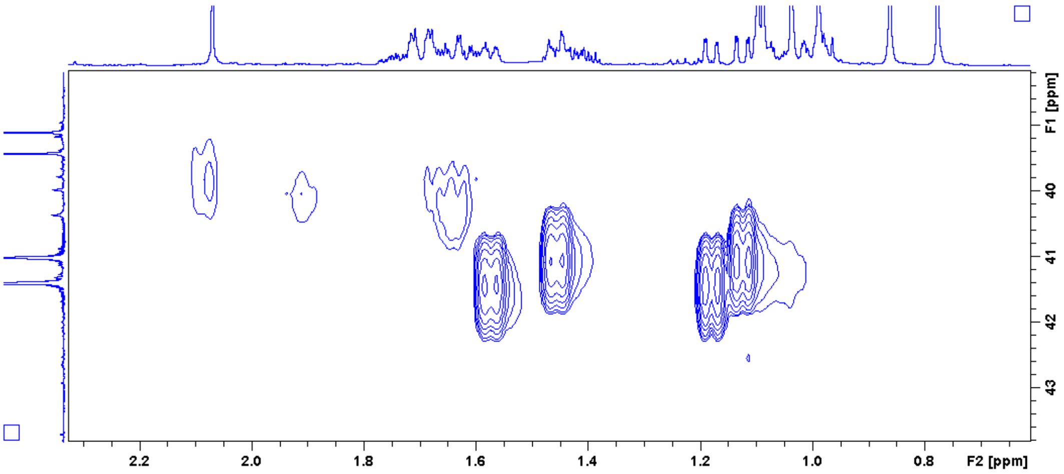
F1 [ppm]
40
41
42
43
2.2
2.0
1.8
1.6
1.4
1.2
1.0
0.8
F2 [ppm]

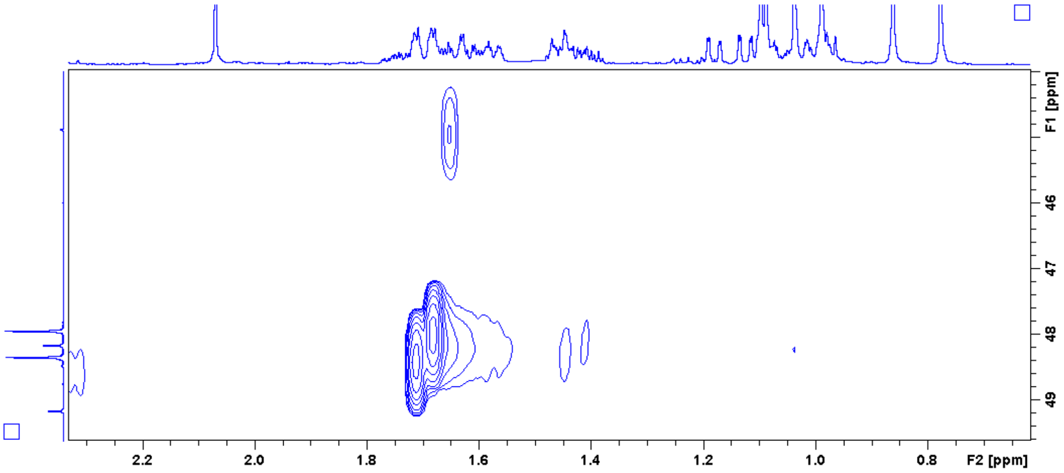
F1 [ppm]
46
47
48
49
2.2
2.0
1.8
1.6
1.4
1.2
1.0
0.8
F2 [ppm]

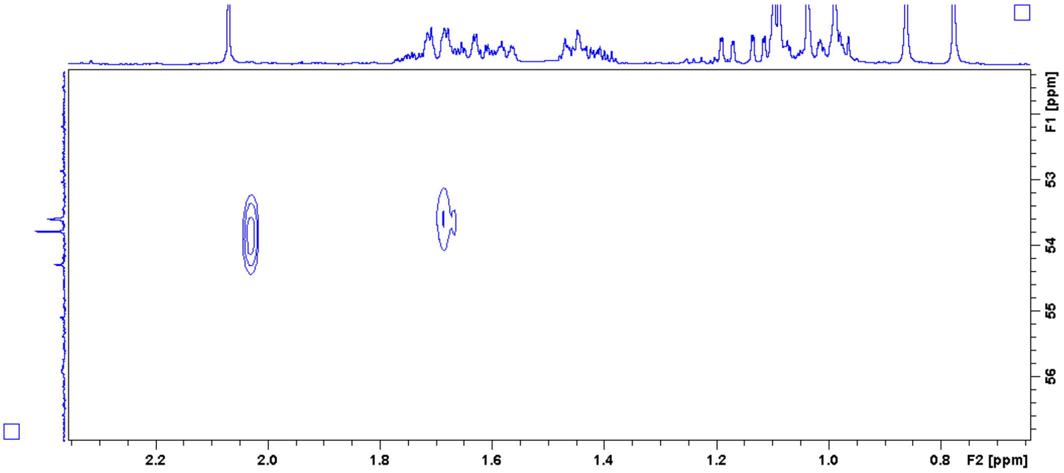


**Figure S 19.** $^{1}H$-$^{13}C$-HMBC ($^{1}H$ 500 MHz; $^{13}C$ 125 MHz) spectrum of EODr in $CDCl_3$

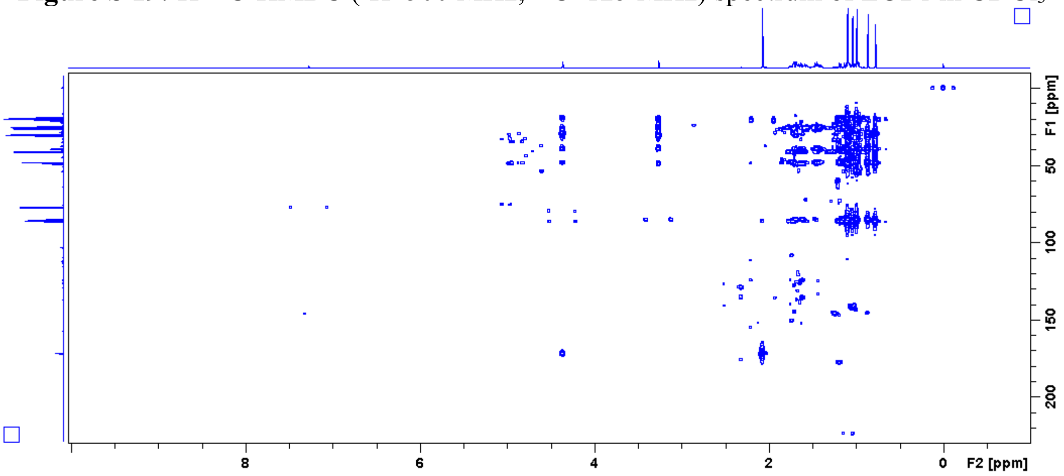


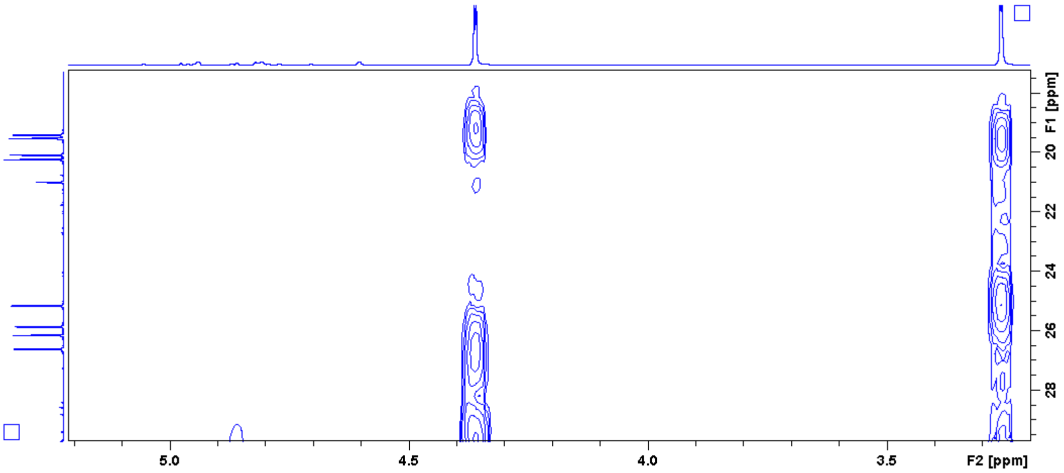

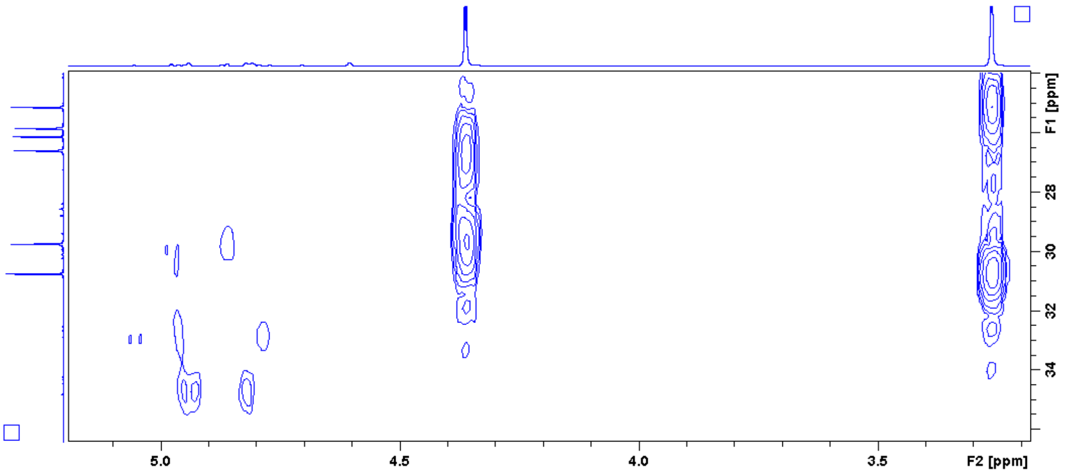

F1 [ppm]
28
30
32
34
5.0
4.5
4.0
3.5
F2 [ppm]


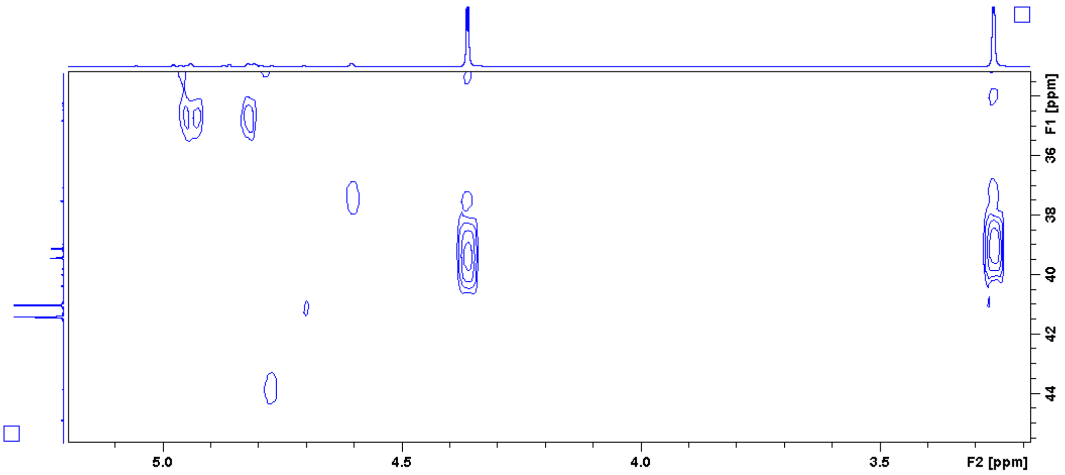

F1 [ppm]
36
38
40
42
44
5.0
4.5
4.0
3.5
F2 [ppm]


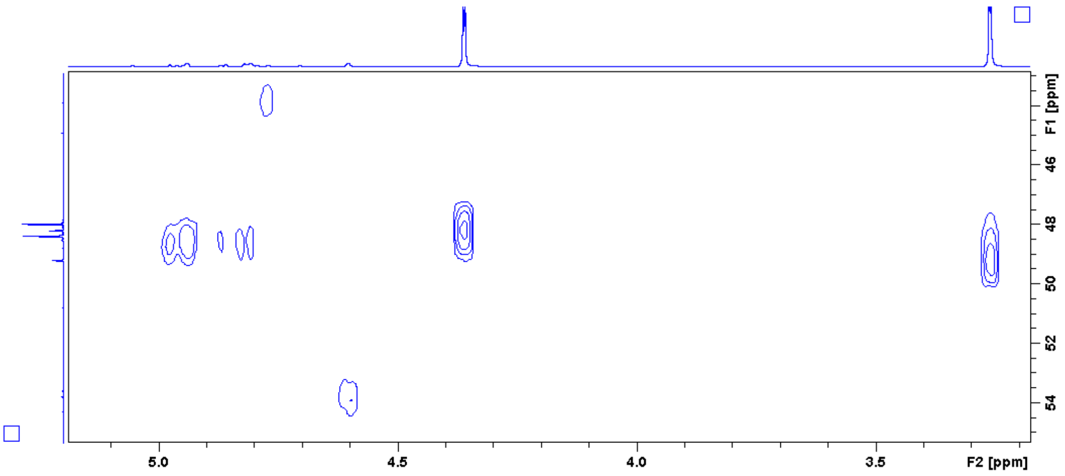

F1 [ppm]
46
48
50
52
54
5.0
4.5
4.0
3.5
F2 [ppm]

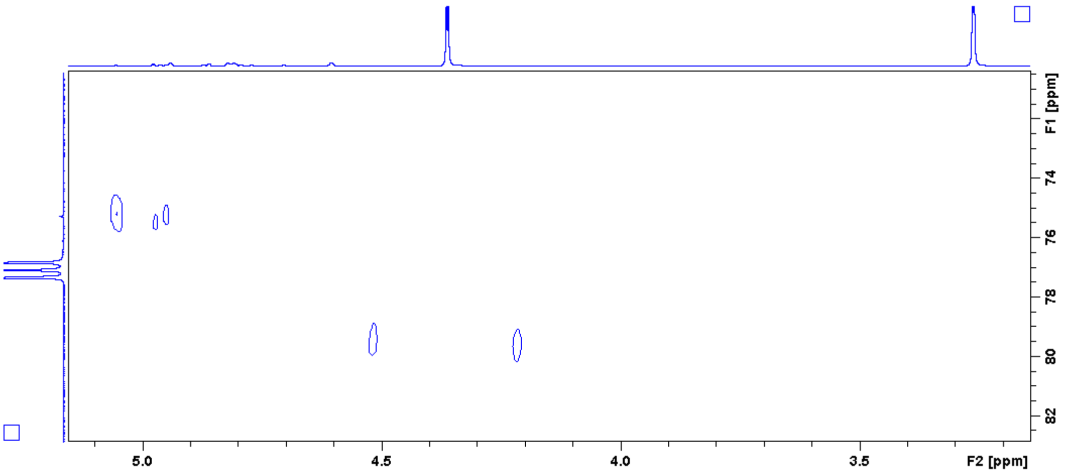
F1 [ppm]
F2 [ppm]

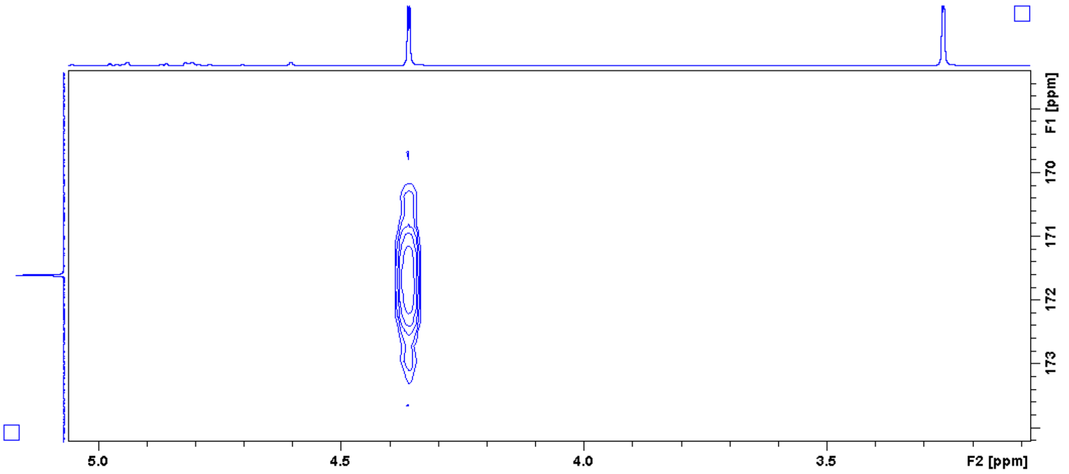
F1 [ppm]
F2 [ppm]

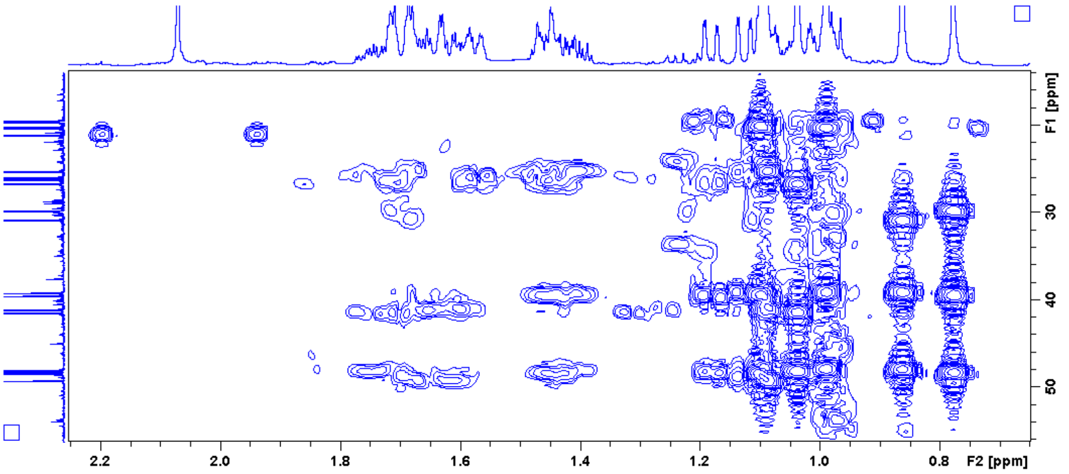
F1 [ppm]
F2 [ppm]

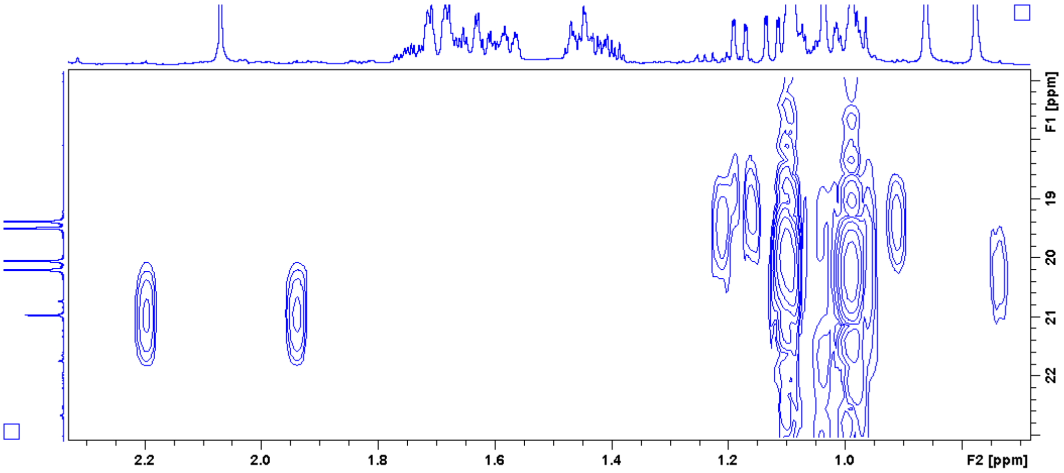

F1 [ppm]
F2 [ppm]


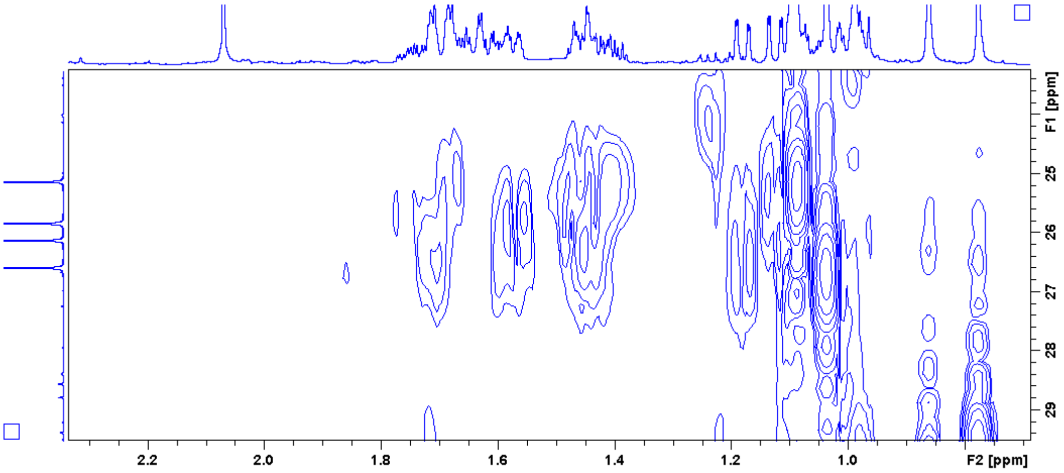

F1 [ppm]
F2 [ppm]


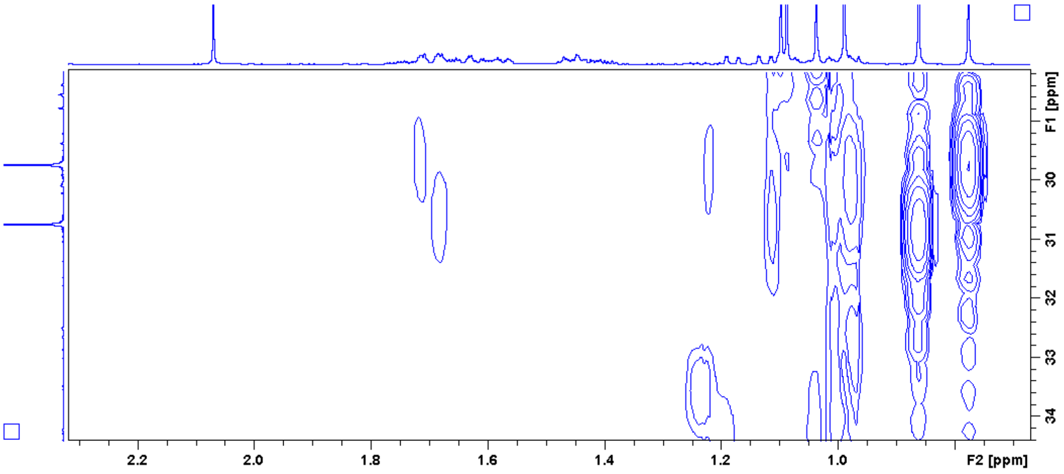

F1 [ppm]
F2 [ppm]

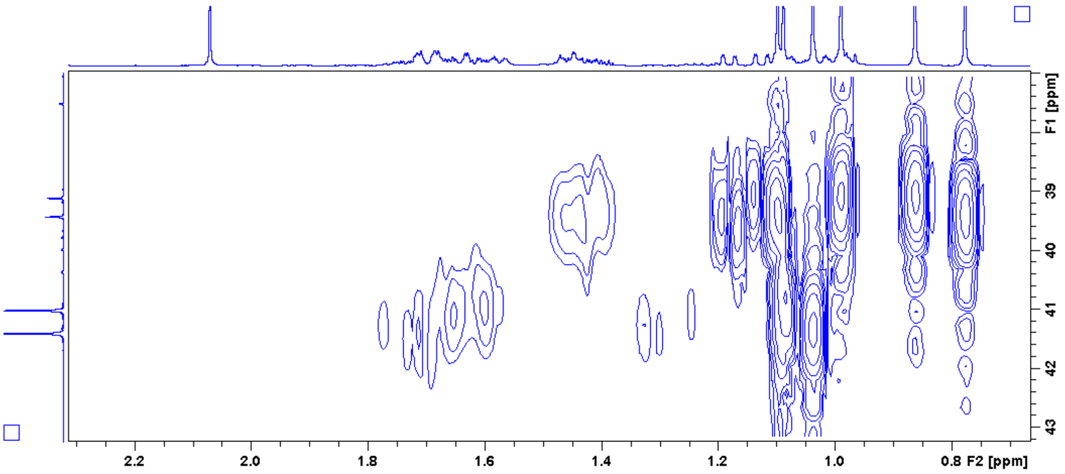
F1 [ppm]
39
40
41
42
43
2.2
2.0
1.8
1.6
1.4
1.2
1.0
0.8 F2 [ppm]

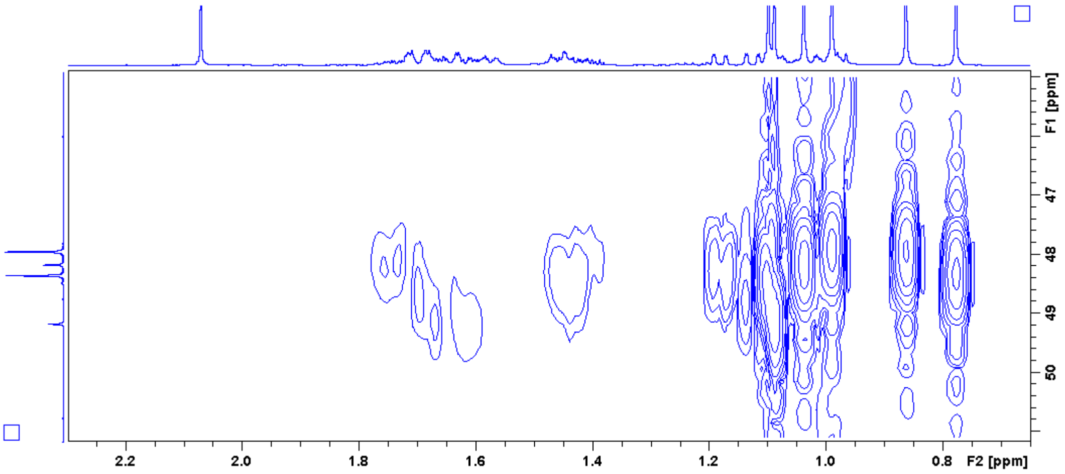
F1 [ppm]
47
48
49
50
2.2
2.0
1.8
1.6
1.4
1.2
1.0
0.8 F2 [ppm]

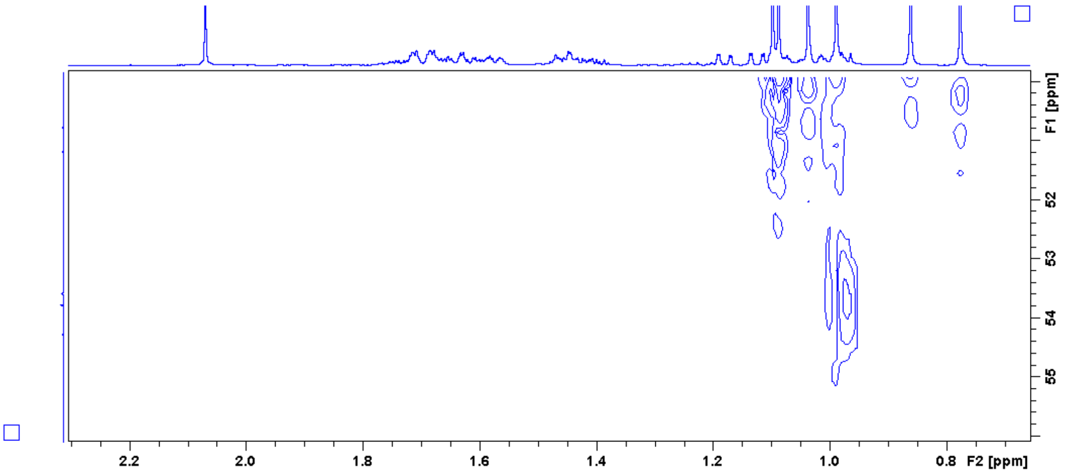
F1 [ppm]
52
53
54
55
2.2
2.0
1.8
1.6
1.4
1.2
1.0
0.8 F2 [ppm]

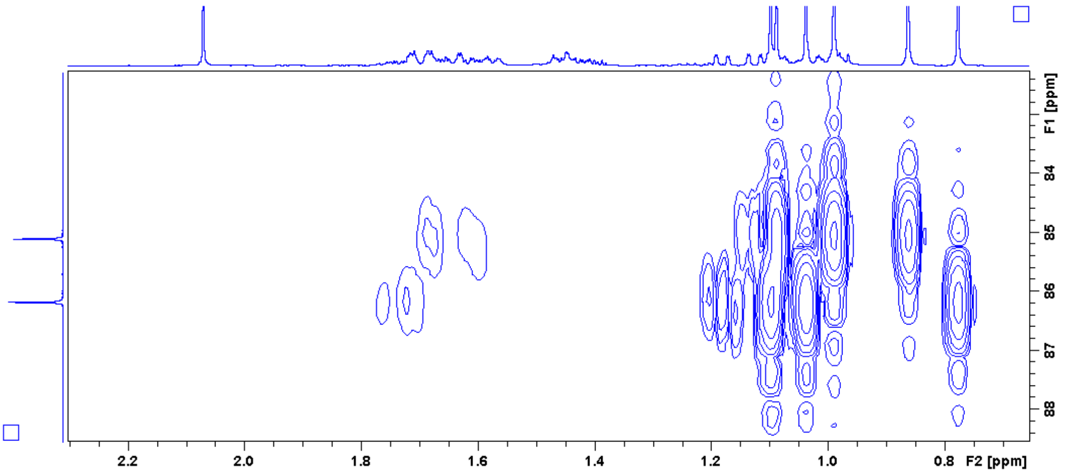
F1 [ppm]
84
85
86
87
88
2.2
2.0
1.8
1.6
1.4
1.2
1.0
0.8
F2 [ppm]

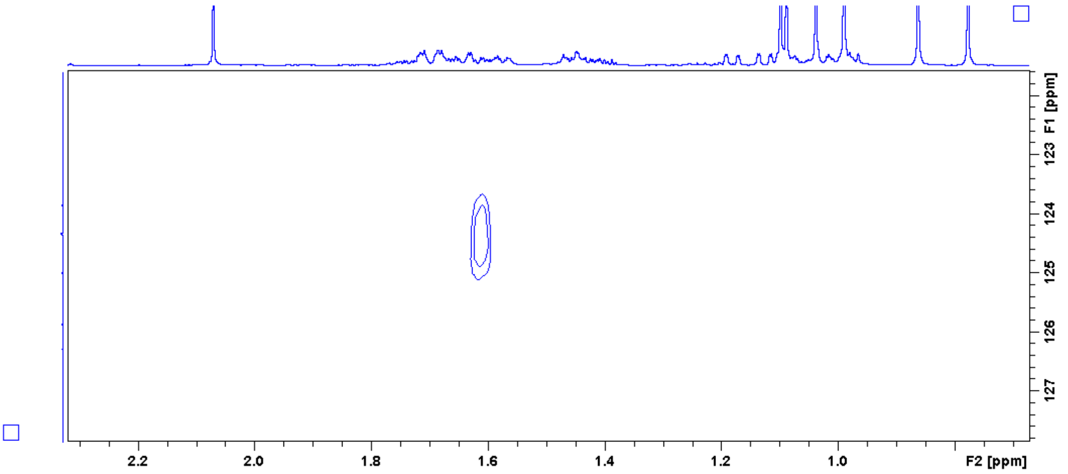
F1 [ppm]
123
124
125
126
127
2.2
2.0
1.8
1.6
1.4
1.2
1.0
F2 [ppm]

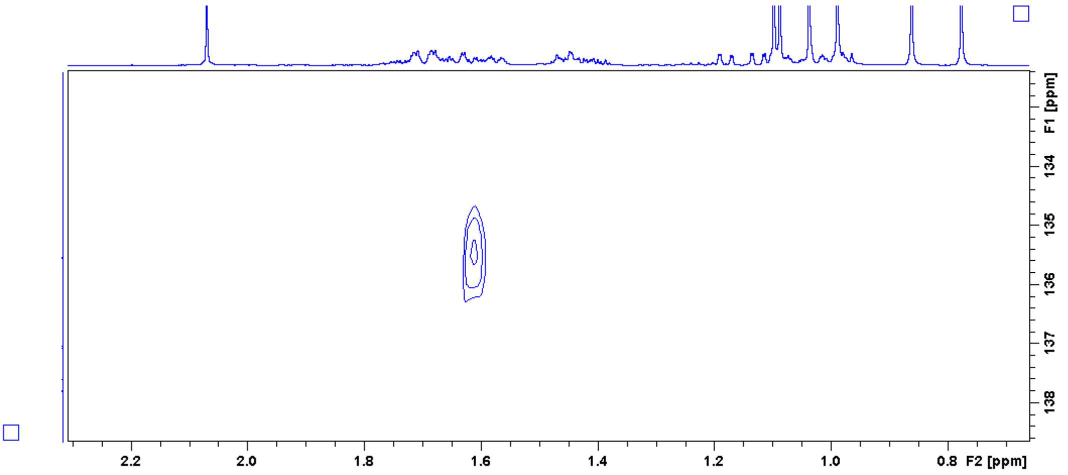
F1 [ppm]
134
135
136
137
138
2.2
2.0
1.8
1.6
1.4
1.2
1.0
0.8
F2 [ppm]

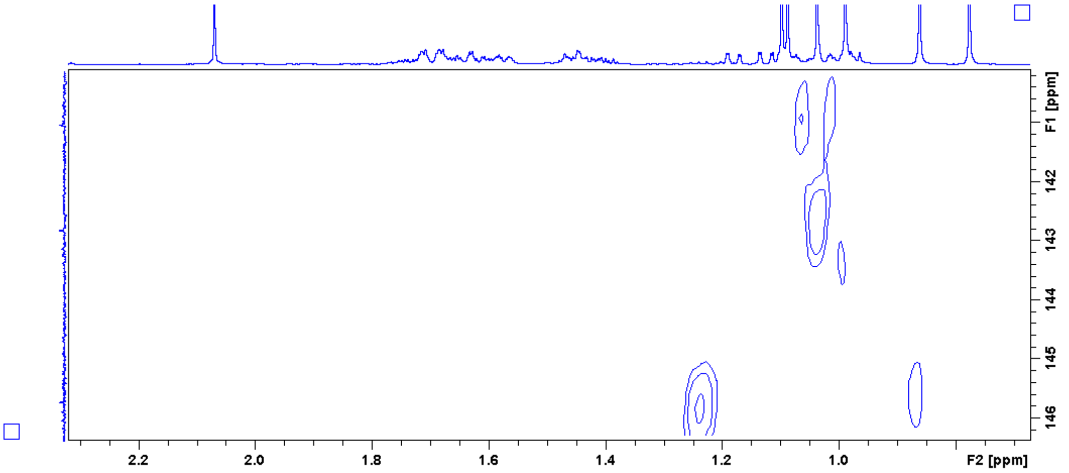
F1 [ppm]
142
143
144
145
146
2.2
2.0
1.8
1.6
1.4
1.2
1.0
F2 [ppm]

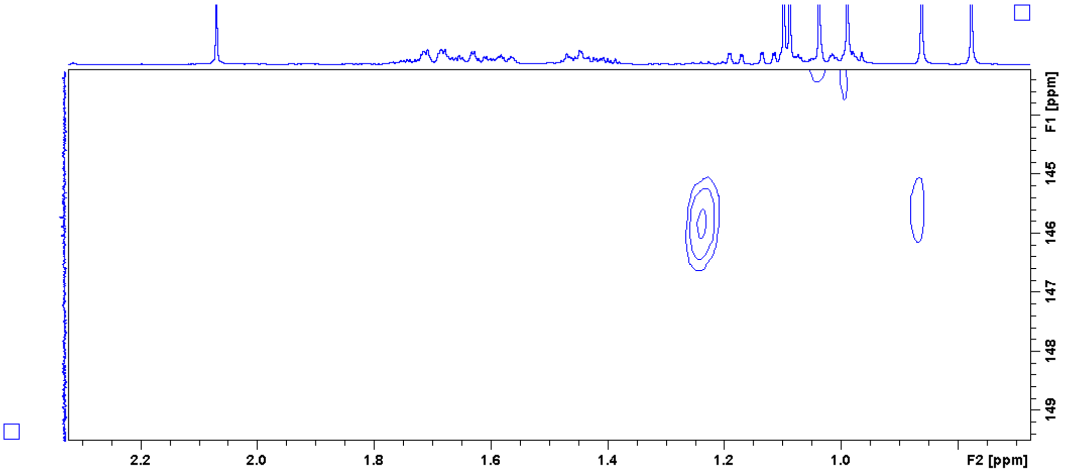
F1 [ppm]
145
146
147
148
149
2.2
2.0
1.8
1.6
1.4
1.2
1.0
F2 [ppm]

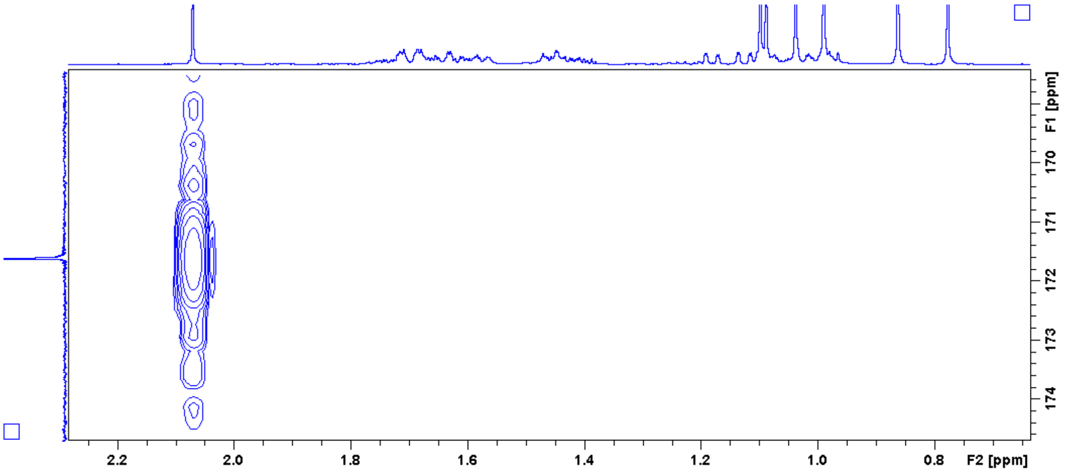
F1 [ppm]
170
171
172
173
174
2.2
2.0
1.8
1.6
1.4
1.2
1.0
0.8
F2 [ppm]

**Figure S 20.**1H NMR (500 MHz) spectrum of fenchol in $CDCl_3$

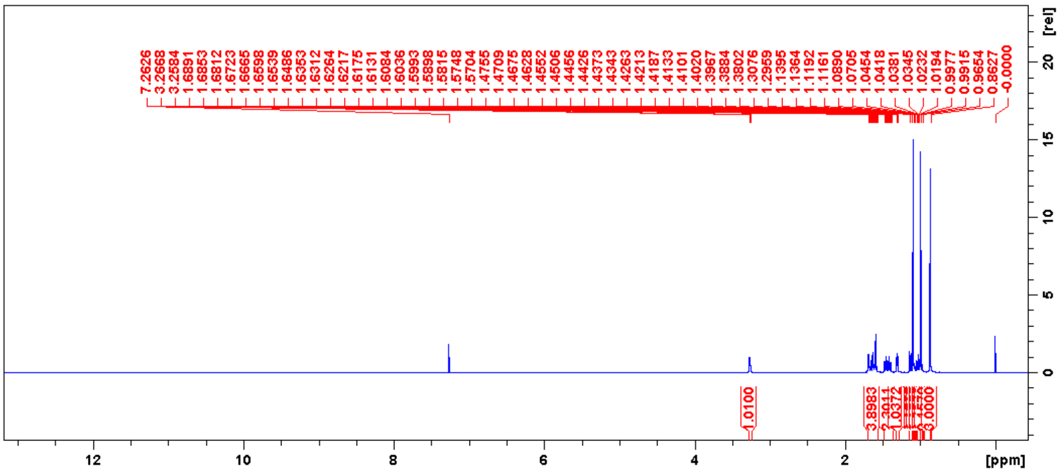

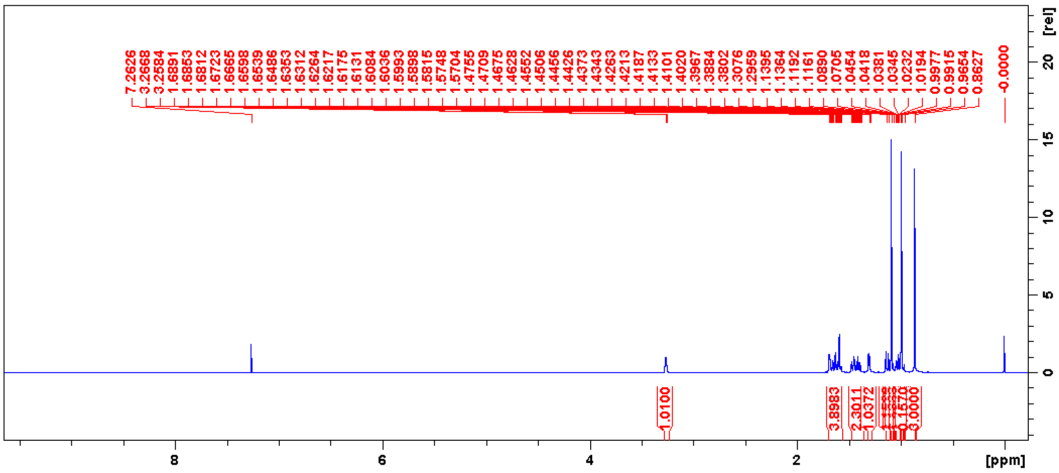

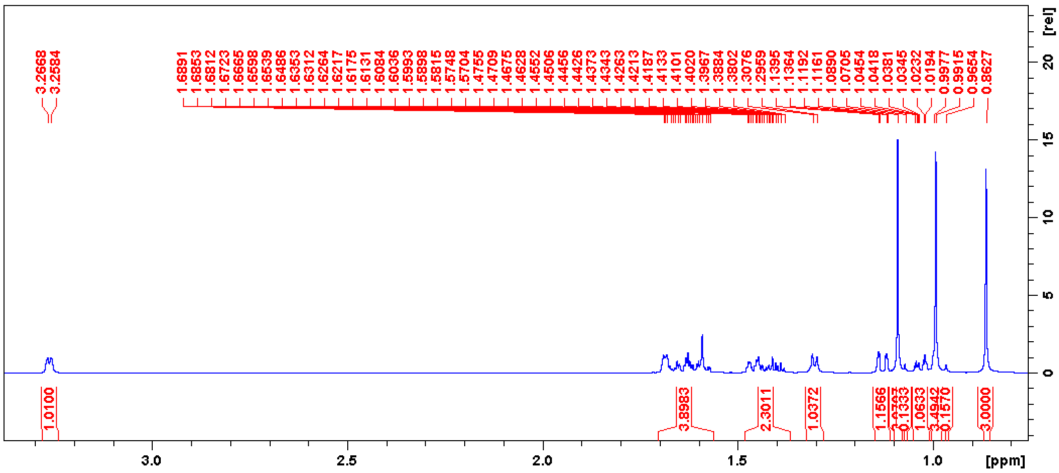

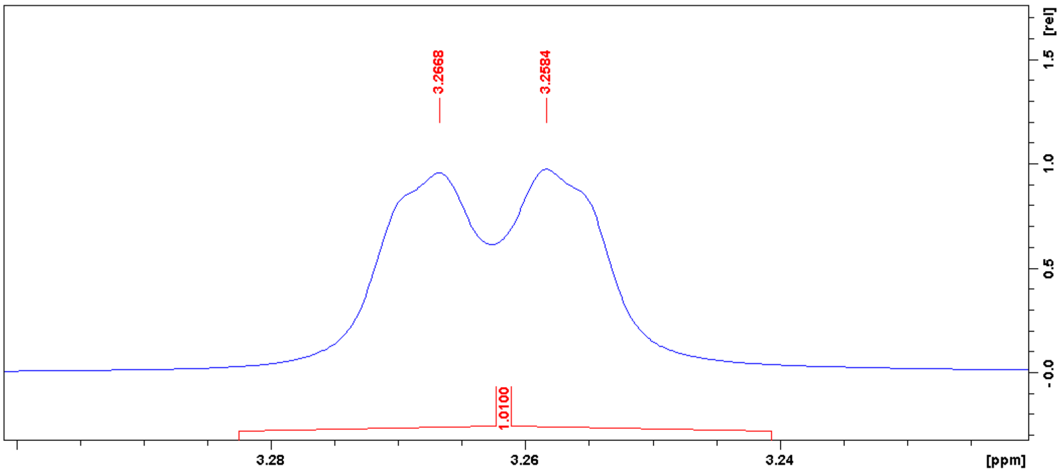

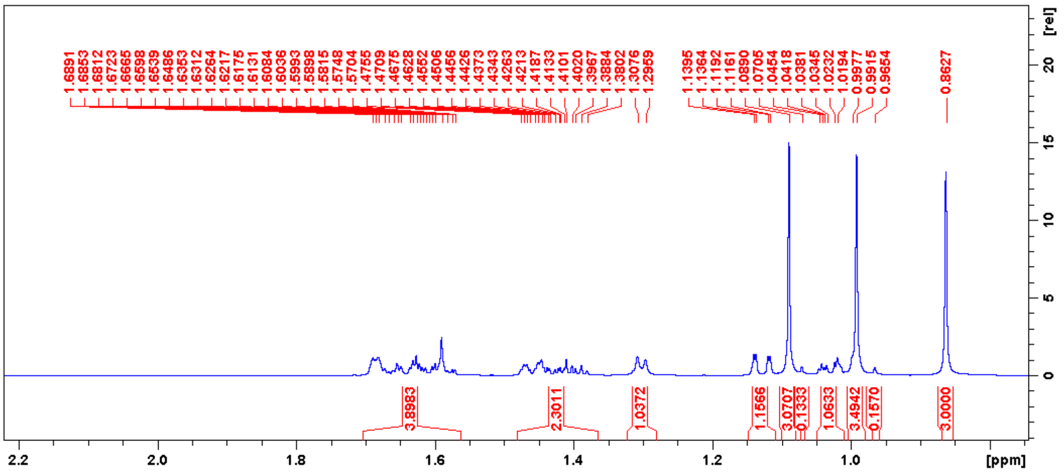

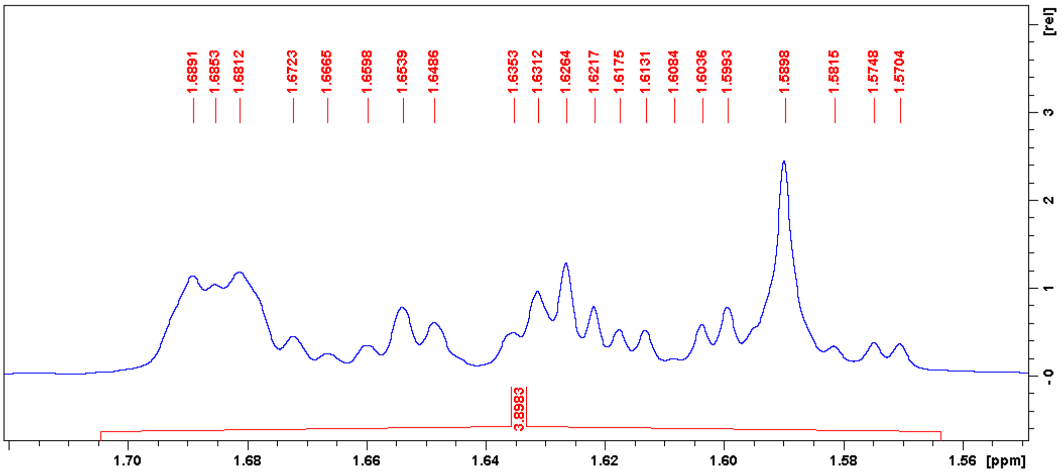

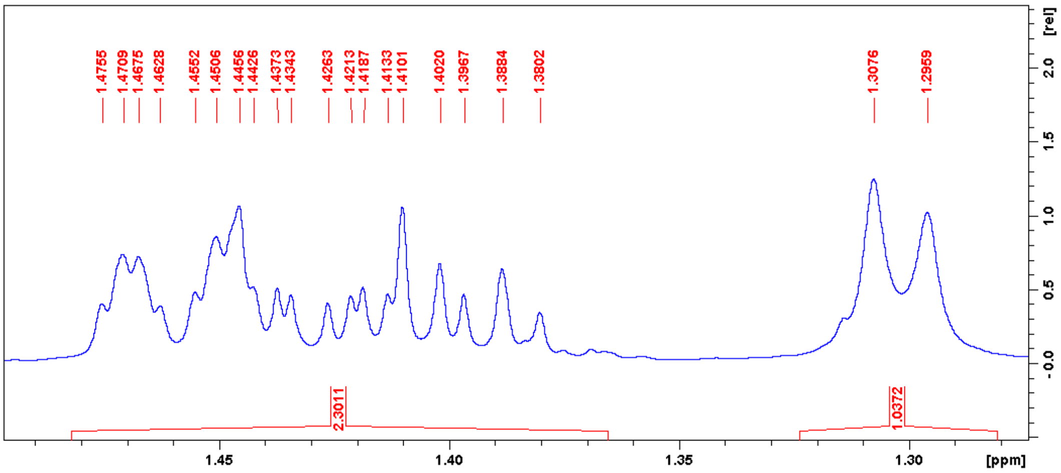
1.4755
1.4709
1.4675
1.4628
1.4552
1.4506
1.4456
1.4426
1.4373
1.4343
1.4263
1.4213
1.4187
1.4133
1.4101
1.4020
1.3967
1.3884
1.3802
1.3076
1.2959
2.3011
1.0372
[rel]
[ppm]

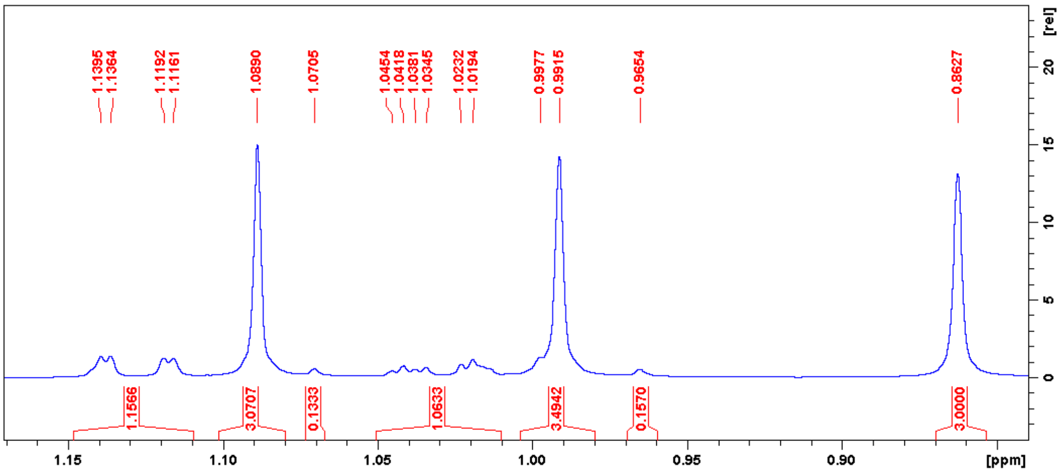
1.1395
1.1364
1.1192
1.1161
1.0890
1.0705
1.0454
1.0418
1.0381
1.0345
1.0232
1.0194
0.9977
0.9915
0.9654
0.8627
1.1566
3.0707
0.1333
1.0633
3.4942
0.1570
3.0000
[rel]
[ppm]

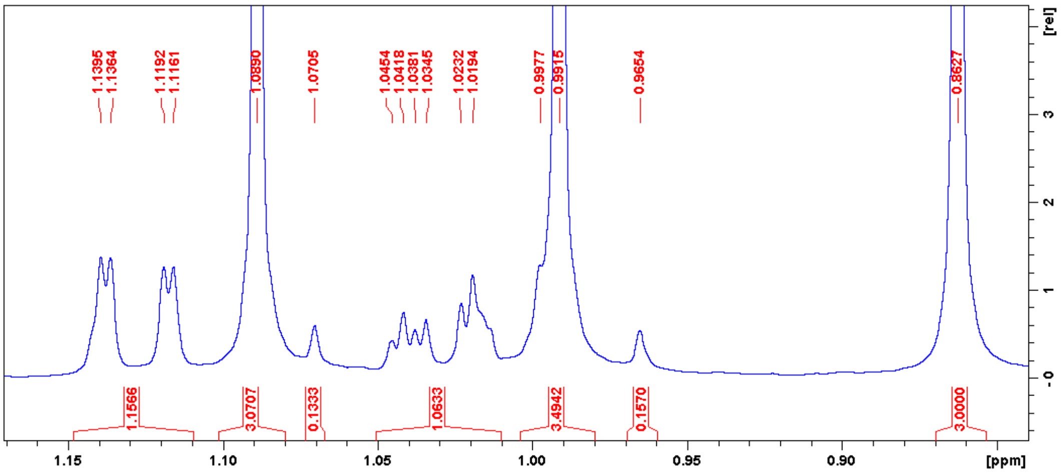
1.1395
1.1364
1.1192
1.1161
1.0890
1.0705
1.0454
1.0418
1.0381
1.0345
1.0232
1.0194
0.9977
0.9915
0.9654
0.8627
1.1566
3.0707
0.1333
1.0633
3.4942
0.1570
3.0000
[rel]
[ppm]

**Figure S 21.**$^{13}$C NMR (125 MHz) spectrum of fenchol in $CDCl_3$

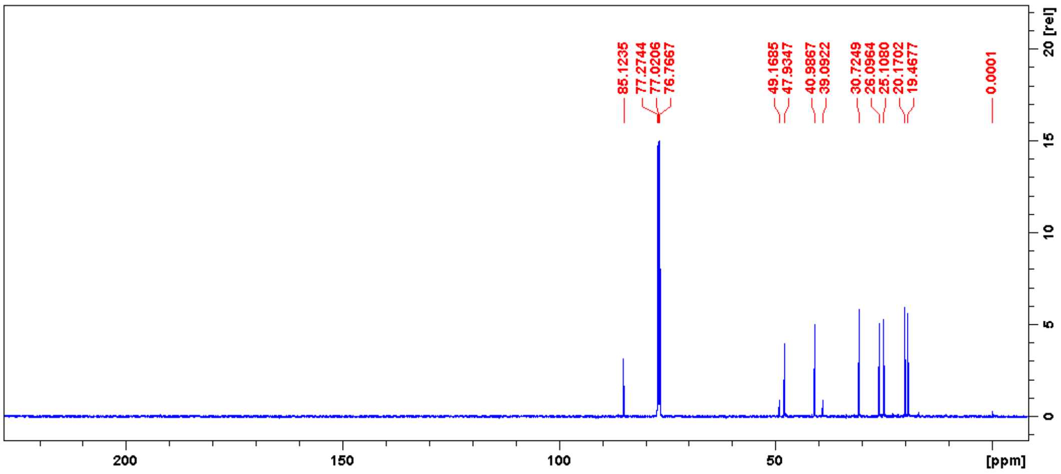


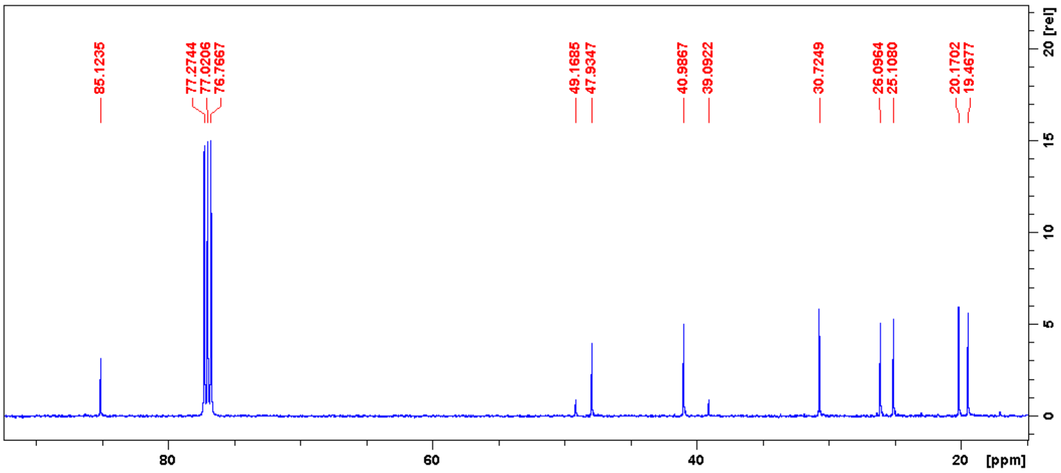


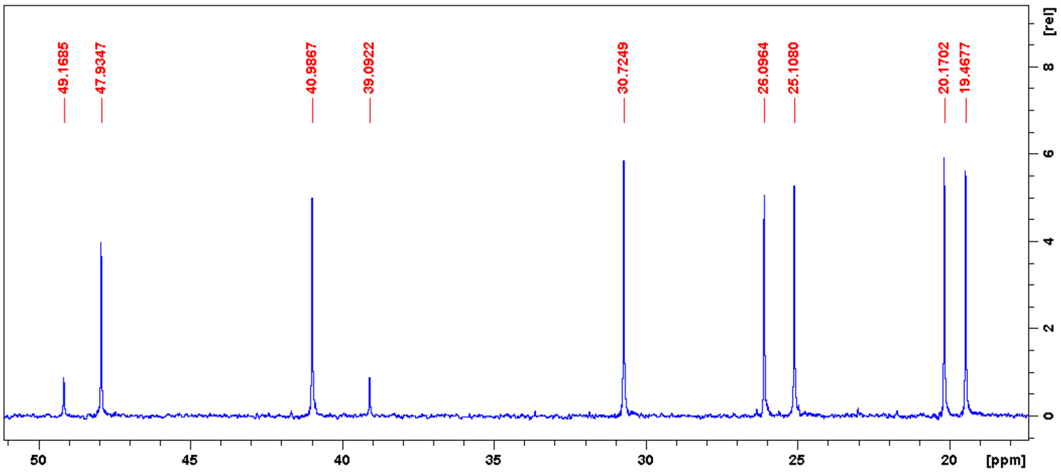

**Figure S 22.**$^{13}$C NMR and DEPT 135 (125 MHz) spectrum of fenchol in $CDCl_3$

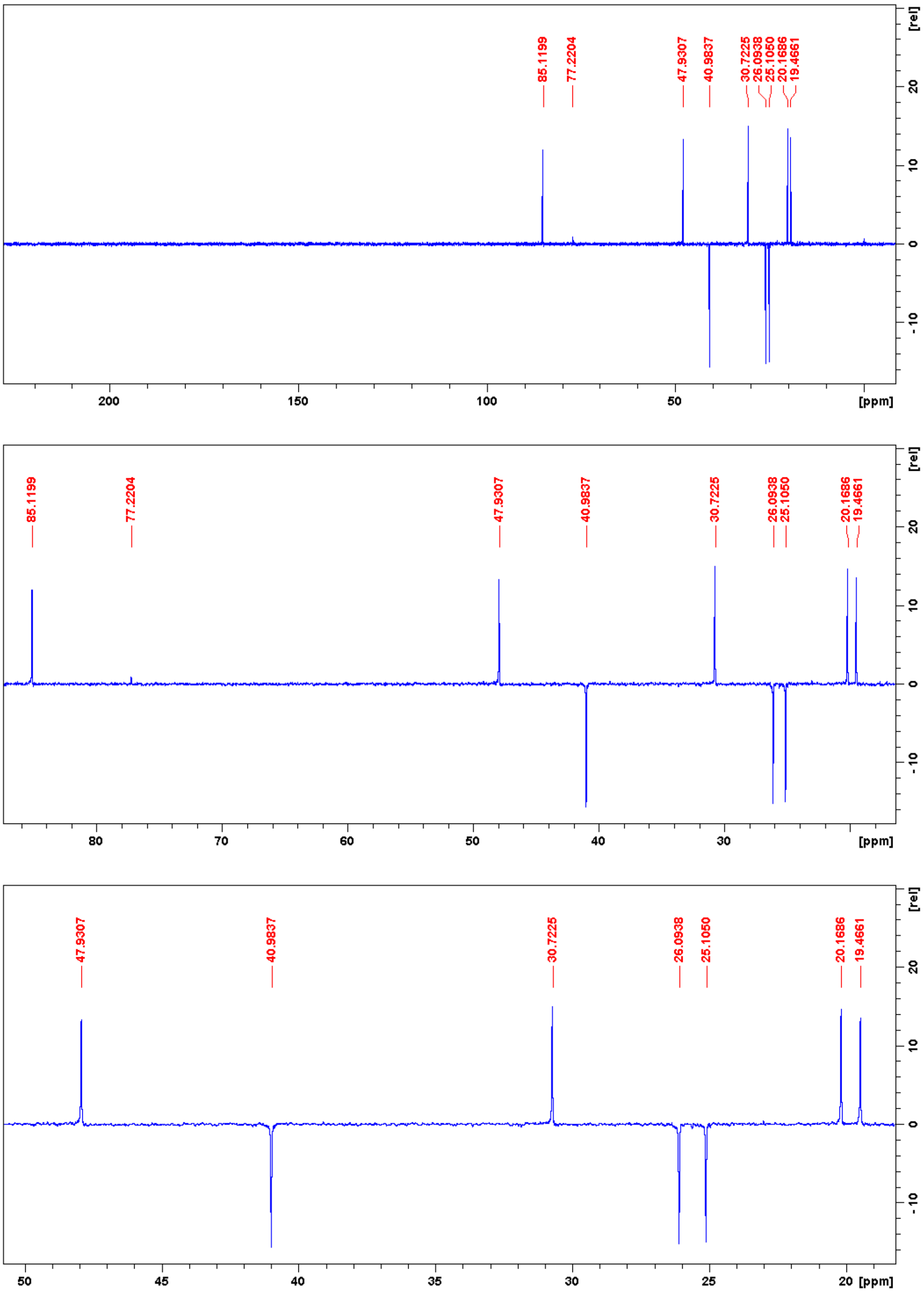

**Figure S 23.** $^{1}$H-$^{1}$H-COSY (500 MHz) spectrum of fenchol in $CDCl_3$

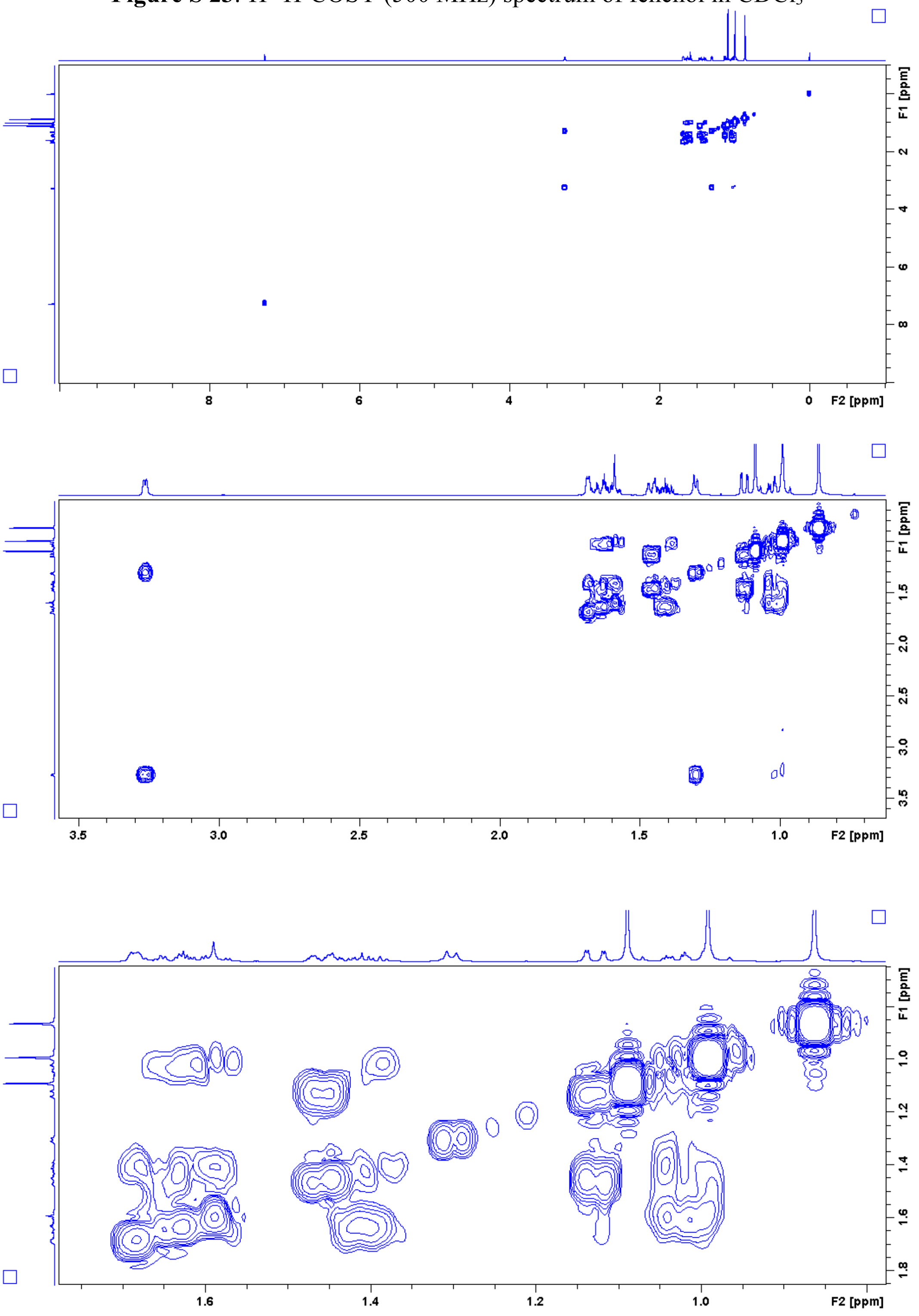

**Figure S 24.** $^{1}H$-$^{13}C$-HSQC ($^{1}H$ 500 MHz; $^{13}C$ 125 MHz) spectrum of fenchol in $CDCl_3$

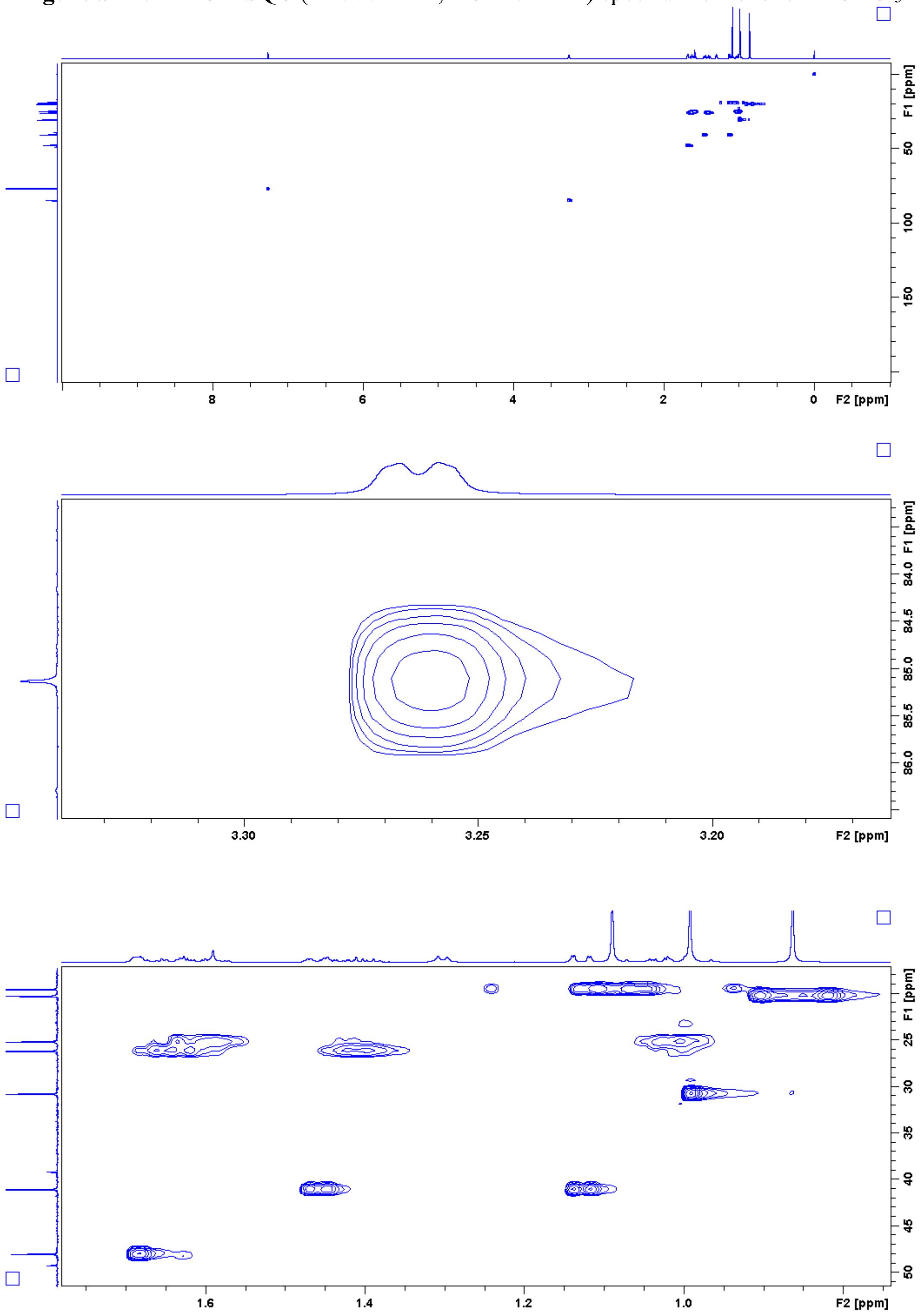

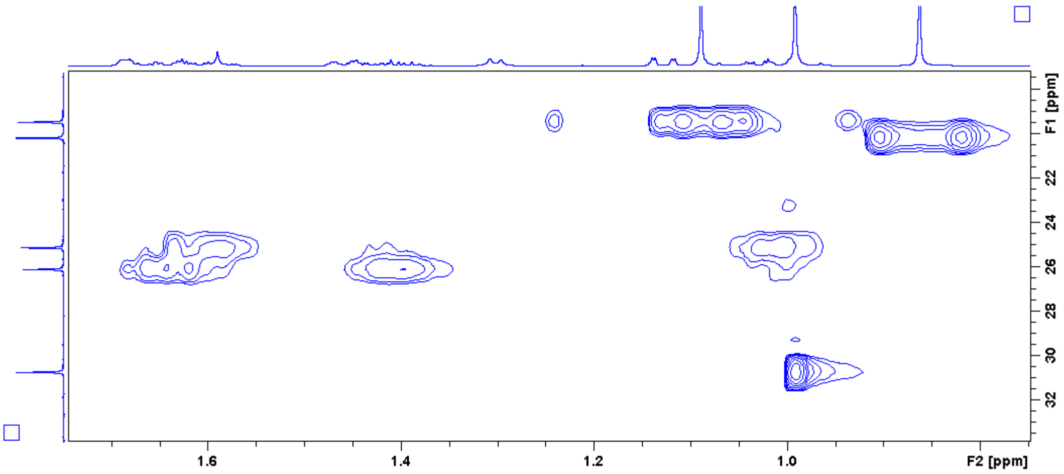

F1 [ppm]
22
24
26
28
30
32
1.6
1.4
1.2
1.0
F2 [ppm]


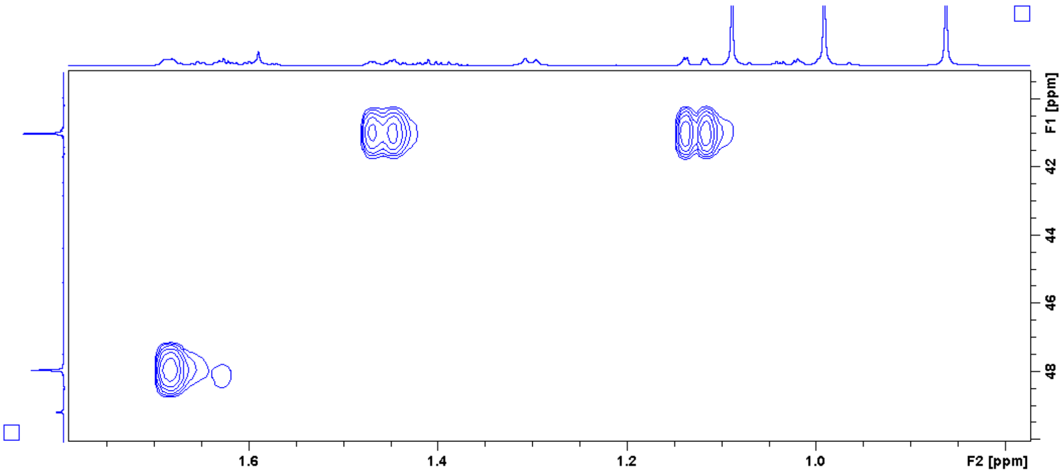

F1 [ppm]
42
44
46
48
1.6
1.4
1.2
1.0
F2 [ppm]

**Figure S 25.**$^{1}$H-$^{13}$C-HMBC ($^{1}$H 500 MHz; $^{13}$C 125 MHz) spectrum of fenchol in $CDCl_3$

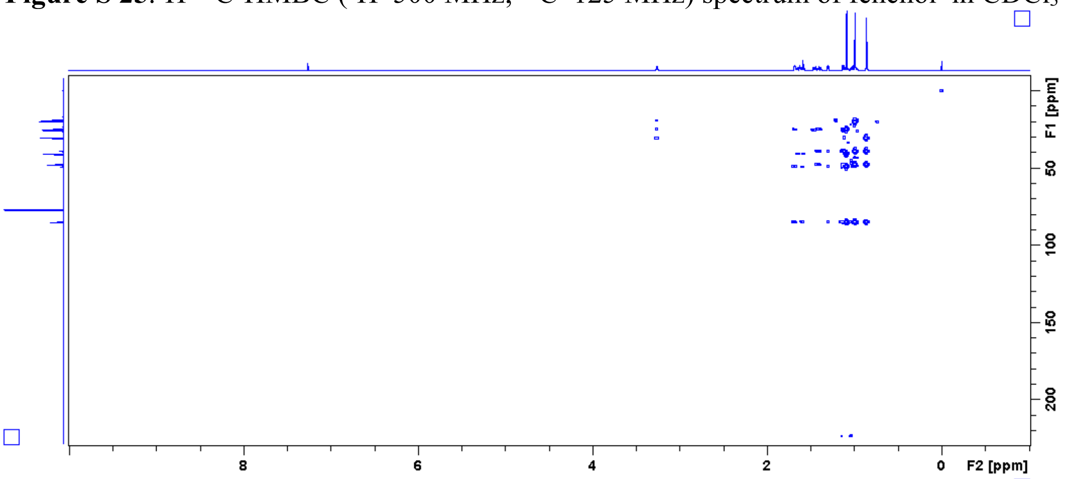


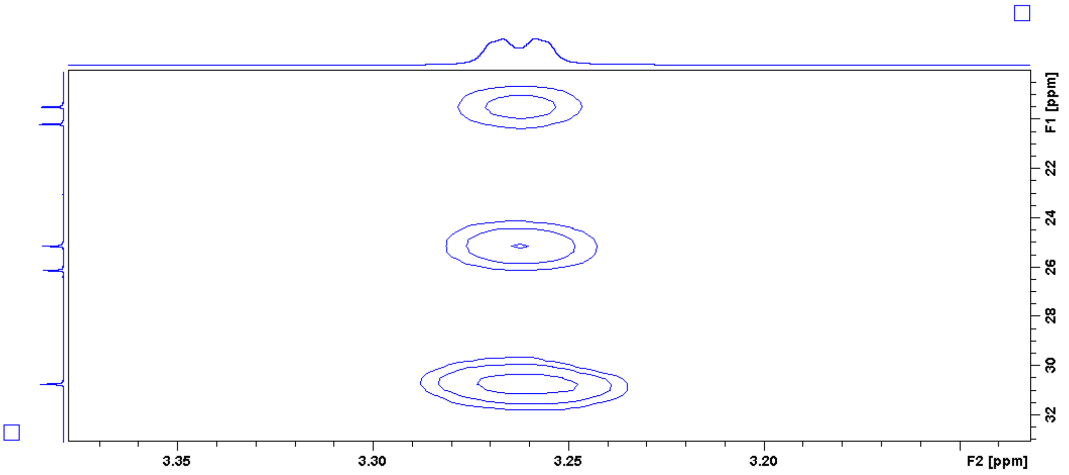


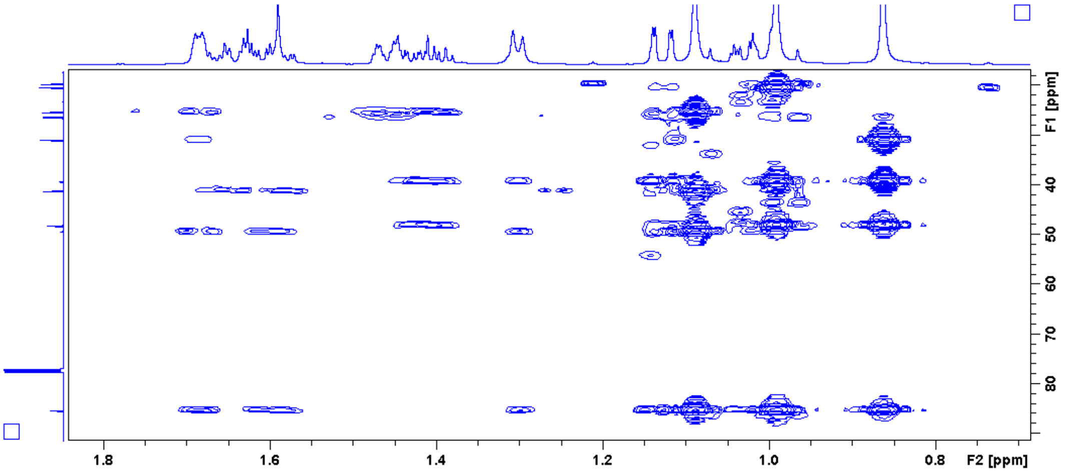

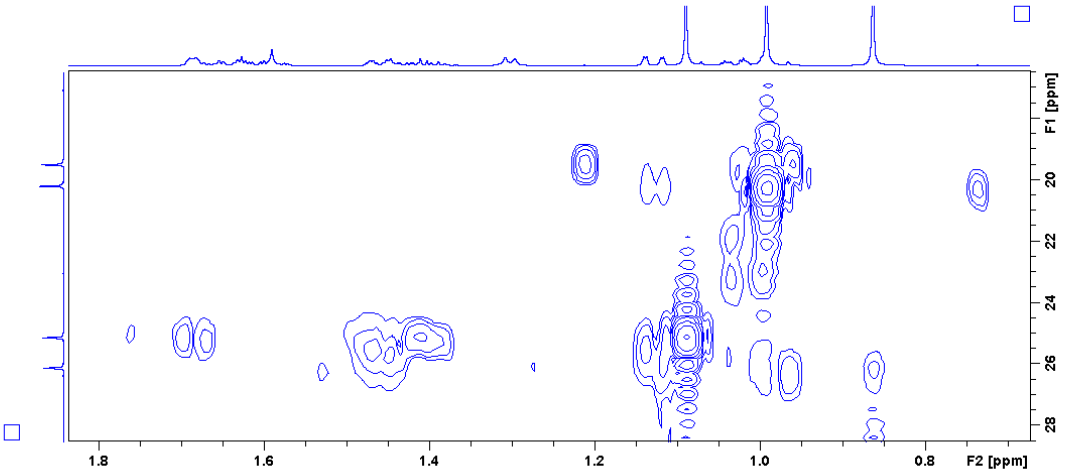
F1 [ppm]
F2 [ppm]

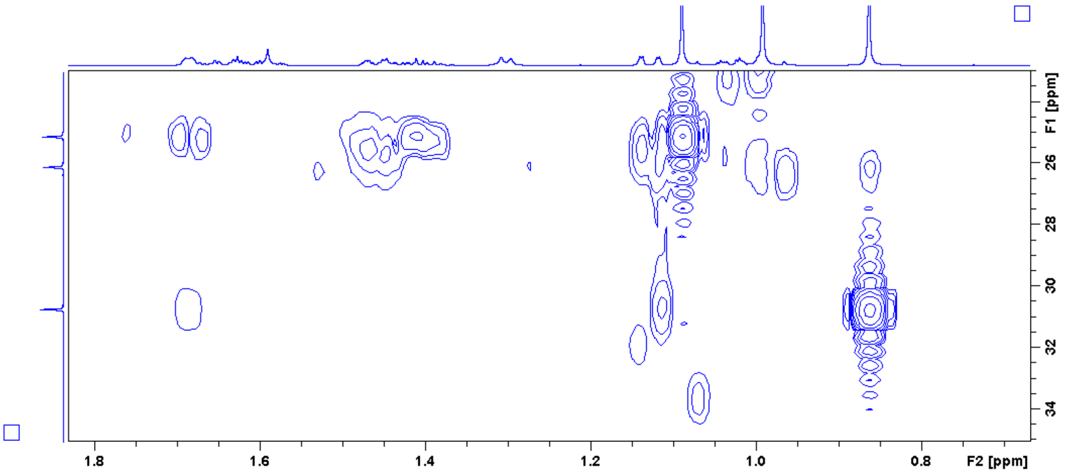
F1 [ppm]
F2 [ppm]

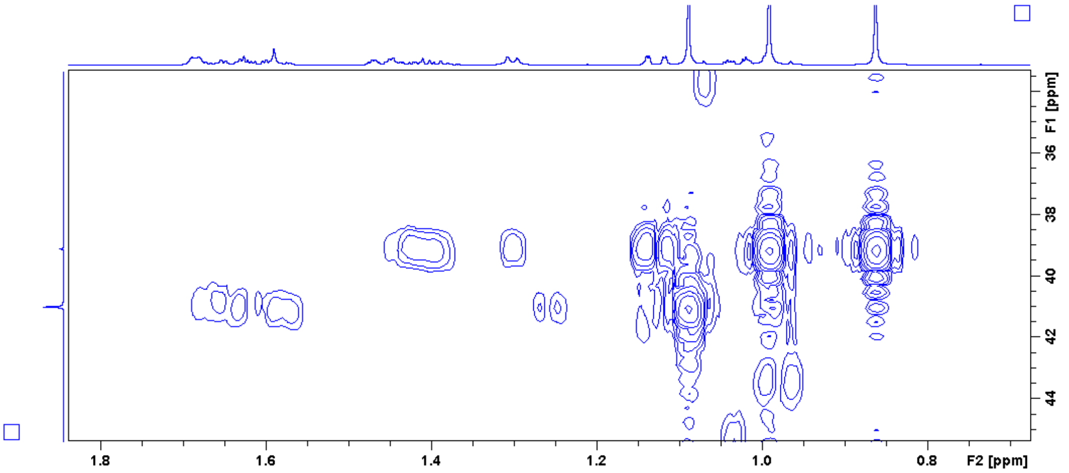
F1 [ppm]
F2 [ppm]

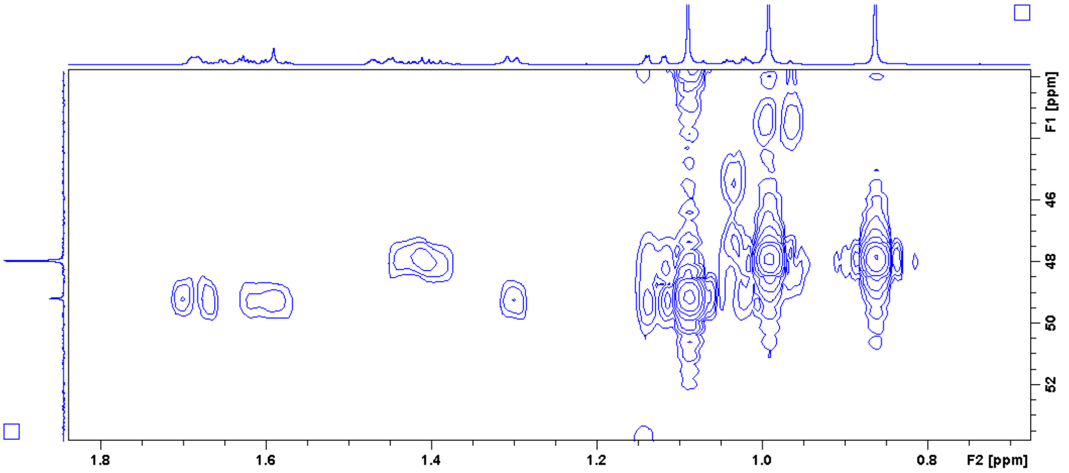
F1 [ppm]
46
48
50
52
1.8
1.6
1.4
1.2
1.0
0.8
F2 [ppm]

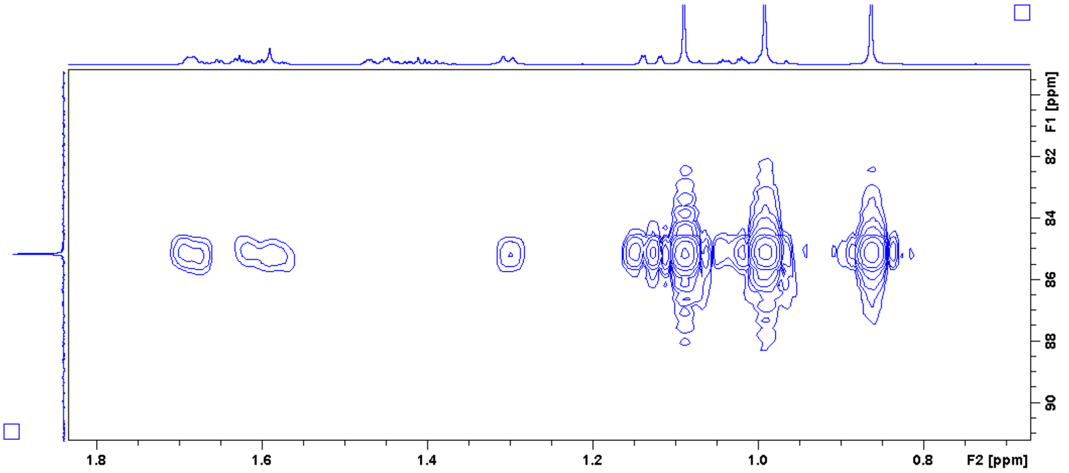
F1 [ppm]
82
84
86
88
90
1.8
1.6
1.4
1.2
1.0
0.8
F2 [ppm]

**Figure S 26**. $^{1}H$ NMR (500 MHz) spectrum of fenchyl acetate in $CDCl_3$

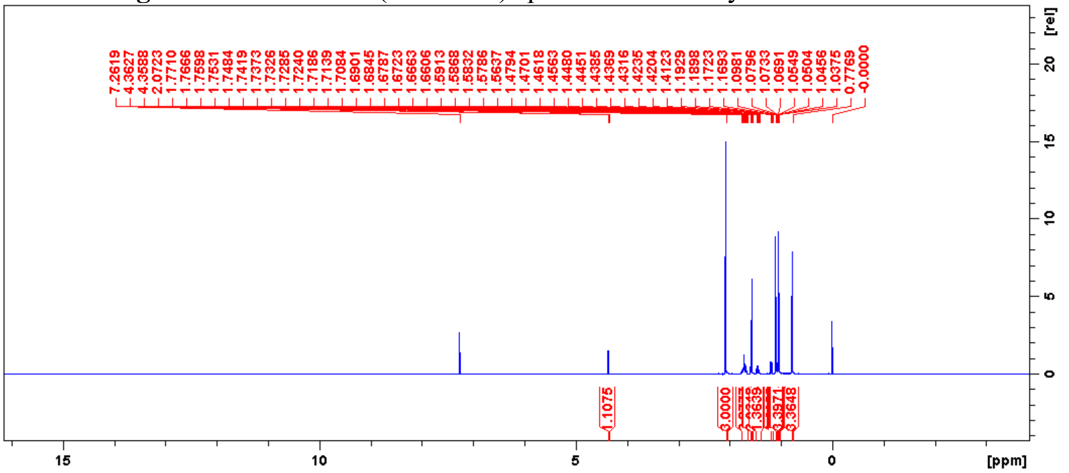


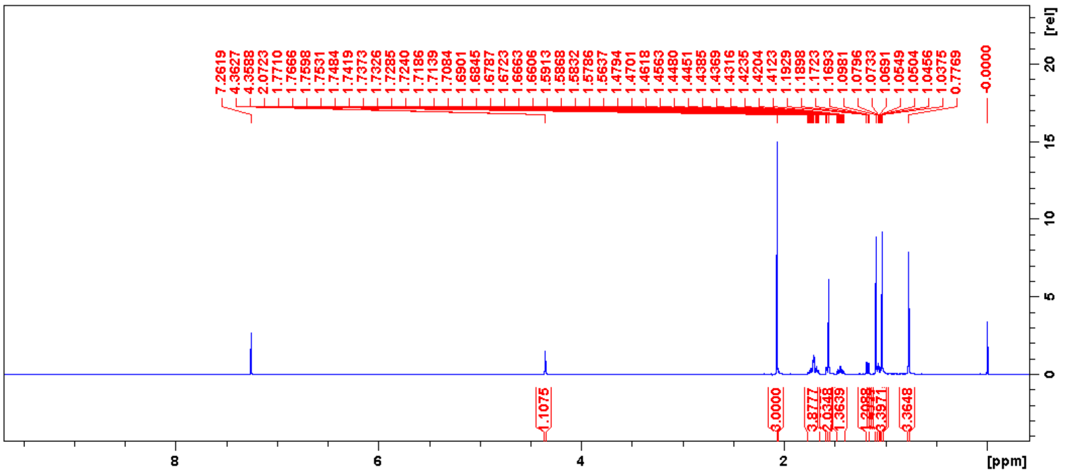


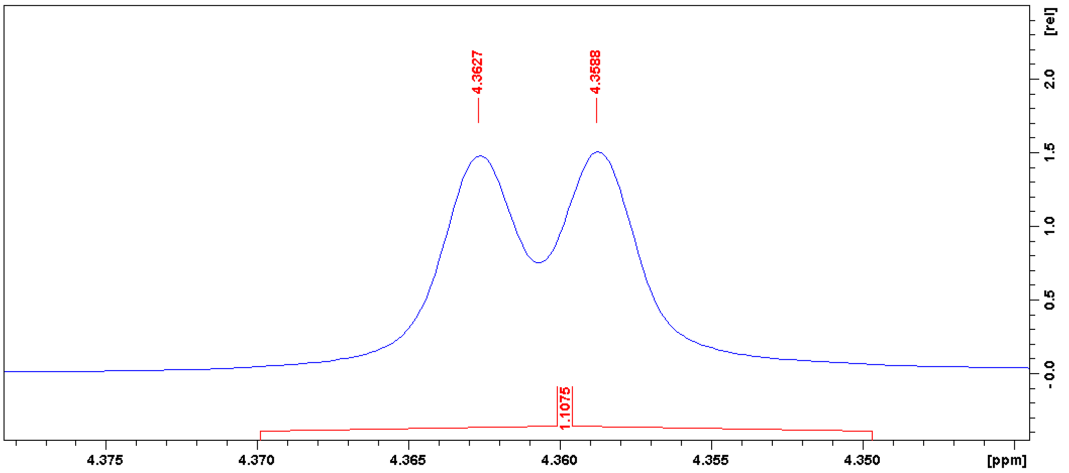

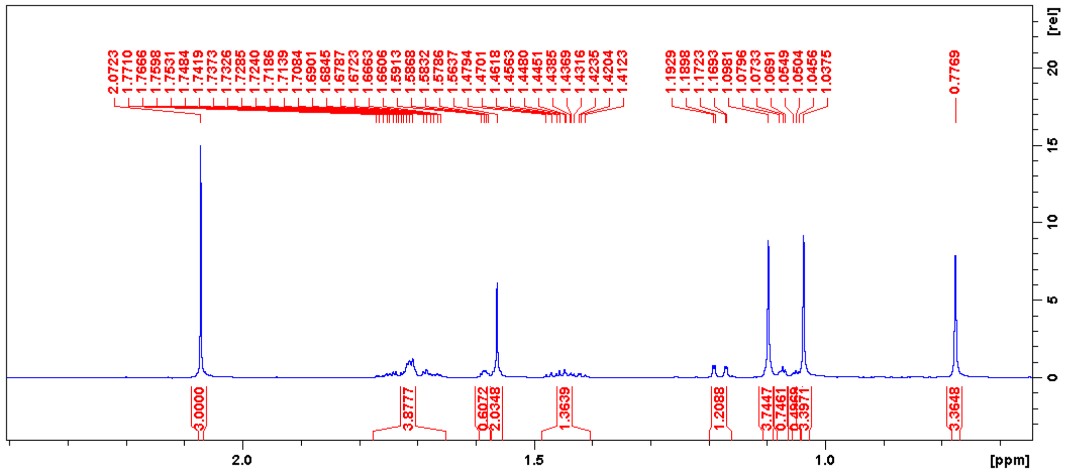

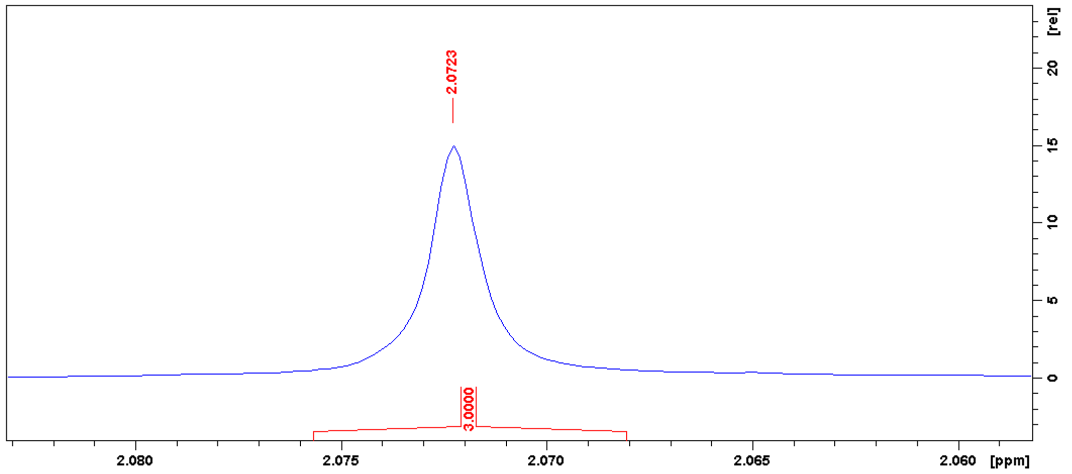

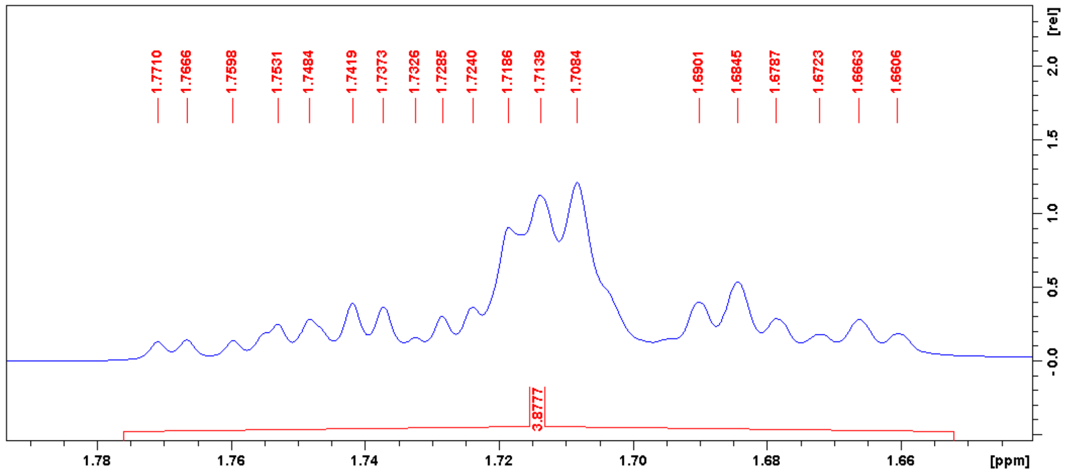

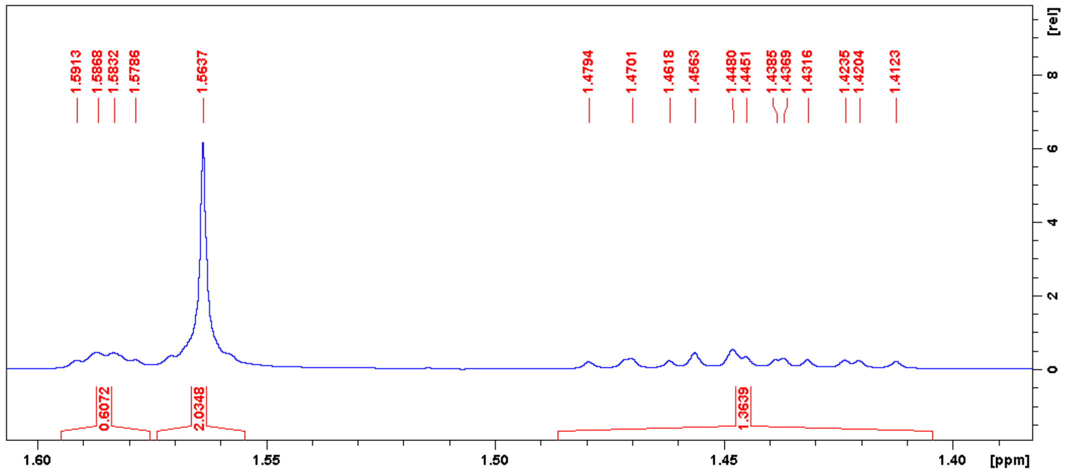

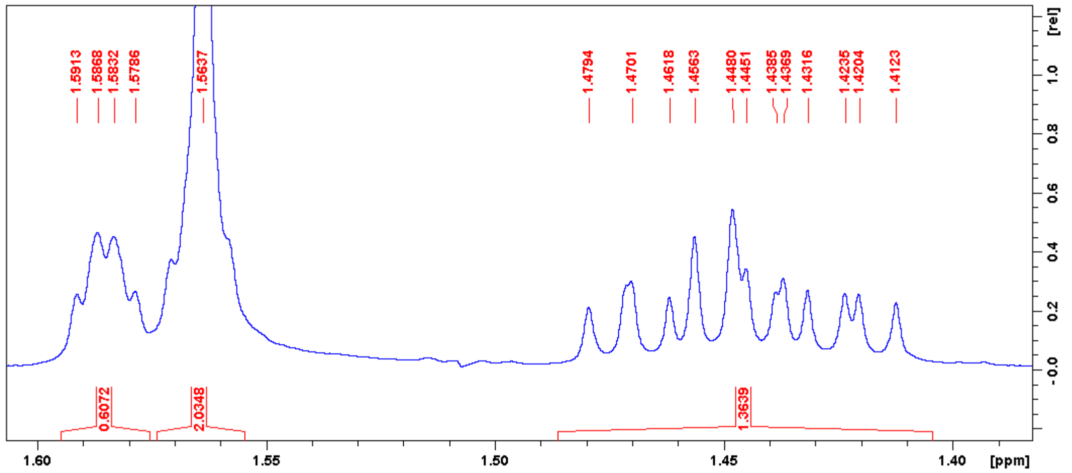

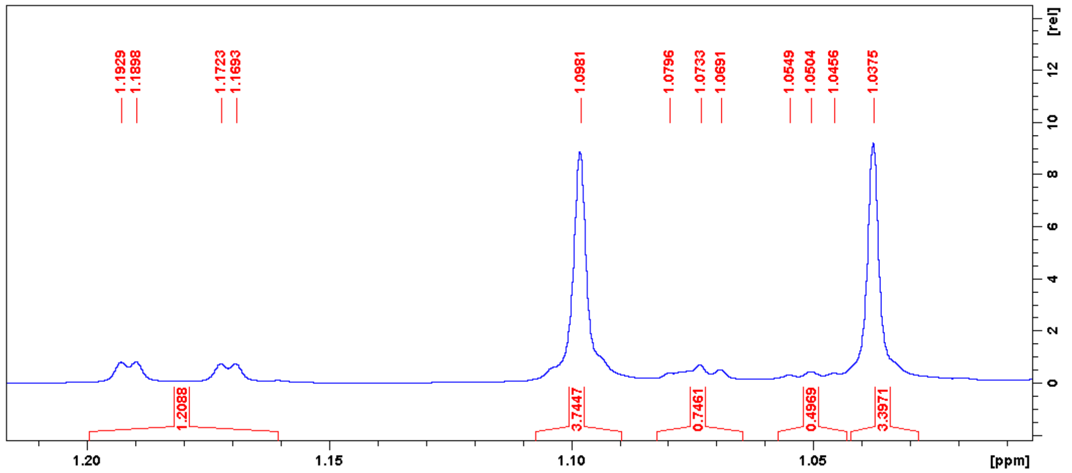

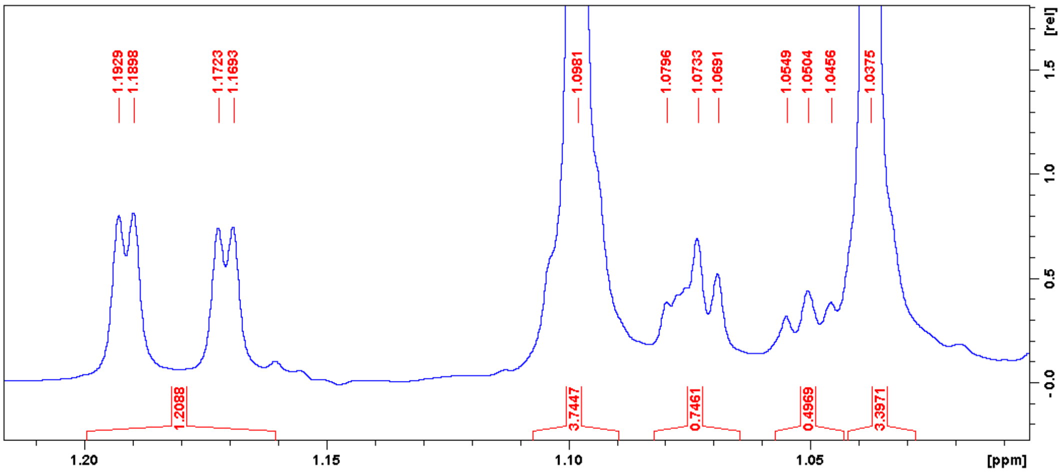
1.1929
1.1898
1.1723
1.1693
1.0981
1.0796
1.0733
1.0691
1.0549
1.0504
1.0456
1.0375
1.2088
3.7447
0.7461
0.4969
3.3971
[ppm]
[rel]

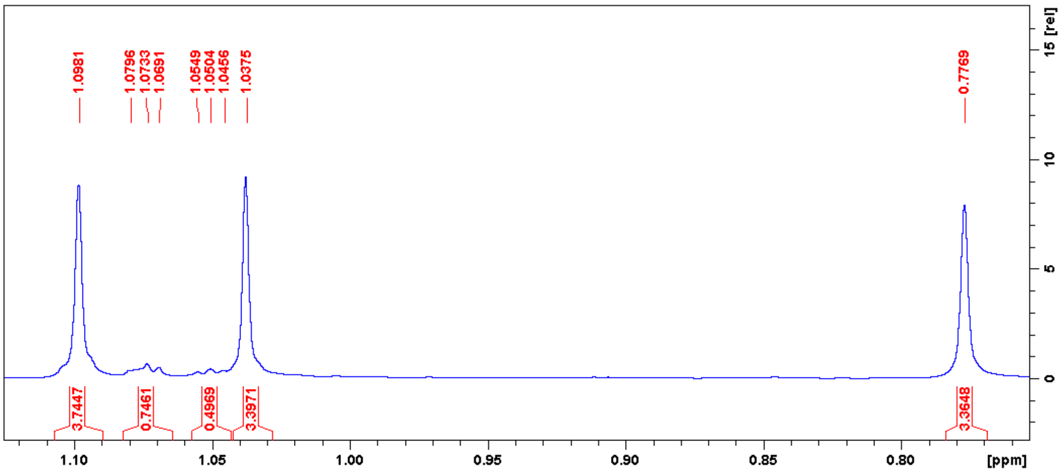
1.0981
1.0796
1.0733
1.0691
1.0549
1.0504
1.0456
1.0375
0.7769
3.7447
0.7461
0.4969
3.3971
3.3648
[ppm]
[rel]

**Figure S 27**. $^{13}C$ NMR (125 MHz) spectrum of fenchyl acetate in $CDCl_3$

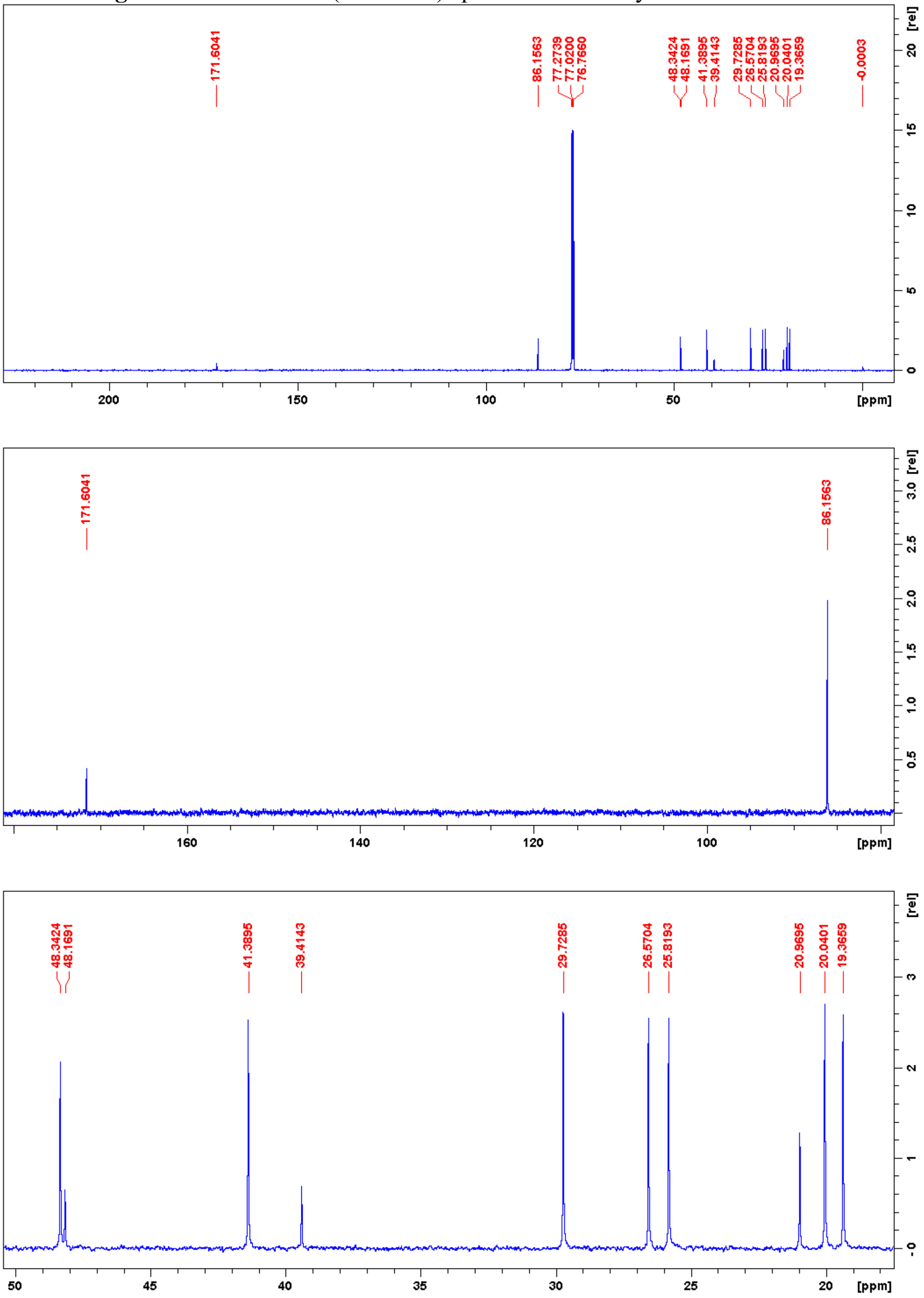

**Figure S 28.** $^{13}C$ NMR and DEPT 135 (125 MHz) spectrum of fenchyl acetate in $CDCl_3$

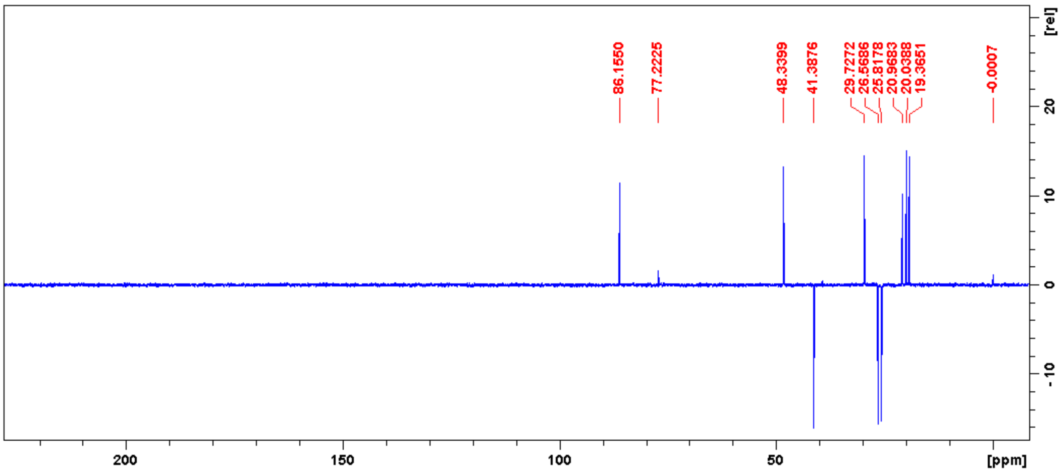


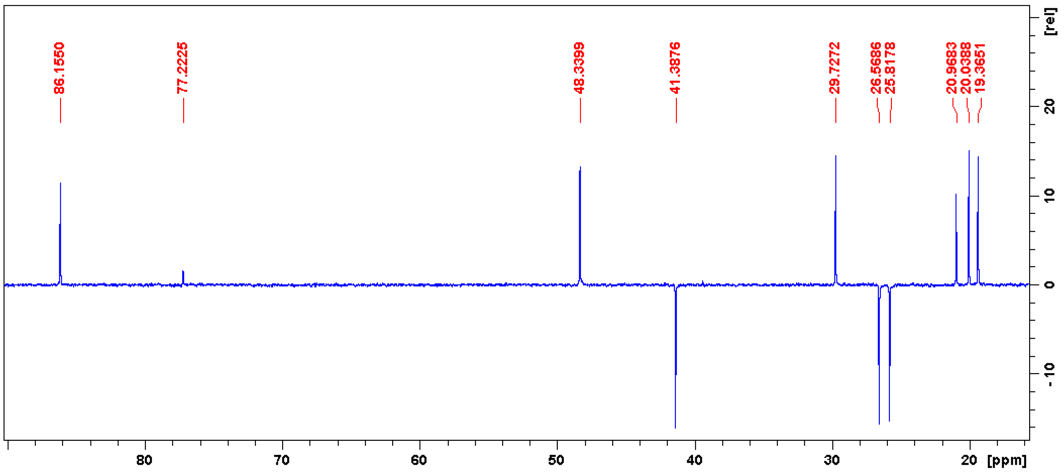


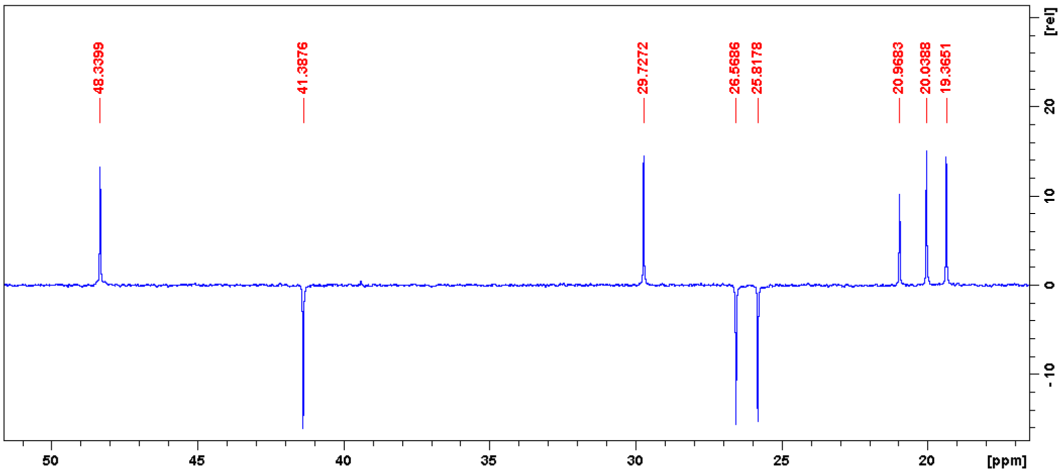

**Figure S 29**. $^{1}$H-$^{1}$H-COSY (500 MHz) spectrum of fenchyl acetate in $CDCl_3$

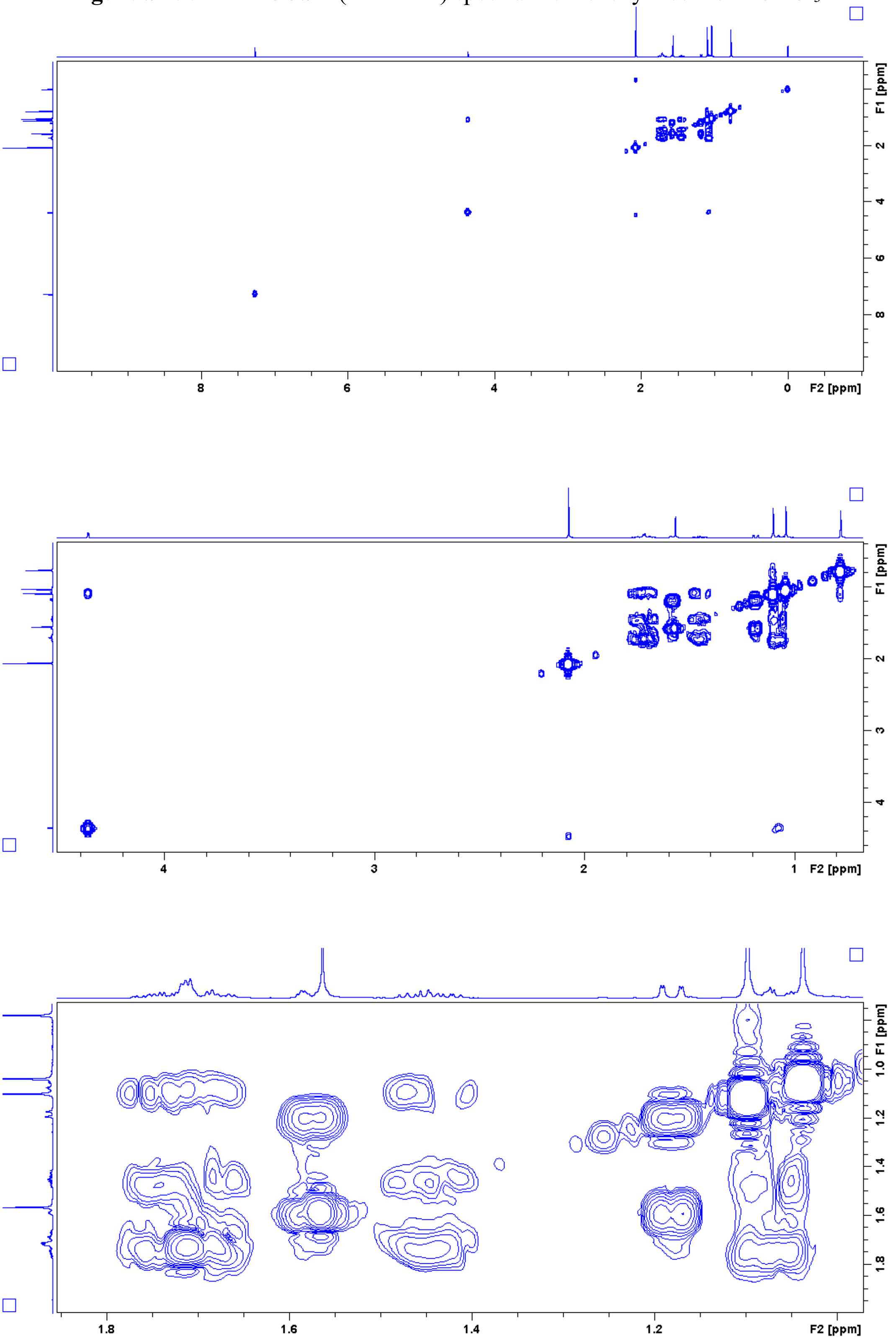

**Figure S 30**. $^{1}H$-$^{13}C$-HSQC ($^{1}H$ 500 MHz; $^{13}C$ 125 MHz) spectrum of fenchyl acetate in $CDCl_3$.

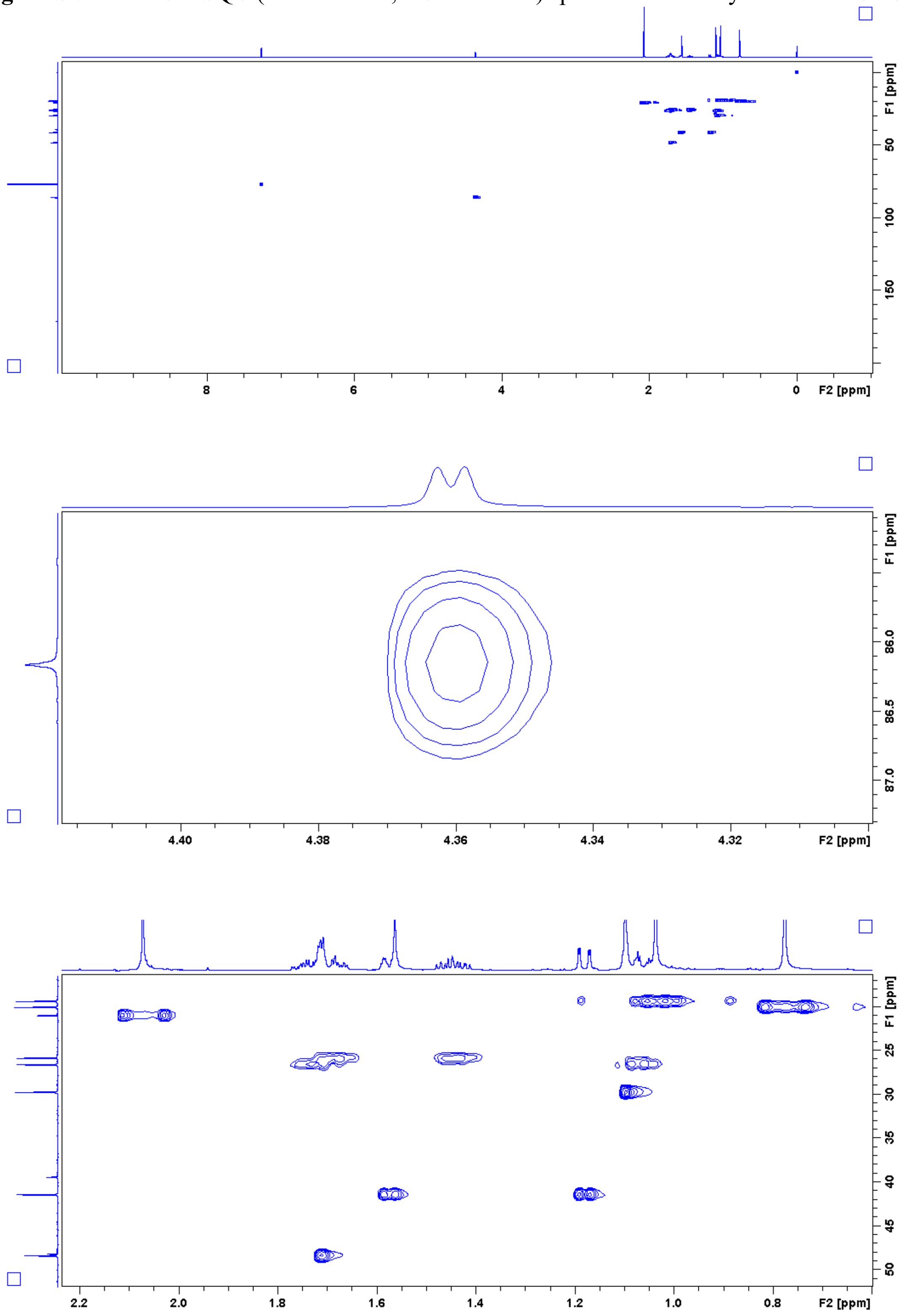

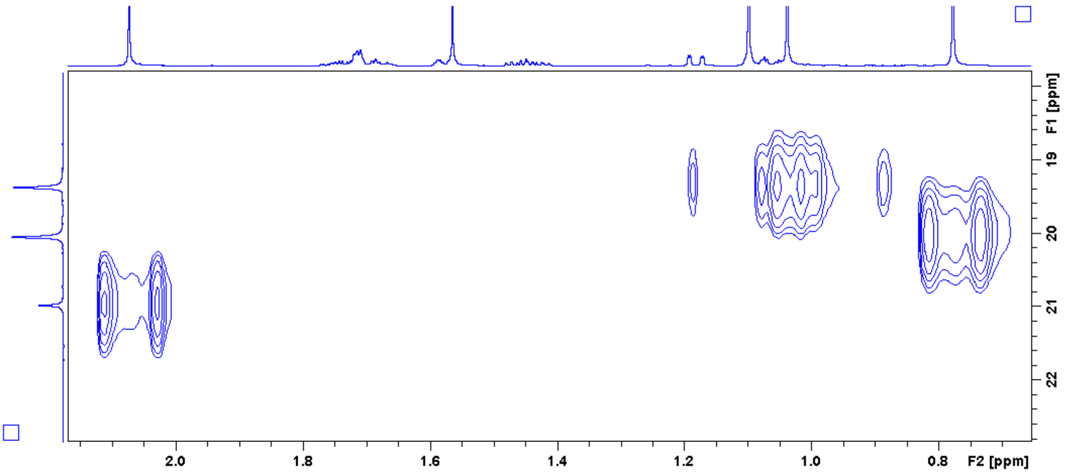
F1 [ppm]
19
20
21
22
2.0
1.8
1.6
1.4
1.2
1.0
0.8
F2 [ppm]

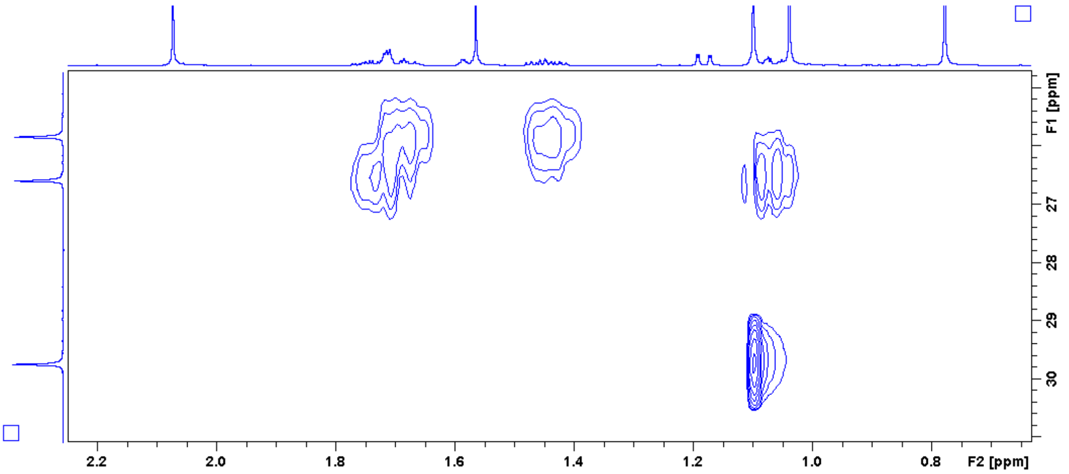
F1 [ppm]
27
28
29
30
2.2
2.0
1.8
1.6
1.4
1.2
1.0
0.8
F2 [ppm]

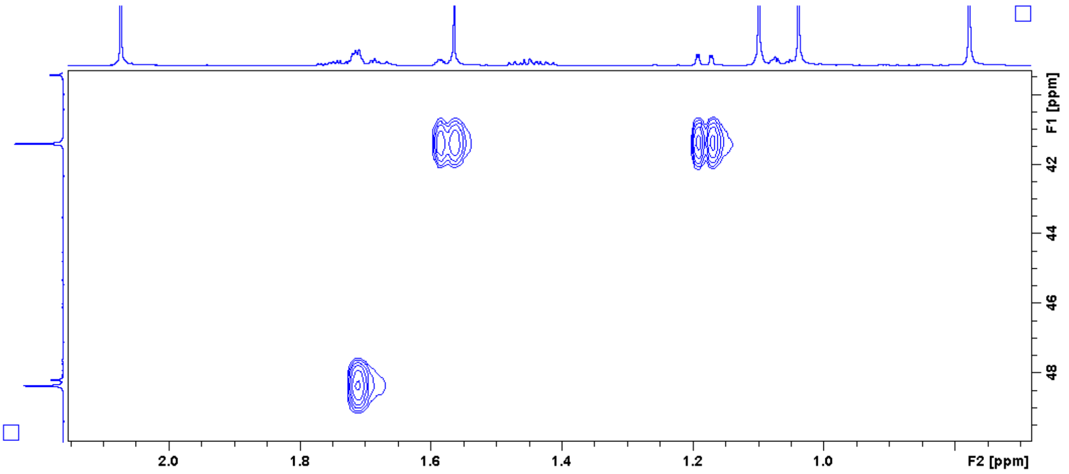
F1 [ppm]
42
44
46
48
2.0
1.8
1.6
1.4
1.2
1.0
F2 [ppm]

**Figure S 31**. $^{1}H$-$^{13}C$-HMBC ($^{1}H$ 500 MHz; $^{13}C$ 125 MHz) spectrum of fenchyl acetate in $CDCl_3$

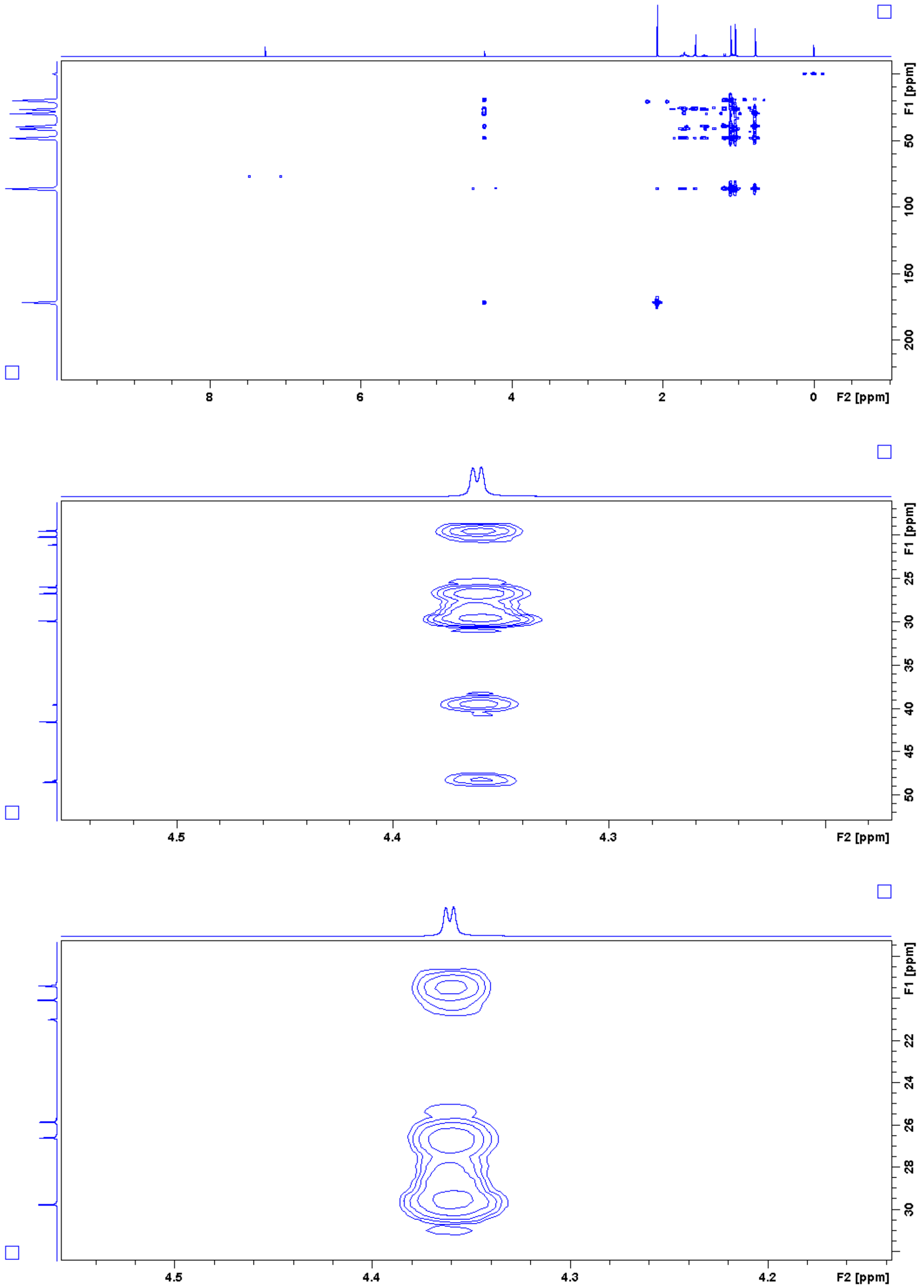

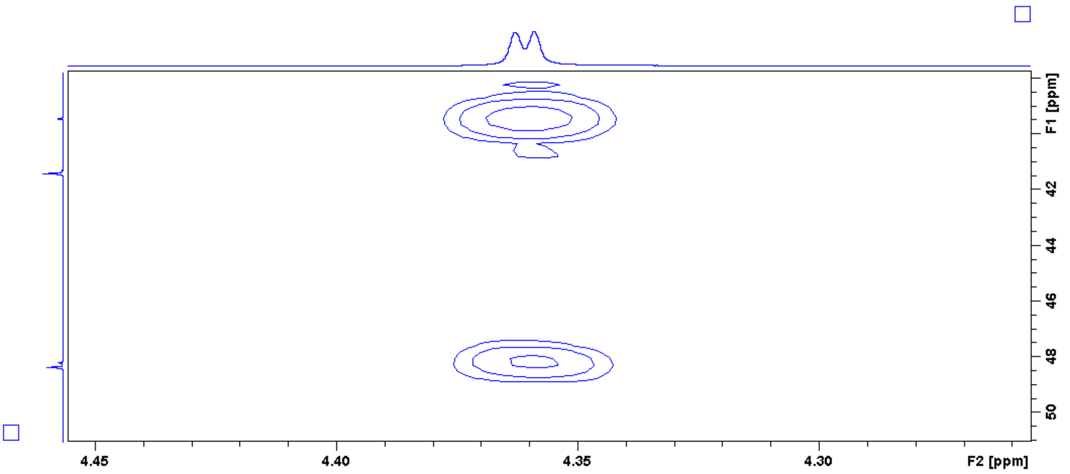

F1 [ppm]
42
44
46
48
50
4.45
4.40
4.35
4.30
F2 [ppm]


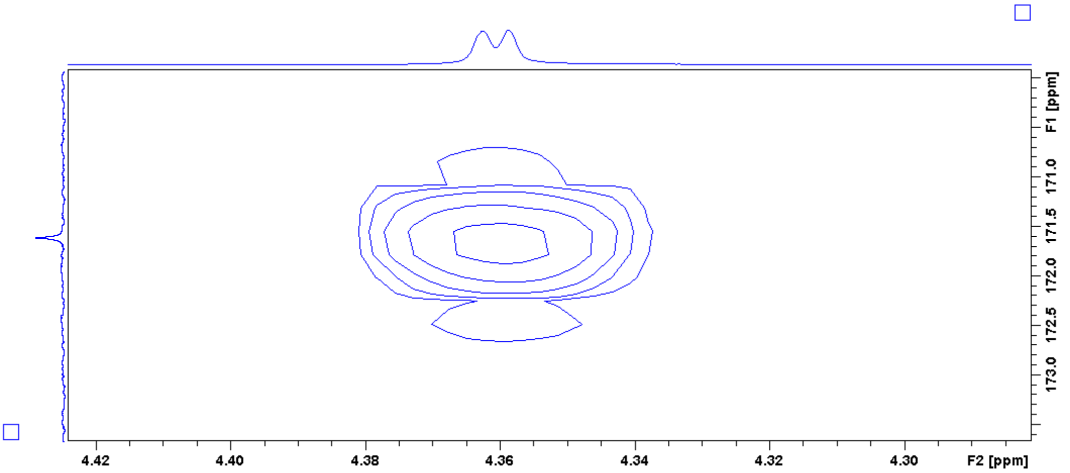

F1 [ppm]
171.0
171.5
172.0
172.5
173.0
4.42
4.40
4.38
4.36
4.34
4.32
4.30
F2 [ppm]


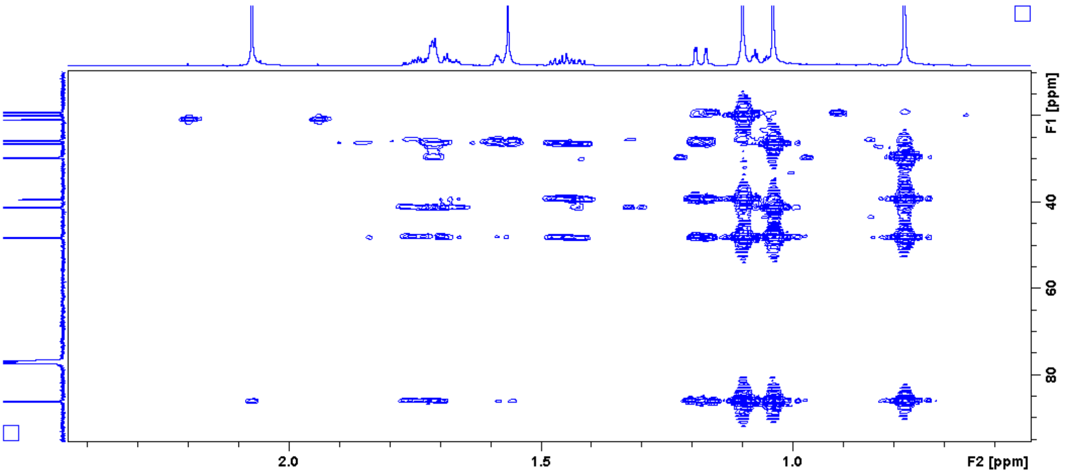

F1 [ppm]
40
60
80
2.0
1.5
1.0
F2 [ppm]

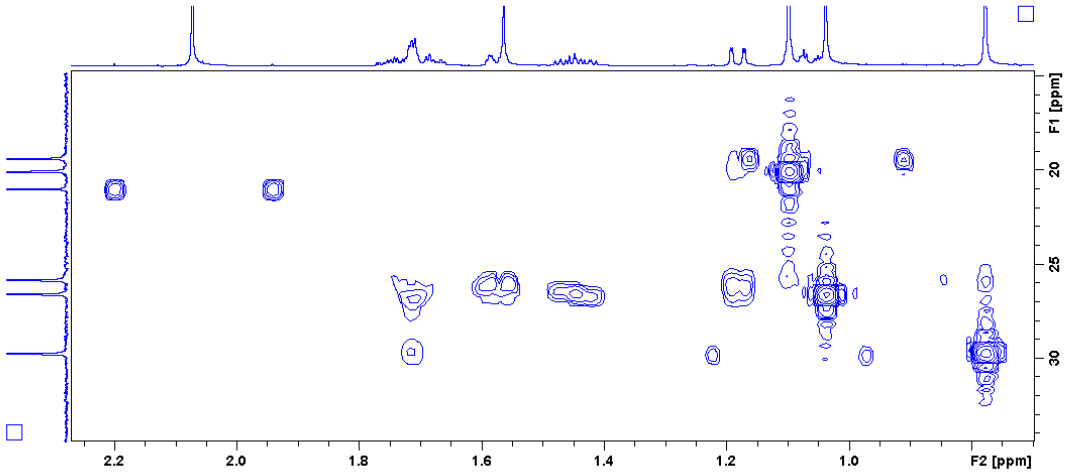
F1 [ppm]
20
25
30
2.2
2.0
1.8
1.6
1.4
1.2
1.0
F2 [ppm]

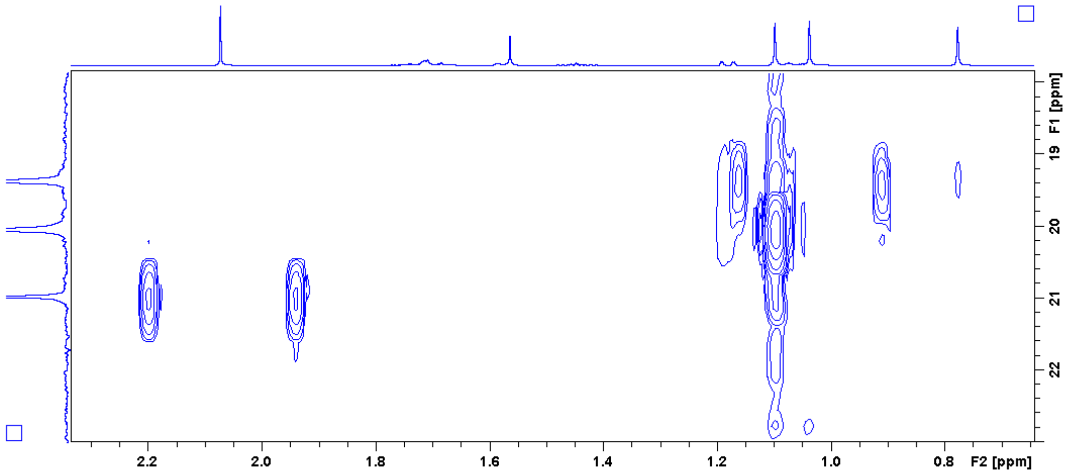
F1 [ppm]
19
20
21
22
2.2
2.0
1.8
1.6
1.4
1.2
1.0
0.8
F2 [ppm]

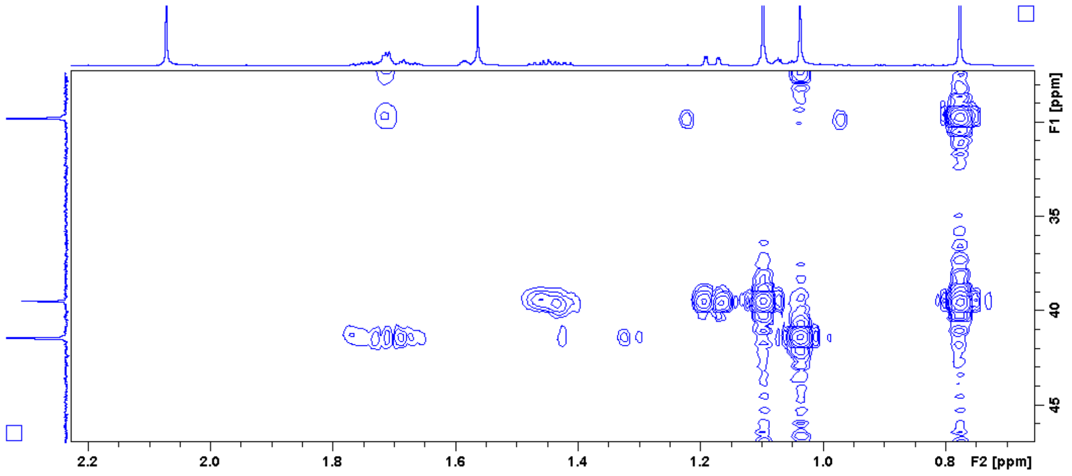
F1 [ppm]
35
40
45
2.2
2.0
1.8
1.6
1.4
1.2
1.0
0.8
F2 [ppm]

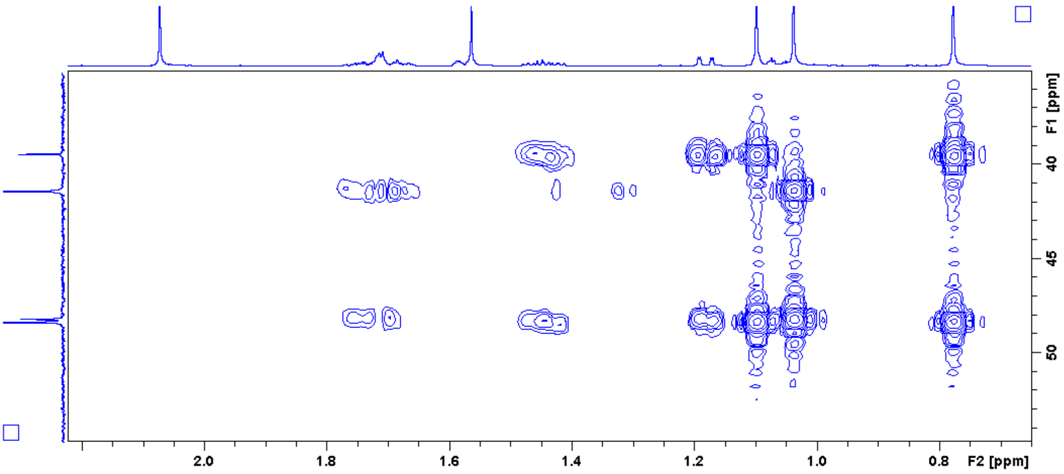
F1 [ppm]
40
45
50
2.0
1.8
1.6
1.4
1.2
1.0
0.8
F2 [ppm]

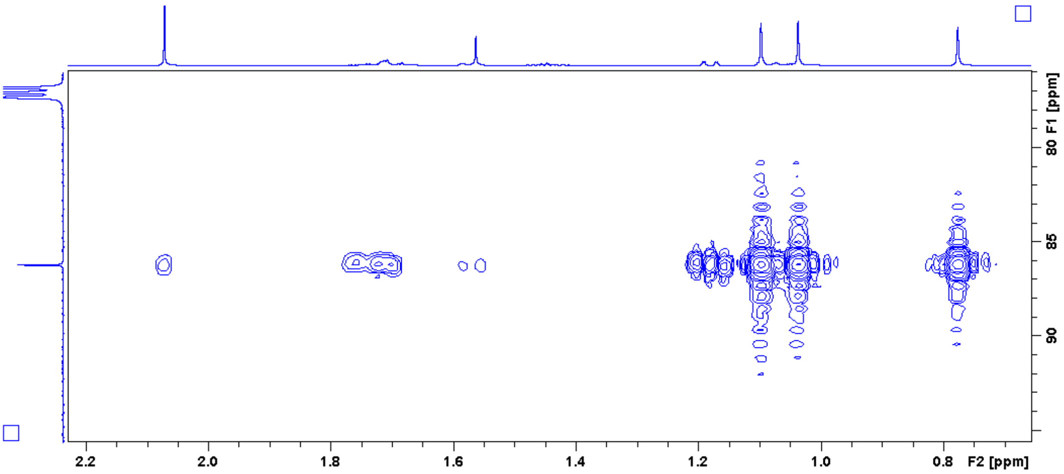
F1 [ppm]
80
85
90
2.2
2.0
1.8
1.6
1.4
1.2
1.0
0.8
F2 [ppm]

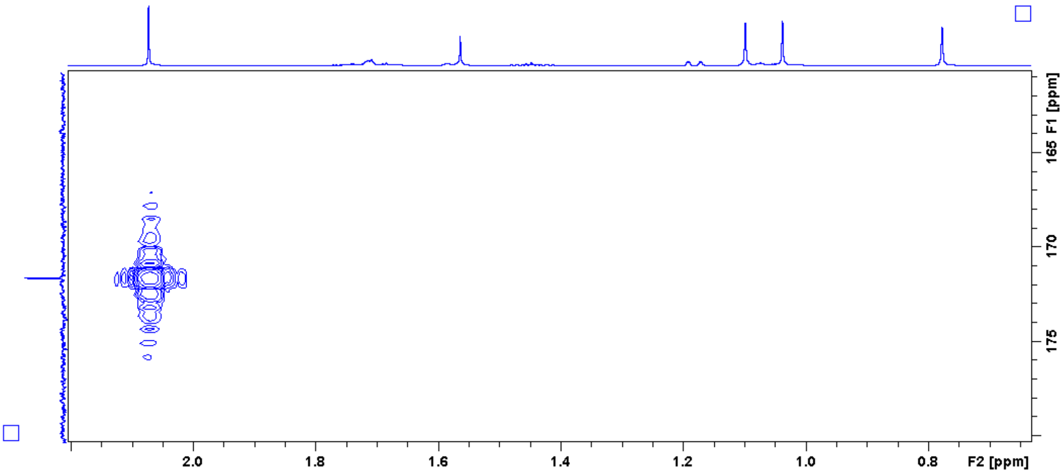
F1 [ppm]
165
170
175
2.0
1.8
1.6
1.4
1.2
1.0
0.8
F2 [ppm]

**Figure S 32.** $^1$H NMR (500 MHz) spectrum of E-caryophyllene in $CDCl_3$

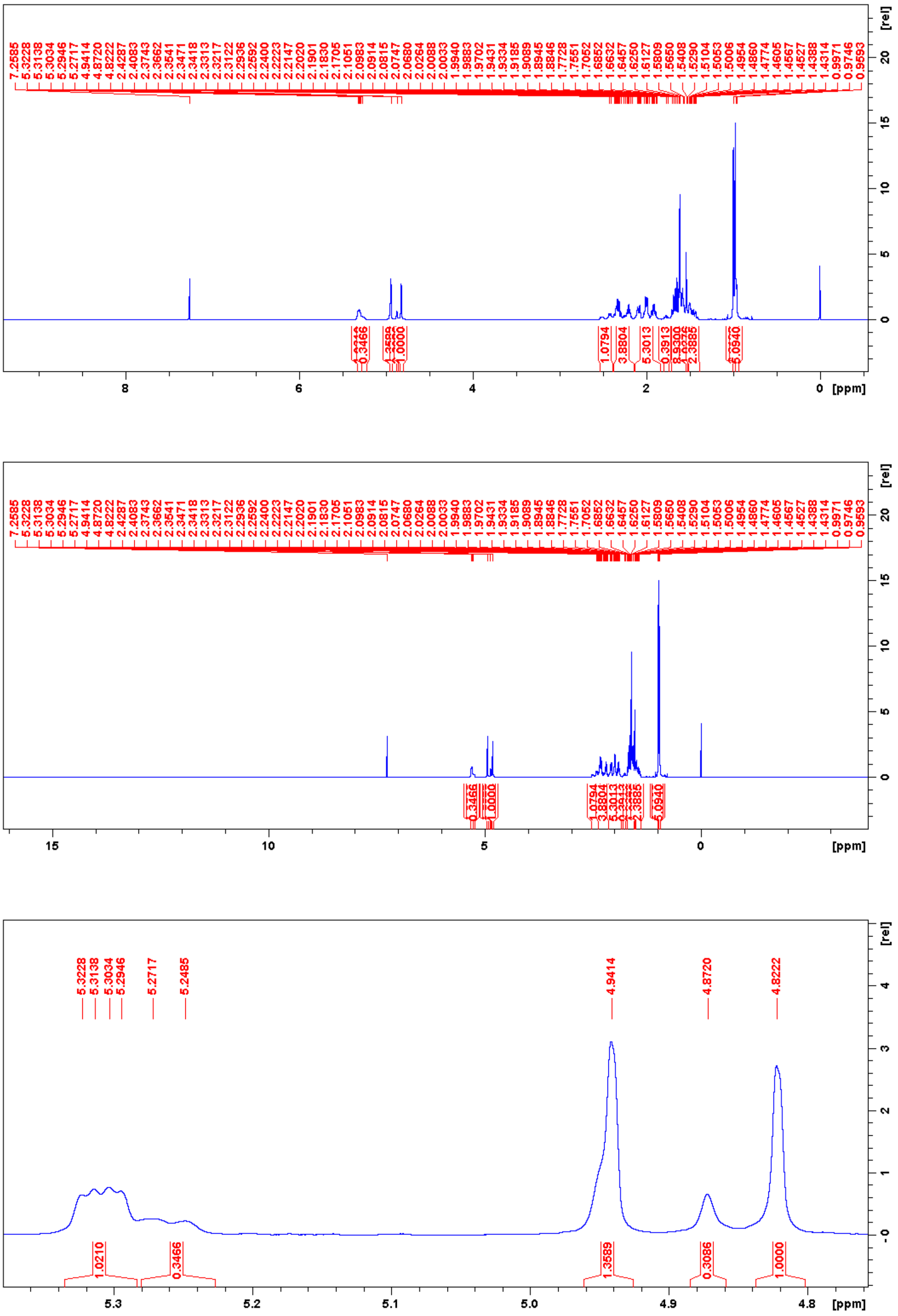

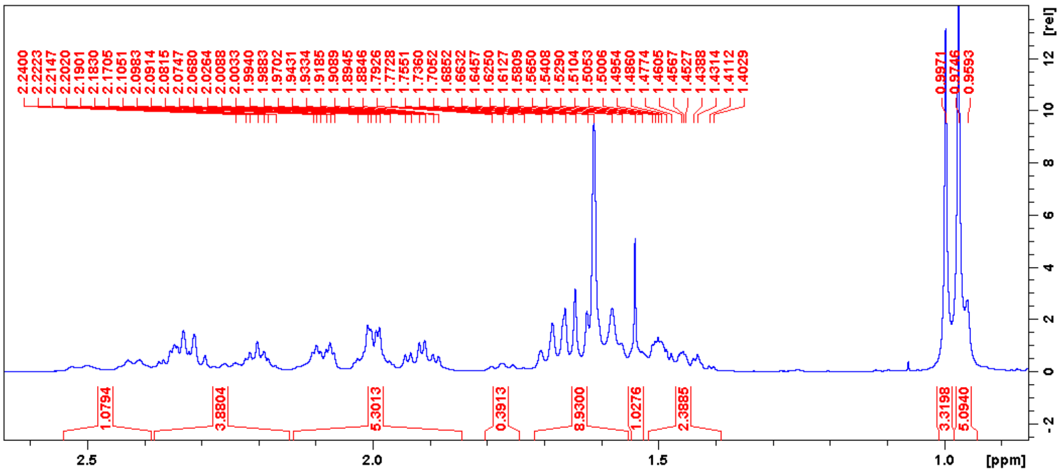
[ppm]
[rel]

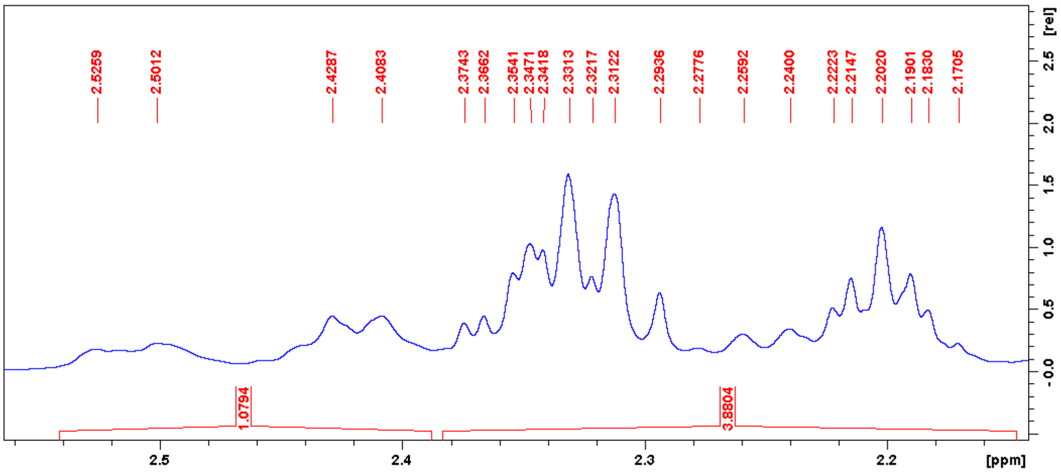
[ppm]
[rel]

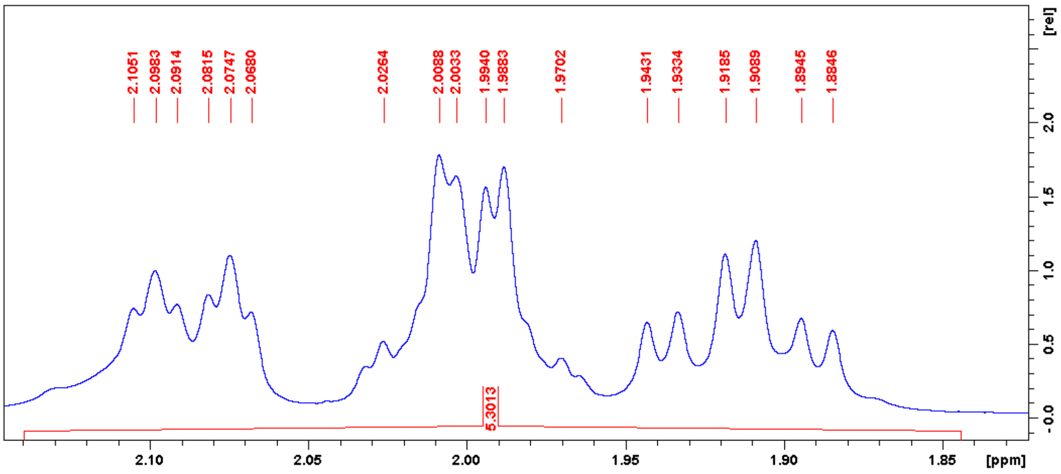
[ppm]
[rel]

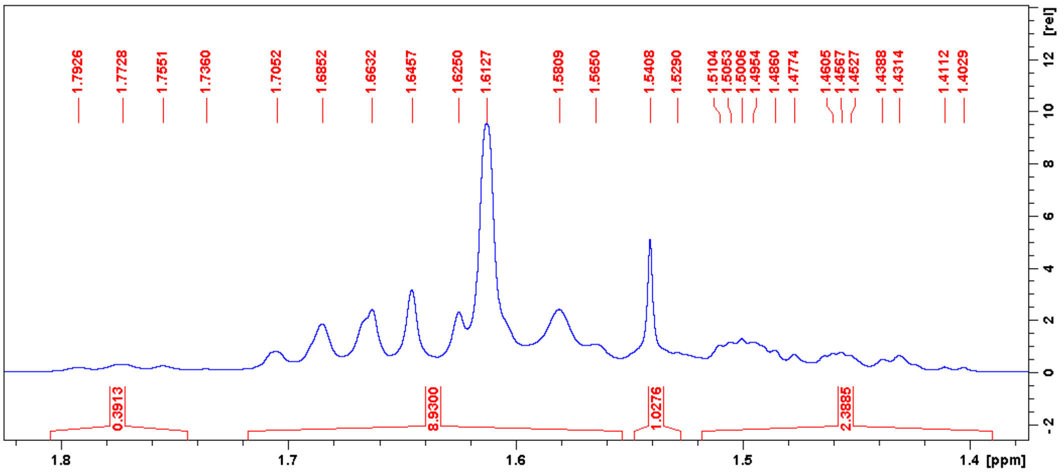

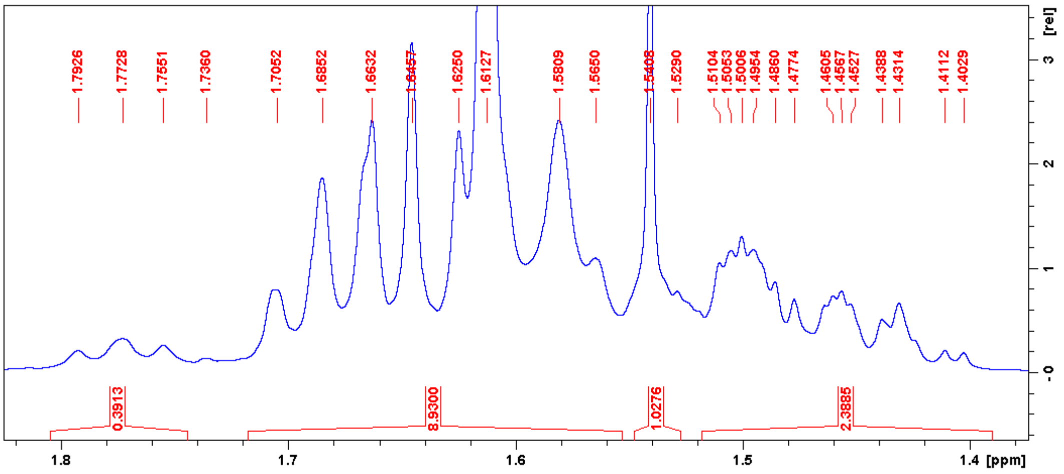

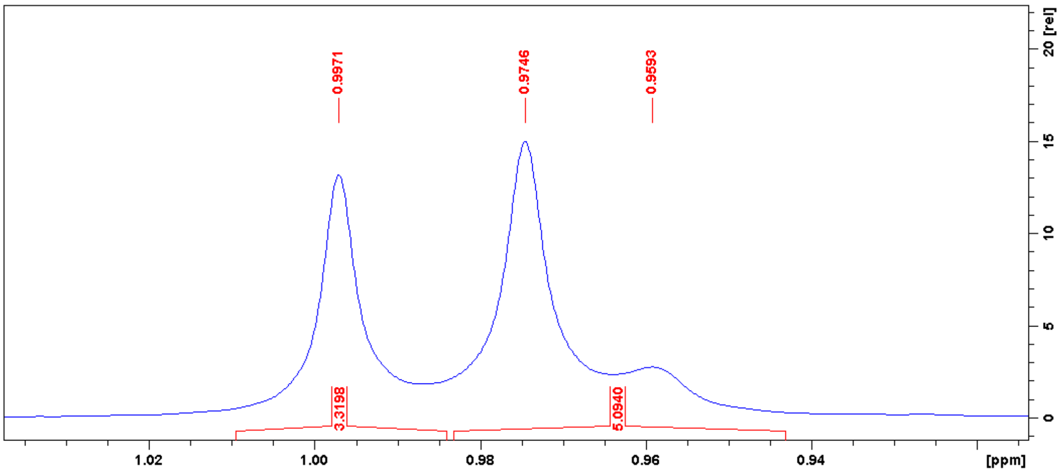

**Figure S 33**. $^{13}$C NMR (125 MHz) spectrum of E-caryophyllene in $CDCl_3$.

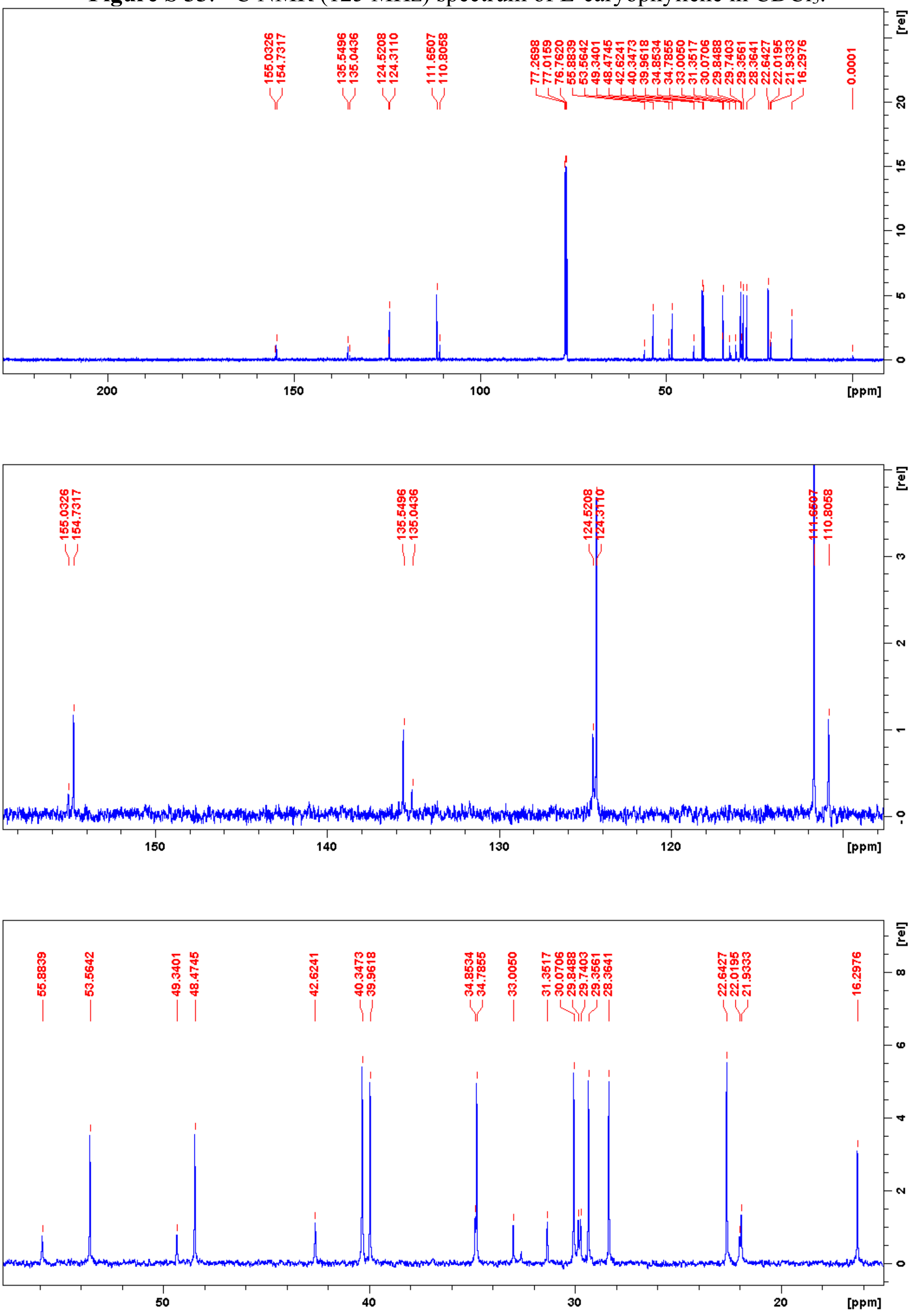

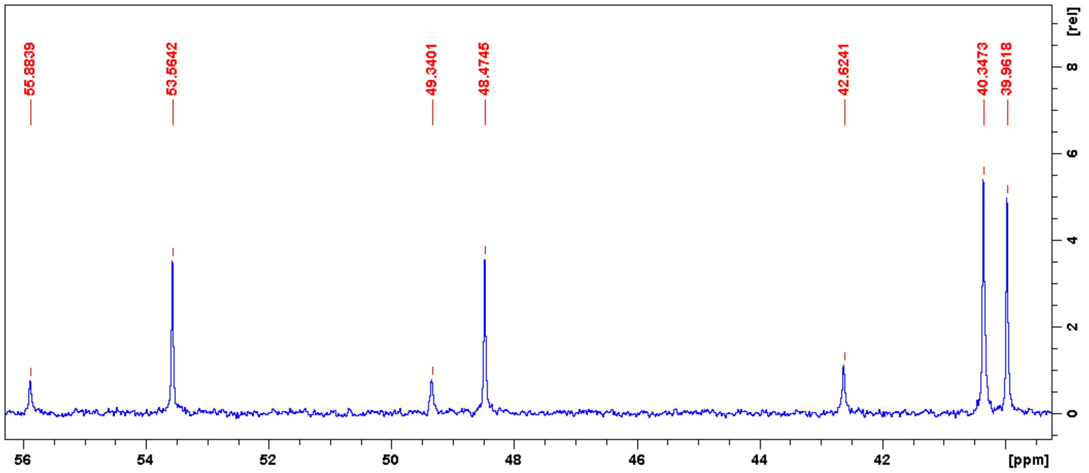
55.8839
53.5642
49.3401
48.4745
42.6241
40.3473
39.9618
[rel]
[ppm]

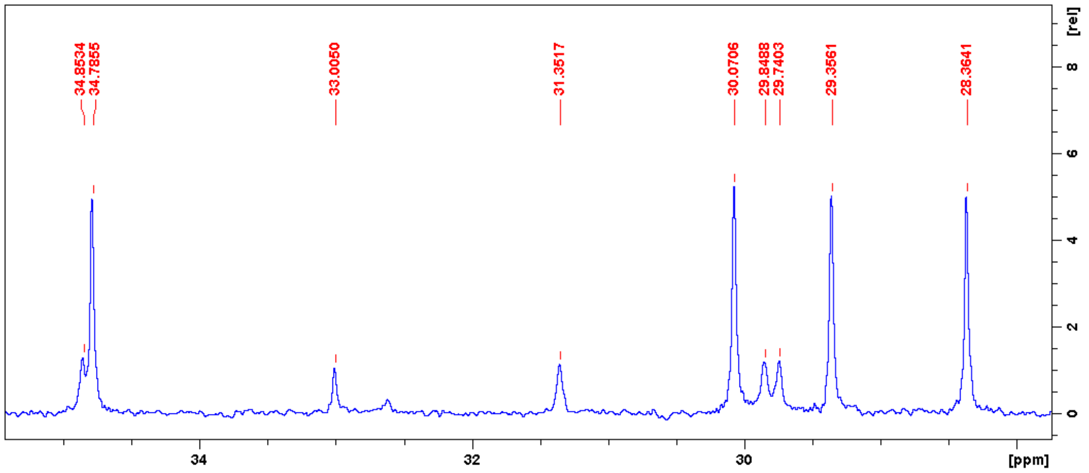
34.8534
34.7855
33.0050
31.3517
30.0706
29.8488
29.7403
29.3561
28.3641
[rel]
[ppm]

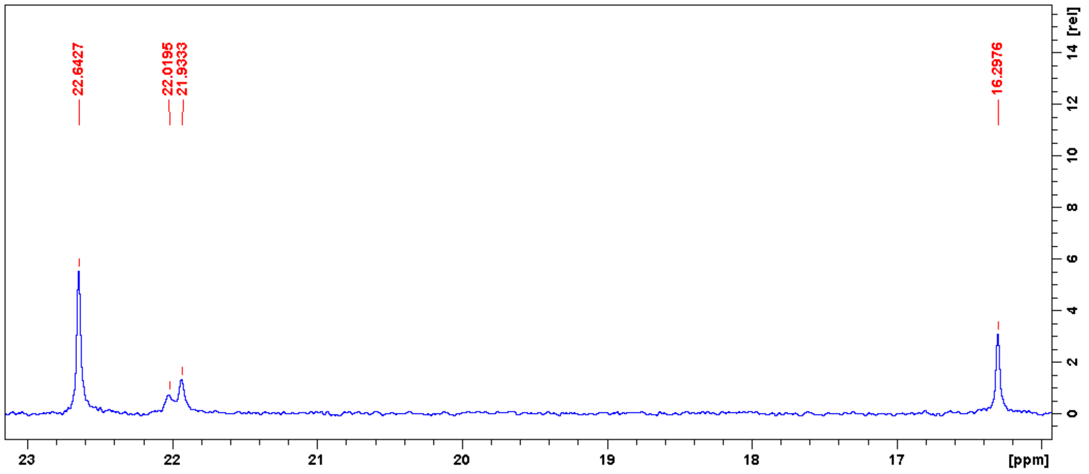
22.6427
22.0195
21.9333
16.2976
[rel]
[ppm]

**Figure S 34.** $^{13}$C NMR and DEPT 135 (125 MHz) spectrum of E-caryophyllene in $CDCl_3$

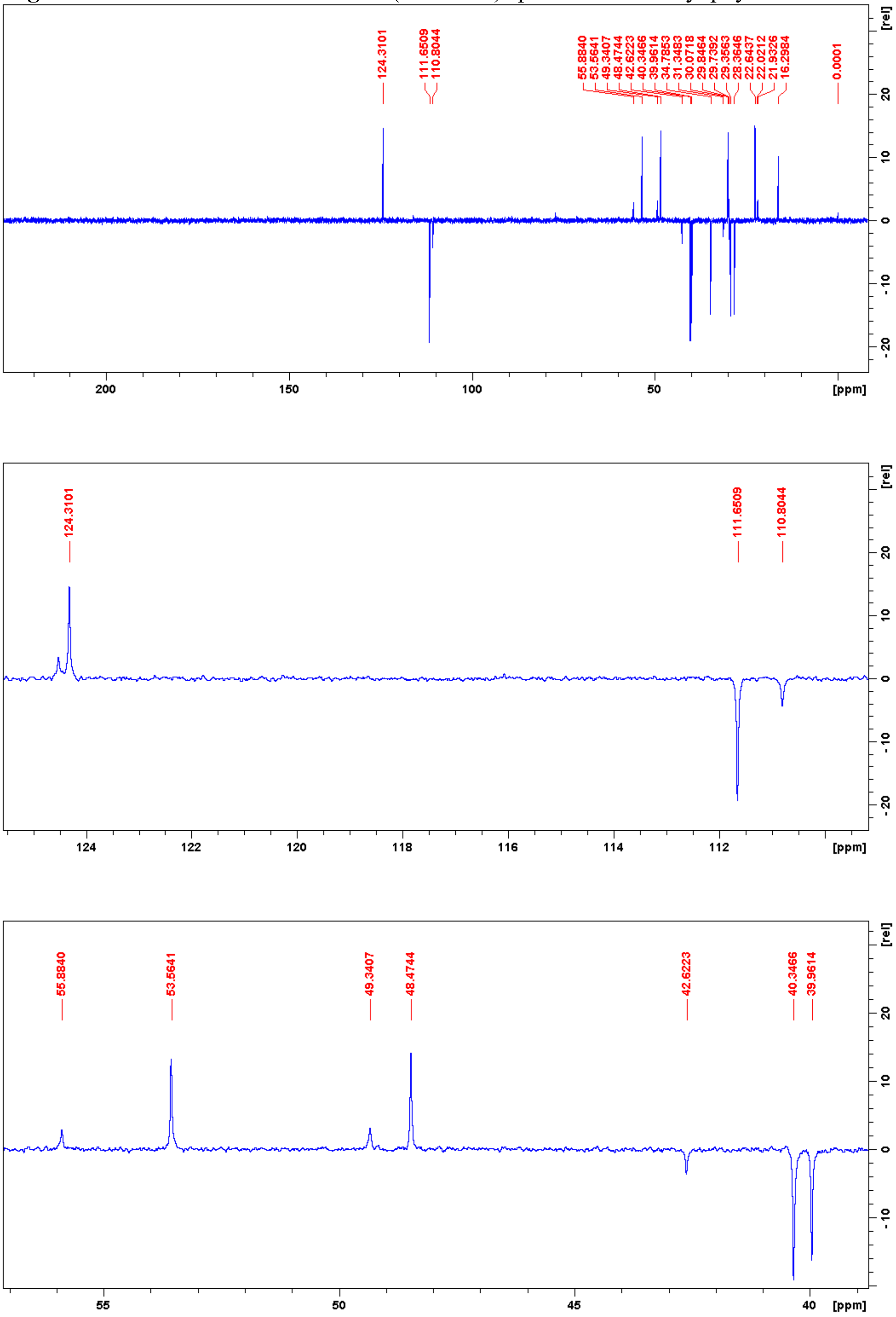

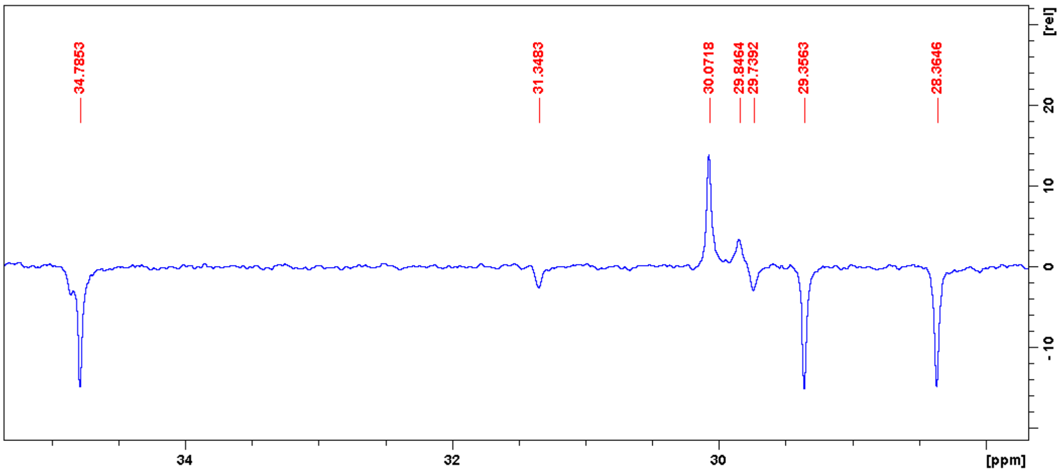
34.7853
31.3483
30.0718
29.8464
29.7392
29.3563
28.3646
[rel]
20
10
0
-10
34
32
30
[ppm]

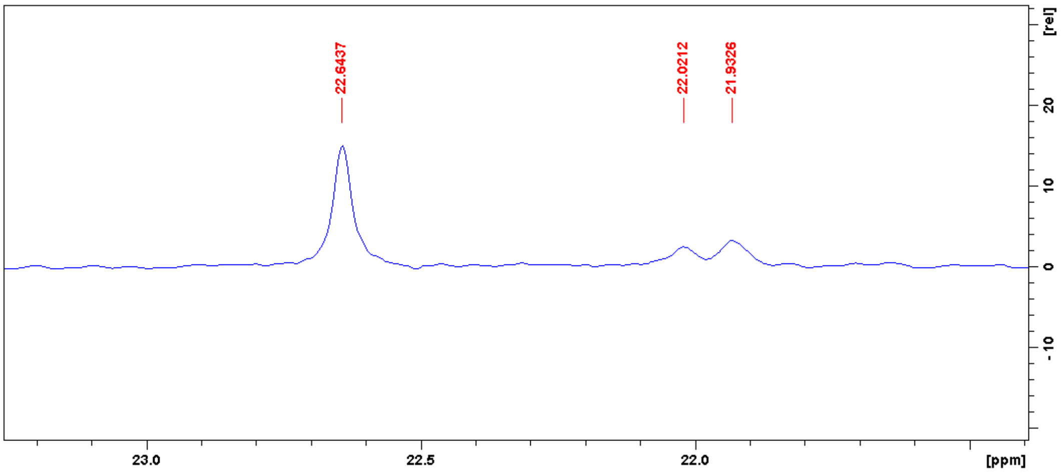
22.6437
22.0212
21.9326
[rel]
20
10
0
-10
23.0
22.5
22.0
[ppm]

**Figure S 35**. $^1$H-$^1$H-COSY (500 MHz) spectrum of E-caryophyllene in CDCl$_3$

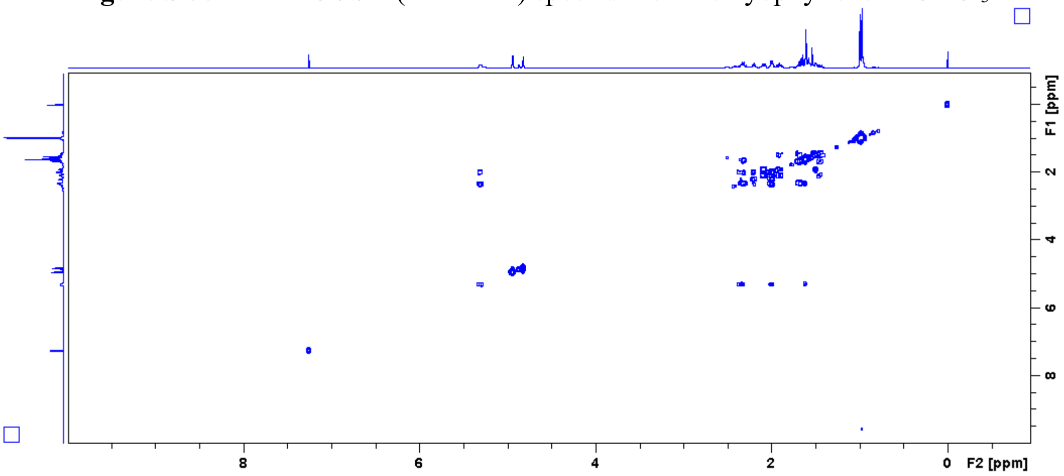


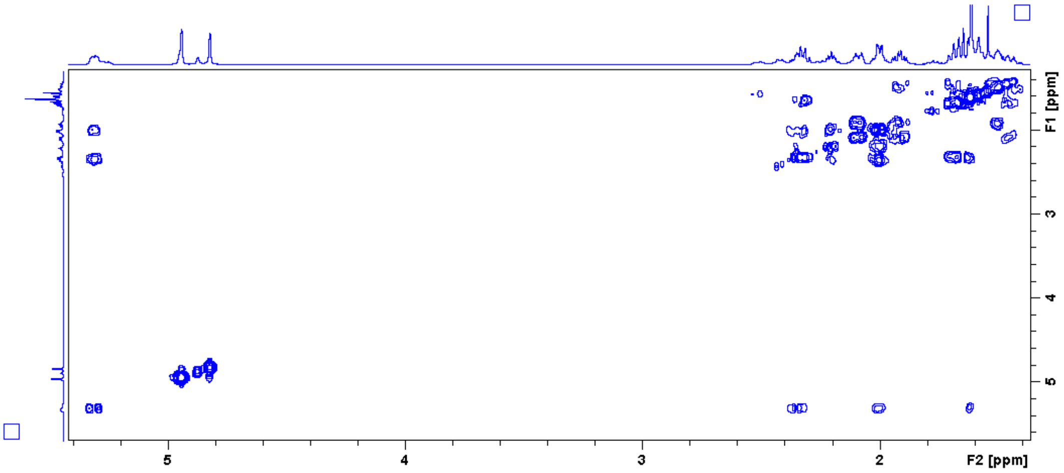


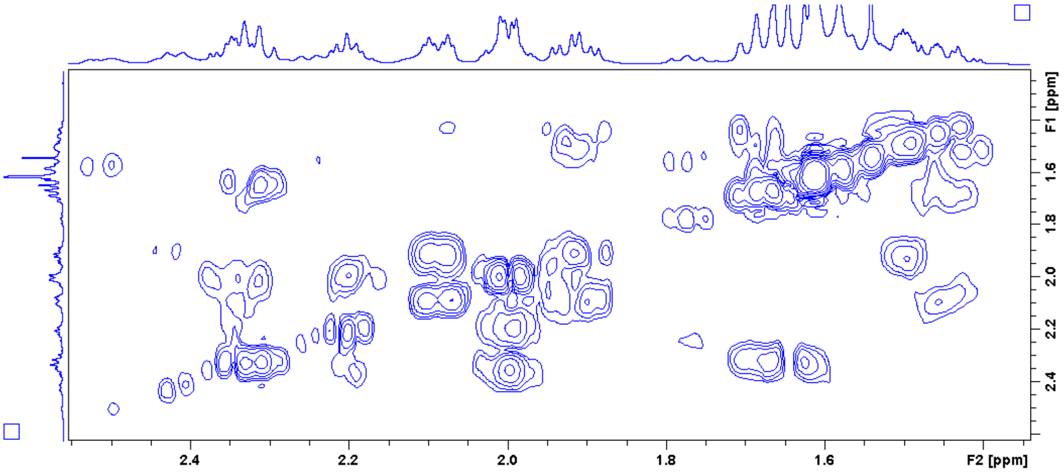

**Figure S 36**. $^1$H-$^{13}$C-HSQC ($^1$H 500 MHz; $^{13}$C 125 MHz) spectrum of E-caryophyllene in $CDCl_3$

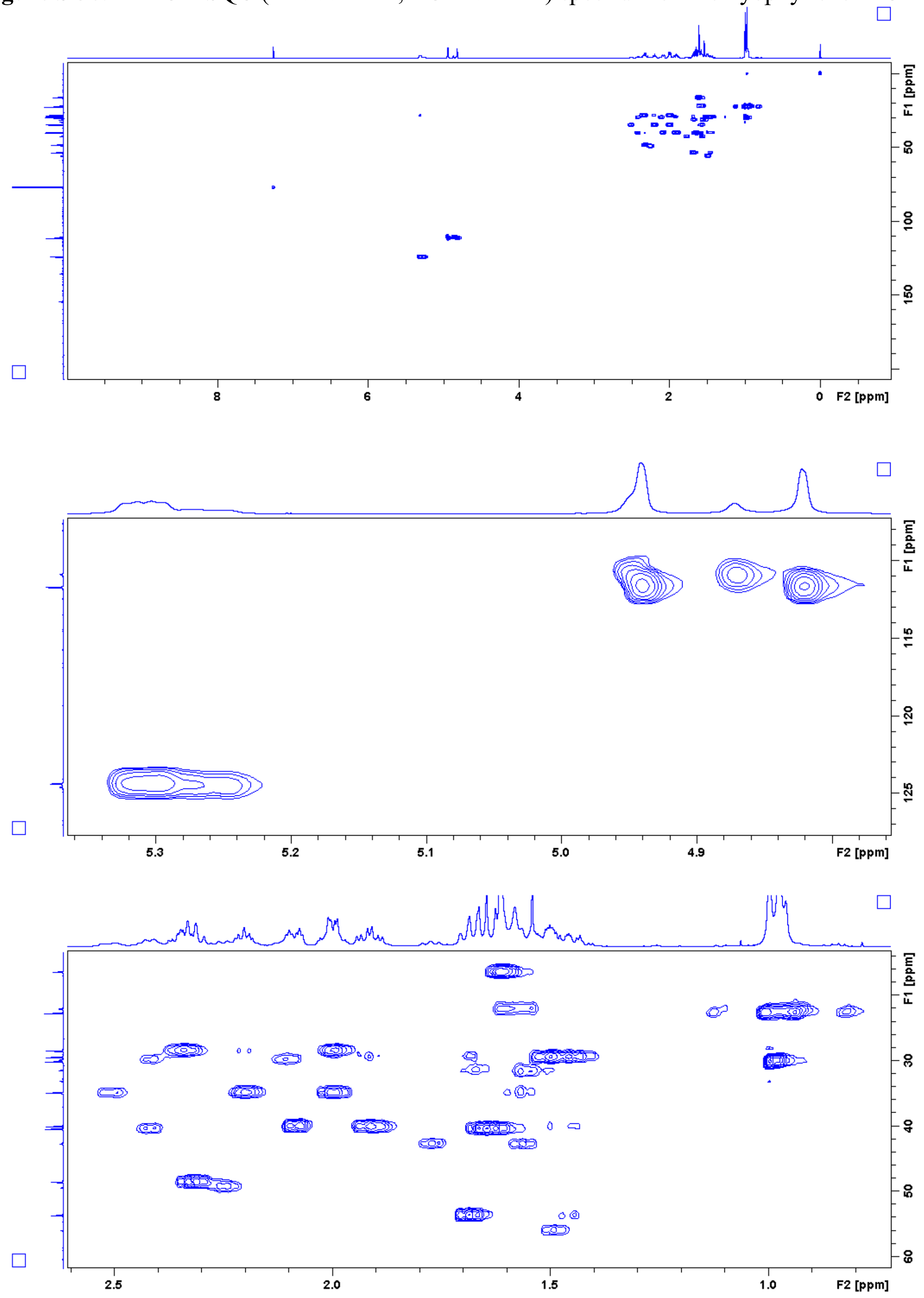

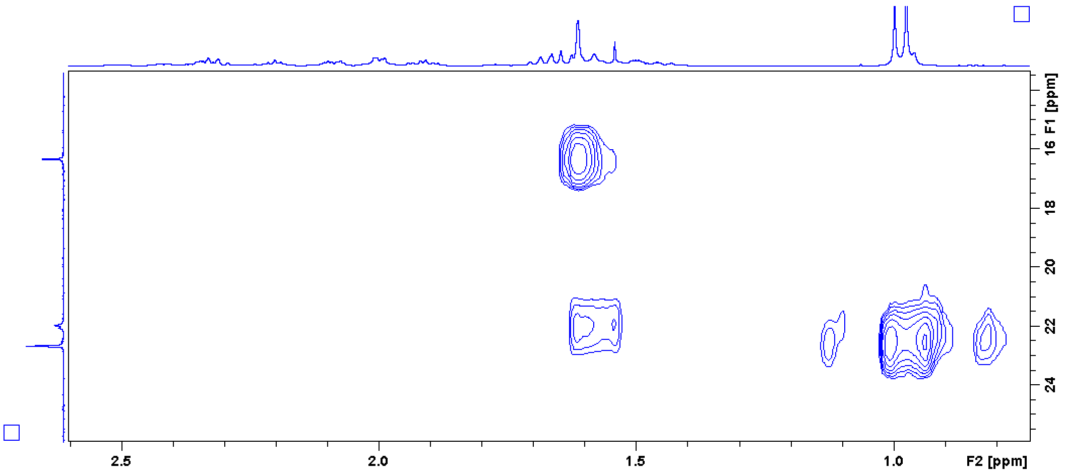
16 F1 [ppm]
18
20
22
24
2.5
2.0
1.5
1.0
F2 [ppm]

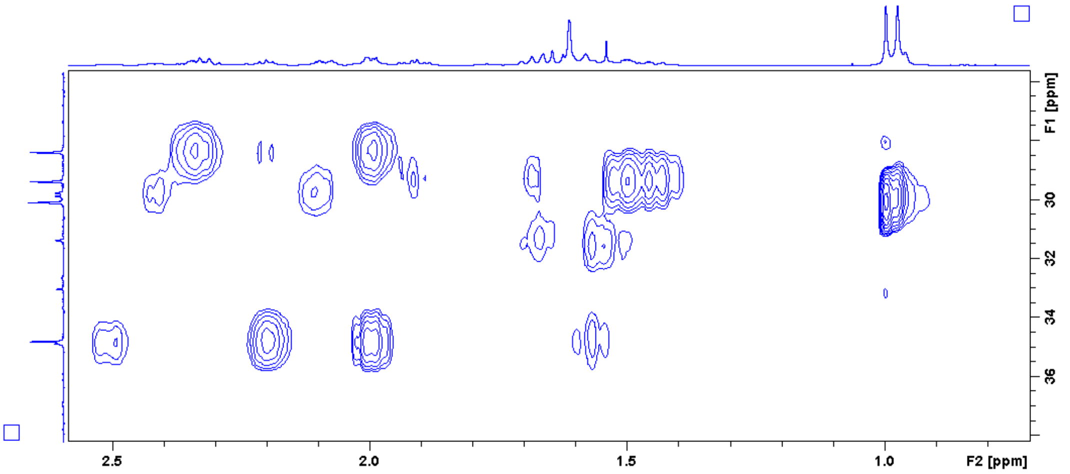
F1 [ppm]
30
32
34
36
2.5
2.0
1.5
1.0
F2 [ppm]

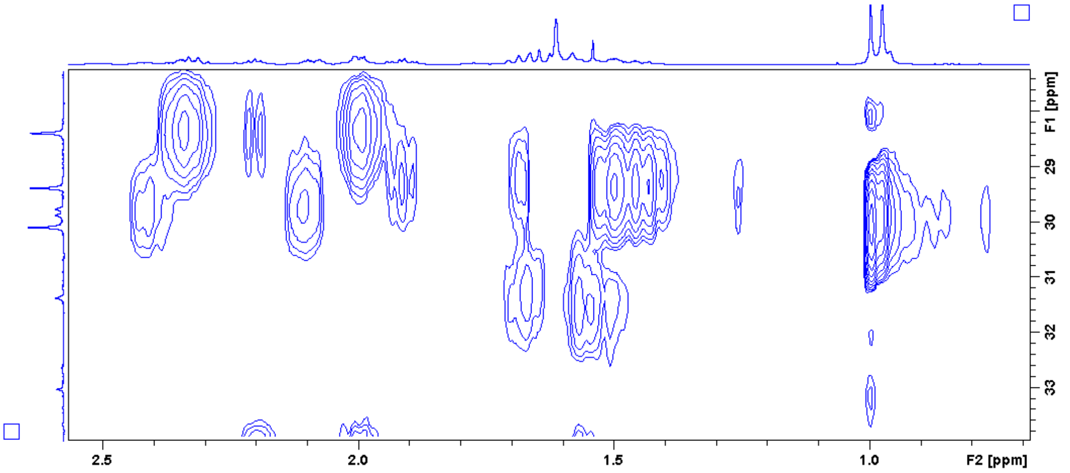
F1 [ppm]
29
30
31
32
33
2.5
2.0
1.5
1.0
F2 [ppm]

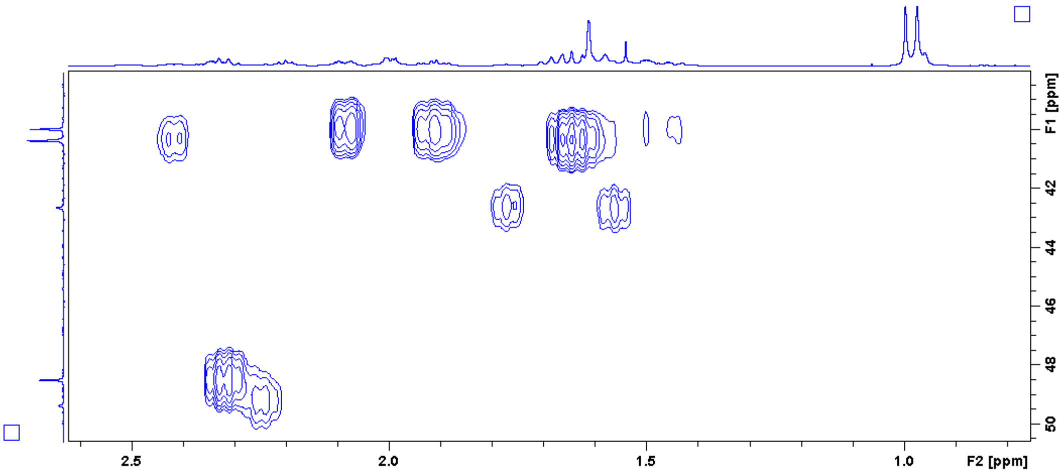
F1 [ppm]
42
44
46
48
50
2.5
2.0
1.5
1.0
F2 [ppm]

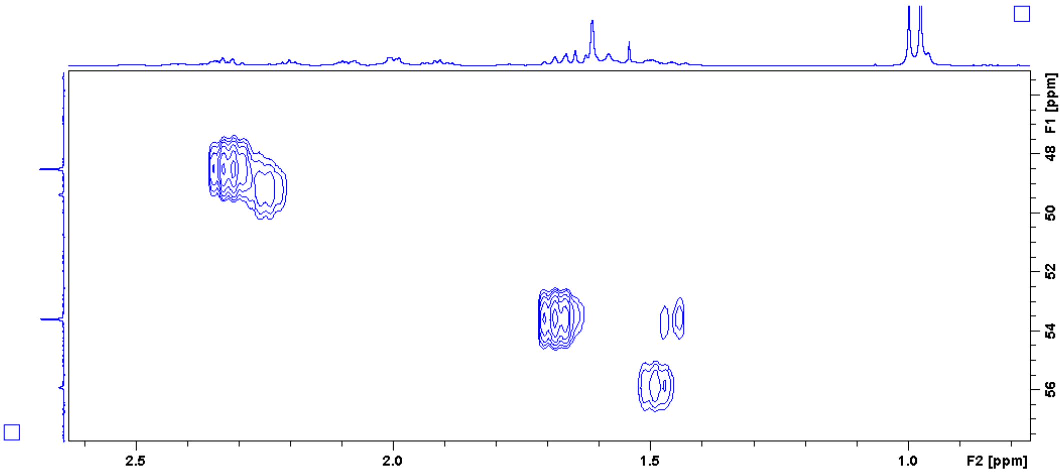
F1 [ppm]
48
50
52
54
56
2.5
2.0
1.5
1.0
F2 [ppm]

**Figure S 37**. $^{1}$H-$^{13}$C-HMBC ($^{1}$H 500 MHz; $^{13}$C 125 MHz) spectrum of E-caryophyllene in $CDCl_3$

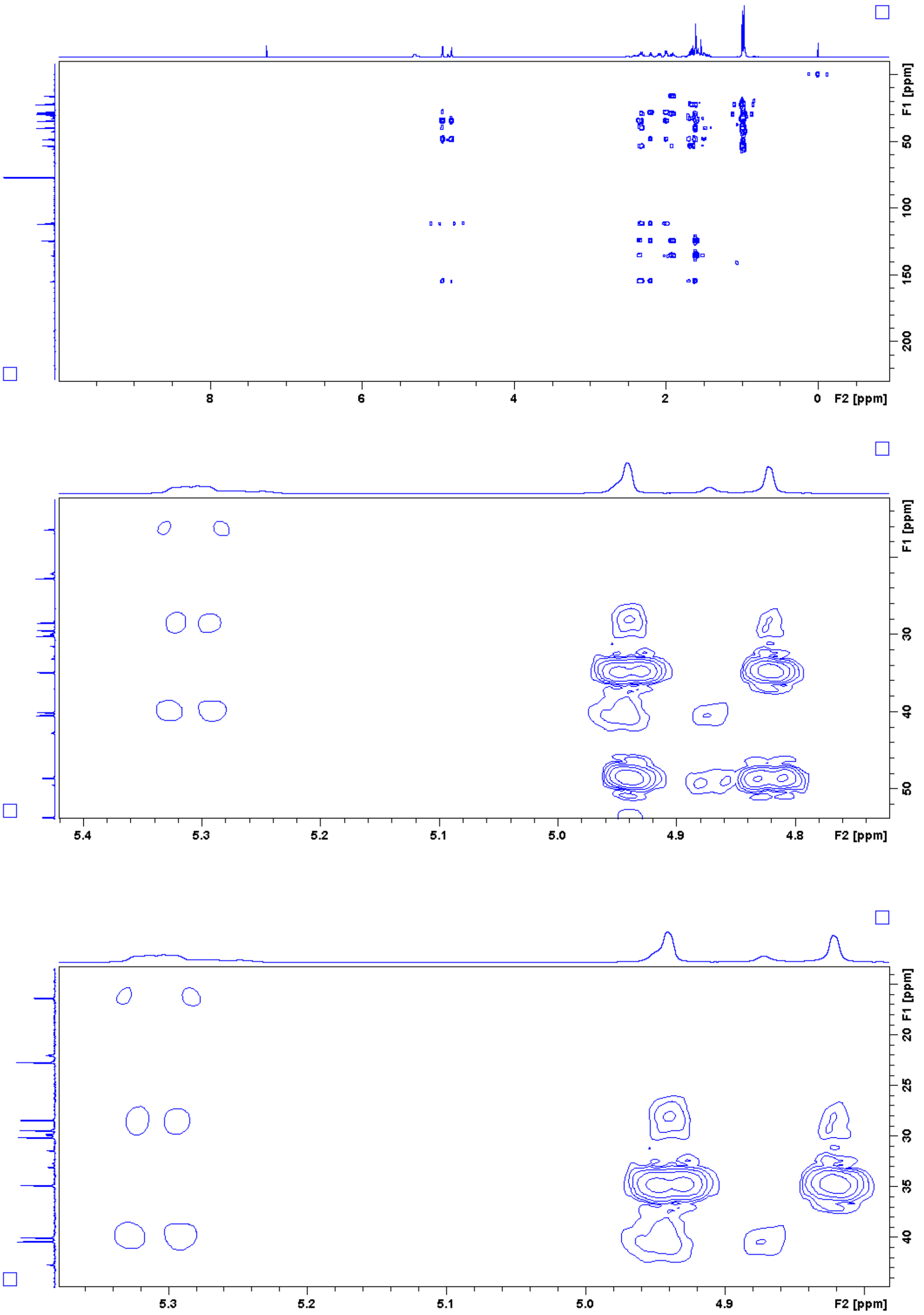

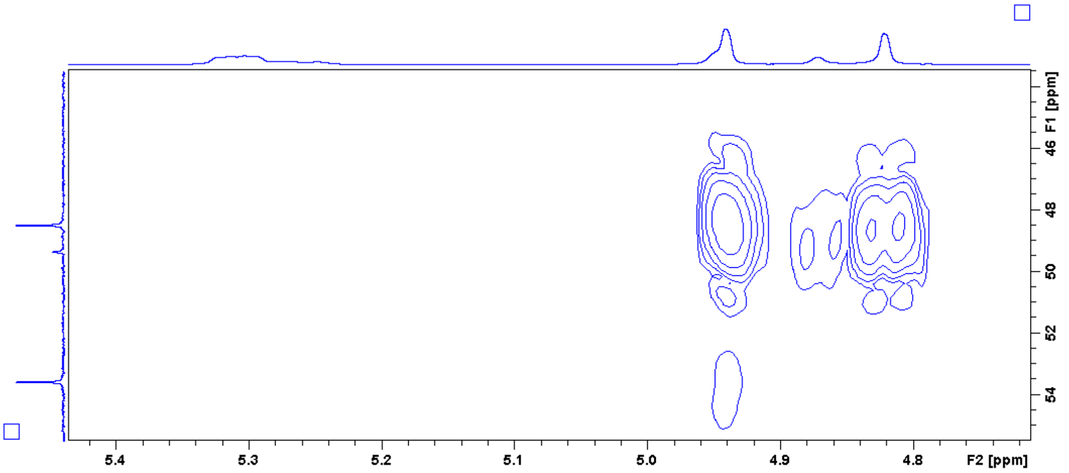
F1 [ppm]
46
48
50
52
54
5.4
5.3
5.2
5.1
5.0
4.9
4.8
F2 [ppm]

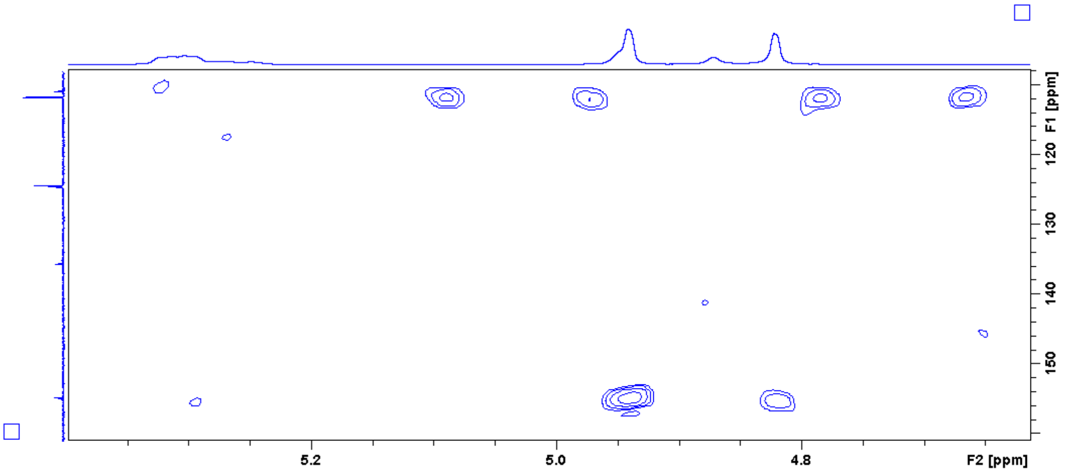
F1 [ppm]
120
130
140
150
5.2
5.0
4.8
F2 [ppm]

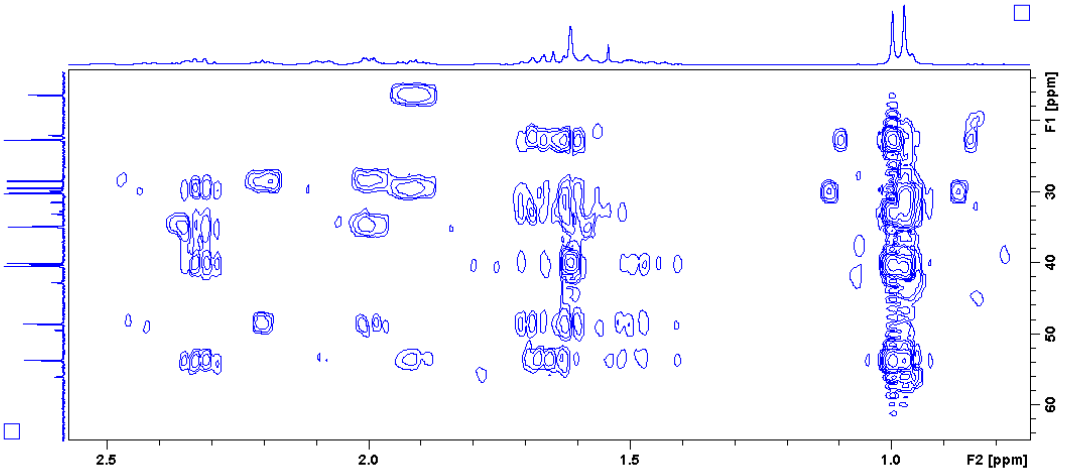
F1 [ppm]
30
40
50
60
2.5
2.0
1.5
1.0
F2 [ppm]

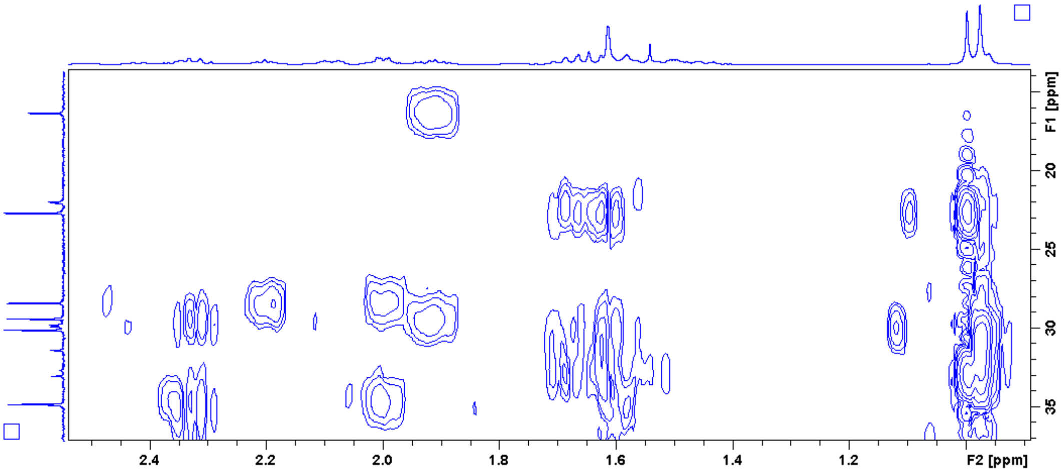
F1 [ppm]
20
25
30
35
2.4
2.2
2.0
1.8
1.6
1.4
1.2
F2 [ppm]

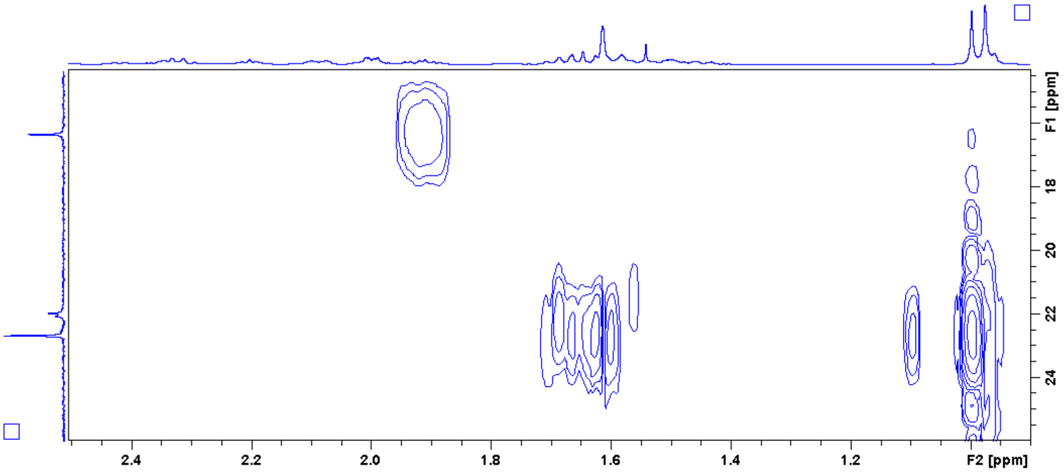
F1 [ppm]
18
20
22
24
2.4
2.2
2.0
1.8
1.6
1.4
1.2
F2 [ppm]

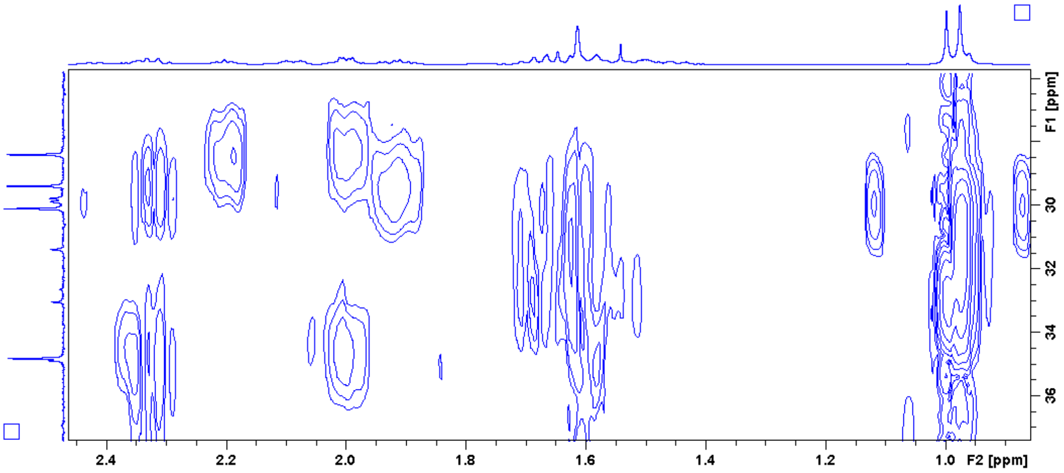
F1 [ppm]
30
32
34
36
2.4
2.2
2.0
1.8
1.6
1.4
1.2
1.0
F2 [ppm]

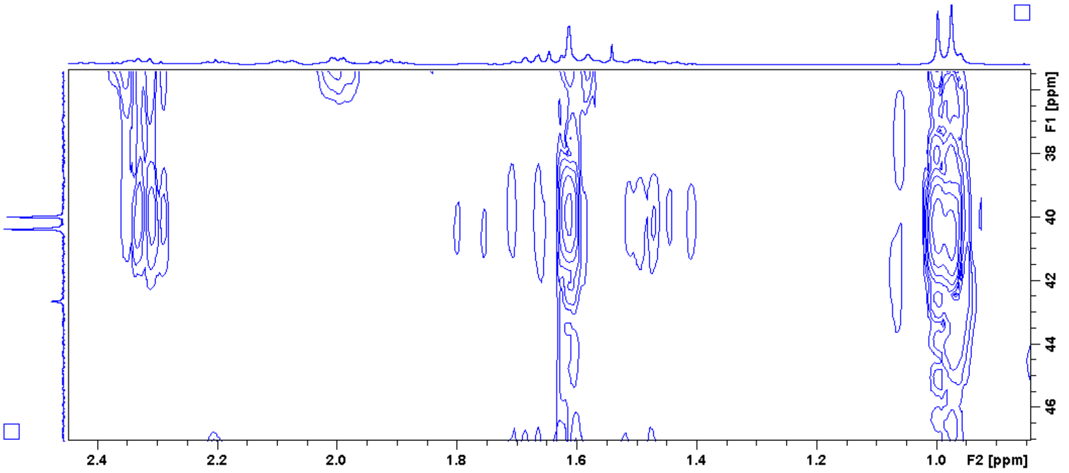
F1 [ppm]
38
40
42
44
46
2.4
2.2
2.0
1.8
1.6
1.4
1.2
1.0
F2 [ppm]

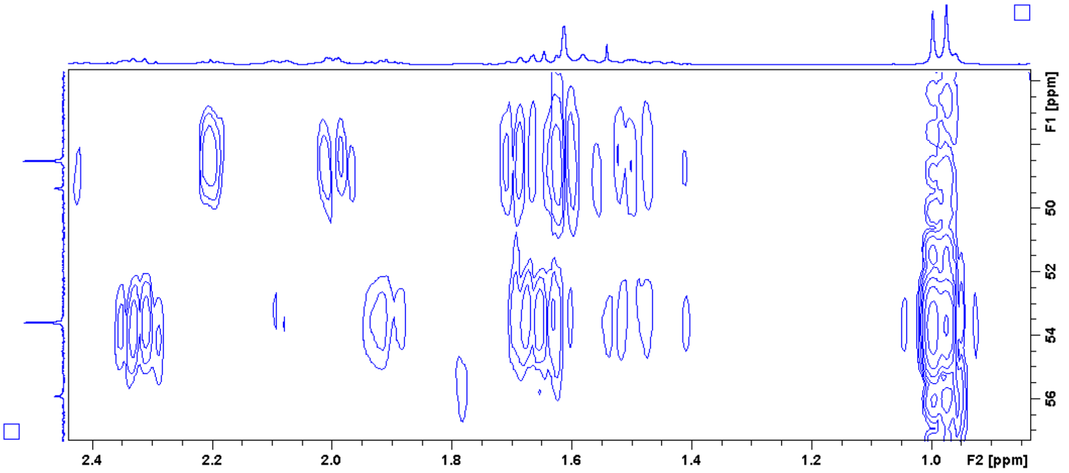
F1 [ppm]
50
52
54
56
2.4
2.2
2.0
1.8
1.6
1.4
1.2
1.0
F2 [ppm]

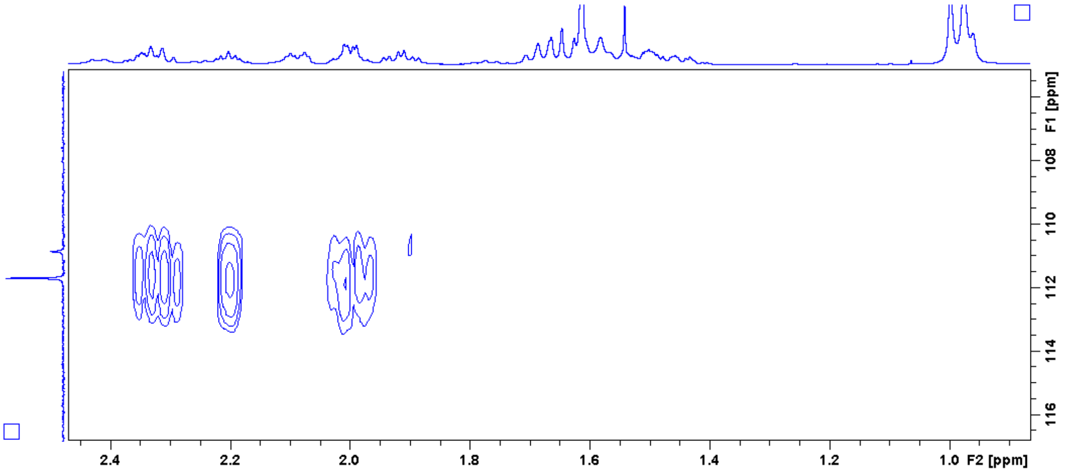
F1 [ppm]
108
110
112
114
116
2.4
2.2
2.0
1.8
1.6
1.4
1.2
1.0
F2 [ppm]

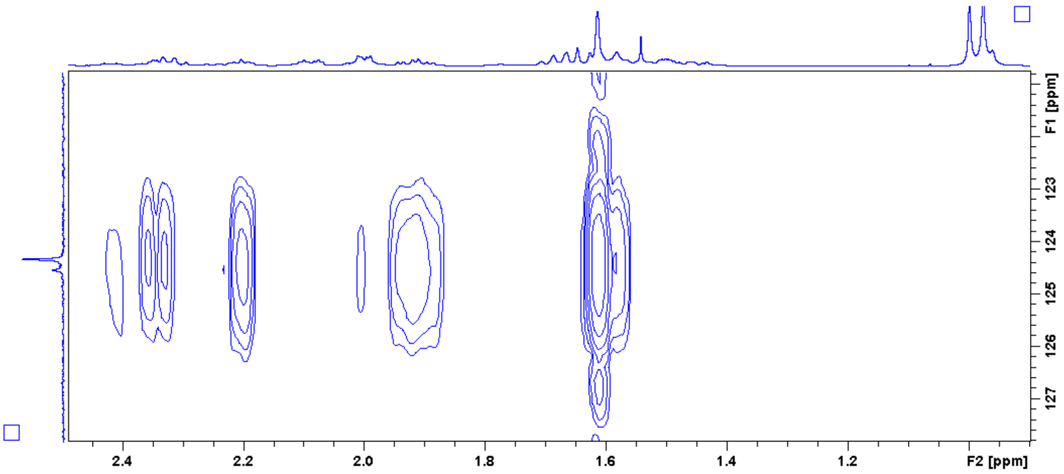

F1 [ppm]
123
124
125
126
127
2.4
2.2
2.0
1.8
1.6
1.4
1.2
F2 [ppm]


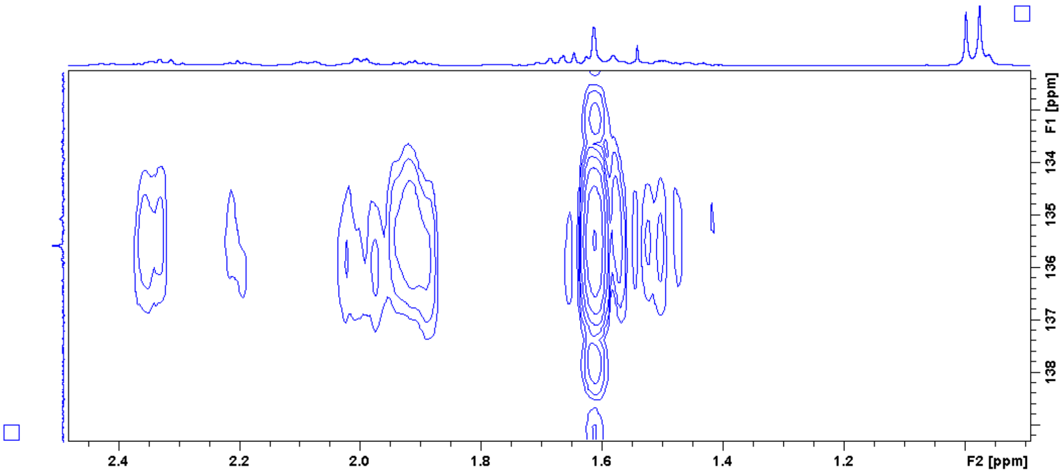

F1 [ppm]
134
135
136
137
138
2.4
2.2
2.0
1.8
1.6
1.4
1.2
F2 [ppm]


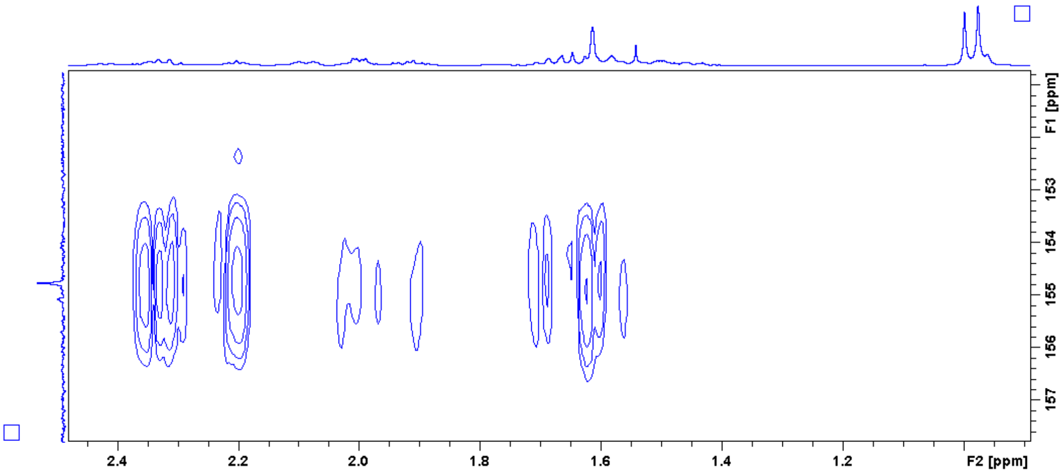

F1 [ppm]
153
154
155
156
157
2.4
2.2
2.0
1.8
1.6
1.4
1.2
F2 [ppm]

**Figure S 38.** $^{1}H$ NMR (500 MHz) spectrum of caryophyllene oxide in $CDCl_3$.

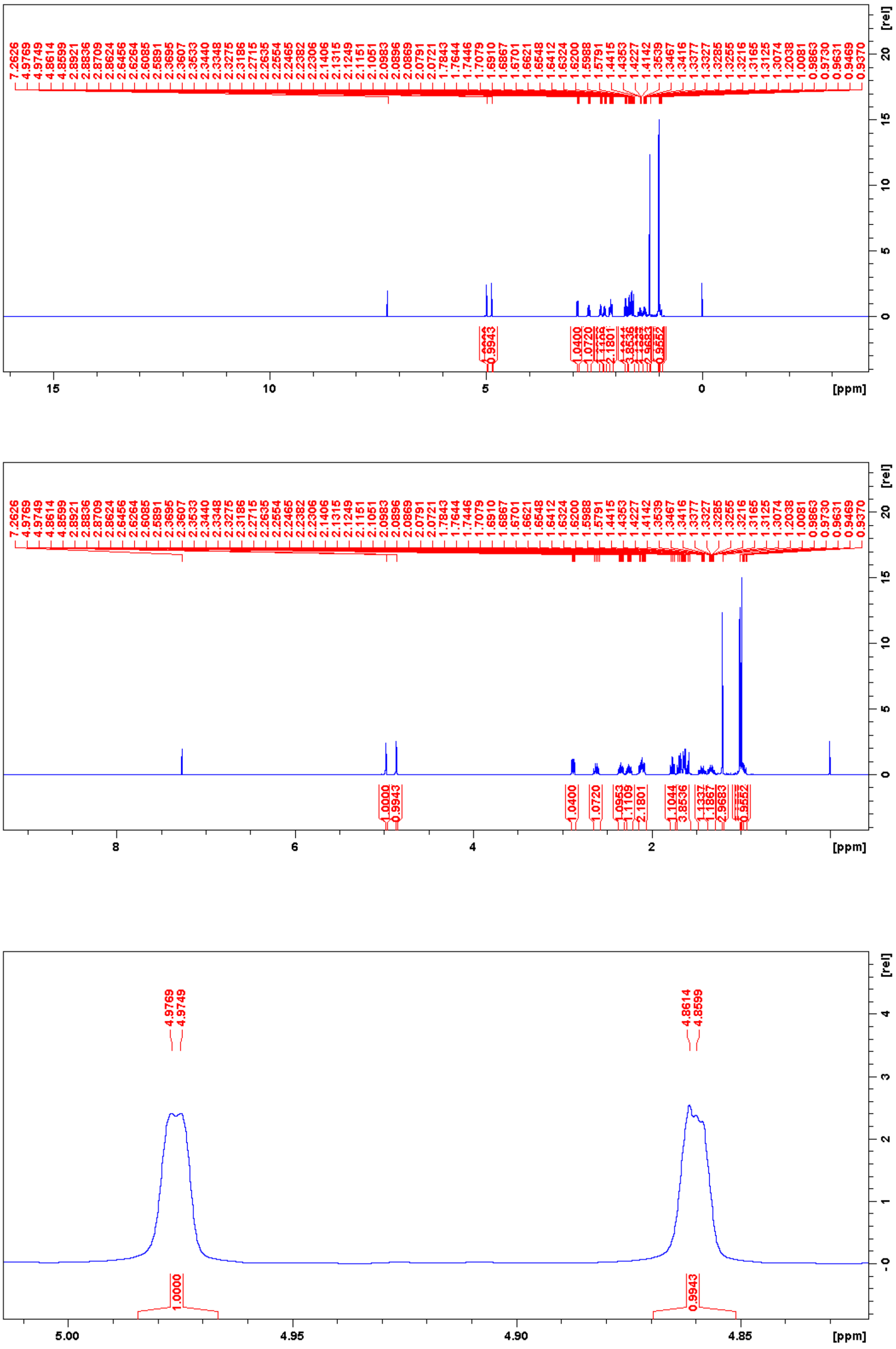

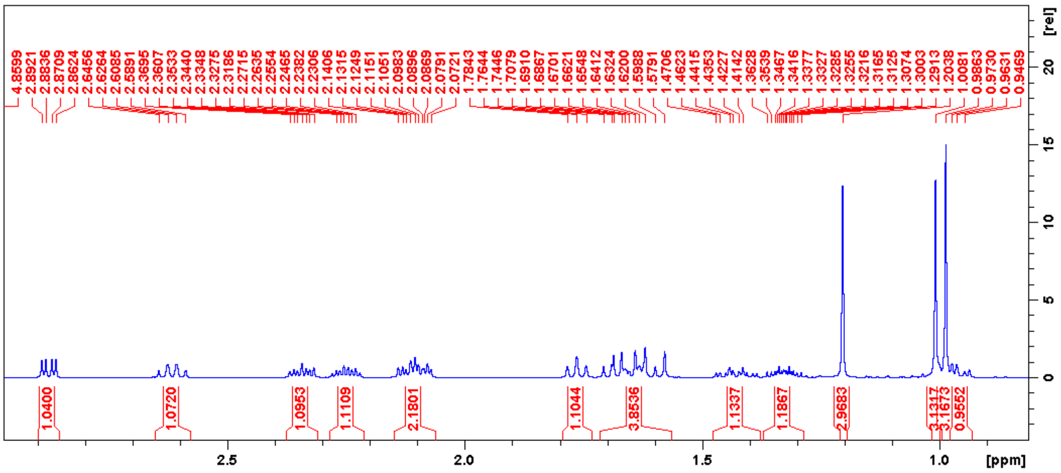
4.8599
2.8921
2.8836
2.8709
2.8624
2.6456
2.6264
2.6085
2.5891
2.3695
2.3607
2.3533
2.3440
2.3348
2.3275
2.3186
2.2715
2.2635
2.2554
2.2465
2.2382
2.2306
2.1406
2.1315
2.1249
2.1151
2.1051
2.0983
2.0896
2.0869
2.0791
2.0721
1.7843
1.7644
1.7446
1.7079
1.6910
1.6867
1.6701
1.6621
1.6548
1.6412
1.6324
1.6200
1.5988
1.5791
1.4706
1.4623
1.4415
1.4353
1.4227
1.4142
1.3628
1.3539
1.3467
1.3416
1.3377
1.3327
1.3285
1.3255
1.3216
1.3165
1.3125
1.3074
1.3003
1.2913
1.2038
1.0081
0.9863
0.9730
0.9631
0.9469
1.0400
1.0720
1.0953
1.1109
2.1801
1.1044
3.8536
1.1337
1.1867
2.9683
3.1317
3.1673
0.9552
[rel]
20
15
10
5
0
2.5
2.0
1.5
1.0
[ppm]

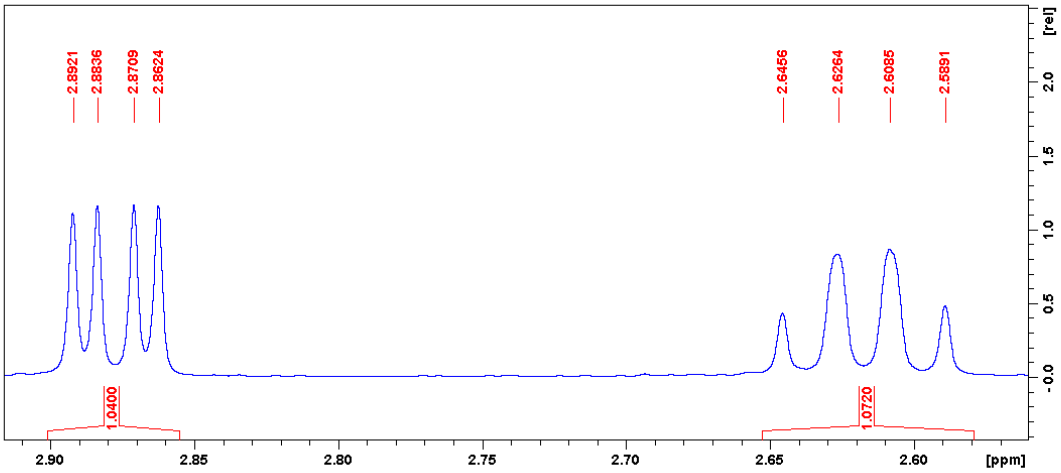
2.8921
2.8836
2.8709
2.8624
2.6456
2.6264
2.6085
2.5891
1.0400
1.0720
[rel]
2.0
1.5
1.0
0.5
-0.0
2.90
2.85
2.80
2.75
2.70
2.65
2.60
[ppm]

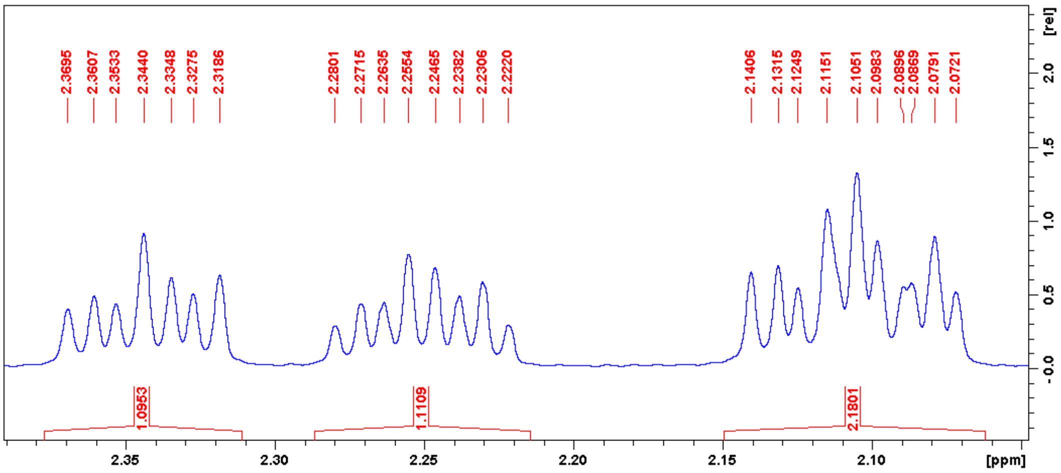
2.3695
2.3607
2.3533
2.3440
2.3348
2.3275
2.3186
2.2801
2.2715
2.2635
2.2554
2.2465
2.2382
2.2306
2.2220
2.1406
2.1315
2.1249
2.1151
2.1051
2.0983
2.0896
2.0869
2.0791
2.0721
1.0953
1.1109
2.1801
[rel]
2.0
1.5
1.0
0.5
-0.0
2.35
2.30
2.25
2.20
2.15
2.10
[ppm]

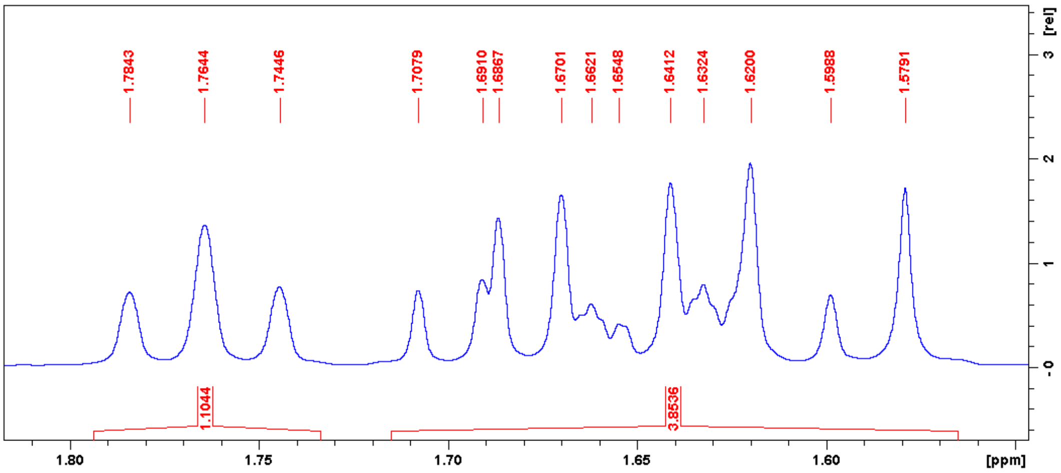
1.7843
1.7644
1.7446
1.7079
1.6910
1.6867
1.6701
1.6621
1.6548
1.6412
1.6324
1.6200
1.5988
1.5791
1.1044
3.8536
[rel]
[ppm]

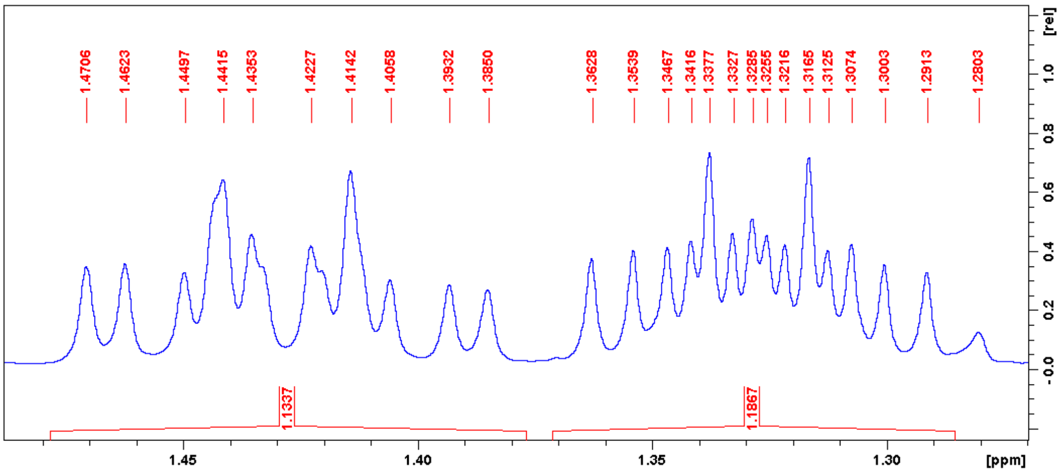
1.4706
1.4623
1.4497
1.4415
1.4353
1.4227
1.4142
1.4058
1.3932
1.3850
1.3628
1.3539
1.3467
1.3416
1.3377
1.3327
1.3285
1.3255
1.3216
1.3165
1.3125
1.3074
1.3003
1.2913
1.2803
1.1337
1.1867
[rel]
[ppm]

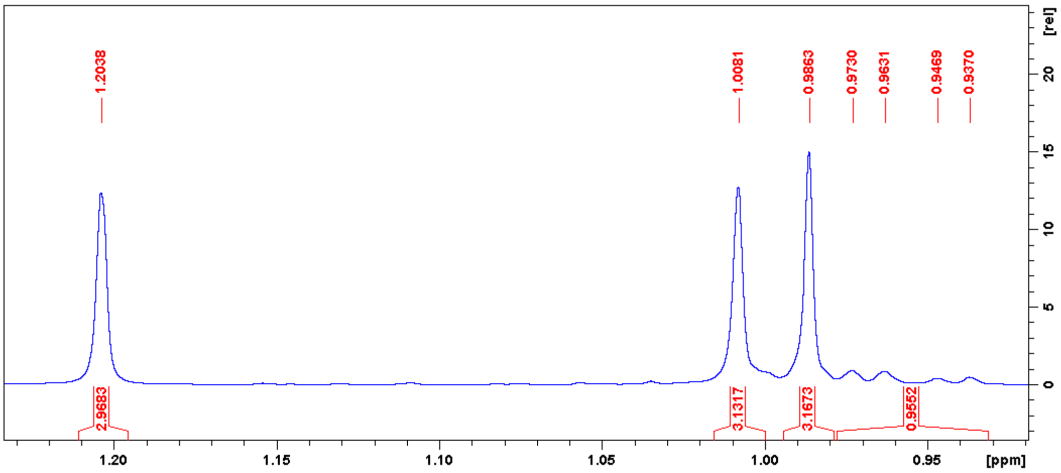
1.2038
1.0081
0.9863
0.9730
0.9631
0.9469
0.9370
2.9683
3.1317
3.1673
0.9552
[rel]
[ppm]

**Figure S 39**. $^{13}C$ NMR (125 MHz) spectrum of caryophyllene oxide in $CDCl_3$.

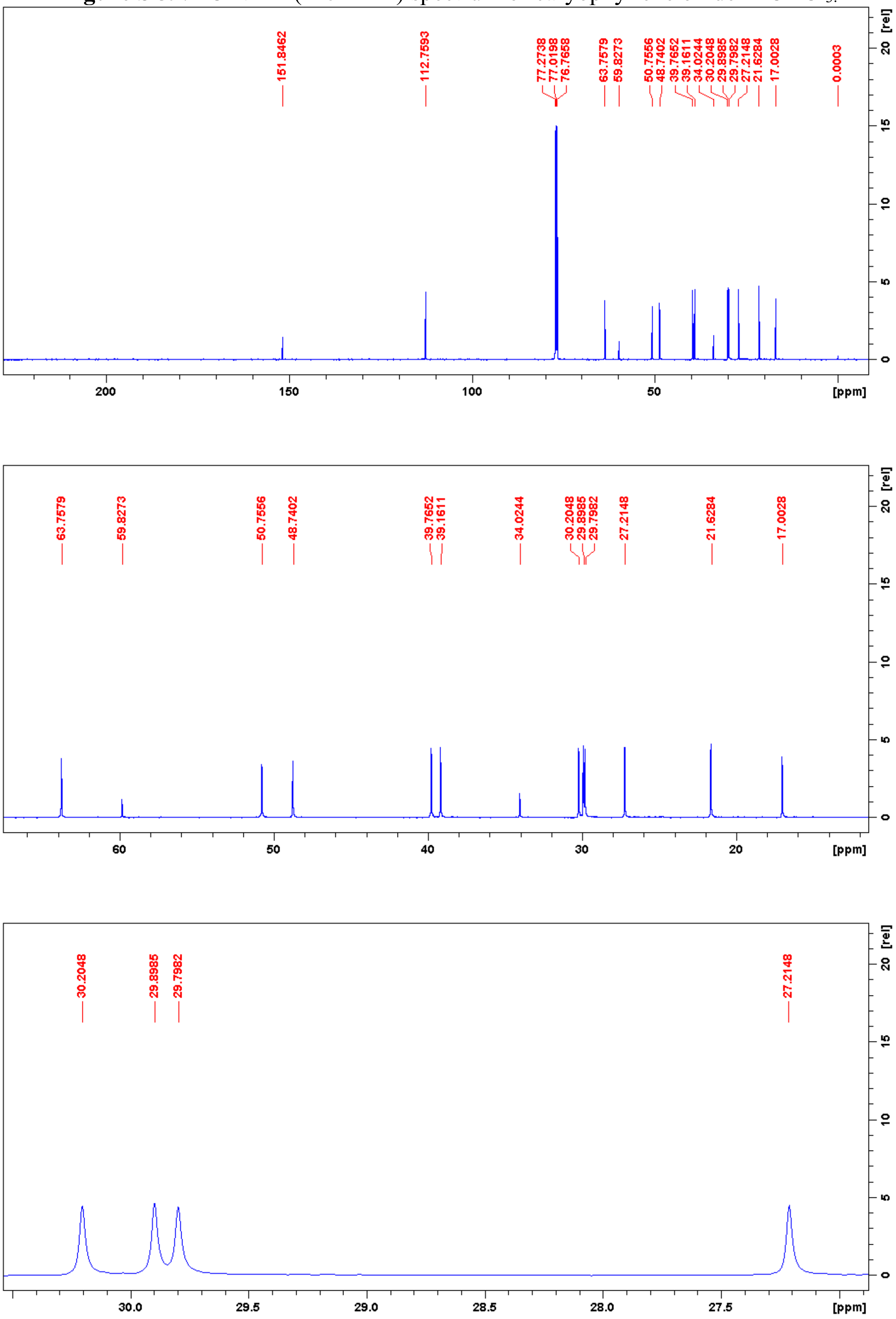

**Figure S 40.** $^{13}C$ NMR and DEPT 135 (125 MHz) spectrum of caryophyllene oxide in $CDCl_3$.

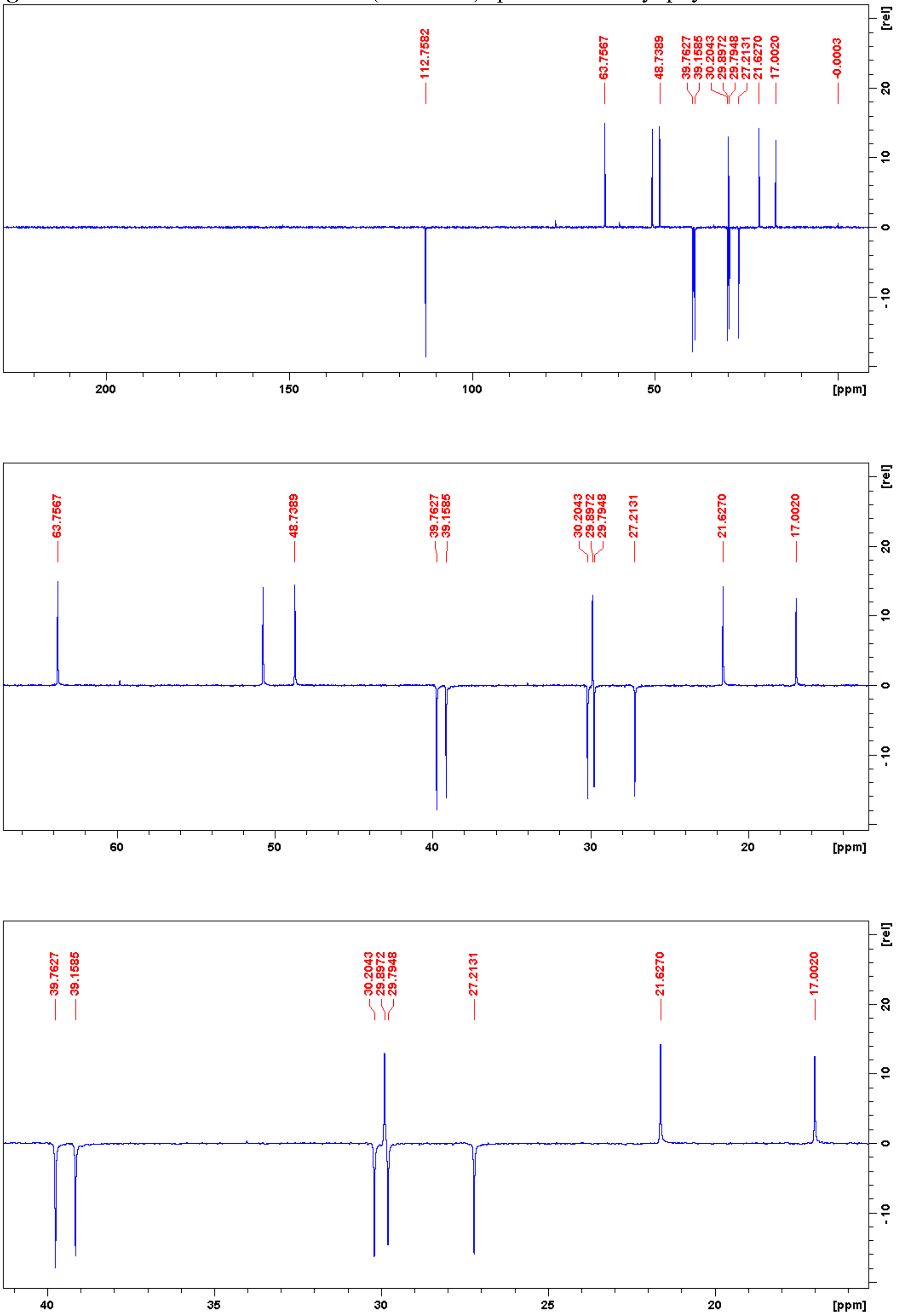

**Figure S 41.** $^{1}H$-$^{1}H$-COSY (500 MHz) spectrum of caryophyllene oxide in $CDCl_3$.

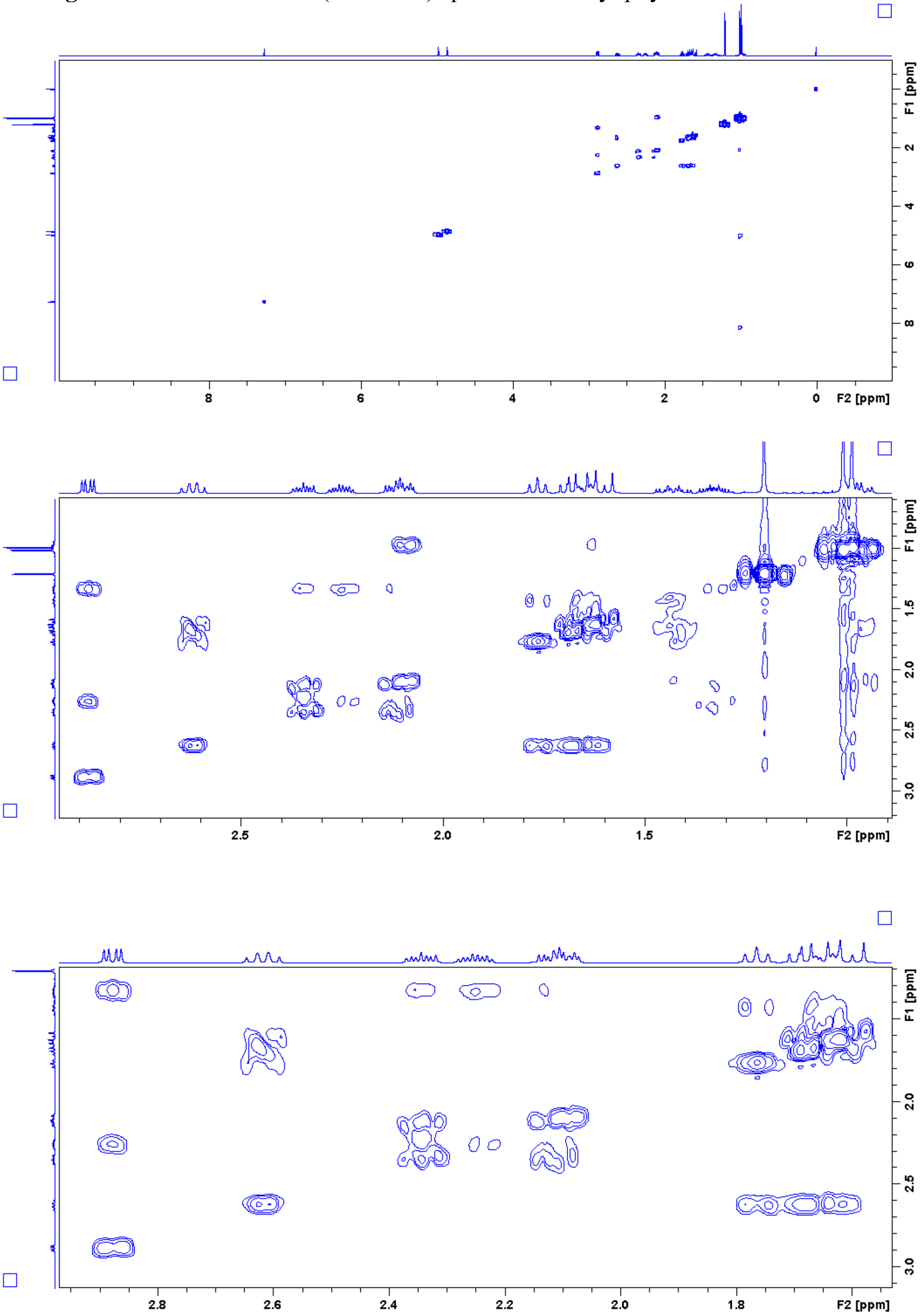

**Figure S 42.** $^{1}H$-$^{13}C$-HSQC ($^{1}H$ 500 MHz; $^{13}C$ 125 MHz) spectrum of caryophyllene oxide in $CDCl_3$.

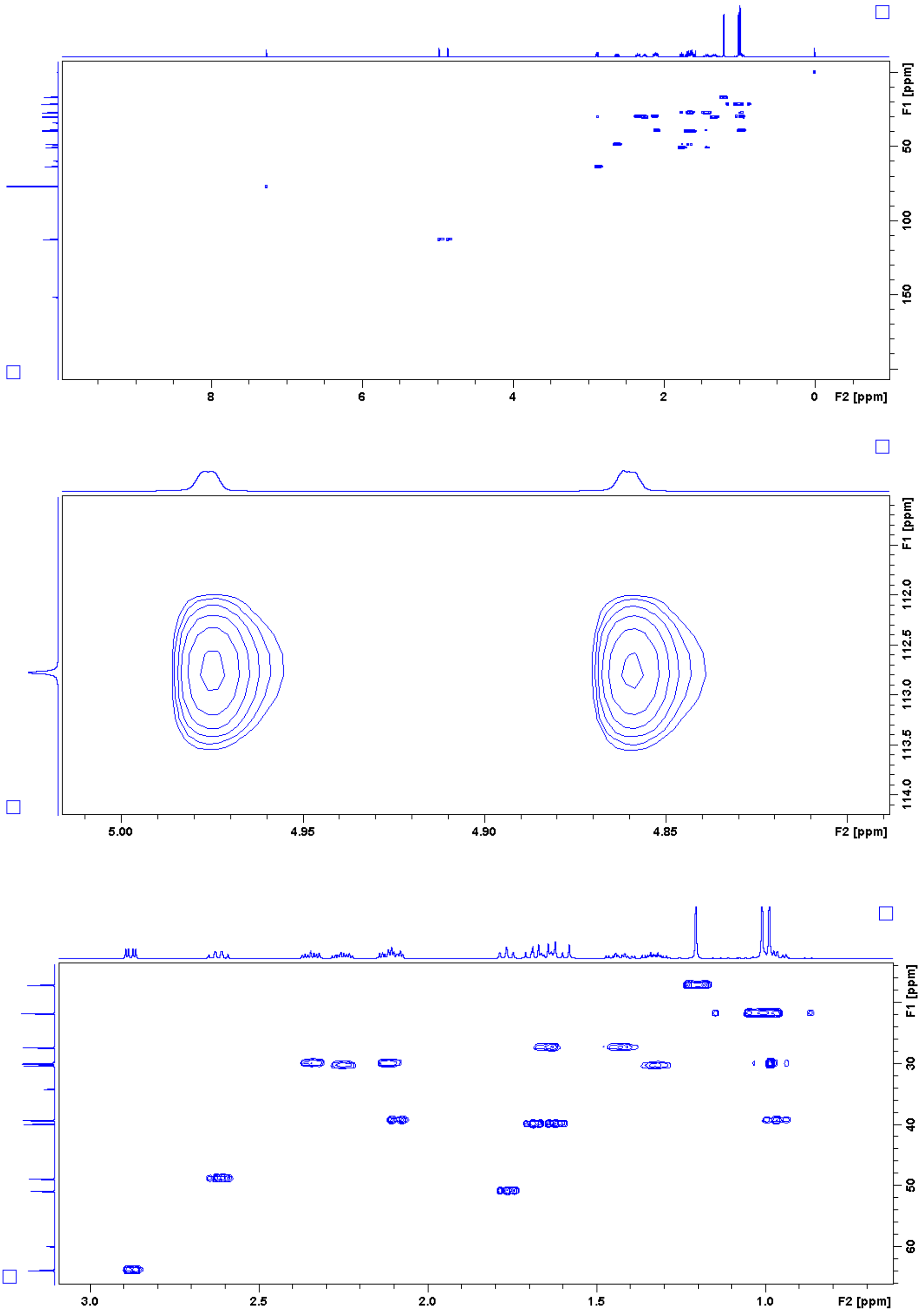

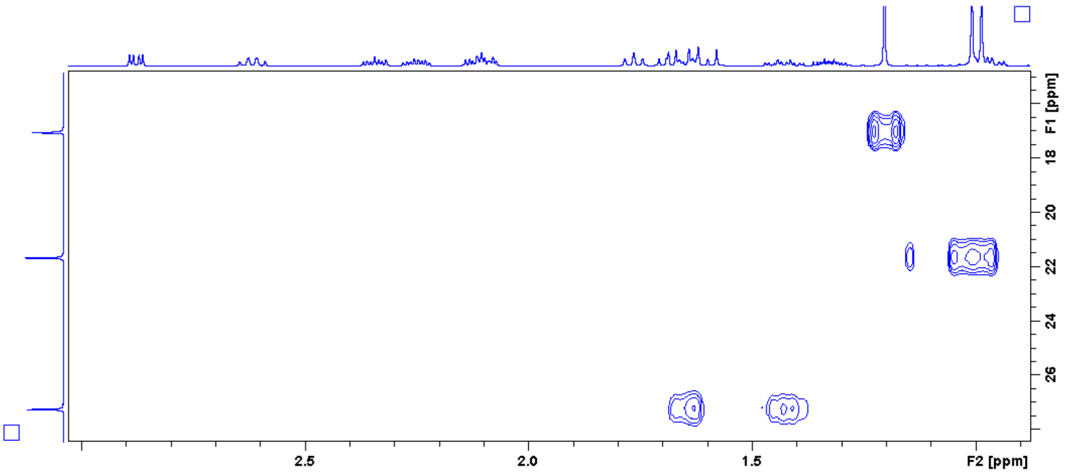
F1 [ppm]
18
20
22
24
26
2.5
2.0
1.5
F2 [ppm]

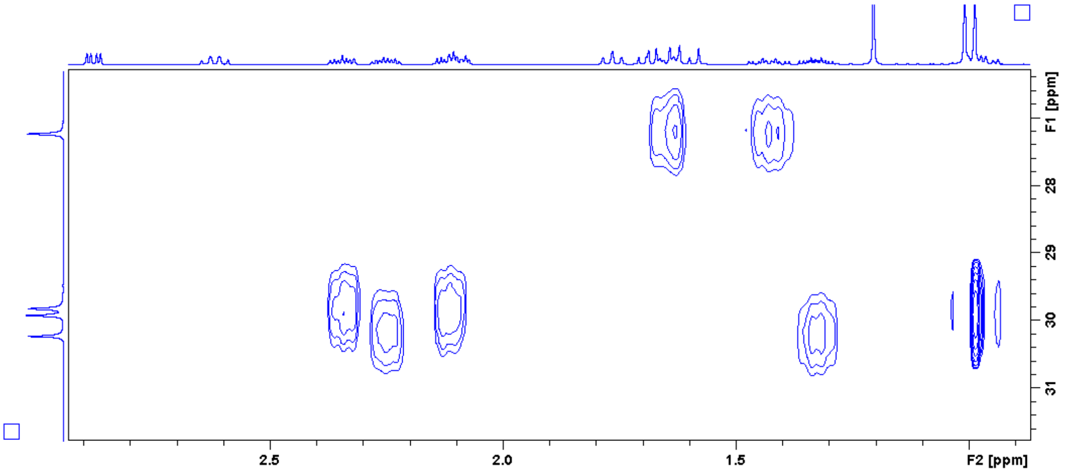
F1 [ppm]
28
29
30
31
2.5
2.0
1.5
F2 [ppm]

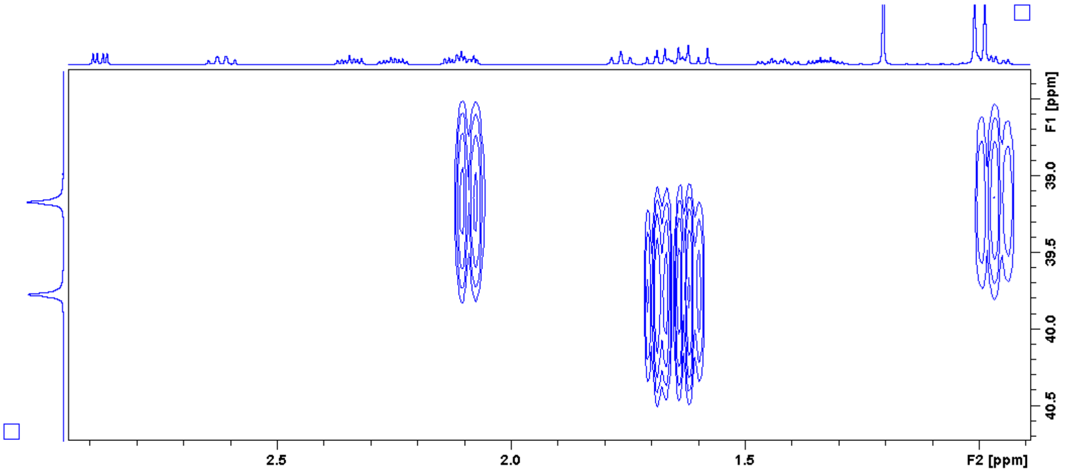
F1 [ppm]
39.0
39.5
40.0
40.5
2.5
2.0
1.5
F2 [ppm]

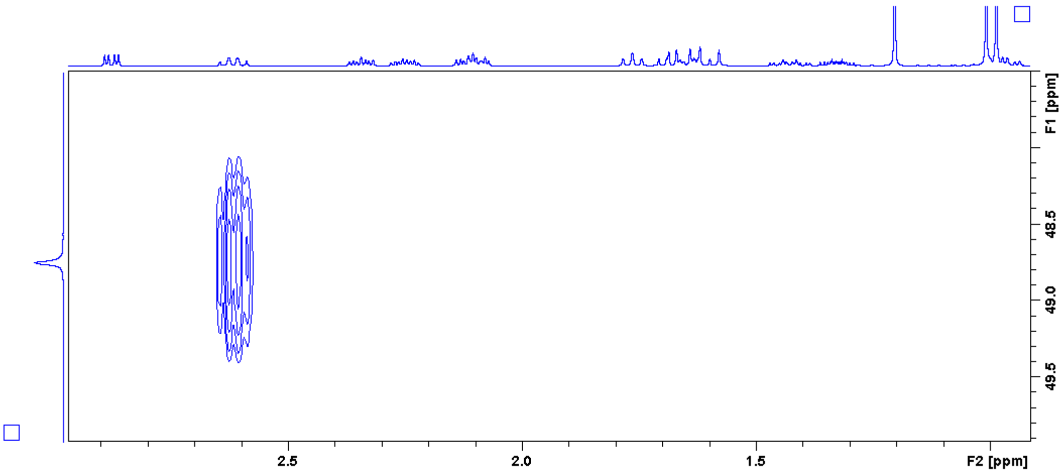
F1 [ppm]
48.5
49.0
49.5
2.5
2.0
1.5
F2 [ppm]

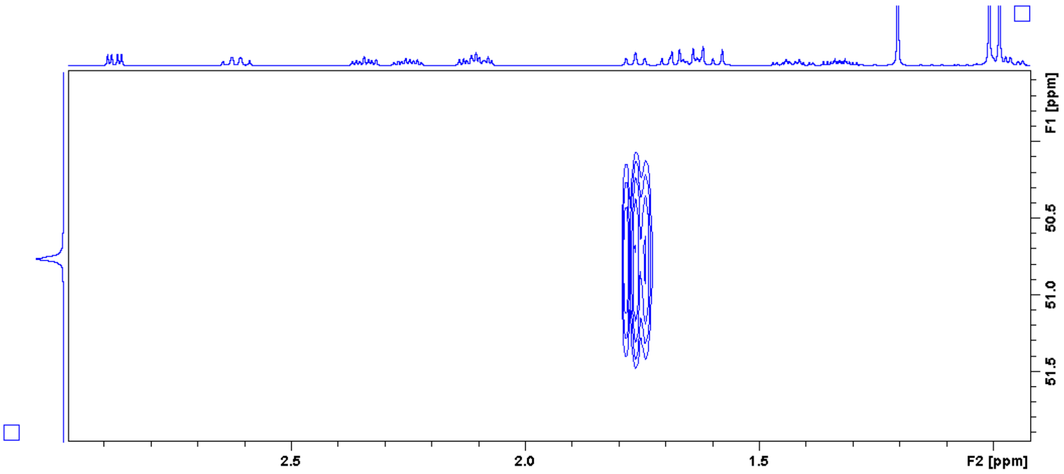
F1 [ppm]
50.5
51.0
51.5
2.5
2.0
1.5
F2 [ppm]

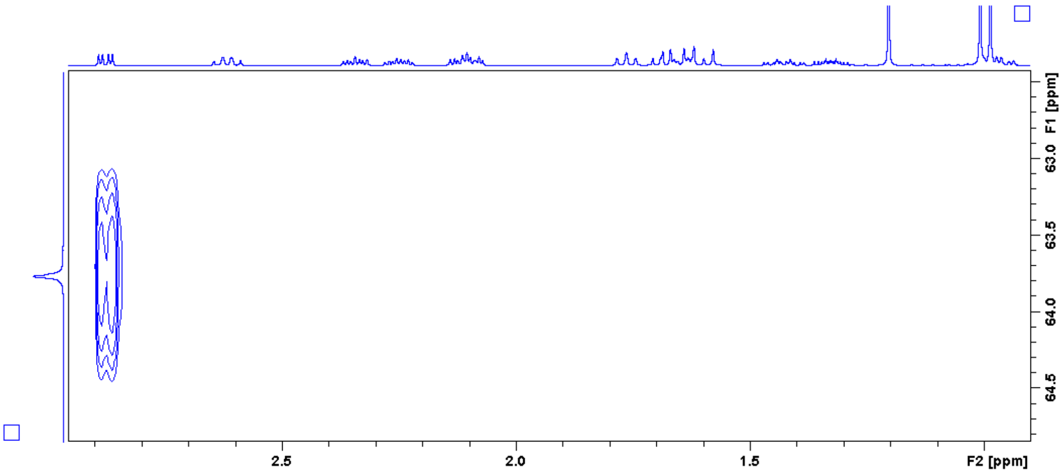
F1 [ppm]
63.0
63.5
64.0
64.5
2.5
2.0
1.5
F2 [ppm]

**Figure S 43.** $^{1}H$-$^{13}C$-HMBC ($^{1}H$ 500 MHz; $^{13}C$ 125 MHz) spectrum of caryophyllene oxide in $CDCl_3$

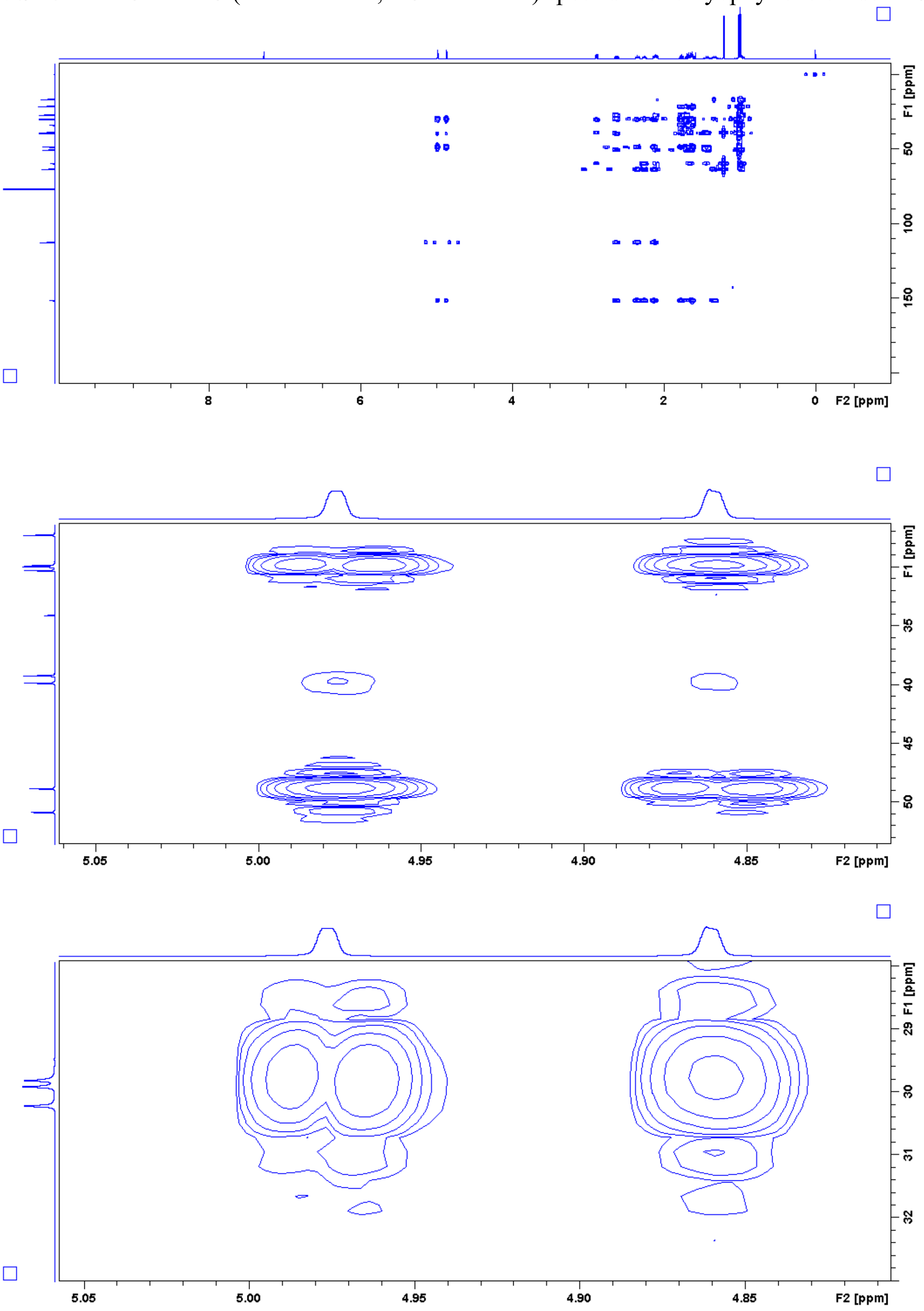

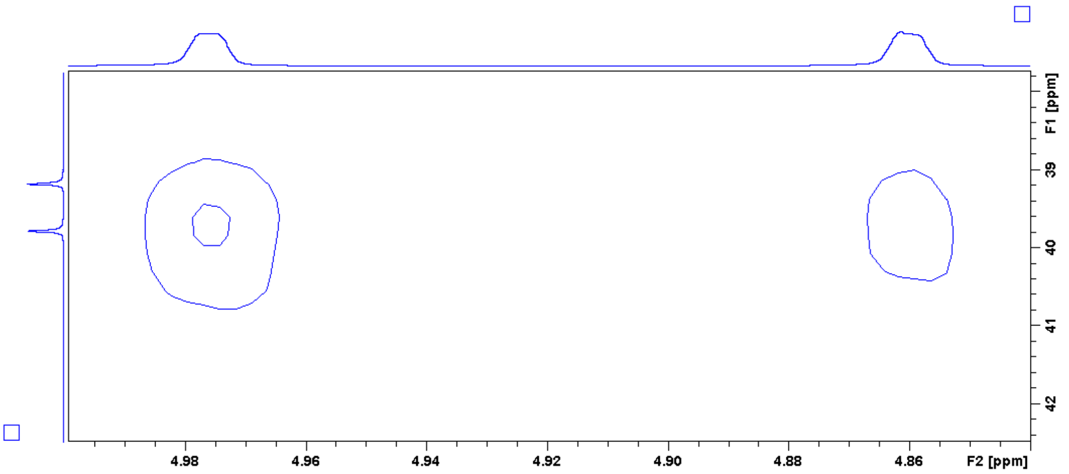

F1 [ppm]
39
40
41
42
4.98
4.96
4.94
4.92
4.90
4.88
4.86
F2 [ppm]


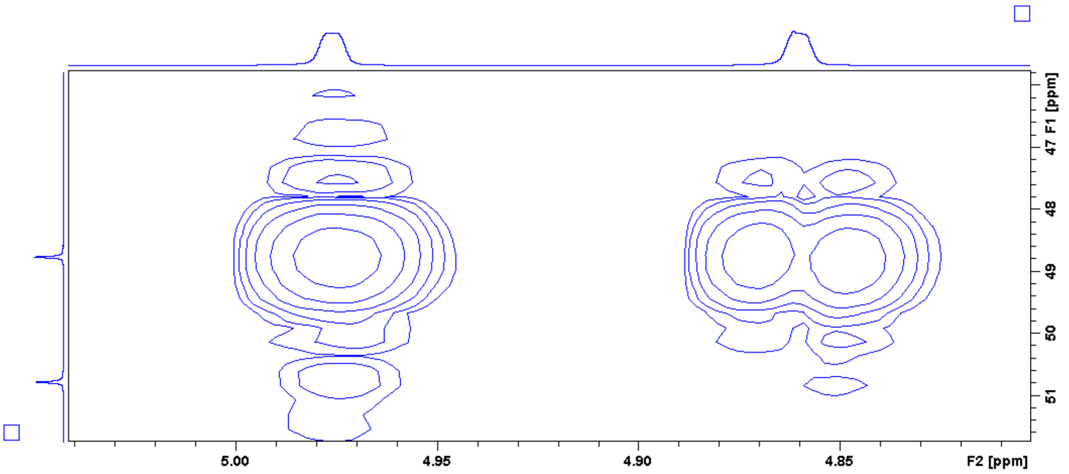

F1 [ppm]
47
48
49
50
51
5.00
4.95
4.90
4.85
F2 [ppm]


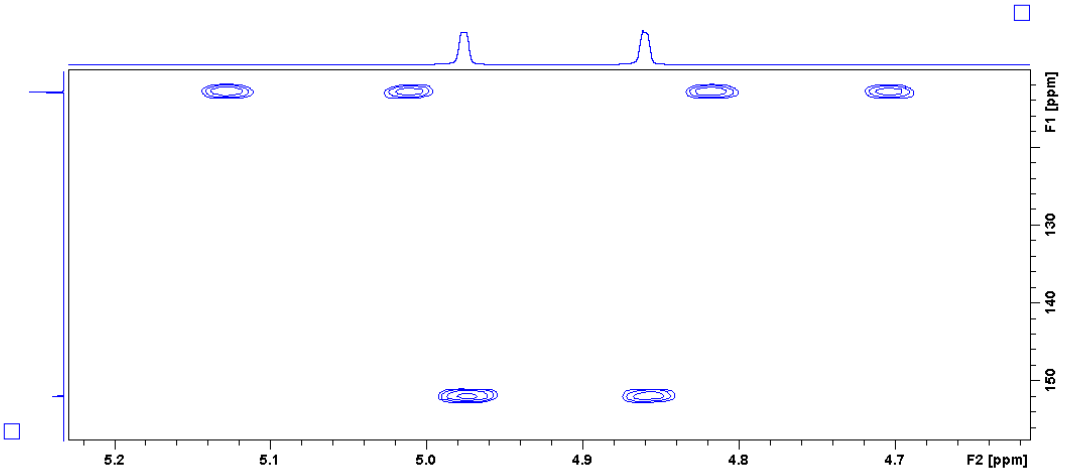

F1 [ppm]
130
140
150
5.2
5.1
5.0
4.9
4.8
4.7
F2 [ppm]

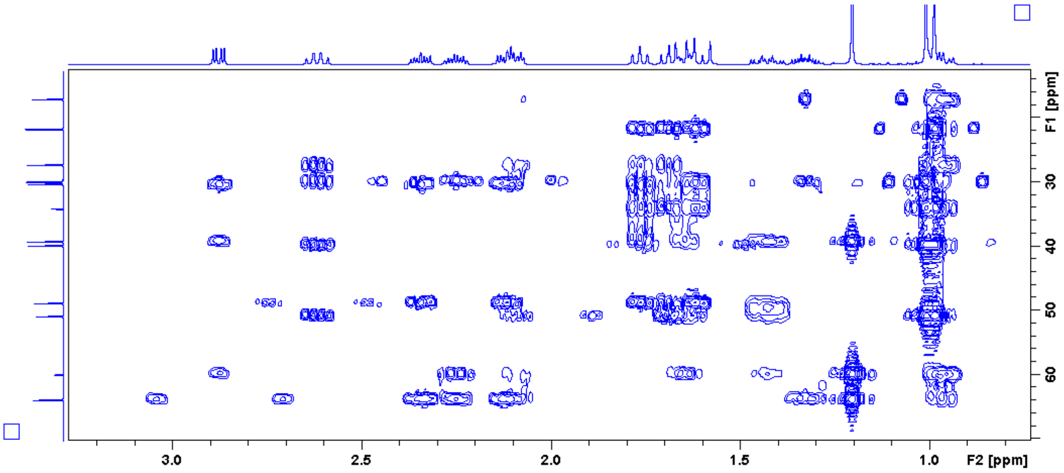
3.0
2.5
2.0
1.5
1.0
F2 [ppm]
F1 [ppm]
30
40
50
60

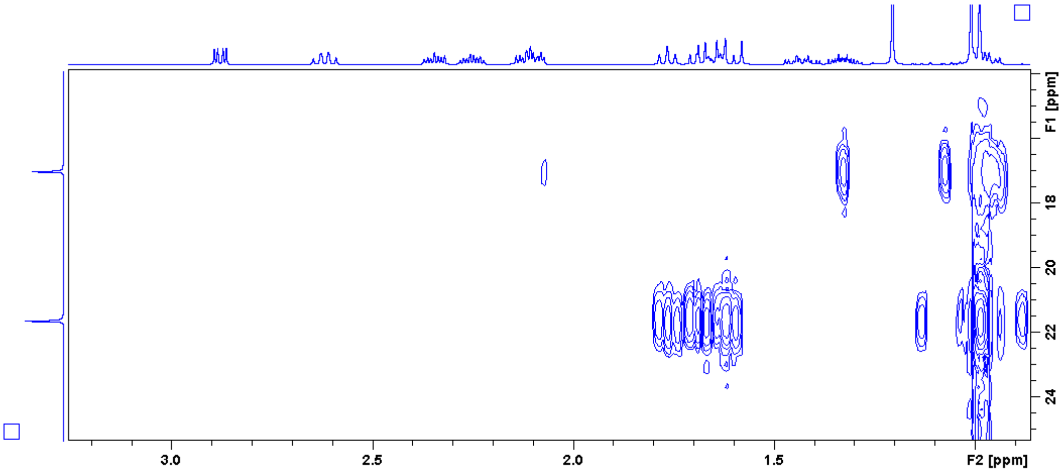
3.0
2.5
2.0
1.5
F2 [ppm]
F1 [ppm]
18
20
22
24

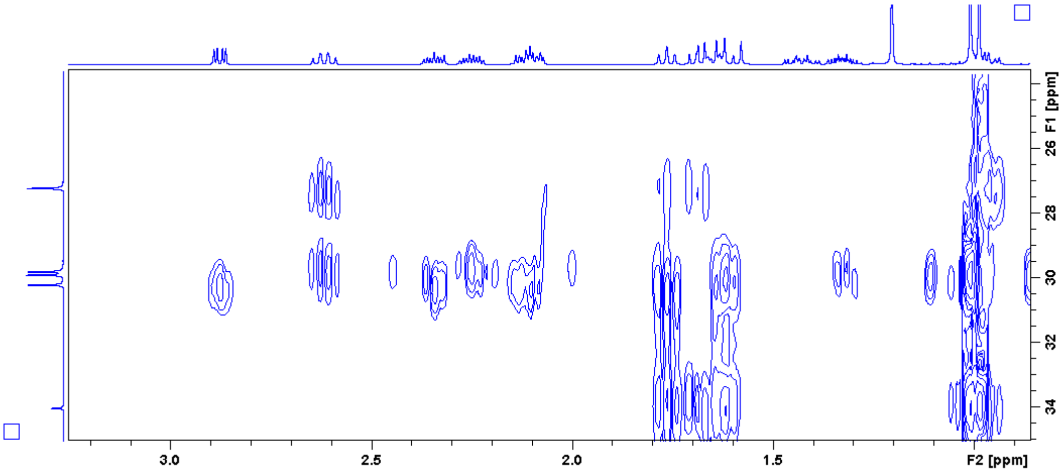
3.0
2.5
2.0
1.5
F2 [ppm]
F1 [ppm]
26
28
30
32
34

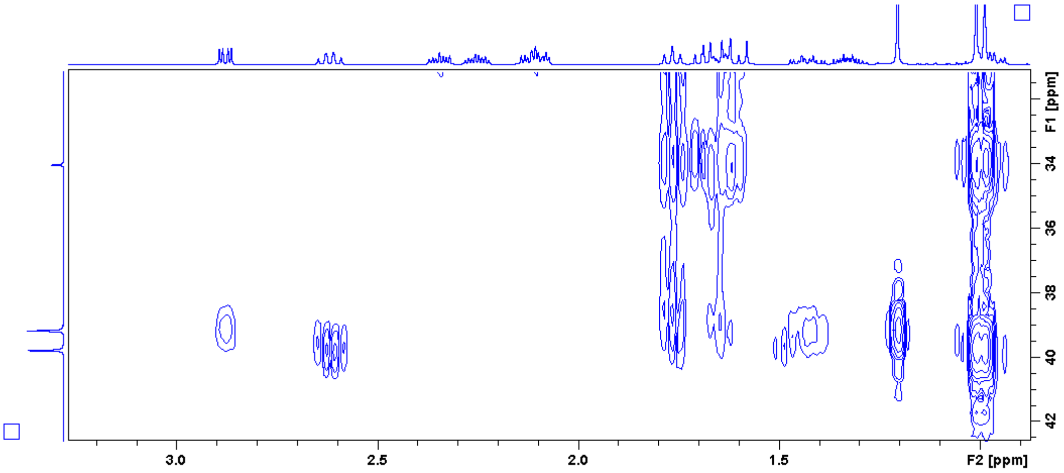
F1 [ppm]
34
36
38
40
42
3.0
2.5
2.0
1.5
F2 [ppm]

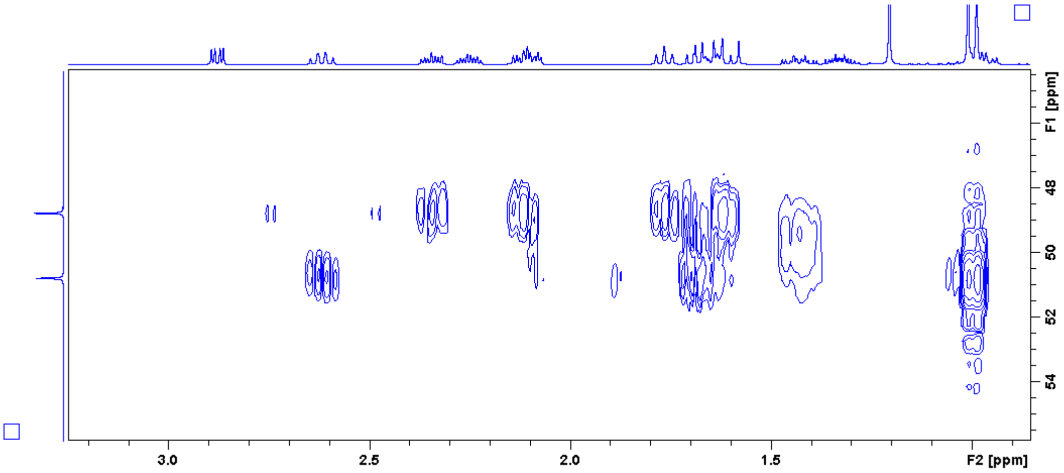
F1 [ppm]
48
50
52
54
3.0
2.5
2.0
1.5
F2 [ppm]

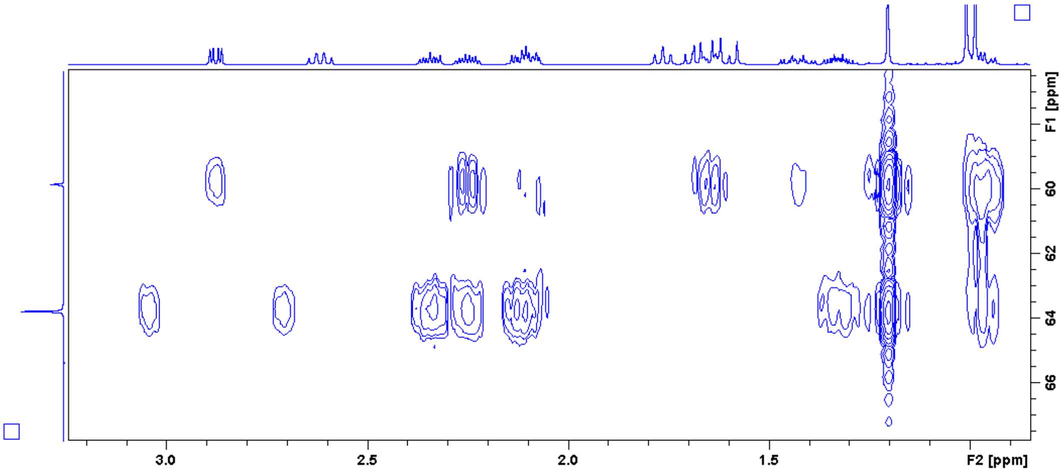
F1 [ppm]
60
62
64
66
3.0
2.5
2.0
1.5
F2 [ppm]

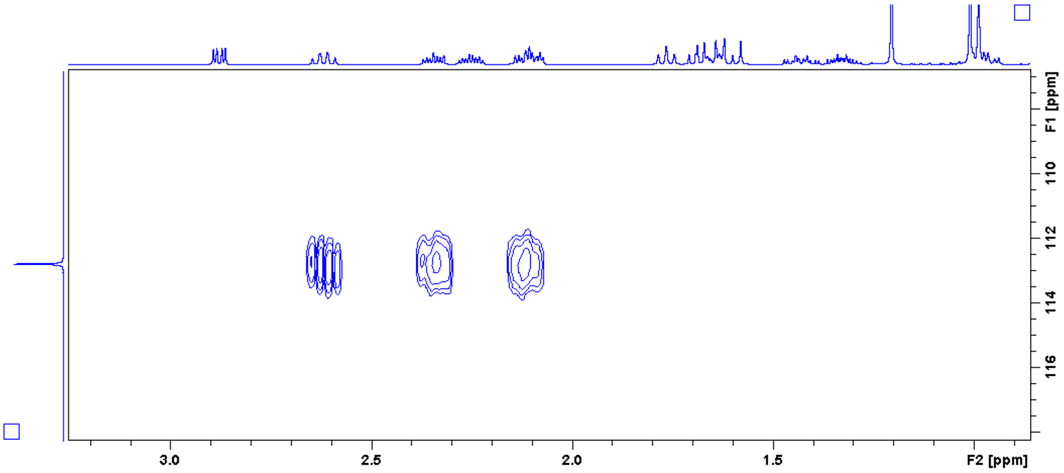
F1 [ppm]
110
112
114
116
3.0
2.5
2.0
1.5
F2 [ppm]

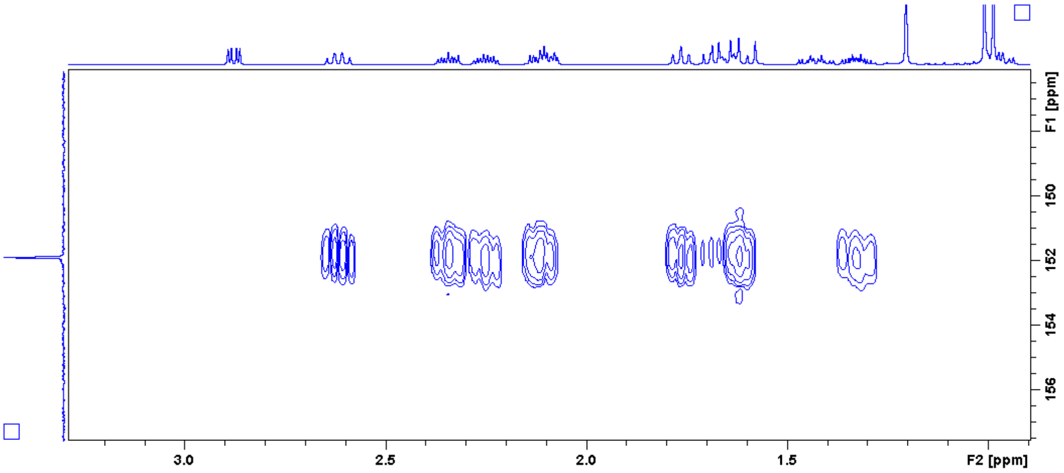
F1 [ppm]
150
152
154
156
3.0
2.5
2.0
1.5
F2 [ppm]

## Larvicidal Evaluation via SCD

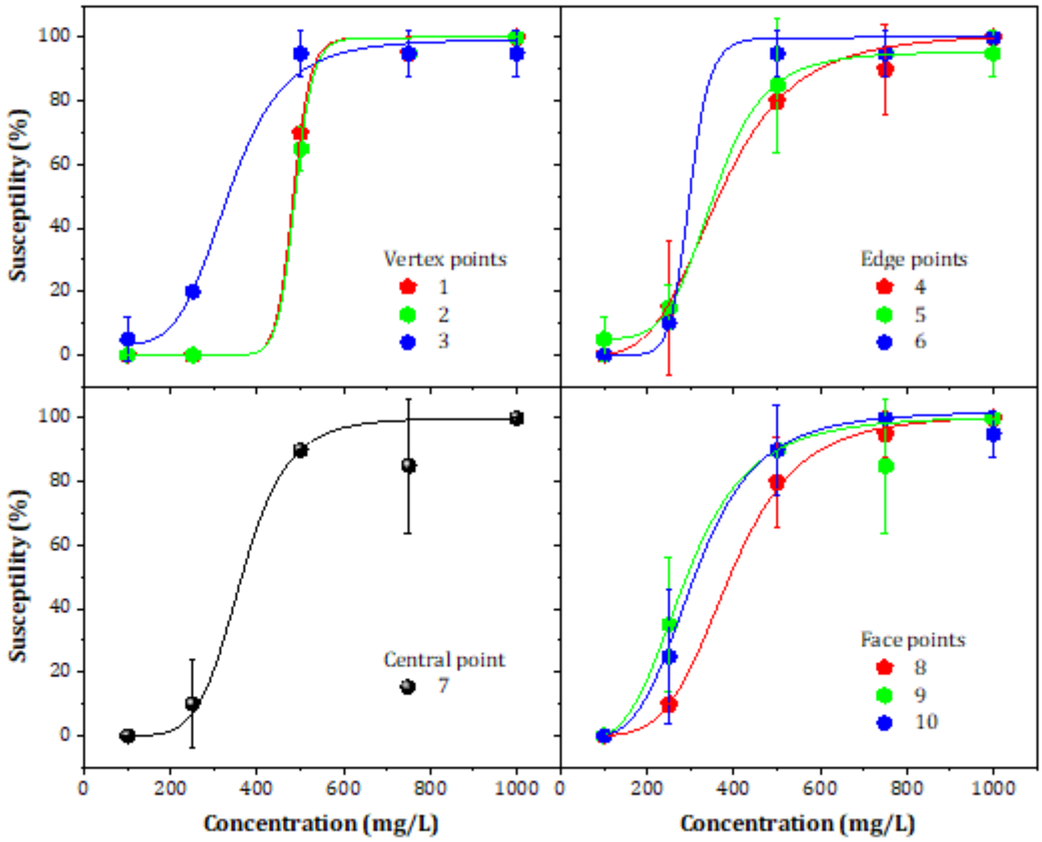


**Figure S 44.** Profile of the larvicidal activity of EO*Dr*-$PS_{80}$ interactions against *A. albopictus* in SCD.

## Larvicidal Evaluation Under Optimal Conditions

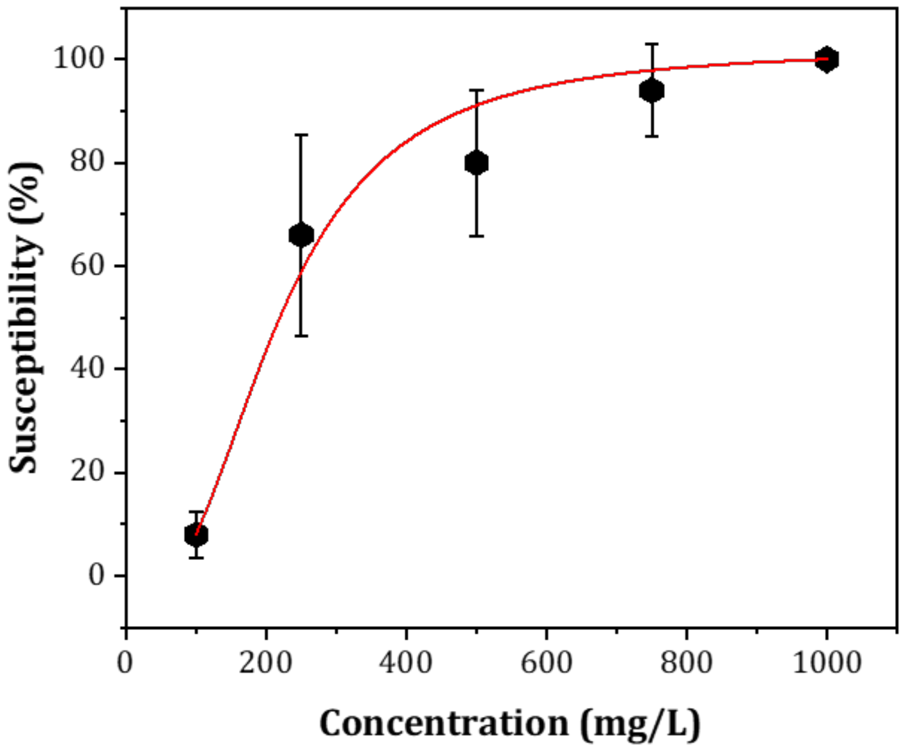


**Figure S 45.** Larvicidal profile against *A. albopictus* of EO*Dr*-$PS_{80}$ under optimal conditions ($LC_{50}$ 214.5±11.6 mg.$L^{-1}$; $LC_{90}$ 503.8±12.1 mg.$L^{-1}$; $R^2_{Aj}$ 0.997).

**Ecotoxicological Evaluation Under Optimal Conditions**

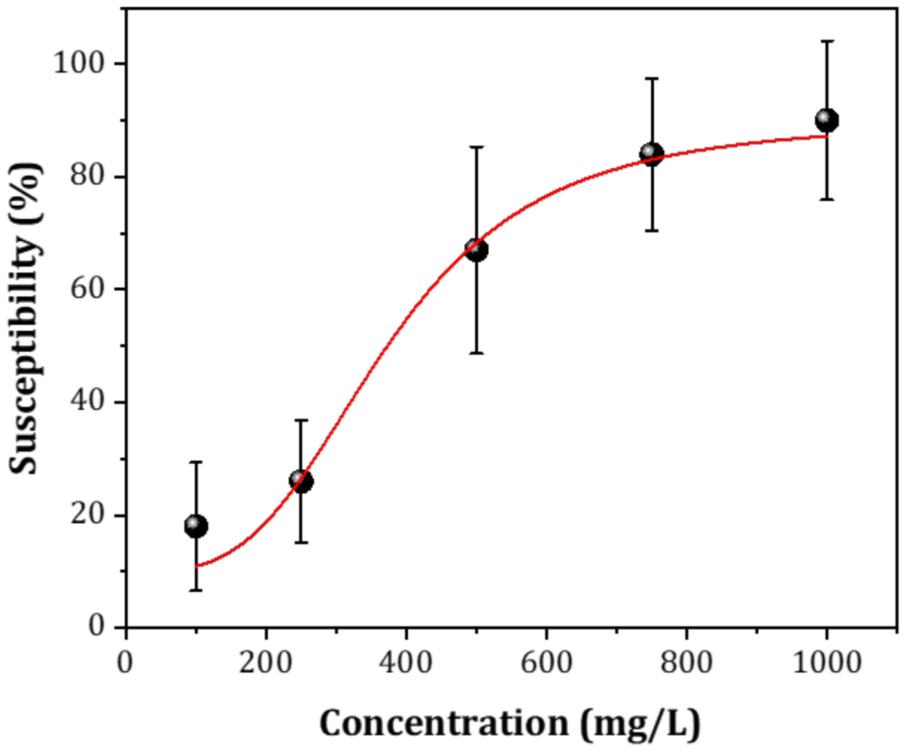


**Figure S 46.** Ecotoxicological profile against *A. salina* under optimal SCD conditions. $LC_{50}$ 372.8±27.2 mg.$L^{-1}$; $LC_{90}$ 716.2±102.7 mg.$L^{-1}$; $R^2_{Aj}$ 0.979.

## Chemical profile: $^1$H NMR and $^{13}$C NMR

**Table S 1**. NMR data for $^1$H (125 MHz; $CDCl_3$) of the standard samples fenchol, fenchyl acetate, caryophyllene, and caryophyllene oxide presented as chemical shifts (δ).

| Position | Fenchol | Fenchyl acetate | Caryophyllene | Caryophyllene oxide |
|---|---|---|---|---|
| 1 | - | - | 1.66 | 1.76 (t) |
| 2 | 3.25 (d) | 4.36 (d) | 1.51-1.40 (m) | 1.64-1.57 (m); 1.47-1.38 (m) |
| 3 | - | - | 2.35-2.31 (m); 2.02-1.97 (m) | 2.09-2.07 (m); 0.97-0.93 (m) |
| 4 | 1.68 (m) | 1.71-1.70 (m) | - | - |
| 5 | 1.60–1.57 (m); 1.04–1.00 (m) | 1.47-1.41 (m); 1.69-1.66 (m) | 5.32-5.29 (m) | 2.87 (q) |
| 6 | 1.67–1.61 (m); 1.42–1.38 (m) | 1.77–1.73 (m); 1.07–1.04 (m) | 2.10-2.06 (dt); 1.94-1.88 (m) | 2.28-2.22 (m); 1.36-1.28 (m) |
| 7 | 1.47–1.44 (dq); 1.13–1.11 (dd) | 1.59–1.57 (q); 1.19–1.16 (dd) | 2.22-2.17 (m); 2.00-1.98 (m) | 2.39-2.31 (m); 2.14-2.10 (m) |
| 8 | 1.08 (s) | 1.09 (s) | - | - |
| 9 | 0.99 (s) | 1.03 (s) | 2.35-2.31 (m) | 2.61 (q) |
| 10 | 0.86 (s) | 0.77 (s) | 1.62-1.56 (m) | 1.70-1.65 (m); 1.36-1.29 (m) |
| 11 | - | - | - | - |
| 12 | - | 2.07 (s) | 1.61 (s) | 1.20 (s) |
| 13 | - | - | 4.94 (s); 4.82 (s) | 4.97 (d); 4.85 (t) |
| 14 | - | - | 0.97 (s) | 0.98 (s) |
| 15 | - | - | 0.99 (s) | 1.0 (s) |

**Table S 2.** NMR data for $^{13}C$ (500 MHz; $CDCl_3$) of the standard samples fenchol, fenchyl acetate, caryophyllene, and caryophyllene oxide presented as chemical shifts (δ).

| Position | Fenchol | Fenchyl acetate | Caryophyllene | Caryophyllene oxide |
|---|---|---|---|---|
| 1 | 49.1 | 48.1 | 53.5 | 50.7 |
| 2 | 85.1 | 86.1 | 29.3 | 27.2 |
| 3 | 39.0 | 39.4 | 28.3 | 39.1 |
| 4 | 47.9 | 48.3 | 135.5 | 59.8 |
| 5 | 25.1 | 25.8 | 124.3 | 63.7 |
| 6 | 26.1 | 26.5 | 39.9 | 30.2 |
| 7 | 40.9 | 41.3 | 34.8 | 29.7 |
| 8 | 19.4 | 29.7 | 155.0 | 151.8 |
| 9 | 30.7 | 19.3 | 48.4 | 48.7 |
| 10 | 20.1 | 20.0 | 40.3 | 39.7 |
| 11 | - | 171.6 | 33.0 | 34.0 |
| 12 | - | 20.9 | 16.2 | 17.0 |
| 13 | - | - | 111.6 | 112.7 |
| 14 | - | - | 30.0 | 29.8 |
| 15 | - | - | 22.6 | 21.6 |

**Larvicidal Evaluation via SCD**

**Table S 3**. The mortality percentages of EO*Dr*-$PS_{80}$ for the determination of the larvicidal profiles and $LC_{50}$ and $LC_{90}$ against *A. albopictus* in SCD.

| Mixtures | 100 (mg.L$^{-1}$) | | 250 (mg.L$^{-1}$) | | 500 (mg.L$^{-1}$) | | 750 (mg.L$^{-1}$) | | 1000 (mg.L$^{-1}$) | |
|---|---|---|---|---|---|---|---|---|---|---|
| | % Mortality | | | | | | | | | |
| | $R_1$ | $R_2$ | $R_1$ | $R_2$ | $R_1$ | $R_2$ | $R_1$ | $R_2$ | $R_1$ | $R_2$ |
| $X_1$ | 0 | 0 | 0 | 0 | 70 | 70 | 90 | 100 | 100 | 100 |
| $X_2$ | 0 | 0 | 0 | 0 | 60 | 70 | 90 | 100 | 100 | 100 |
| $X_3$ | 10 | 0 | 20 | 20 | 90 | 100 | 90 | 100 | 90 | 100 |
| $X_4$ | 0 | 0 | 0 | 30 | 80 | 80 | 80 | 100 | 100 | 100 |
| $X_5$ | 10 | 0 | 20 | 10 | 70 | 100 | 90 | 100 | 90 | 100 |
| $X_6$ | 0 | 0 | 10 | 10 | 90 | 100 | 90 | 100 | 100 | 100 |
| $X_7$ | 0 | 0 | 0 | 20 | 90 | 90 | 70 | 100 | 100 | 100 |
| $X_8$ | 0 | 0 | 10 | 10 | 70 | 90 | 90 | 100 | 100 | 100 |
| $X_9$ | 0 | 0 | 20 | 50 | 90 | 90 | 70 | 100 | 100 | 100 |
| $X_{10}$ | 0 | 0 | 40 | 10 | 80 | 100 | 100 | 100 | 90 | 100 |

X: Mixture Experiments; R: Replicate; n: sample universe of 1.000 *A. albopictus* larvae.

**Table S 4.** Parameters for determining the predictive equation for the EO*Dr*-$PS_{80}$ interaction. The special cubic model is used as a reference for the SCD.

| **Component** | **β Coefficient** | **Standard Error** | **t value** | **Significance** |
|---|---|---|---|---|
| | | $LC_{50}$ | | |
| $X_1$ | 461.3 | 34.4 | 13.4 | $5.5 \times 10^{-9}$*** |
| $X_2$ | 445.3 | 34.4 | 12.9 | $8.4 \times 10^{-9}$*** |
| $X_3$ | 339.5 | 34.4 | 9.9 | $2.1 \times 10^{-7}$*** |
| $X_1X_2$ | –385.2 | 173.2 | –2.2 | $4.4 \times 10^{-2}$* |
| $X_1X_3$ | –220.4 | 173.2 | –1.2 | $2.2 \times 10^{-1}$ |
| $X_2X_3$ | –330.1 | 173.2 | –1.9 | $7.9 \times 10^{-2}$# |
| $X_1X_2X_3$ | 651.3 | 1141.9 | 0.6 | $5.8 \times 10^{-1}$ |
| | Determination coefficient | | $R^2$= 0.988 | $R^2_{Adj}$ = 0.981 |
| | | $LC_{90}$ | | |
| $X_1$ | 596.7 | 90.0 | 6.6 | $\times 10^{-5}$*** |
| $X_2$ | 649.6 | 90.0 | 7.2 | $\times 10^{-6}$*** |
| $X_3$ | 512.7 | 90.0 | 5.7 | $\times 10^{-5}$*** |
| $X_1X_2$ | –92.2 | 452.9 | –0.2 | $8.4 \times 10^{-1}$ |
| $X_1X_3$ | –55.6 | 452.9 | –0.1 | $9.0 \times 10^{-1}$ |
| $X_2X_3$ | –589.1 | 452.9 | –1.3 | $2.3 \times 10^{-1}$ |
| $X_1X_2X_3$ | 1490.7 | 2986.0 | 0.5 | $6.3 \times 10^{-1}$ |
| | Determination coefficient | | $R^2$= 0.965 | $R^2_{Adj}$ = 0.946 |

probabilistic significance at 0.001 (***); 0.05 (*) and 0.1 (#).

## REFERENCES


(1) Brandão CM, Cavalcante KSB, Teles RM, Marques GEC, Monteiro OS, Andrade EHA, Maia JGS. Composition and Larvicidal Activity of the Oil of Dizygostemon riparius (Plantaginaceae), a New Aromatic Species Occurring in Maranhão, Brazil. Chem Biodivers. **2020** Nov;17 (11): e2000462. Epub 2020 Oct 1. PMID: 32924270. DOI: 10.1002/cbdv.202000462.

(2) Santos AS; Alves SM; Figueiredo FJC; Rocha Neto OG. Descrição de sistema e de métodos de extração de óleos essenciais e determinação de umidade de biomassa em laboratório. Ministério da Agricultura, Pecuária e Abastecimento; Embrapa Amazônia Oriental. Comunicado Técnico 99, 1ª edição, ISSN 1517-2244, Novembro: Belém, PA, **2004**.

(3) Farmacopéia Brasileira IV, parte 1. 5. ed. São Paulo: Editora Atheneu, v. 1.320p, **2010**.

(4) Dool H van Den, Kratz P Dez, A generalization of the retention index system including linear temperature programmed gas-liquid partition chromatography, Journal of Chromatography A, 11, **1963**, 463-471, ISSN 0021-9673, DOI: https://doi.org/10.1016/S0021-9673(01)80947-X.

(5) Adams, R.P. Identification of Essential Oil Components by Gas Chromatography/Mass Spectroscopy, 4th ed.; Allured Publishing, Corp: Carol Stream, IL, USA, **2007**; 803p.

(6) Gutiérrez JM, González C, Maestro A, Solè I, Pey CM, Nolla J, Nano-emulsions: New applications and optimization of their preparation, Current Opinion in Colloid & Interface Science, 13, Issue 4, **2008**, 245-251, ISSN 1359-0294, DOI: https://doi.org/10.1016/j.cocis.2008.01.005.

(7) Erythropel HC, Zimmerman JB, Winter TM, Petitjean L, Melnikov F, Lam CH, Lounsbury AW, Mellor KE, Janković NZ, Tu Q, Pincus LN, Falinski MM, Shi W, Coish P, Plata DL, Anastas PT. (**2018**). The Green ChemisTREE: 20 years after taking root with the 12 principles. Green chemistry, 20 (9), 1929-1961. DOI: https://doi.org/10.1039/C8GC00482J.

(8) Soulaimani, B., Laghzaoui, E., Babram, M.A., Spooner-Hart, R.N., Hassani, L., & Abbad, A. (**2022**). Optimization of the insecticidal response of combined essential oils from Satureja alpina, Rosmarinus officinalis and Ammodaucus leucotrichus using a designed mixtures approach. International Journal of Tropical Insect Science, 42, 2501 - 2512. DOI:10.1007/s42690-022-00778-7, 2022.

(9) Wessal Ouedrhiri, Mounyr Balouiri, Samira Bouhdid, Sandrine Moja, Fouad Ouazzani Chahdi, Mustapha Taleb, Hassane Greche, Mixture design of Origanum compactum, Origanum majorana and Thymus serpyllum essential oils: Optimization of their antibacterial effect, Industrial Crops and Products, 89, **2016**, Pages 1-9, ISSN 0926-6690, DOI: https://doi.org/10.1016/j.indcrop.2016.04.049.

(10) Fadil M, Fikri-Benbrahim K, Rachiq S, Ihssane B, Lebrazi S, Chraibi M, Haloui T, Farah A. Combined treatment of Thymus vulgaris L., Rosmarinus officinalis L. and Myrtus communis L. essential oils against Salmonella typhimurium: Optimization of antibacterial activity by mixture design methodology. Eur J Pharm Biopharm. **2018** May; 126: 211-220. Epub 2017 Jun 3. PMID: 28583590. DOI: 10.1016/j.ejpb.2017.06.002.

(11) Lazcano Díaz E, Padilla Camberos E, Castillo Herrera GA, Estarrón Espinosa M, Espinosa Andrews H, Paniagua Buelnas NA, Gutiérrez Ortega A, Martínez Velázquez M. Development of essential oil-based phyto-formulations to control the cattle tick Rhipicephalus microplus using a mixture design approach. Exp Parasitol. **2019** Jun; 201:26-33. Epub 2019 Apr 25. PMID: 31029699. DOI: 10.1016/j.exppara.2019.04.008.

(12) Cornell, JA. A primer on experiments with mixtures. John Wiley & Sons, New Jersey, **2011**.
(13) Who (World Health Organization), Guidelines for laboratory and field testing of mosquito larvicides, WHO, Geneva, **2005**. Available in: http://apps.who.int/iris/bitstream/10665/69101/1/WHO_CDS_WHOPES_GCDPP_2005.13.pdf. Accessed on: 10 jan. 2025.
(14) Ministério da Saúde. Secretaria de Vigilância em Saúde e Ambiente. Departamento de Doenças Transmissíveis. Coordenação Geral de Vigilância de Arboviroses. Recomendações para o Manejo da Resistência do *Aedes aegypti* à inseticidas. Nota Informativa nº 29/**2024**-CGARB/DEDT/SVSA/MS (Temeguard 007-009): https://extranet.who.int/prequal/vector-control-products/temeguard. Ficha Técnica Temefós 1G Fersol. Available in: https://www.fersol.com.br/_files/ugd/437206_4ff9f1682cd846b9ac2d5231c3511aea.pdf. Accessed on: 23 fev. 2025.
(15) Consoli, RAGB.; Oliveira, RL. Principais mosquitos de importância sanitária no Brasil. Rio de Janeiro: editora FIOCRUZ, **1994**. 228 p.
(16) Associação Brasileira de Normas Técnicas. NBR. 16530:2016 - Ecotoxicologia Aquática - Toxicidade Aguda - Método de ensaio com *Artemia sp*. (Crustacea, Brachiopoda). São Paulo: ABNT, **2016**.
(17) Associação Brasileira de Normas Técnicas. NBR ISO IEC 17025:2017 - Requisitos gerais para a competência de laboratórios de ensaio e calibração. Rio de Janeiro: ABNT, **2017**.
(18) Lawson, J. Design and analysis of experiments with R. RCR Press, New York, **2015**.
(19) Lawson, J; Willden, C. Mixture Experiments in R Using mixexp. Journal of Statistical Software, v. 72, n. 2, 1-20, **2016**. DOI: https://doi.org/10.18637/jss.v072.c02.
